# Supplementary material for: Using Explainable Artificial Intelligence to Discover Interactions in an Ecological Model for Obesity
Source: Int J Environ Res Public Health. 2022 Aug 2;19(15):9447. doi: 10.3390/ijerph19159447 (PMC9367834; doi:10.3390/ijerph19159447)
Supplement: Supplementary file 1 [file ijerph-19-09447-s001.zip › Supplementary File S2-Data_Partioning_Rcode.pdf]

```
library(tidyverse)

options(scipen = 999) ### turn off scientific notation

# read in obesity data

source('~/Documents/abcd/Neighborhood_Household_Dynamics_NDA/source_scripts/wave_1_
whtr_sharma_10182021.R')

# load household data

# call in demographic data

demographic =
read_tsv("/home/ben/Documents/abcd/Neighborhood_Household_Dynamics_NDA/pdem02.txt")

# take out first row of data descriptions

demographic = tail(demographic,-1)

# replace missing data with NA

demographic[demographic == 777] <- NA
demographic[demographic == 999] <- NA

# demo_prnt_empl_time had too many missing values (4762)

parent_household = select(demographic,

subjectkey,demo_prnt_marital_v2,demo_prnt_ed_v2,demo_prnt_empl_v2,demo_prnt_income_v
2,demo_prnt_prtnr_v2,demo_comb_income_v2,demo_fam_exp1_v2,demo_fam_exp2_v2,demo
_fam_exp3_v2,demo_fam_exp4_v2,demo_fam_exp5_v2,demo_fam_exp6_v2,demo_fam_exp7
_v2,demo_roster_v2)

# self contains binary encodings for each race

self = select(demographic,
        subjectkey,
        demo_ethn_v2,
        demo_race_a_p___10,
        demo_race_a_p___11,
        demo_race_a_p___12,
        demo_race_a_p___13,
        demo_race_a_p___14,
```

```
        demo_race_a_p___15,
        demo_race_a_p___16,
        demo_race_a_p___17,
        demo_race_a_p___18,
        demo_race_a_p___19,
        demo_race_a_p___20,
        demo_race_a_p___21,
        demo_race_a_p___22,
        demo_race_a_p___23,
        demo_race_a_p___24,
        demo_race_a_p___25)

# set self variables as numeric

cols.num <- c(2:NCOL(self))
self[cols.num] <- sapply(self[cols.num],as.numeric)



# replace missing data with NA

parent_household[parent_household == 777] <- NA
parent_household[parent_household == 999] <- NA


# set parent household variables as numeric

cols.num <- c(2:NCOL(parent_household))
parent_household[cols.num] <- sapply(parent_household[cols.num],as.numeric)

# source residential script

source("/home/ben/Documents/abcd/Neighborhood_Household_Dynamics_NDA/source_scripts
/residential.R")

# remove redundant residential variables

residential = select(residential,
-c("reshist_addr1_p1tot","reshist_addr1_p1vlnt","reshist_addr1_drugtot","reshist_addr1_drgsale
","reshist_addr1_mjsale","reshist_addr1_drgposs","reshist_addr1_dui","reshist_addr1_adi_edu_l
","reshist_addr1_popdensity", "reshist_addr1_adi_in_dis", "reshist_addr1_adi_pov",
"reshist_addr1_adi_mortg", "reshist_addr1_adi_wsum"))
```

```
# merge environment with obesity data
# only keeping subjects with complete obesity data

obesogenic_conceptual = merge(x = bmi_wide, y = residential, by.y="subjectkey", by.x = "id",
all.x = TRUE)

# merge household data with obesogenic

obesogenic_conceptual = merge(x = obesogenic_conceptual, y = parent_household,
by.y="subjectkey", by.x = "id", all.x = TRUE)

# merge family id with obesogenic

source("/home/ben/Documents/abcd/Neighborhood_Household_Dynamics_NDA/source_scripts
/familyID.R")

obesogenic_conceptual = merge(
  x = obesogenic_conceptual,
  y = familyID,
  by.y= "id",
  by.x = "id", all.x = TRUE)

# merge obesogenic with self

obesogenic_conceptual = merge(
  x = obesogenic_conceptual,
  y = self,
  by.y= "subjectkey",
  by.x = "id", all.x = TRUE)

# make family id a factor for group folds

obesogenic_conceptual$rel_family_id = as.factor(obesogenic_conceptual$rel_family_id)

# source youth risk behavior scale

source("/home/ben/Documents/abcd/Neighborhood_Household_Dynamics_NDA/source_scripts
/youth_risk_behavior.R")

obesogenic_conceptual = merge(
  x = obesogenic_conceptual,
  y = yrb,
  by.y= "subjectkey",
  by.x = "id", all.x = TRUE)
```

```
# source and merge puberty

source("/home/ben/Documents/abcd/Neighborhood_Household_Dynamics_NDA/source_scripts
/pubertal_07282021.R")

obesogenic_conceptual = merge(
  x = obesogenic_conceptual,
  y = puberty,
  by.y= "id",
  by.x = "id", all.x = TRUE)

# source nutrition and merge

source("/home/ben/Documents/abcd/Neighborhood_Household_Dynamics_NDA/source_scripts
/nutrition_07282021.R")

obesogenic_conceptual = merge(
  x = obesogenic_conceptual,
  y = nutrition,
  by.y= "subjectkey",
  by.x = "id", all.x = TRUE)

# source sports and merge

source("/home/ben/Documents/abcd/Neighborhood_Household_Dynamics_NDA/source_scripts
/sports_activty.R")

saiq = select(saiq, subjectkey, sports_hours)

obesogenic_conceptual = merge(
  x = obesogenic_conceptual,
  y = saiq,
  by.y= "subjectkey",
  by.x = "id", all.x = TRUE)

# source developmental history and merge

source("/home/ben/Documents/abcd/Neighborhood_Household_Dynamics_NDA/source_scripts
/developmental_hist.R")

dhx = select(dhx, -c("devhx_8_coc_crack", "devhx_9_coc_crack", "devhx_8_her_morph",
"devhx_9_her_morph", "devhx_8_oxycont", "devhx_9_oxycont", "devhx_8_other_drugs",
"devhx_9_other_drugs"))
```

```
obesogenic_conceptual = merge(  x = obesogenic_conceptual,   y = dhx,   by.y= "subjectkey",
by.x = "id", all.x = TRUE)


# count missing values for each variable

missing_sum = data.frame(raw_sum = colSums(is.na(obesogenic_conceptual)))

missing_sum$propotion = missing_sum$raw_sum / NROW(obesogenic_conceptual)

## begin creating separate folds
# randomly sort data
# split data into folds, stratified by family & balanced by whtr

# Set seed
set.seed(23322)

# Shuffle row indices: rows
rows <- sample(nrow(obesogenic_conceptual))

# Randomly order data
obesogenic_conceptual <- obesogenic_conceptual[rows, ]

# create stratified folds

obesogenic_conceptual_folded = groupdata2::fold(
  data = obesogenic_conceptual,
  k = 2,
  num_col = 'whtr_sharma',
  id_col = 'rel_family_id')


# remove unwanted variables

obesogenic_conceptual = select(obesogenic_conceptual_folded, -c(id, rel_family_id,
eventname))

# remove unwanted dataframes

rm(bmi_wide,demographic,familyID,obesogenic_conceptual_folded,parent_household,residenti
al, rows, self, nutrition, puberty, yrb, missing_sum,saiq,i,dhx,bmi_clean)

# make all binary variables 0 or 1
```

```
obesogenic_conceptual$demo_prnt_prtnr_v2 = obesogenic_conceptual$demo_prnt_prtnr_v2 -
1
obesogenic_conceptual$demo_ethn_v2 = obesogenic_conceptual$demo_ethn_v2 - 1

saveRDS(obesogenic_conceptual,
"/home/ben/Documents/abcd/Neighborhood_Household_Dynamics_NDA/cross_sectional_medi
an/obesogenic_data_2fold_50_50_01152022.rds")
```
