# Supplementary material for: Using Explainable Artificial Intelligence to Discover Interactions in an Ecological Model for Obesity
Source: Int J Environ Res Public Health. 2022 Aug 2;19(15):9447. doi: 10.3390/ijerph19159447 (PMC9367834; doi:10.3390/ijerph19159447)
Supplement: Supplementary file 1 [file ijerph-19-09447-s001.zip › Supplementary File S3-waist_to_height_ratio_zscore_Rcode.pdf]

```
# compute age and sex adujusted z-scores for bmi and waist circumference

library(tidyverse)
library(AGD)
library(childsds)

options(scipen = 999) ### turn off scientific notation

# load anthropometric data

anthropometric_data =
read_tsv("/home/ben/Documents/abcd/Neighborhood_Household_Dynamics_NDA/abcd_ant01.
txt")

# select subject id, event name (data collection wave), sex, age in months, height (inches),
weight (lbs), waist circumference (in)

anthropometric_data = anthropometric_data[-1,] %>%
  select(id = subjectkey, time = eventname, sex = sex, agemos = interview_age, height_in =
anthroheightcalc, weight_lbs = anthroweightcalc, waist = anthro_waist_cm)

# convert sex to a factor

anthropometric_data$sex = as.factor(anthropometric_data$sex)

# convert age, height, weight, and waist to numeric

anthropometric_data[4:7] <- lapply(anthropometric_data[4:7], as.numeric)

# create new version of age in years

anthropometric_data$ageyears = anthropometric_data$agemos / 12

# compute bmi
# compute height in cm, weight in kg
# convert waist circumference from inches to cm

anthropometric_data = anthropometric_data %>% mutate(
  bmi = 703 * weight_lbs / height_in ^ 2,
  height_cm = height_in * 2.54,
  weight_kg = weight_lbs * 0.453592,
  waist = waist * 2.54)
```

```
### use AGD package to compute BMI z-scores
##  https://stefvanbuuren.github.io/AGD/

bmi_levels <- c("Underweight",
                "Healthy weight",
                "Overweight",
                "Obesity",
                "Severe obesity")

agd_df <- anthropometric_data %>%
  mutate(
        heightz =  AGD::y2z(
        y = height_cm, x = ageyears, sex = sex, ref = cdc.hgt),
        weightz =  AGD::y2z(
        y = weight_kg, x = ageyears, sex = sex, ref = cdc.wgt),
        bmiz =  AGD::y2z(
        y = bmi, x = ageyears, sex = sex, ref = cdc.bmi),
        bmi_pct = pnorm(bmiz),
        # what is the 95th percentile for the child's age and sex?,
        z = qnorm(.95),
        bmi_p95 = AGD::z2y(z = z, x= ageyears, sex = sex, ref = cdc.bmi),
        # percentage of the 95th percentile
        bmi_pct95 = bmi/bmi_p95,
        # bmi category
        bmi_cat = factor(case_when(
        bmi_pct < .05 ~ "Underweight",
        bmi_pct < .85 ~ "Healthy weight",
        bmi_pct < .95 ~ "Overweight",
        bmi_pct95 < 1.2 ~ "Obesity",
        bmi_pct95 >= 1.2 ~ "Severe obesity"),
        levels = bmi_levels)
  )

# use childsds package to calculate z-scores for waist circumference and waist / height ratio

agd_df$wc_sharma = sds(
  value = agd_df$waist,
  age = agd_df$ageyears,
  sex = agd_df$sex, male = 'M', female = "F",
  ref = us.ref, item = "wc_sharma")
```

```
agd_df$whtr_sharma = sds(
  value = agd_df$waist / agd_df$height_cm,
  age = agd_df$ageyears,
  sex = agd_df$sex, male = 'M', female = "F",
  ref = us.ref, item = "whtr_sharma")

# remove outliers that have z-score +/- 4 for height, weight, waist-height ratio, or BMI

bmi_clean = filter(agd_df, bmiz > -4 & bmiz < 4 & heightz > -4 & heightz < 4 & weightz > -4 &
weightz < 4 & whtr_sharma > -4 & whtr_sharma < 4 )

# just include baseline and 1-year follow-up

# remove temporary data.frames

rm(anthropometric_data,agd_df,bmi_levels)
```
