# Supplementary material for: Using Explainable Artificial Intelligence to Discover Interactions in an Ecological Model for Obesity
Source: Int J Environ Res Public Health. 2022 Aug 2;19(15):9447. doi: 10.3390/ijerph19159447 (PMC9367834; doi:10.3390/ijerph19159447)
Supplement: Supplementary file 1 [file ijerph-19-09447-s001.zip › Supplementary File S1-missing_data_convergence_plots_and_Rcode.pdf]

Data Partition # 1

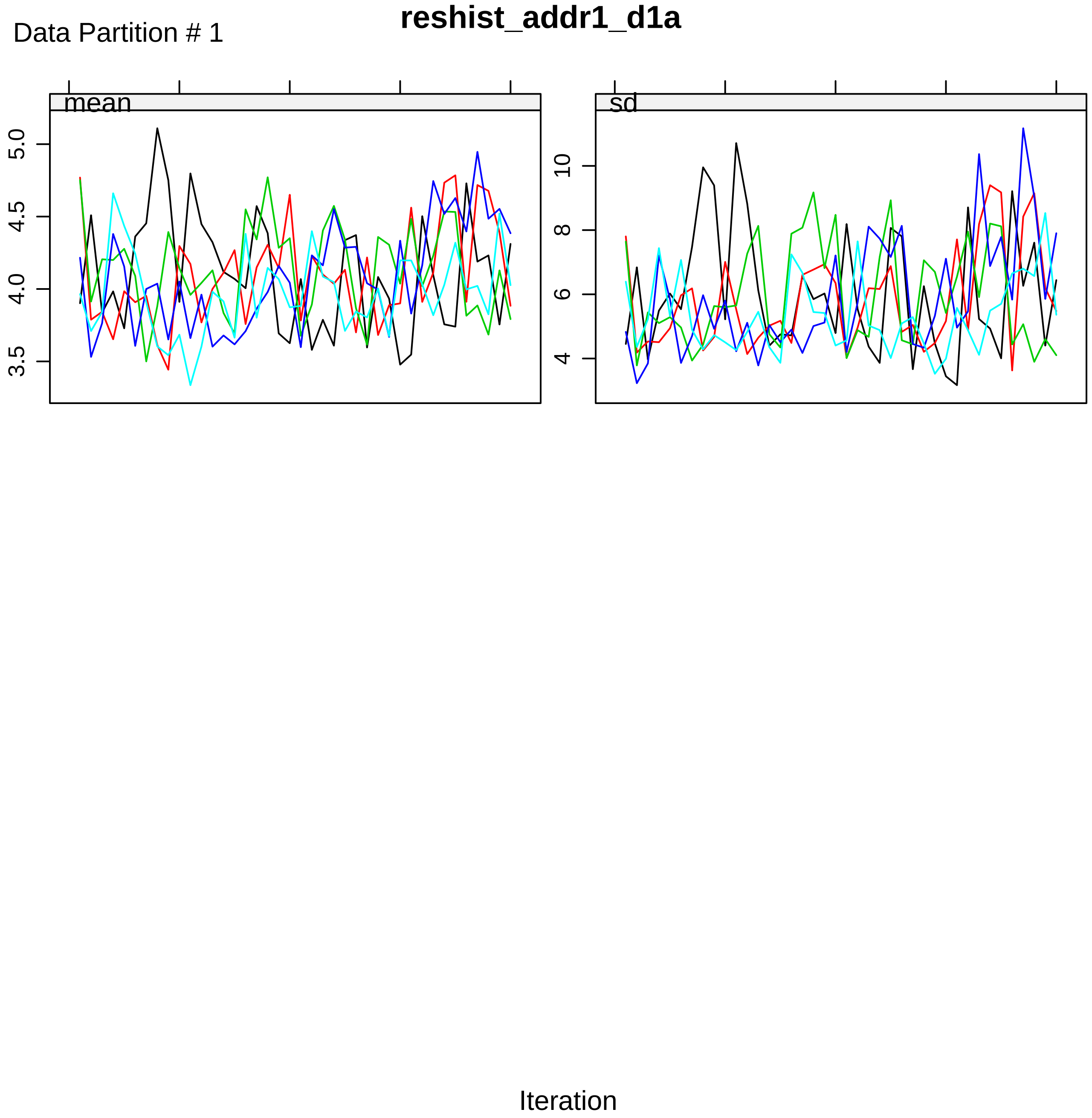

reshist_addr1_walkindex

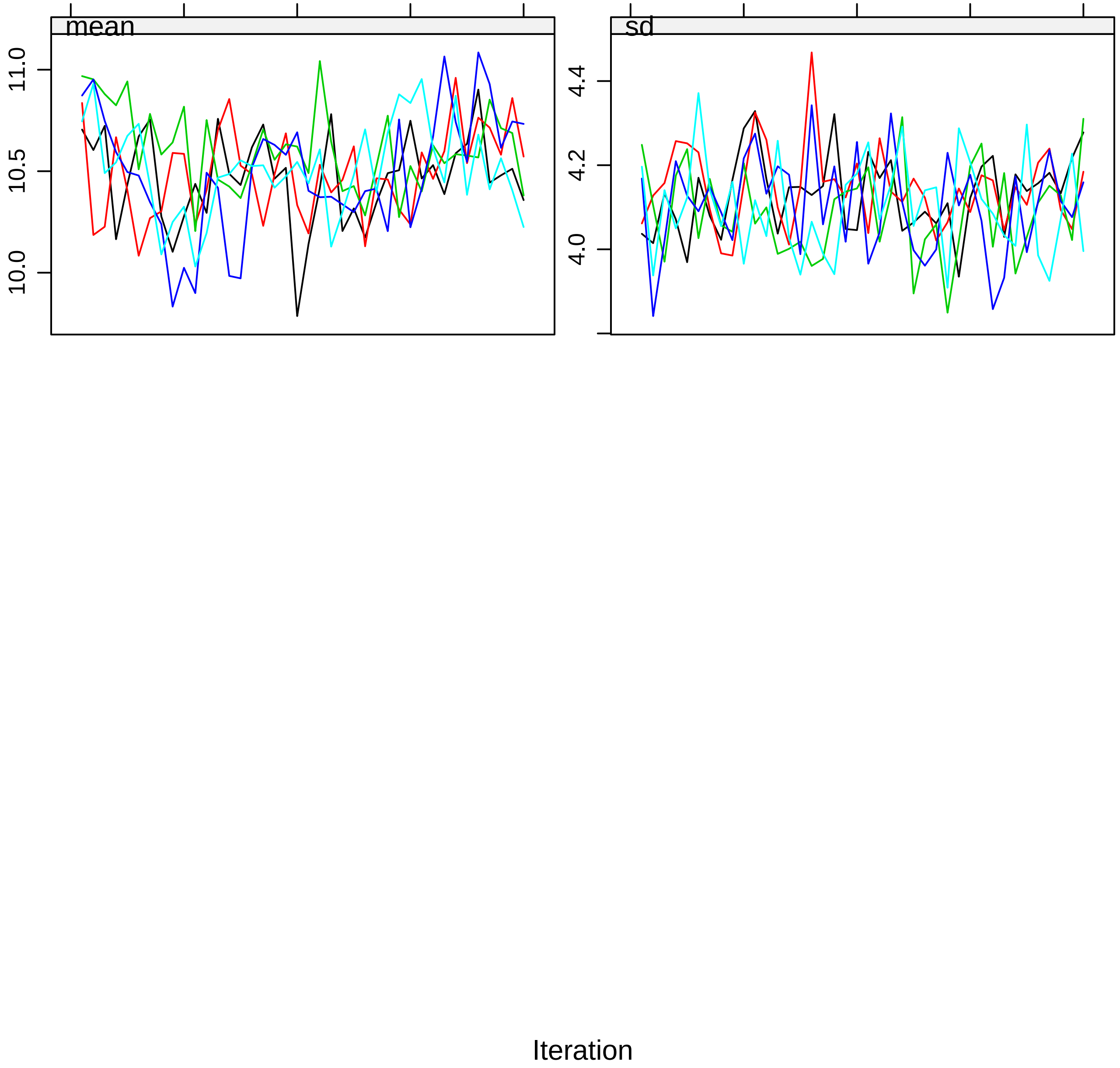

reshist_addr1_grndtot

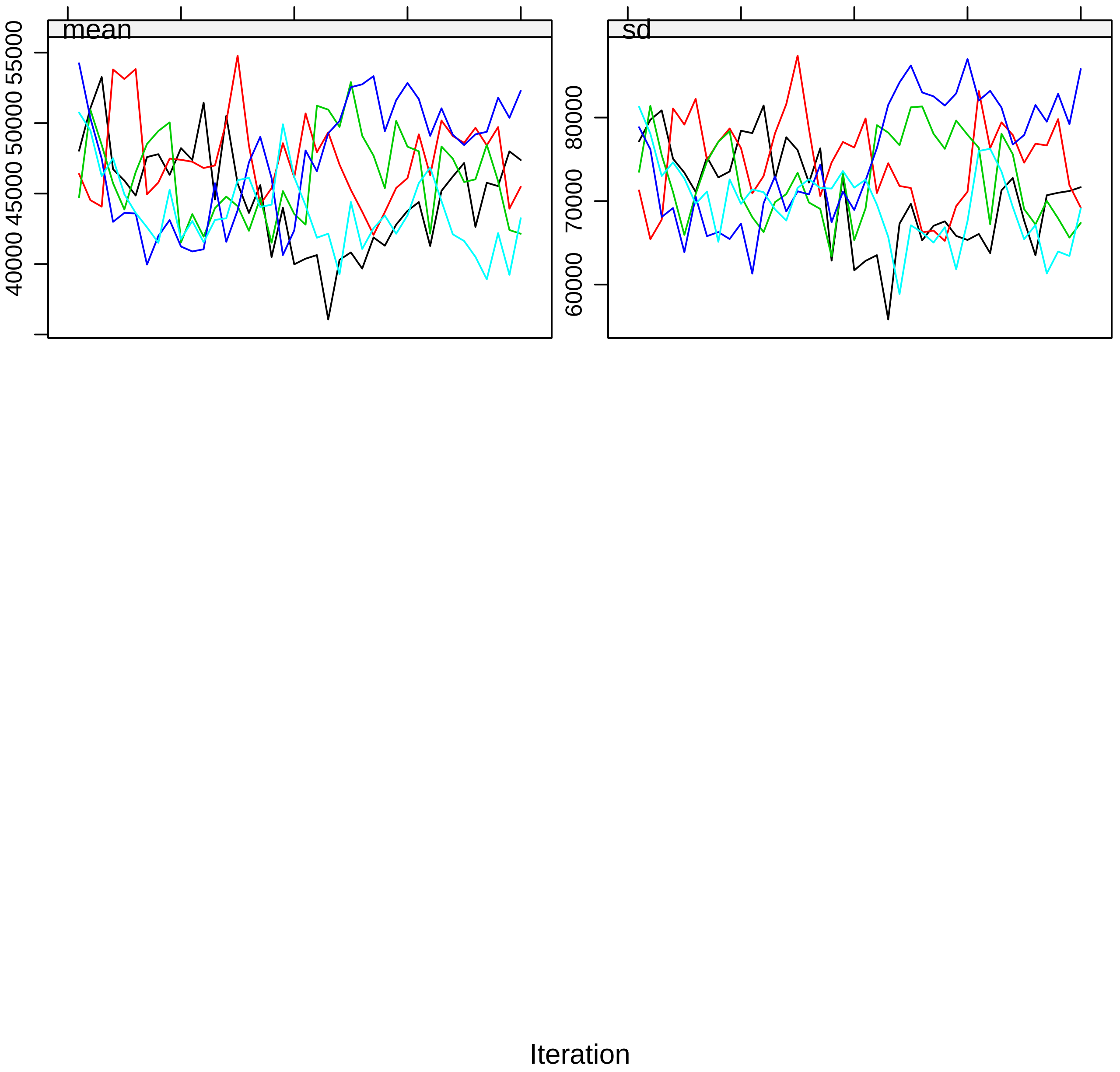

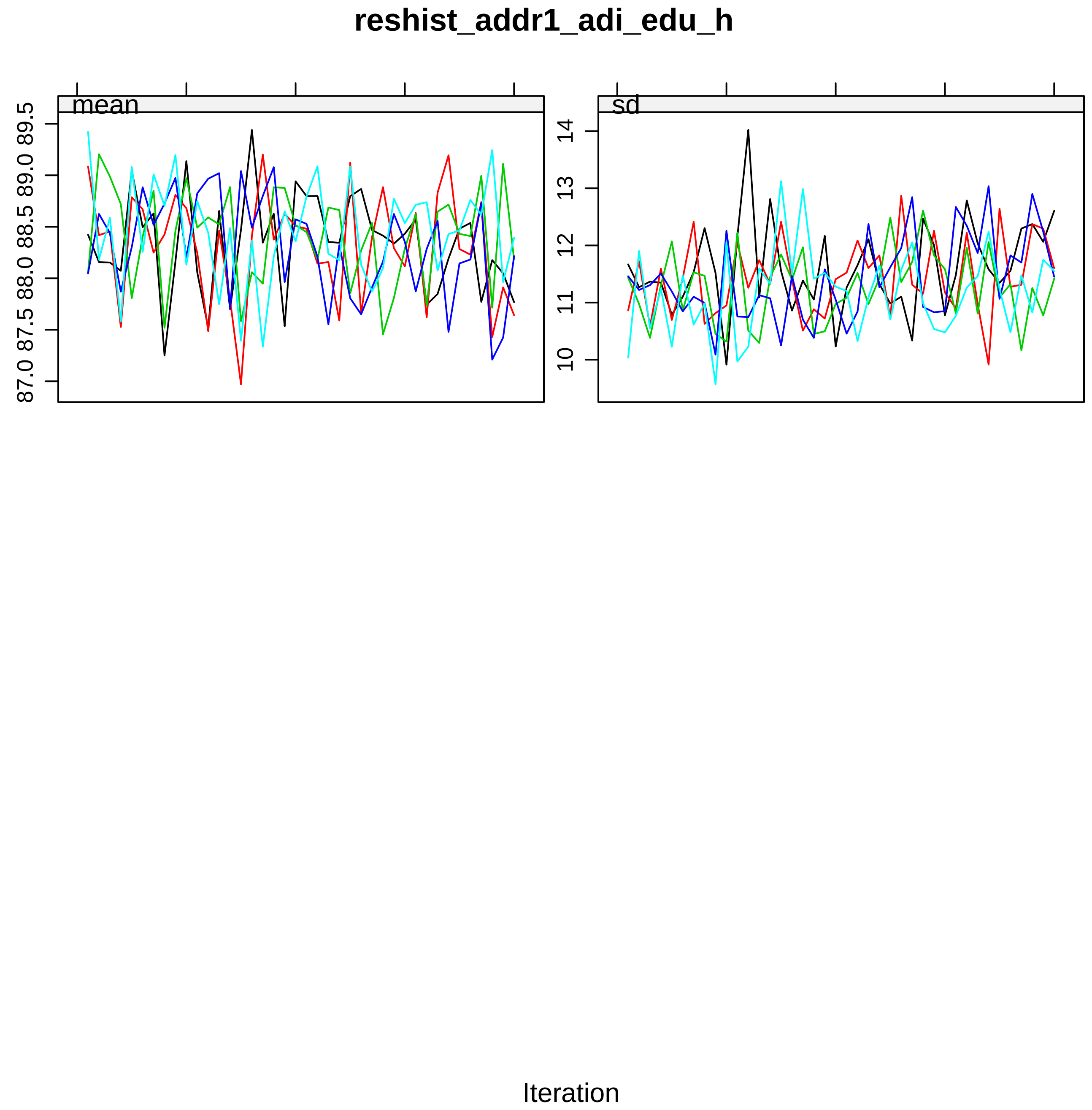
reshist_addr1_adi_edu_h
mean
sd
87.0
87.5
88.0
88.5
89.0
89.5
10
11
12
13
14
Iteration

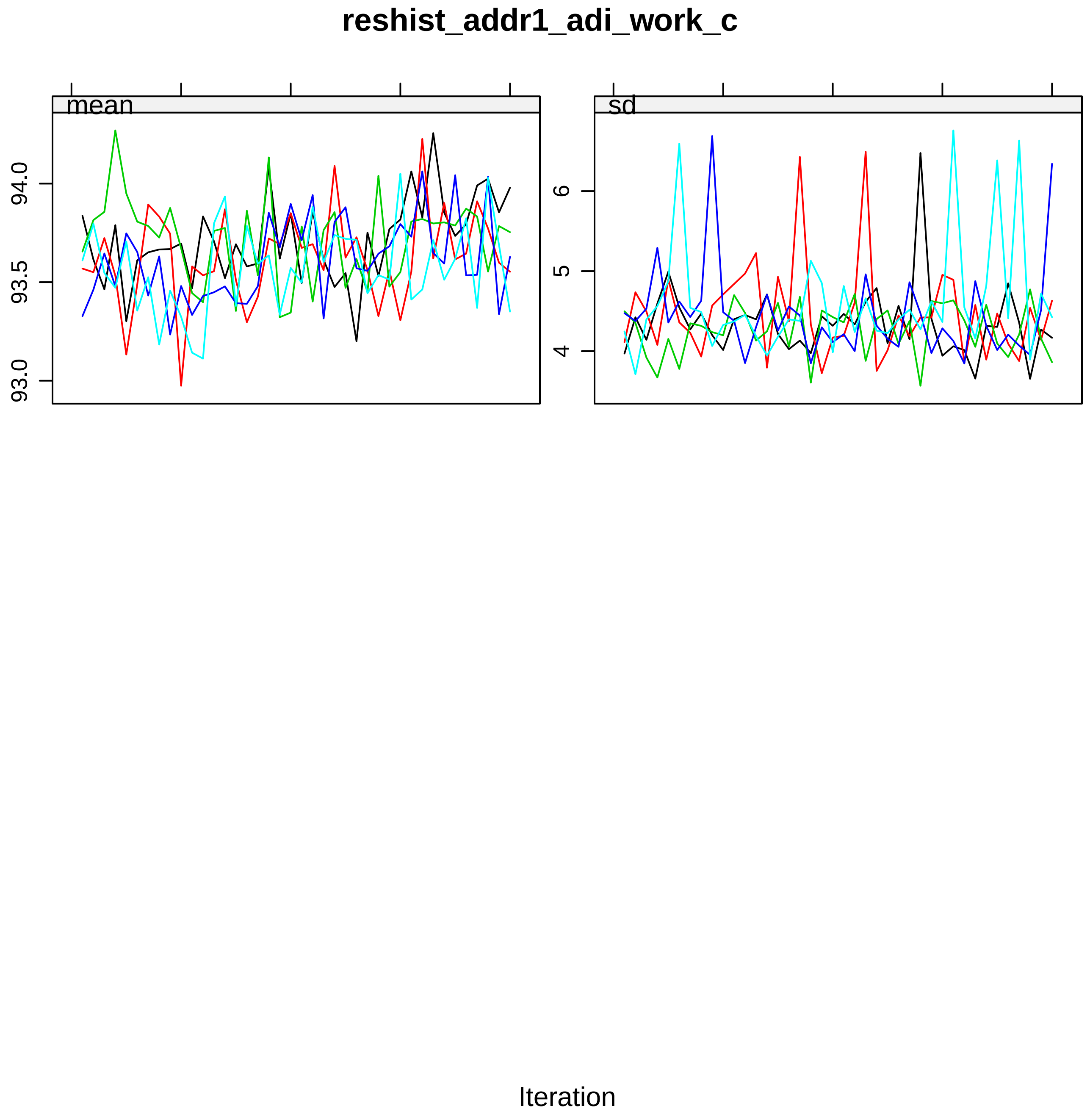
reshist_addr1_adi_work_c
mean
sd
93.0
93.5
94.0
4
5
6
Iteration

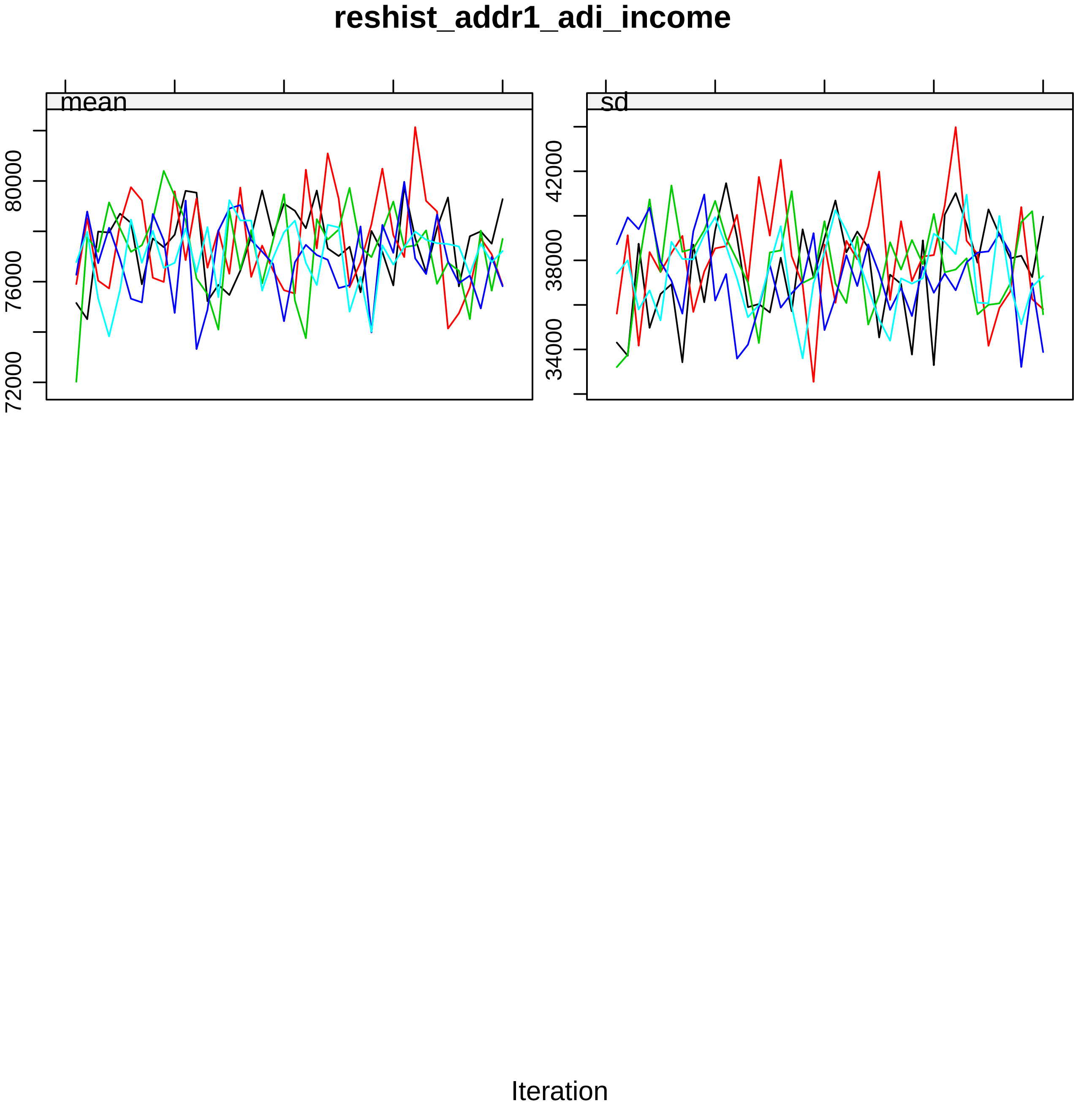

reshist_addr1_adi_income
mean
sd
72000
76000
80000
34000
38000
42000
Iteration

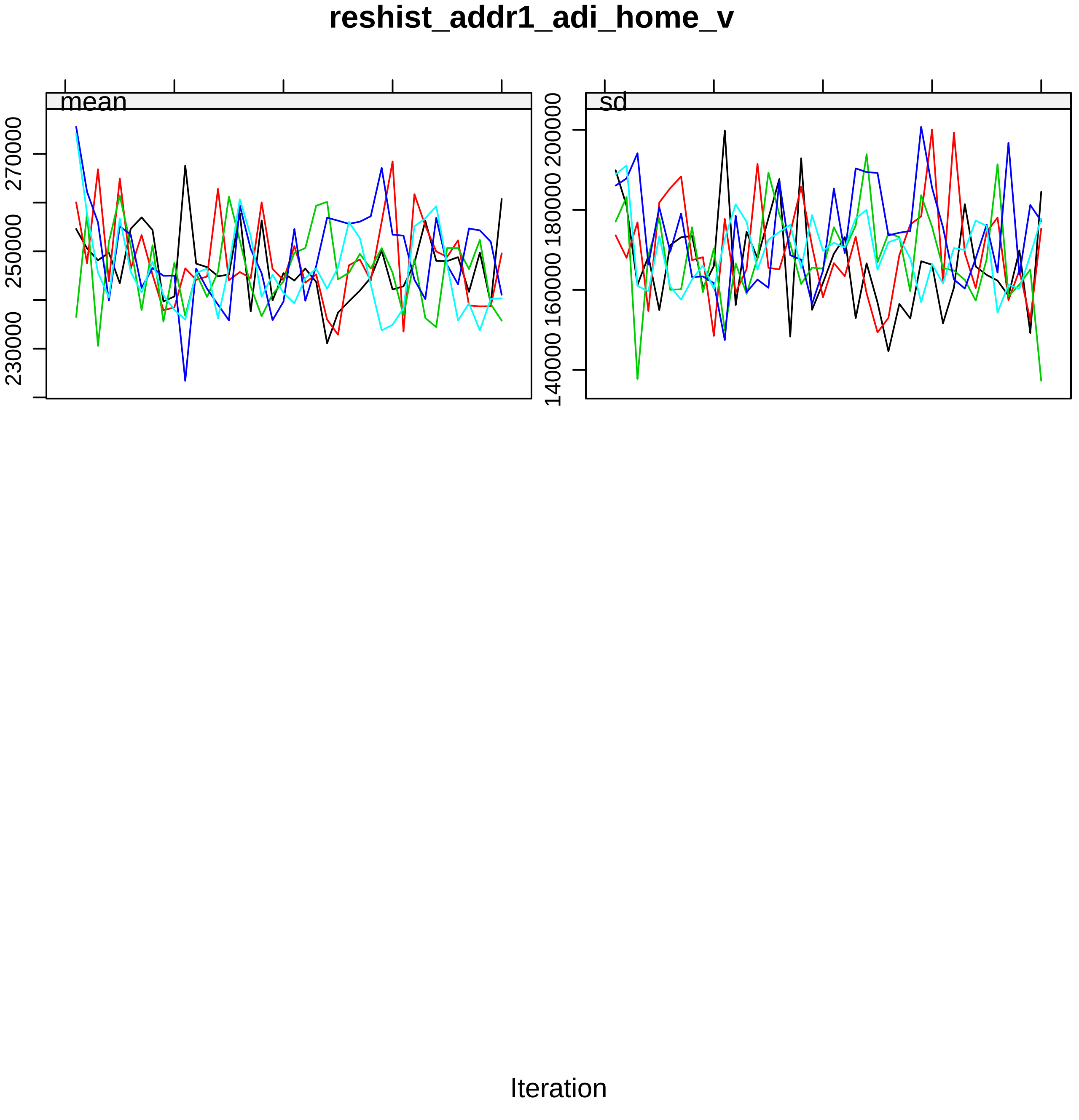
reshist_addr1_adi_home_v
mean
sd
230000
250000
270000
140000
160000
180000
200000
Iteration

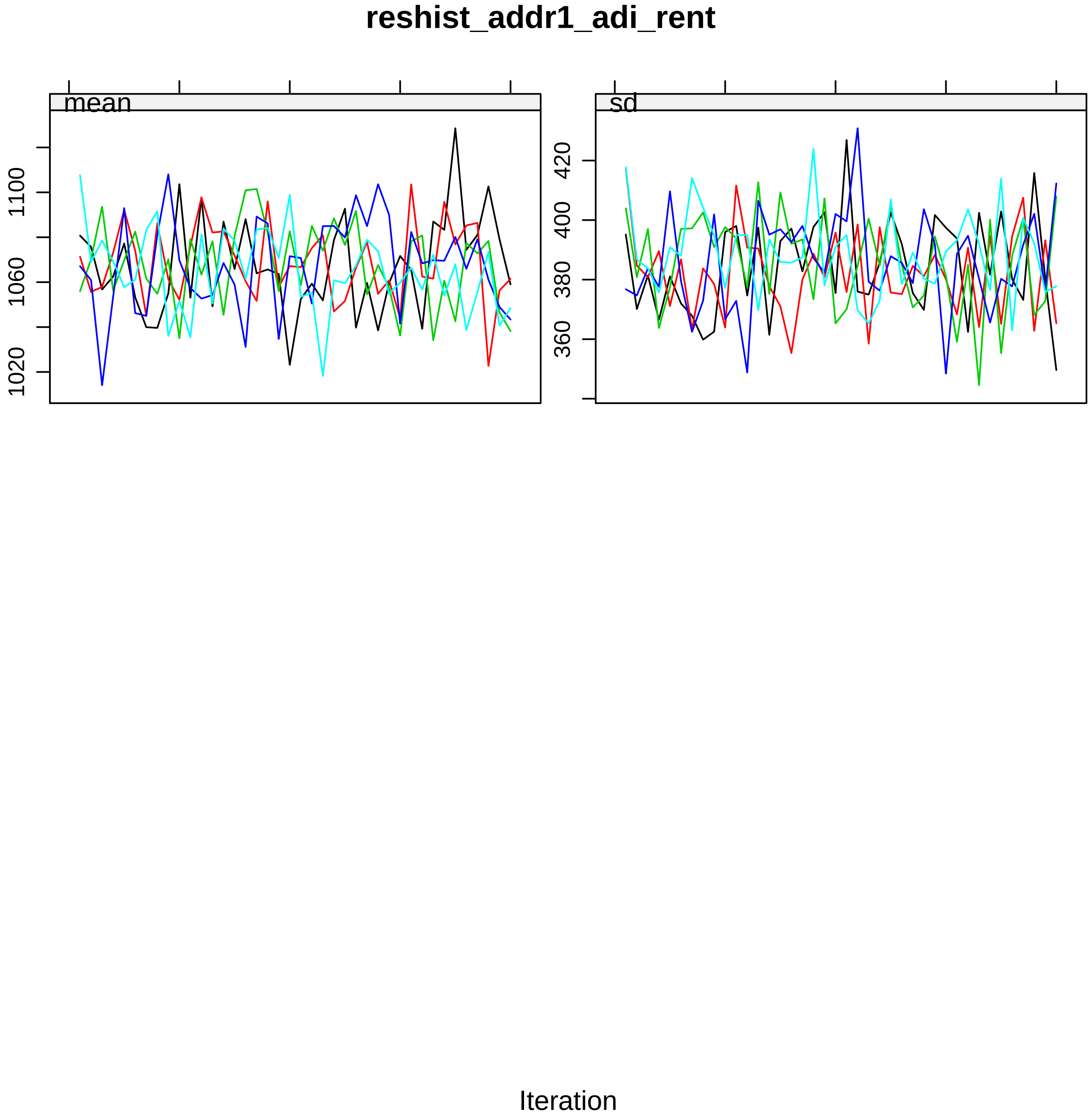

reshist_addr1_adi_rent
mean
sd
1020
1060
1100
360
380
400
420
Iteration

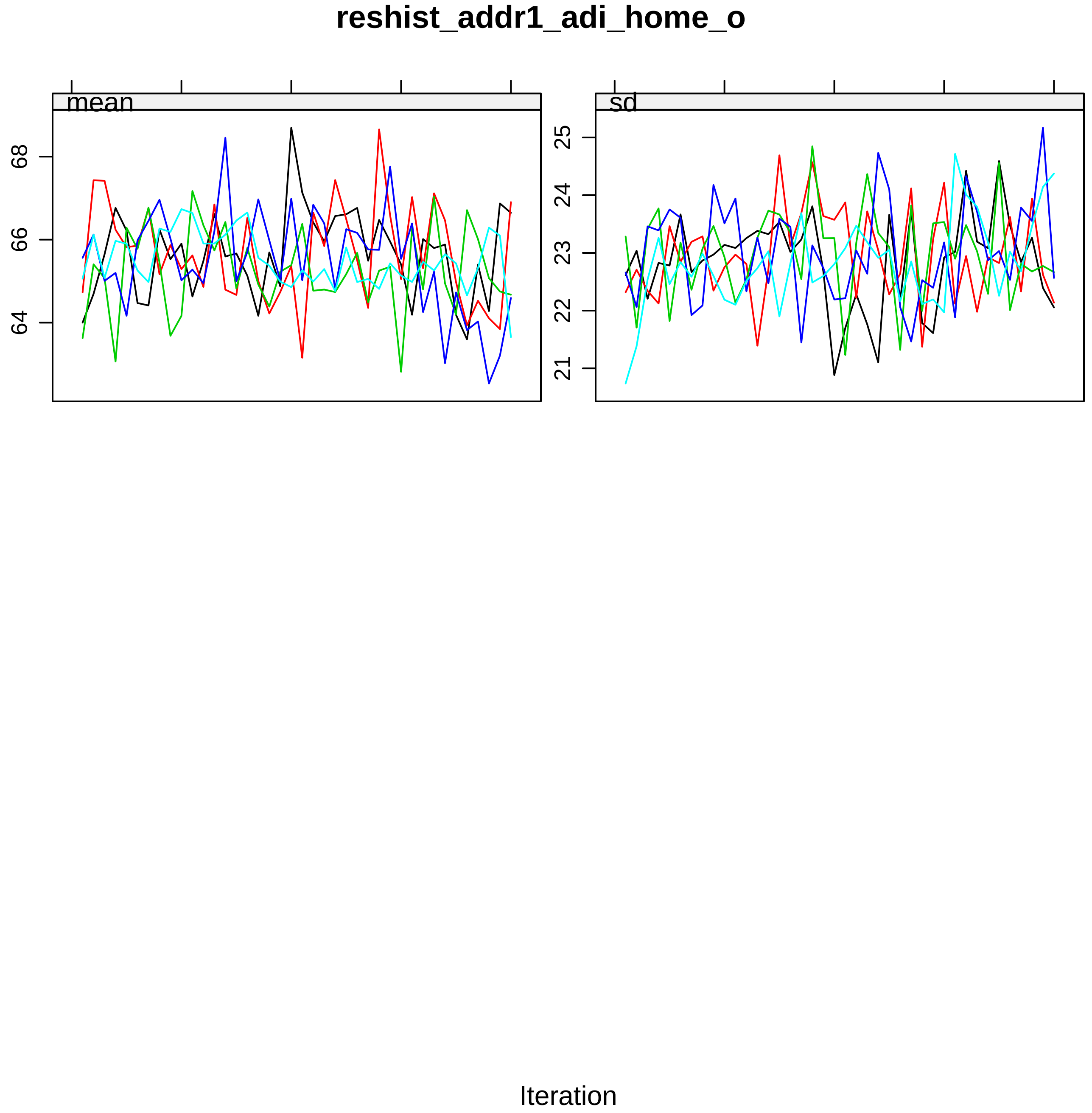

reshist_addr1_adi_home_o
mean
sd
68
66
64
25
24
23
22
21
Iteration

reshist_addr1_adi_crowd

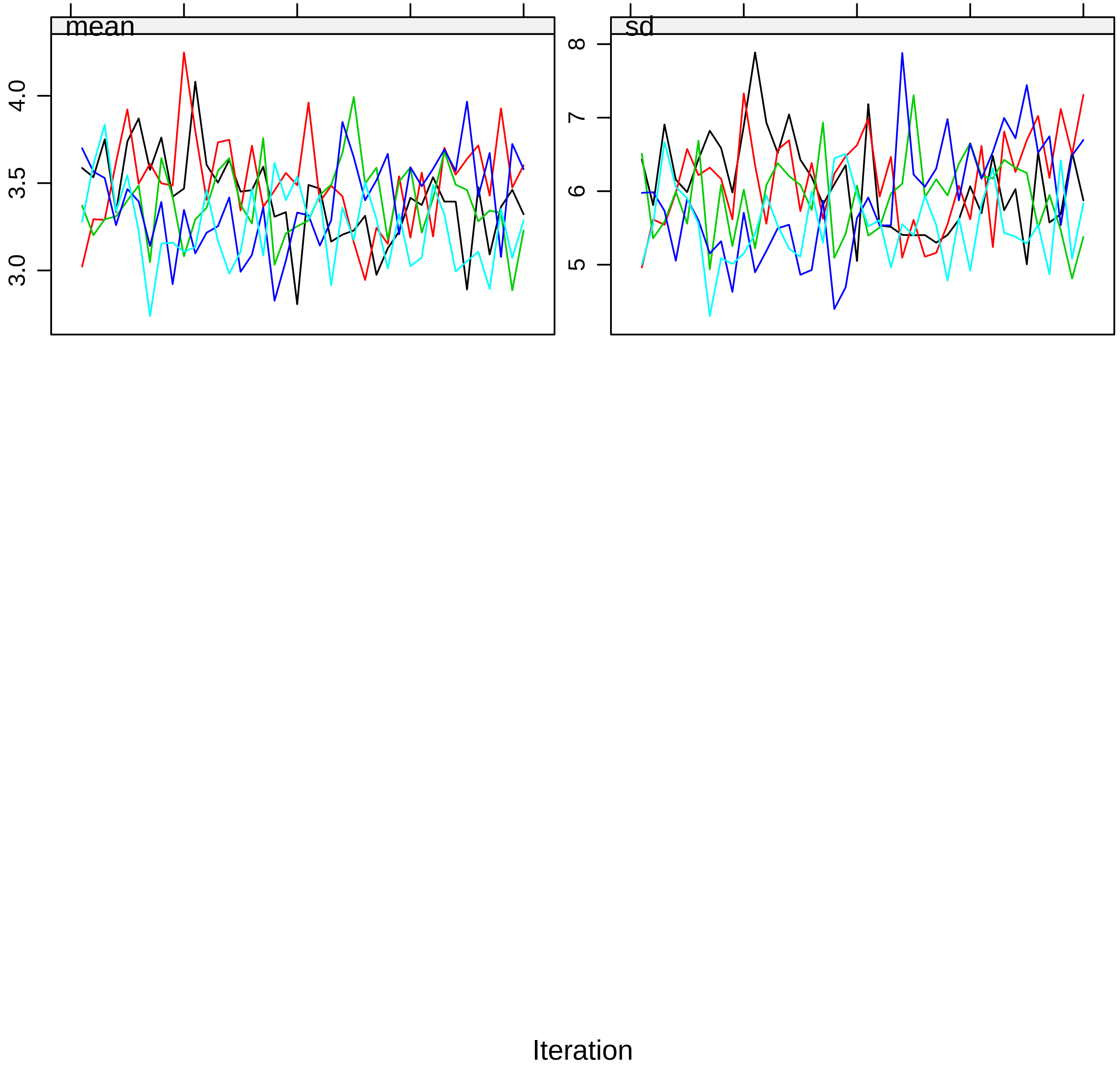

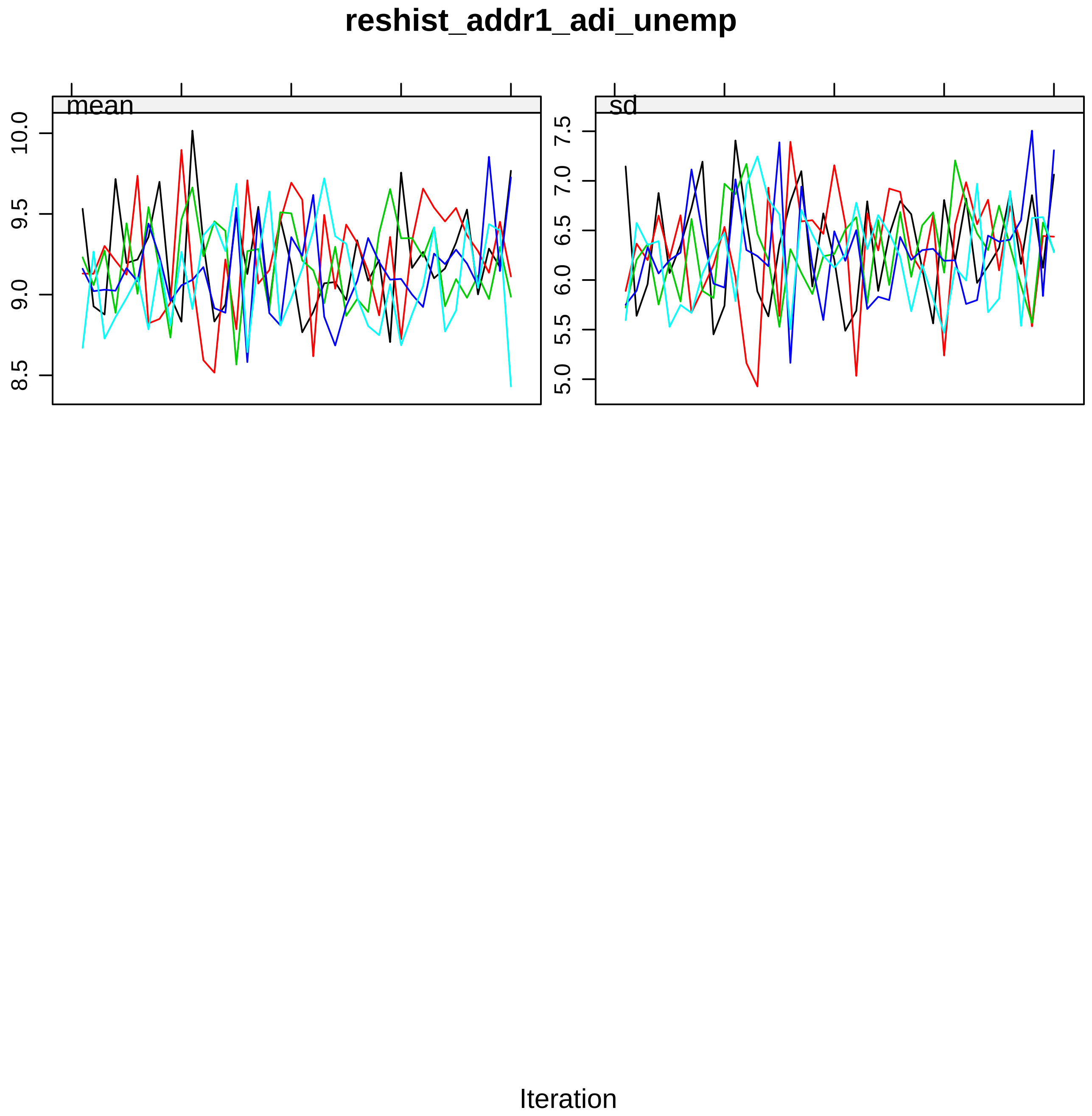
reshist_addr1_adi_unemp
mean
sd
10.0
9.5
9.0
8.5
7.5
7.0
6.5
6.0
5.5
5.0
Iteration

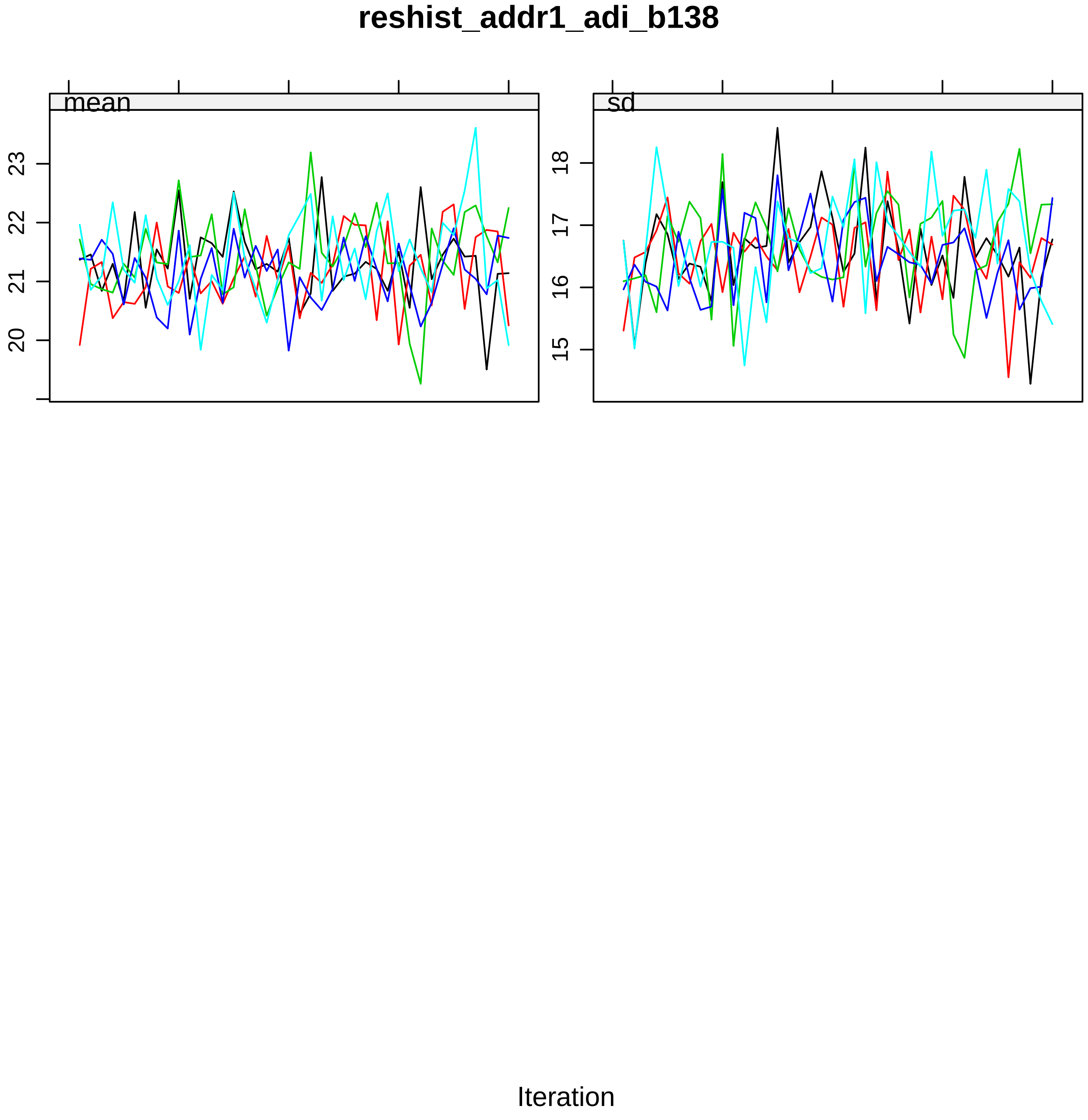

reshist_addr1_adi_b138
mean
sd
23
22
21
20
18
17
16
15
Iteration

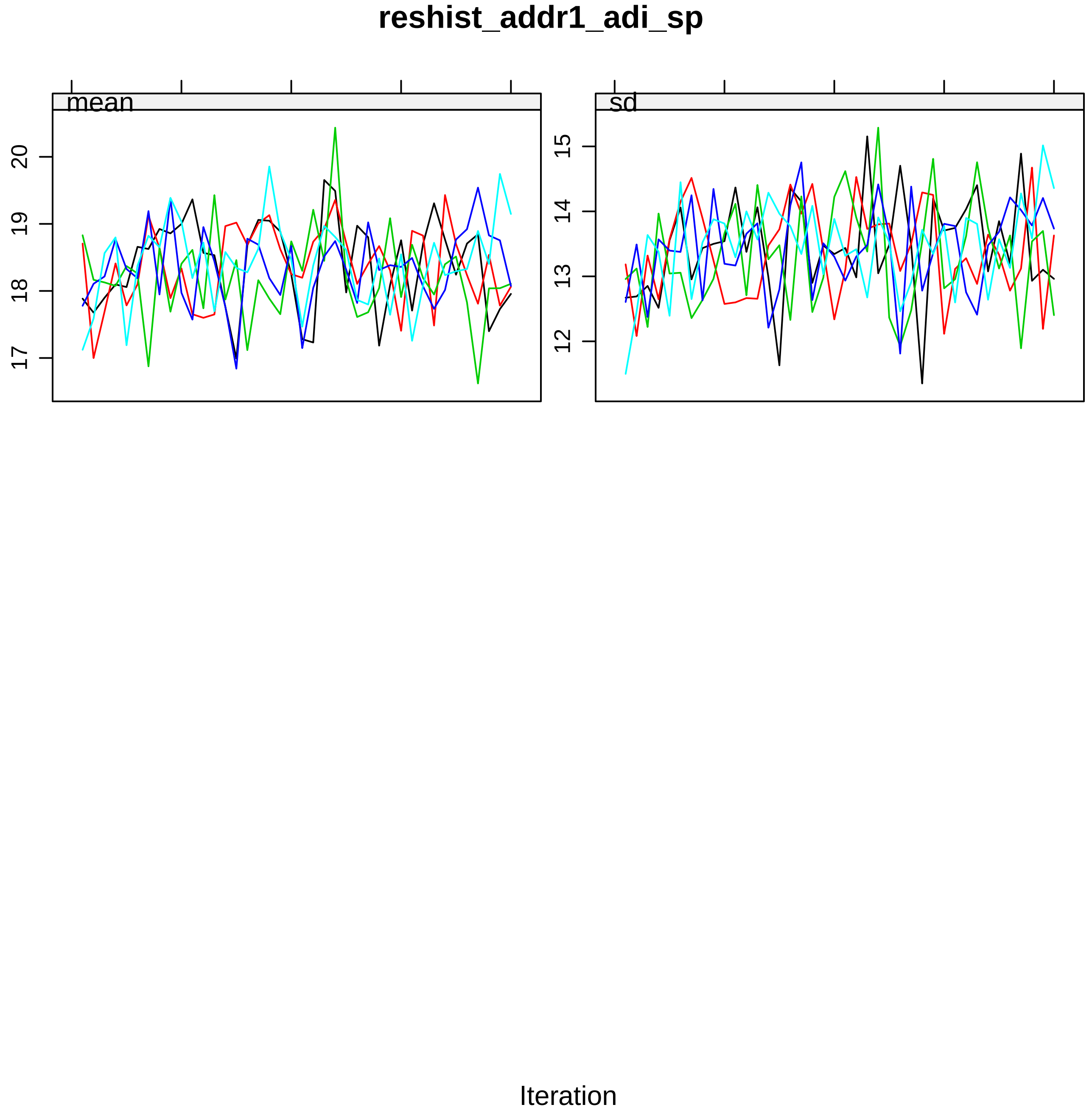

reshist_addr1_adi_sp
mean
sd
20
19
18
17
15
14
13
12
Iteration

reshist_addr1_adi_ncar

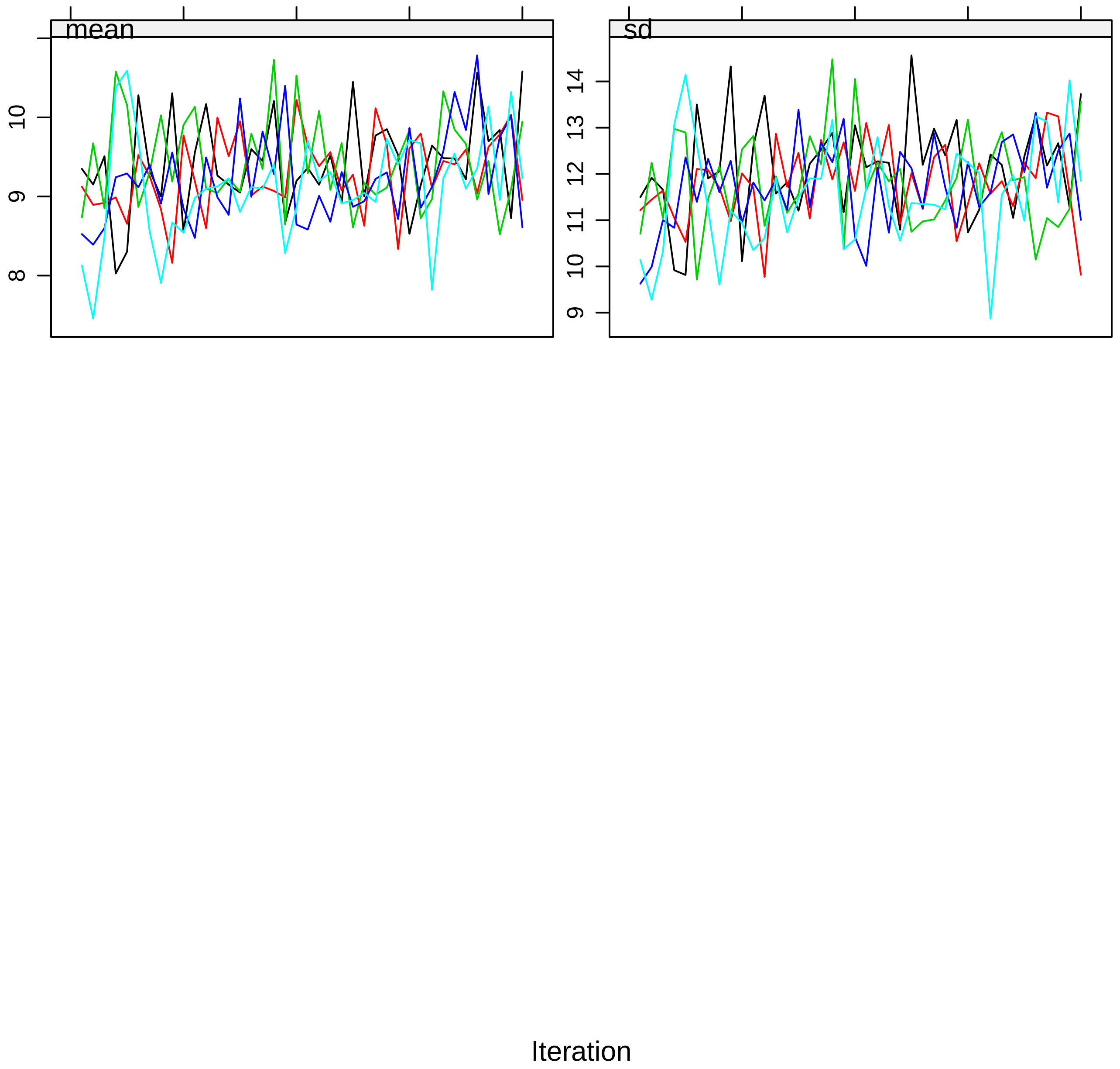

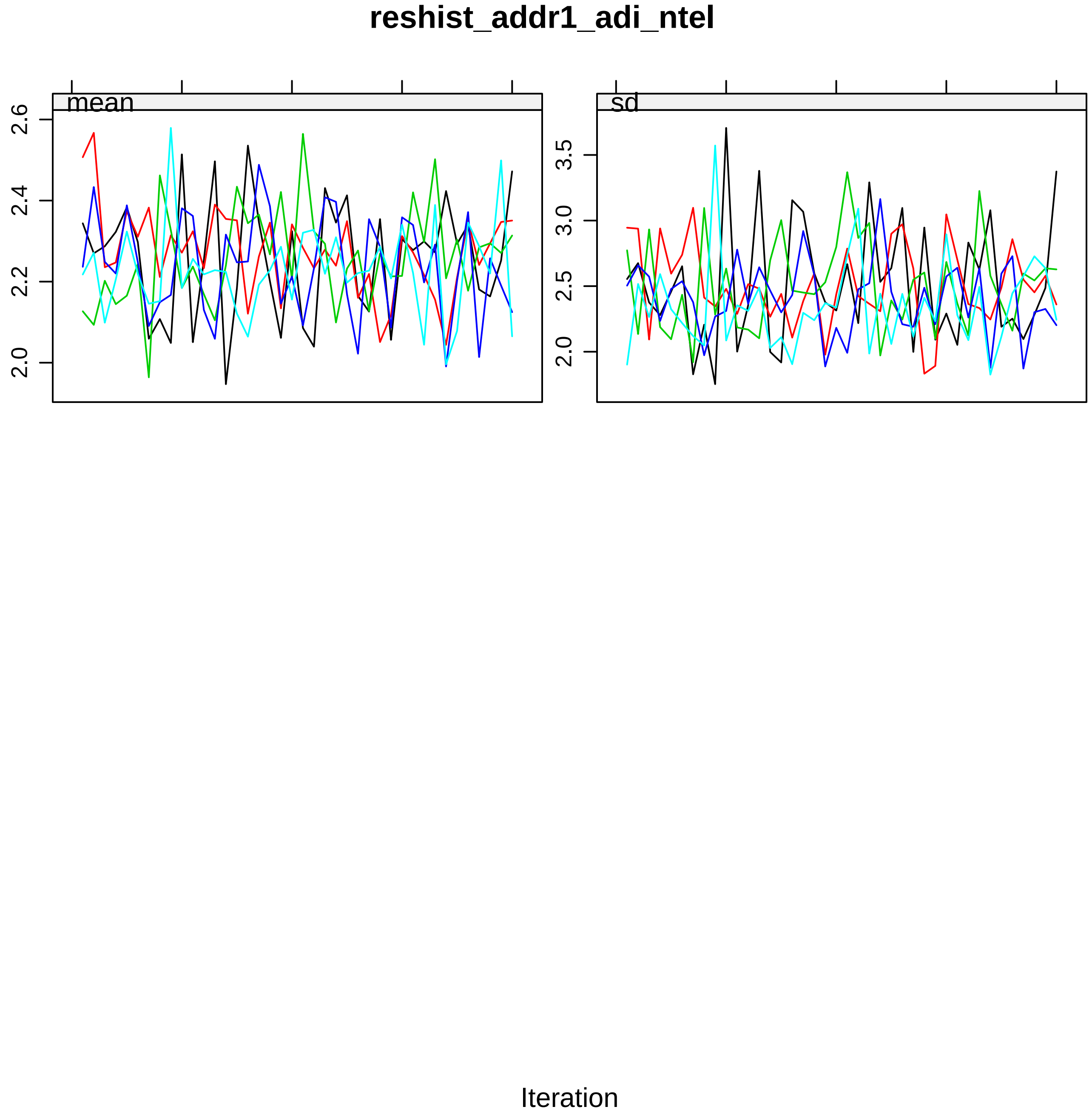
reshist_addr1_adi_ntel
mean
sd
2.0
2.2
2.4
2.6
2.0
2.5
3.0
3.5
Iteration

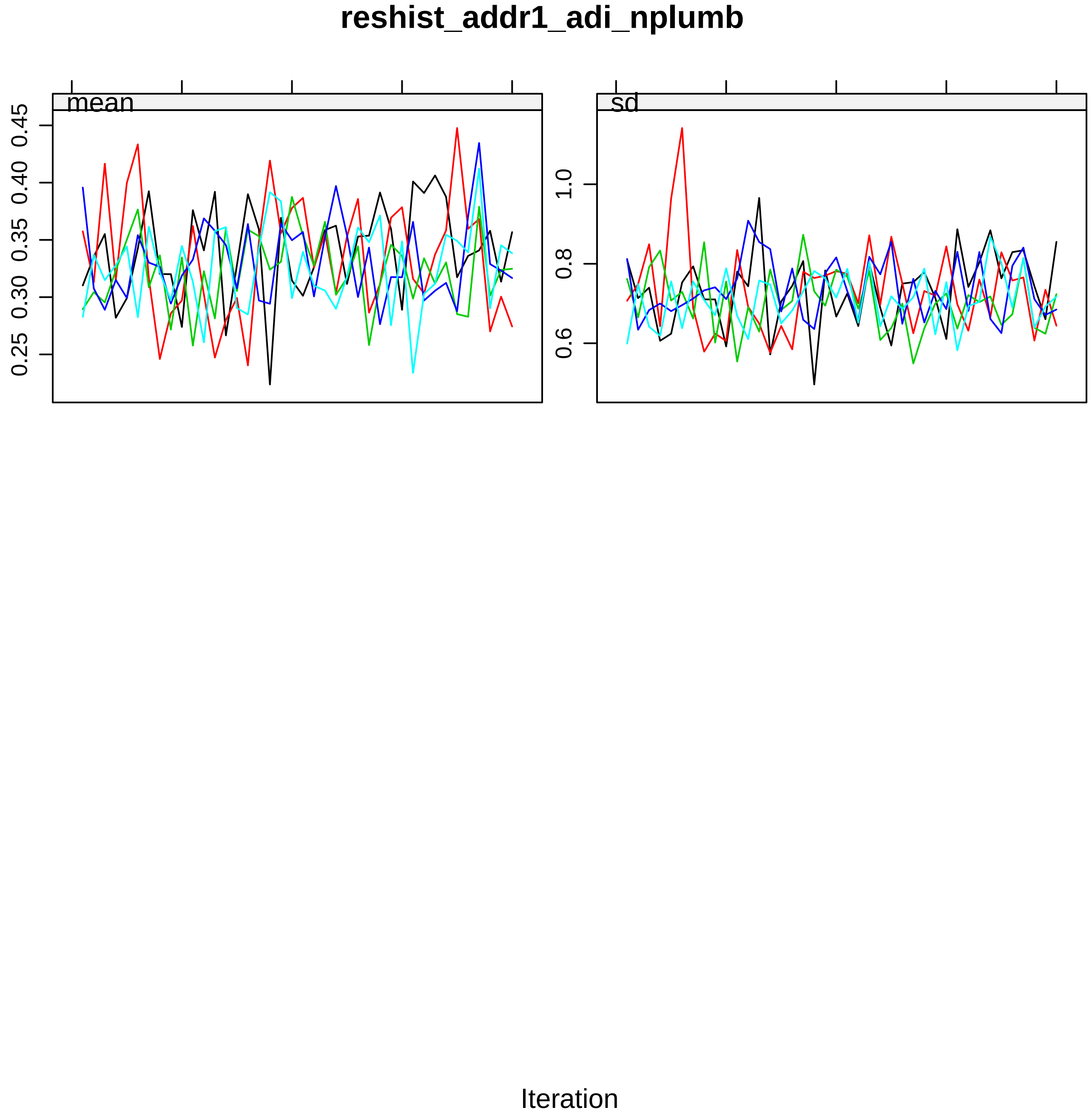

reshist_addr1_adi_nplumb
mean
sd
0.25
0.30
0.35
0.40
0.45
0.6
0.8
1.0
Iteration

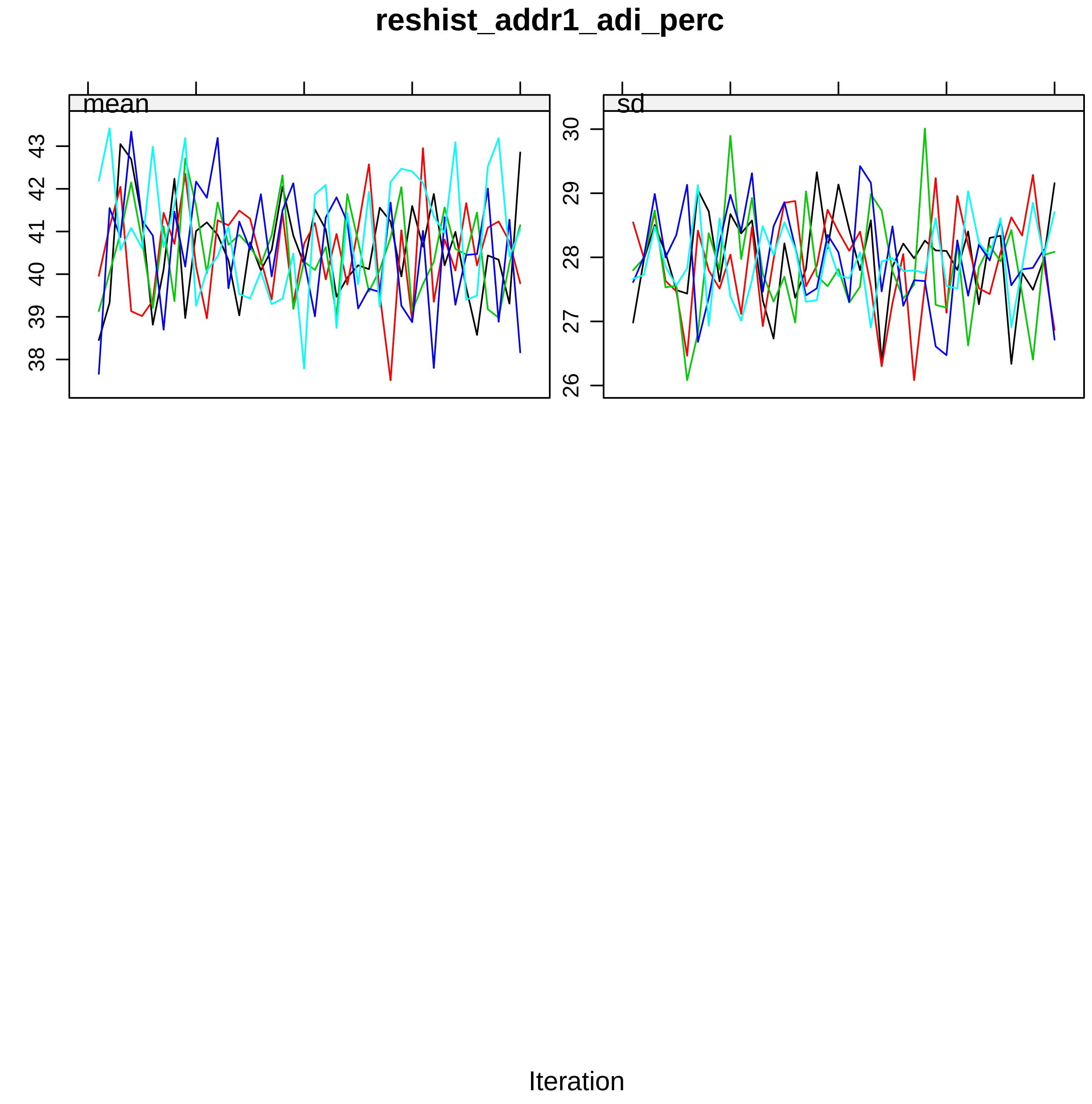
reshist_addr1_adi_perc
mean
sd
38
39
40
41
42
43
26
27
28
29
30
Iteration

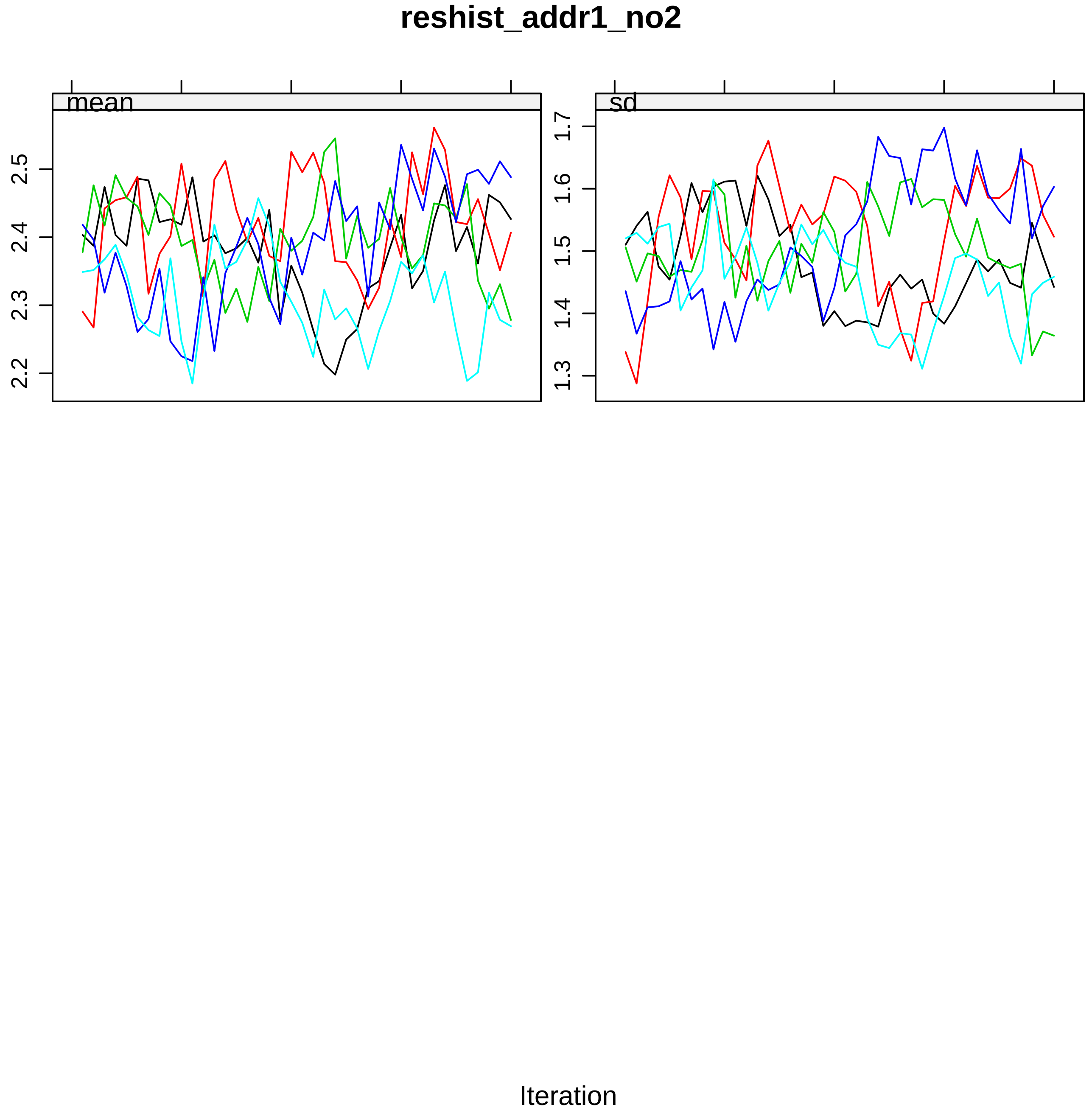
reshist_addr1_no2
mean
sd
2.2
2.3
2.4
2.5
1.3
1.4
1.5
1.6
1.7
Iteration

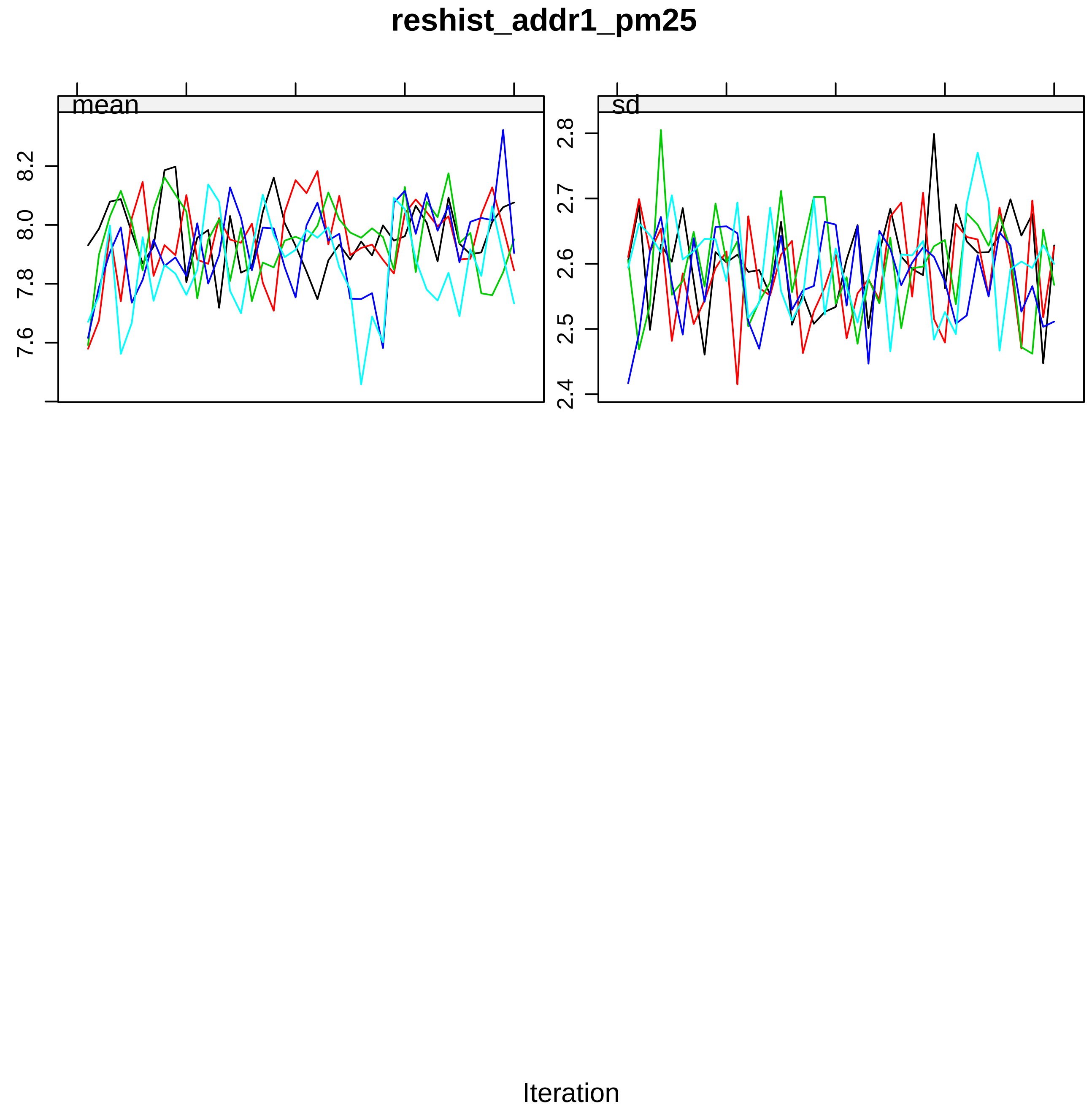

reshist_addr1_pm25
mean
sd
8.2
8.0
7.8
7.6
2.8
2.7
2.6
2.5
2.4
Iteration

reshist_addr1_proxrd

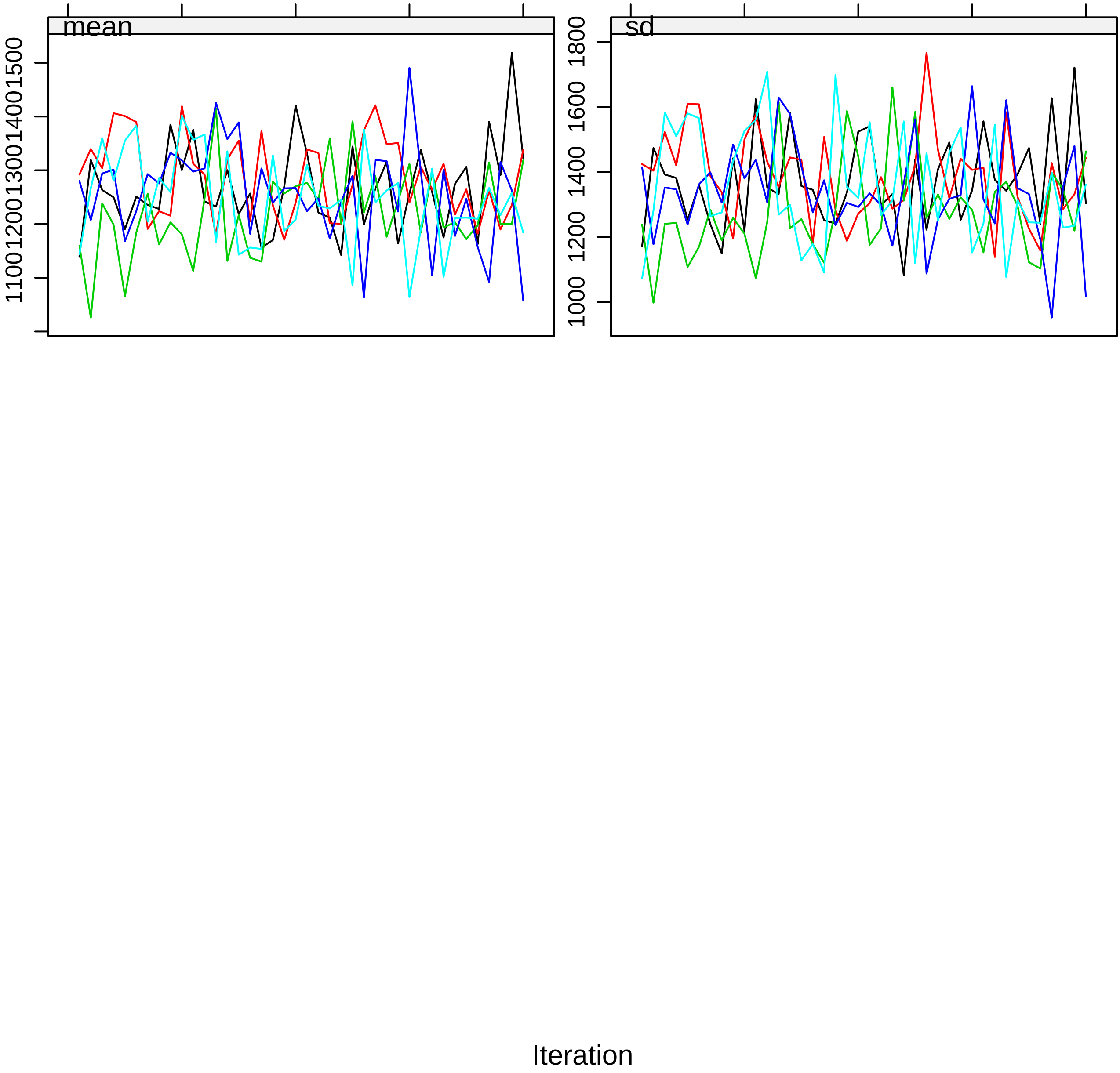

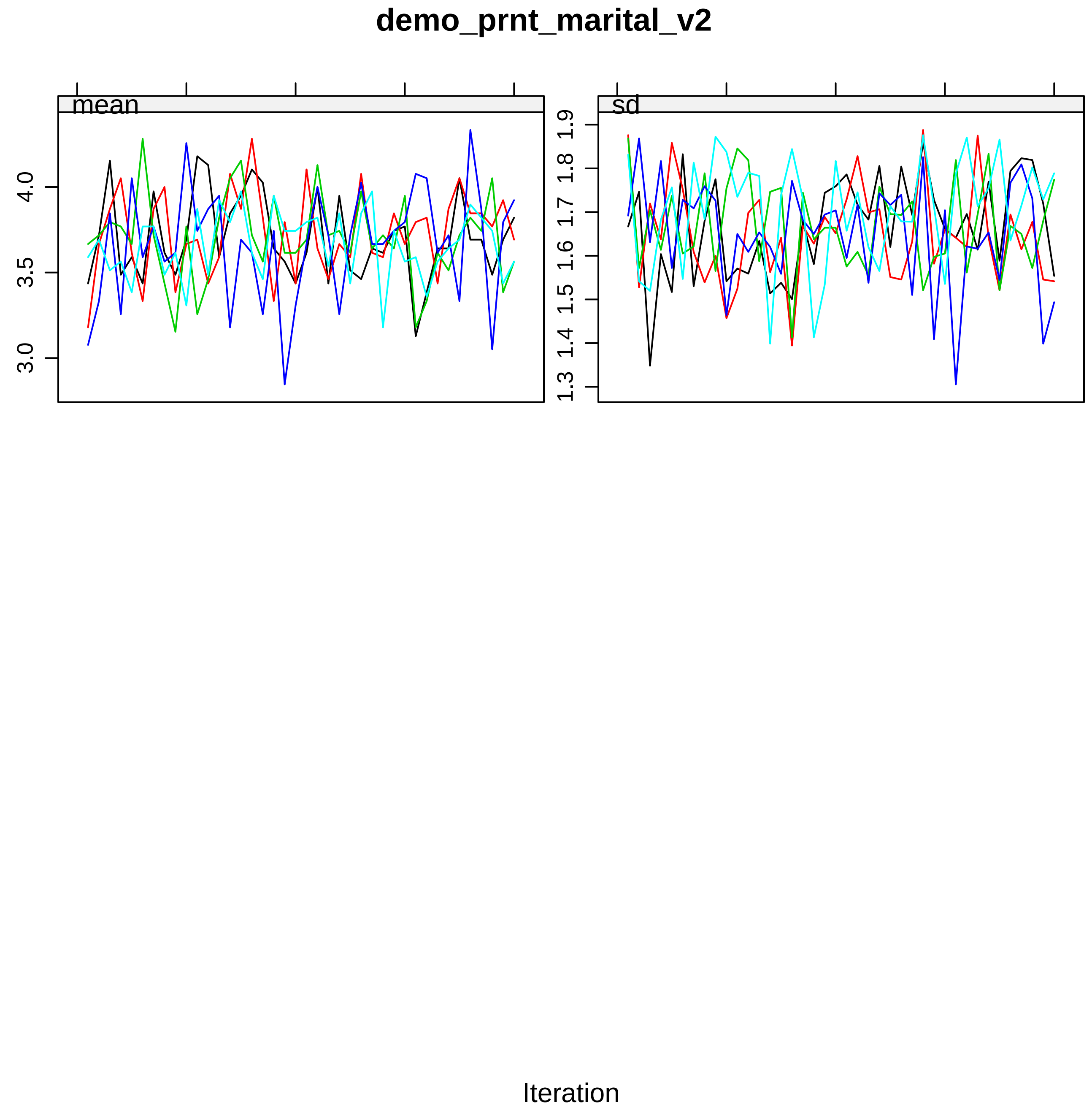
demo_prnt_marital_v2
mean
sd
3.0
3.5
4.0
1.3
1.4
1.5
1.6
1.7
1.8
1.9
Iteration

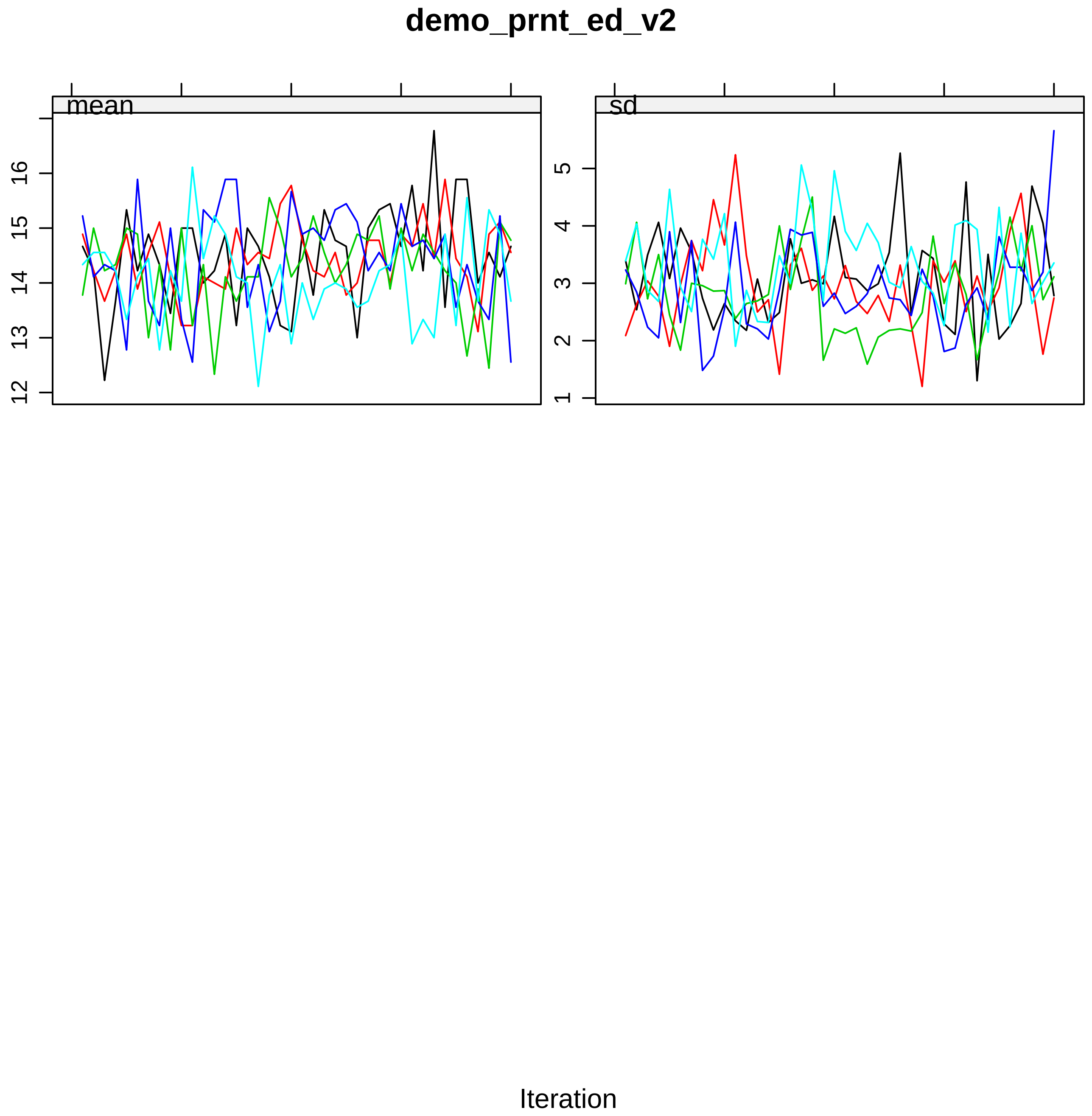
demo_prnt_ed_v2
mean
sd
12
13
14
15
16
1
2
3
4
5
Iteration

demo_prnt_empl_v2

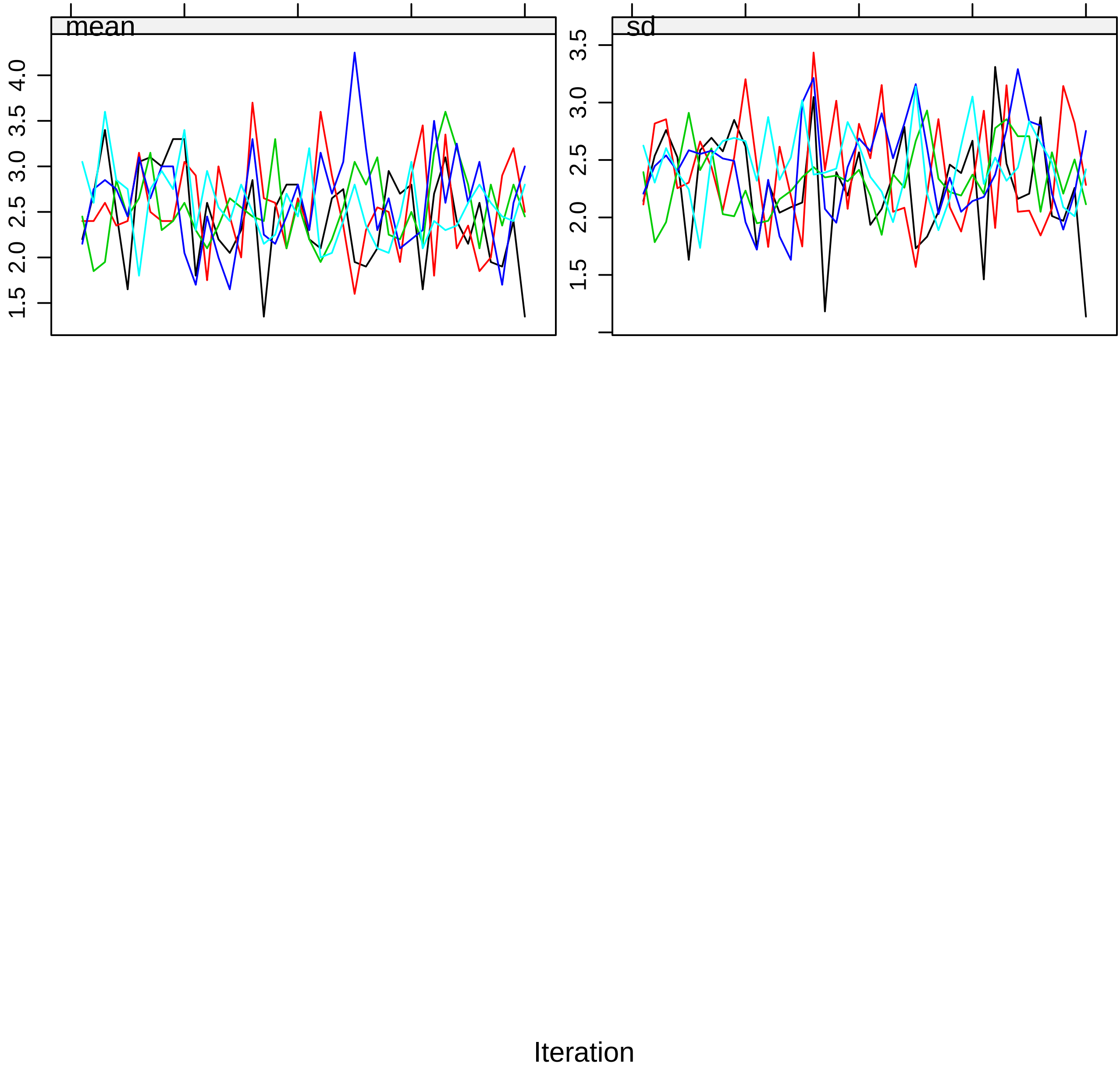

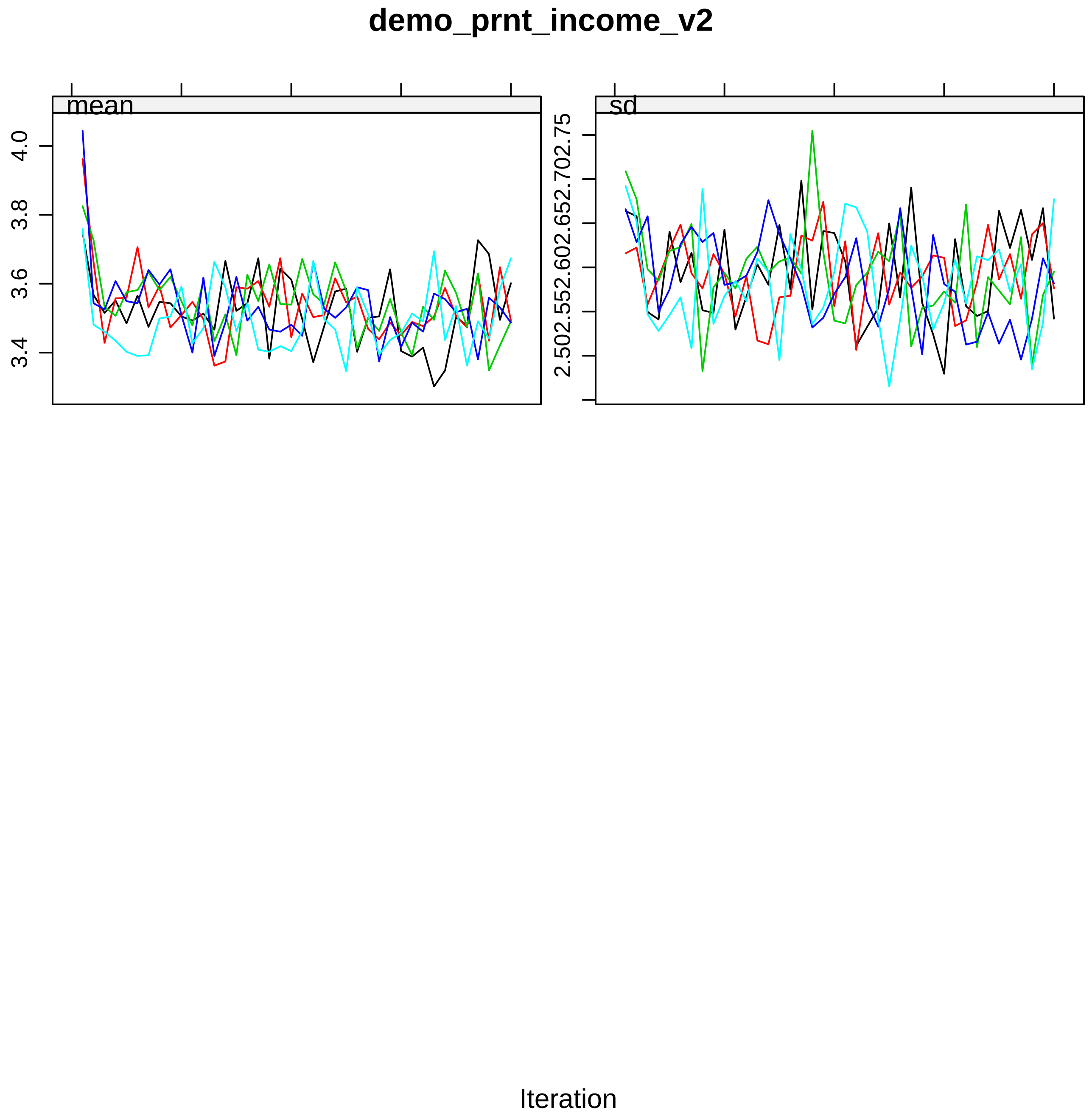

demo_prnt_income_v2
mean
sd
4.0
3.8
3.6
3.4
2.50
2.55
2.60
2.65
2.70
2.75
Iteration

demo_prnt_prtnr_v2

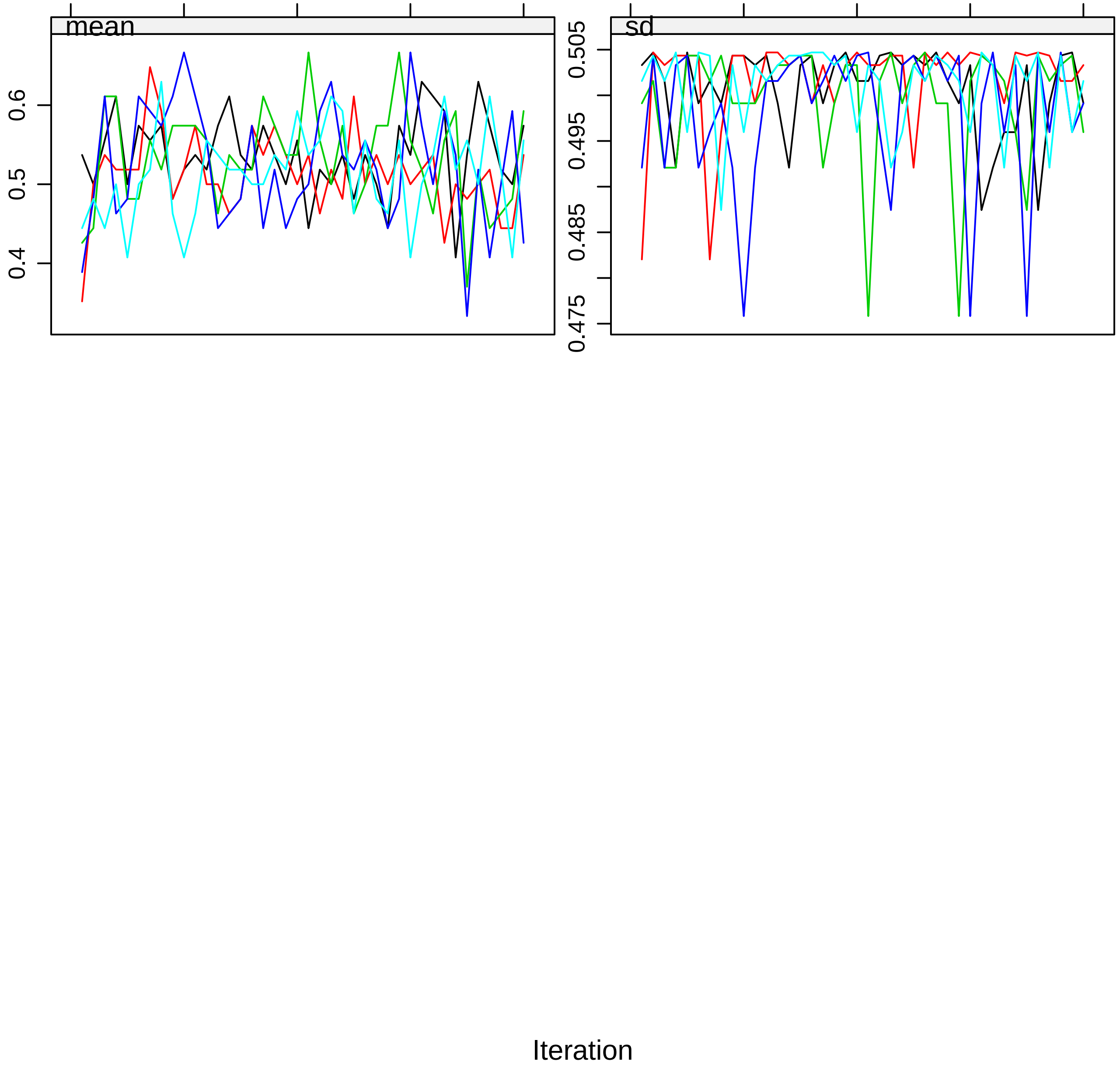

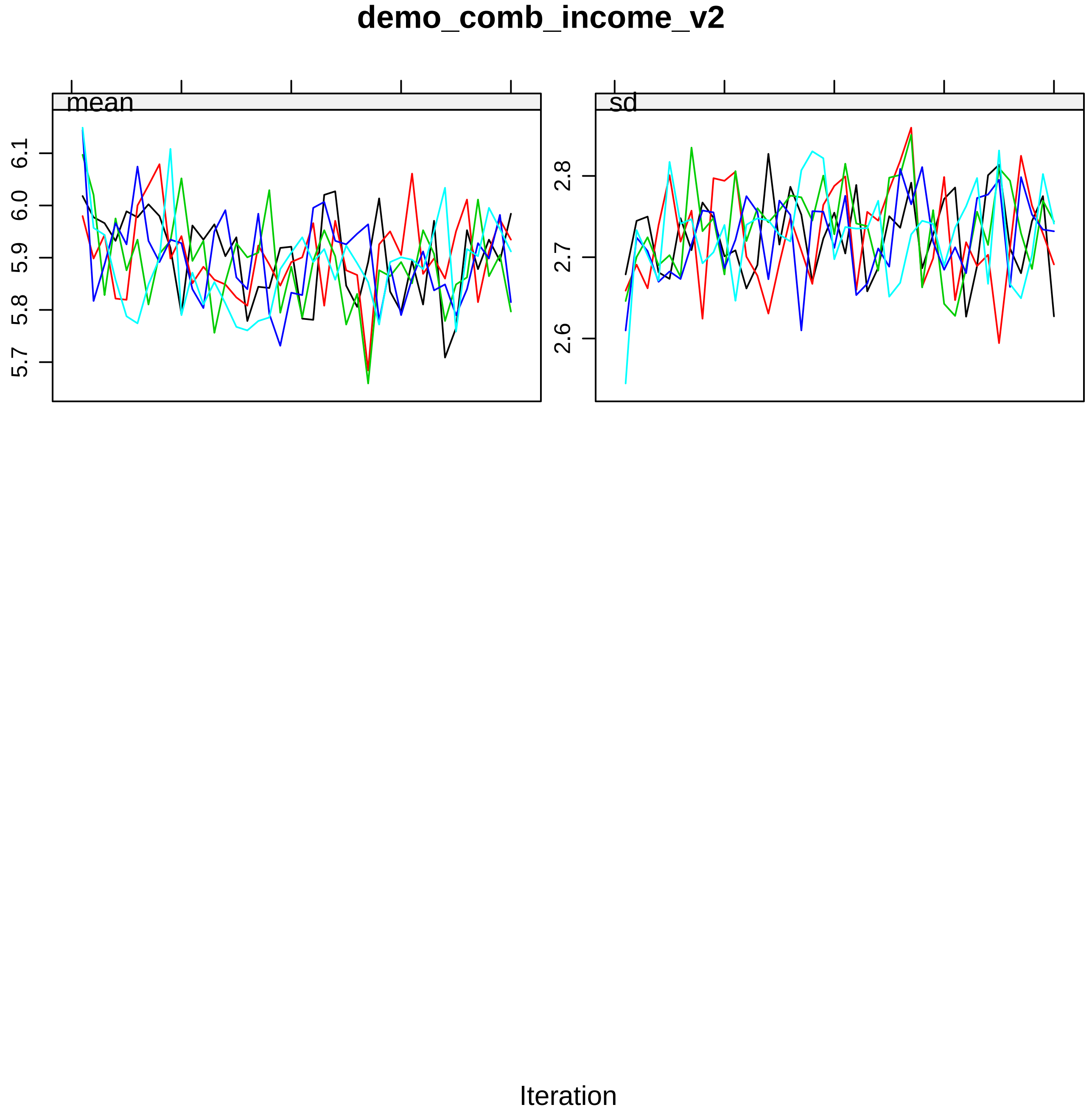
demo_comb_income_v2
mean
sd
6.1
6.0
5.9
5.8
5.7
2.8
2.7
2.6
Iteration

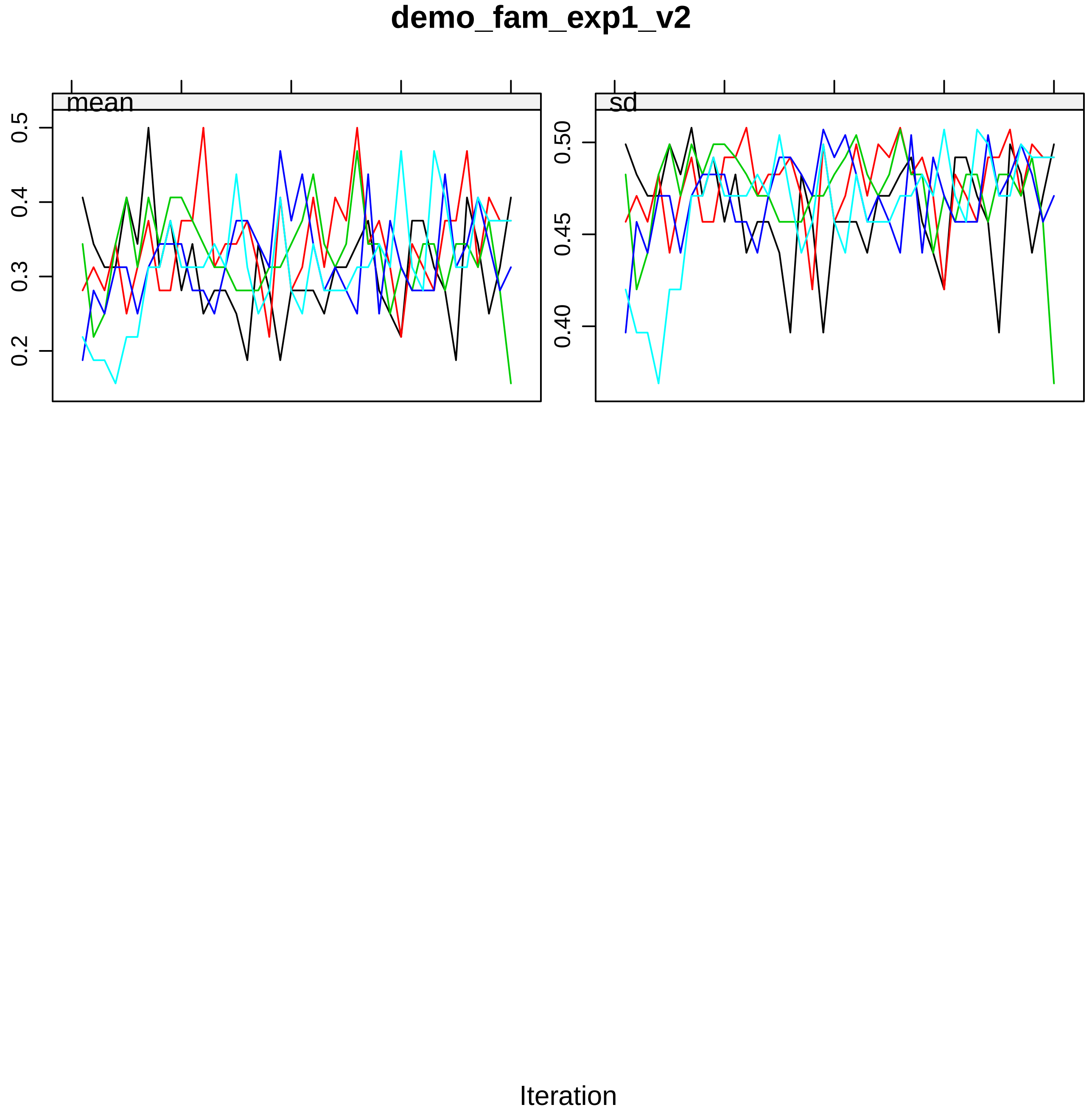
demo_fam_exp1_v2
mean
sd
0.2
0.3
0.4
0.5
0.40
0.45
0.50
Iteration

demo_fam_exp2_v2

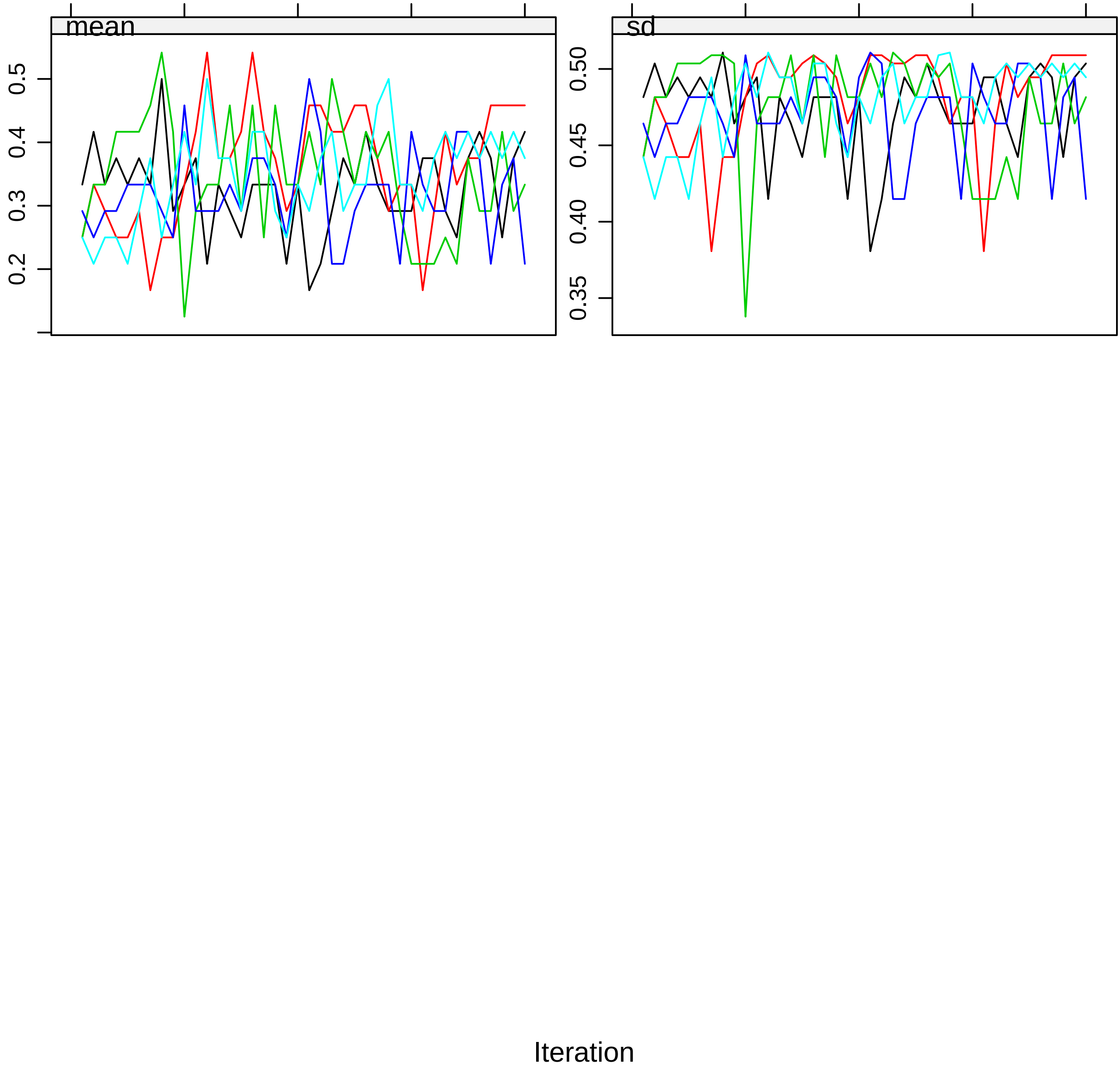

demo_fam_exp3_v2

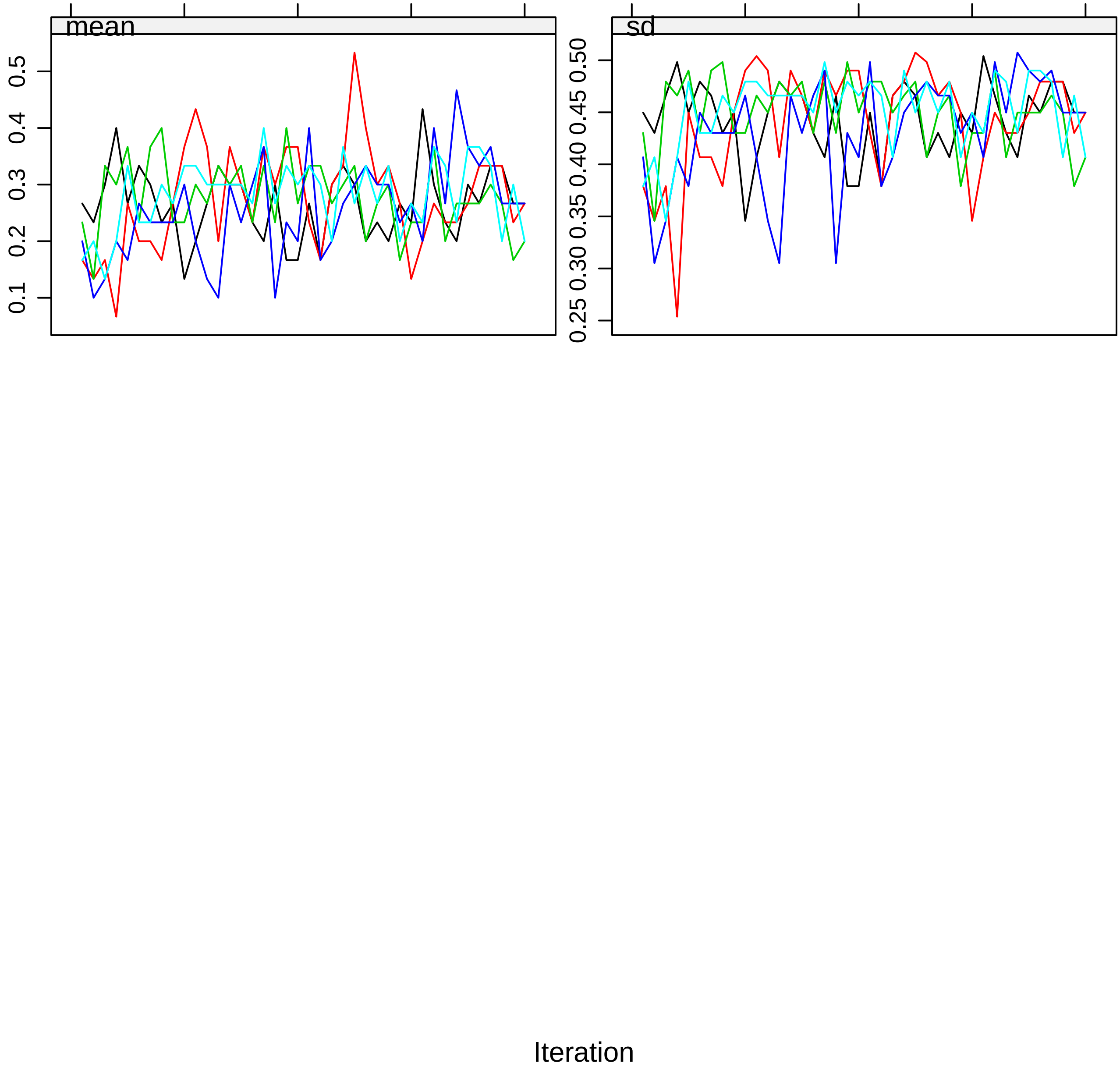

demo_fam_exp4_v2

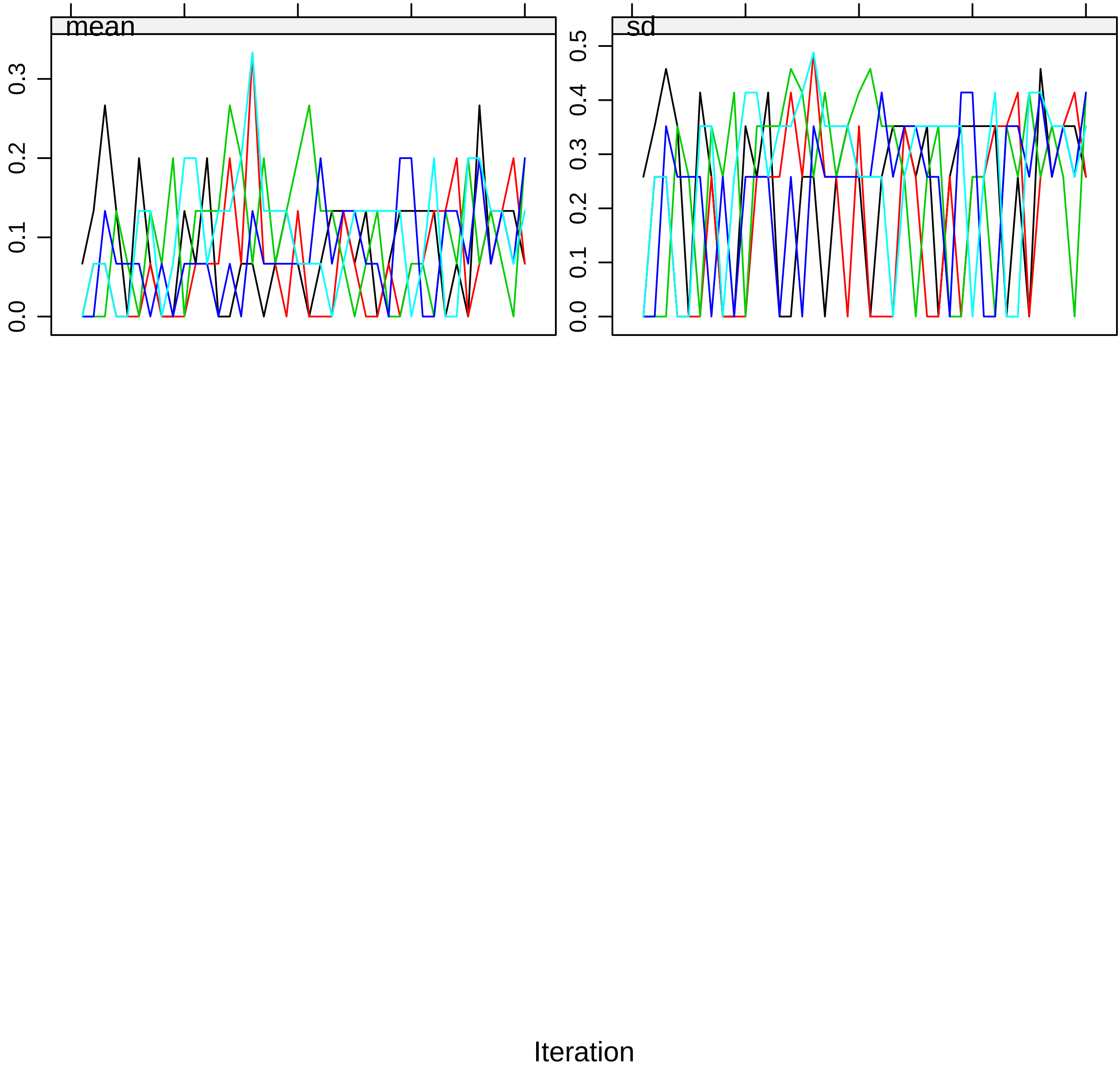

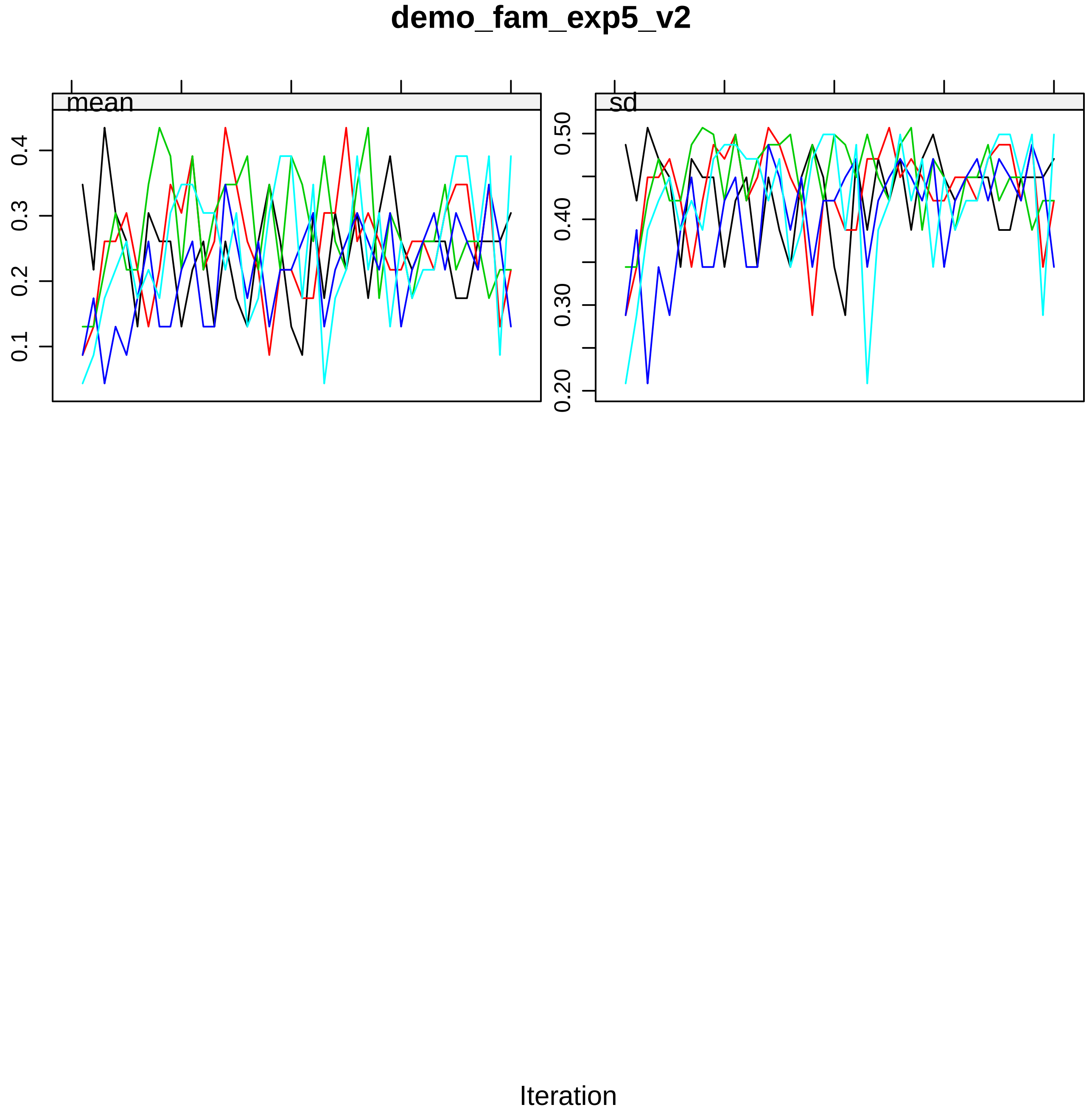

demo_fam_exp5_v2
mean
sd
0.1
0.2
0.3
0.4
0.20
0.30
0.40
0.50
Iteration

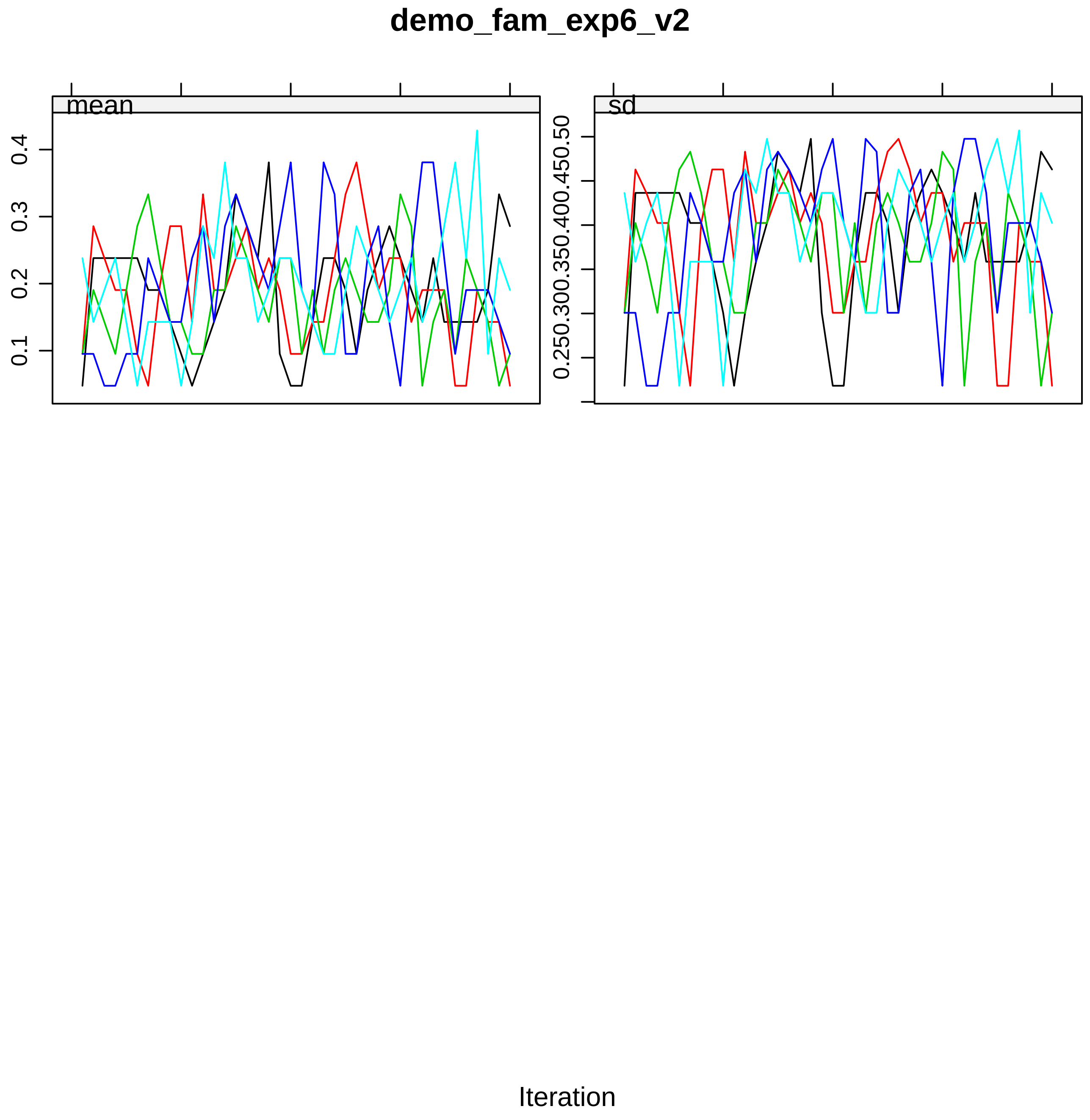
demo_fam_exp6_v2
mean
sd
0.1
0.2
0.3
0.4
0.25
0.30
0.35
0.40
0.45
0.50
Iteration

demo_fam_exp7_v2

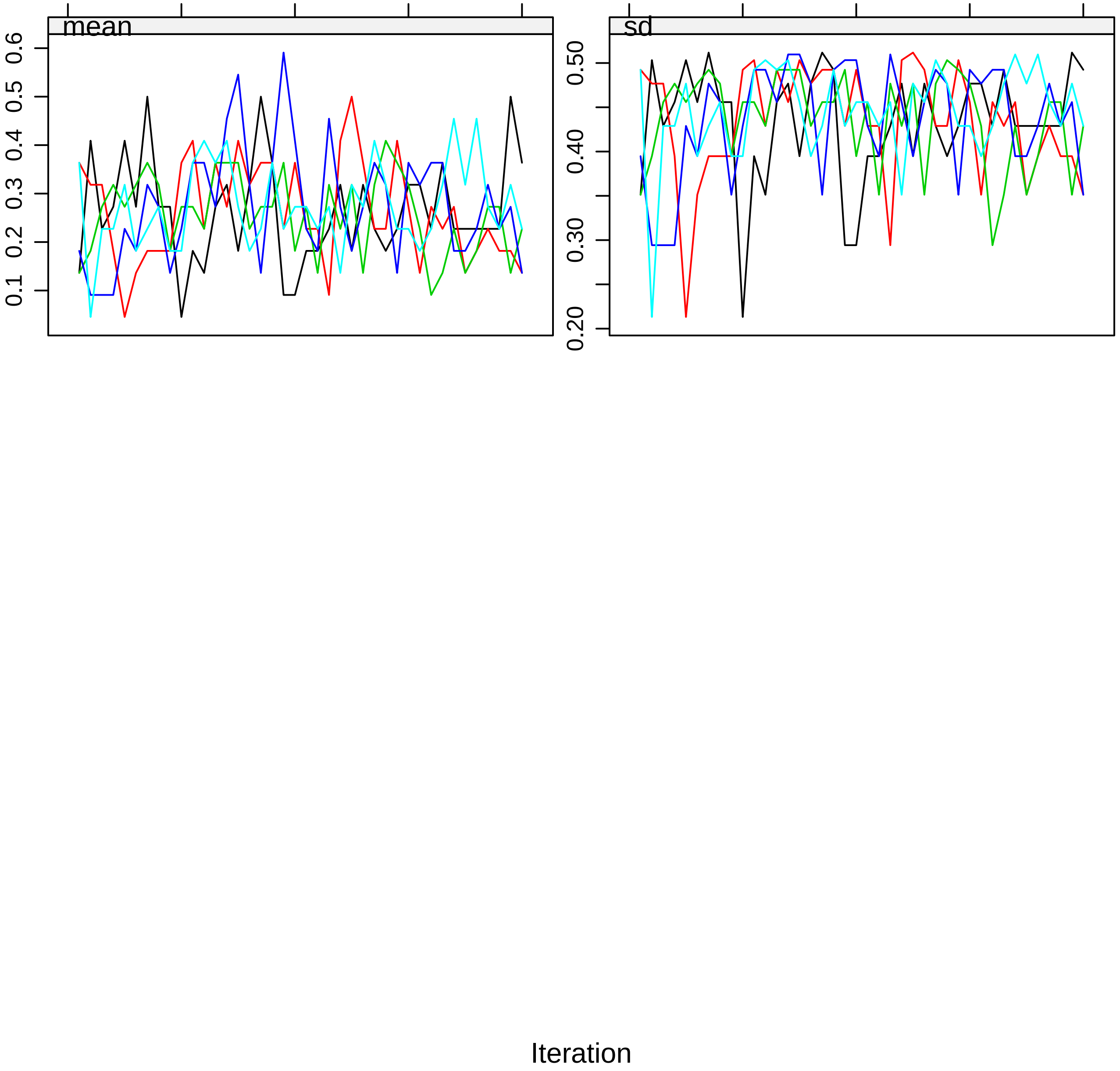

demo_roster_v2

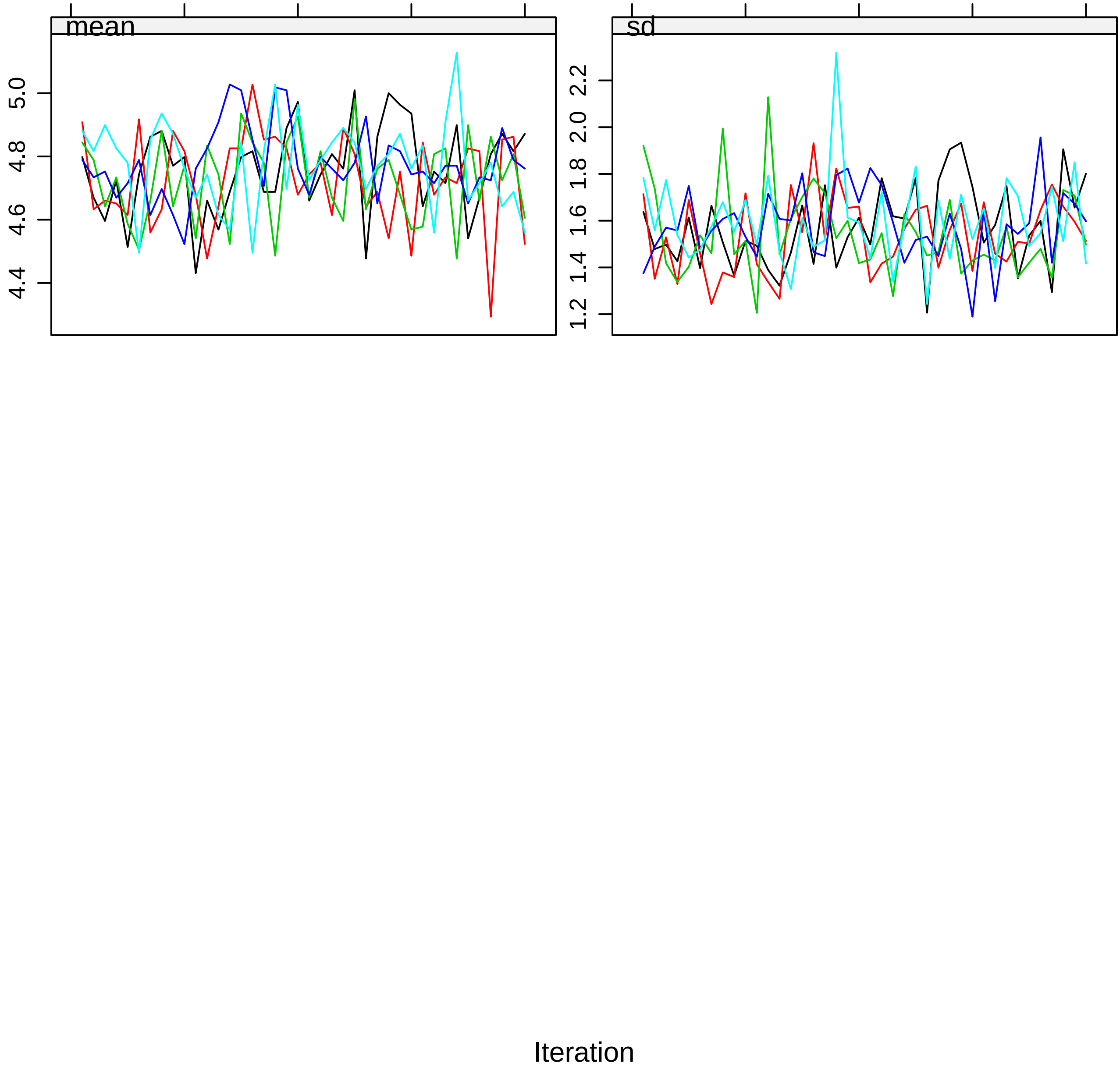

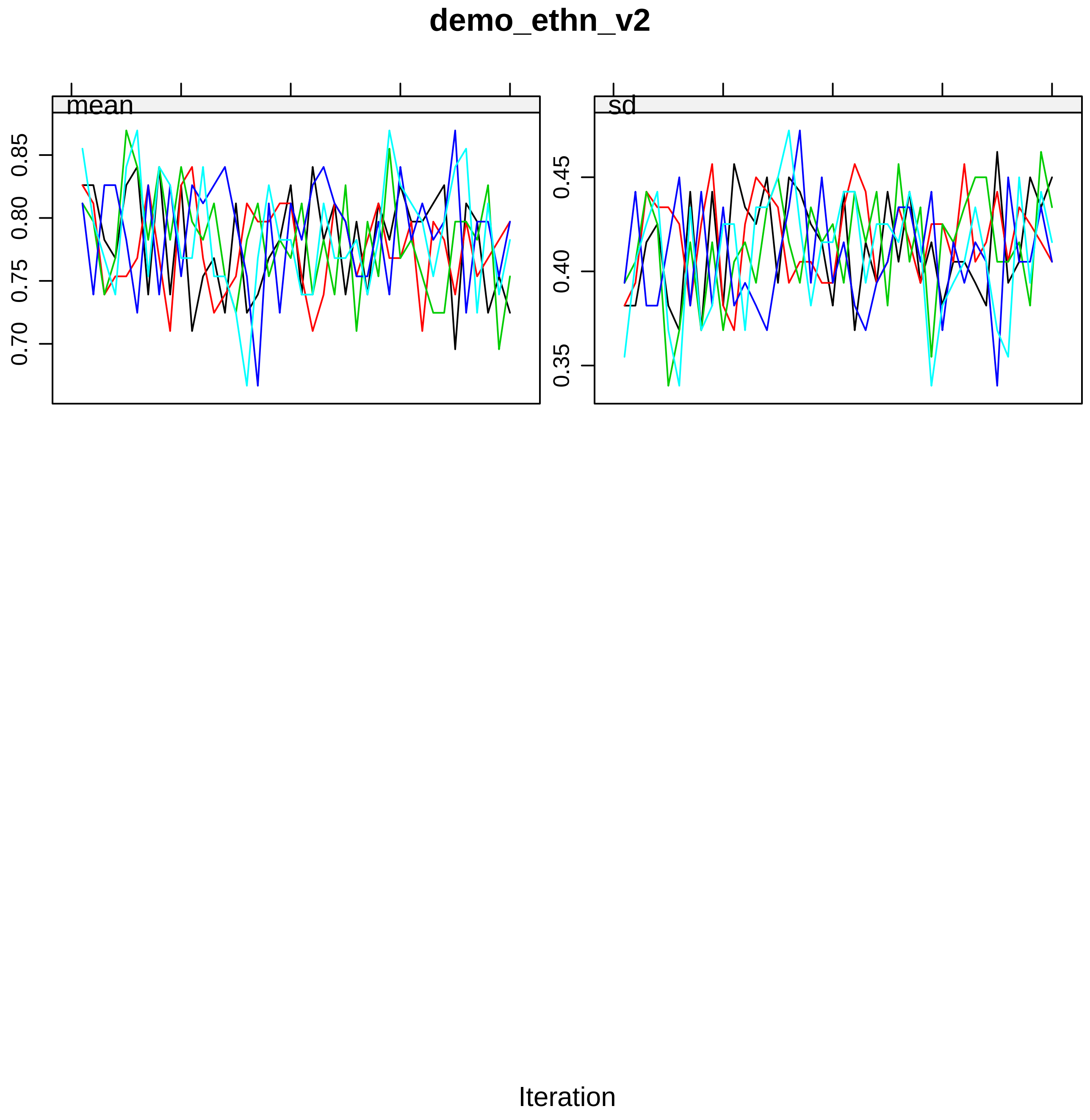
demo_ethn_v2
mean
sd
0.70
0.75
0.80
0.85
0.35
0.40
0.45
Iteration

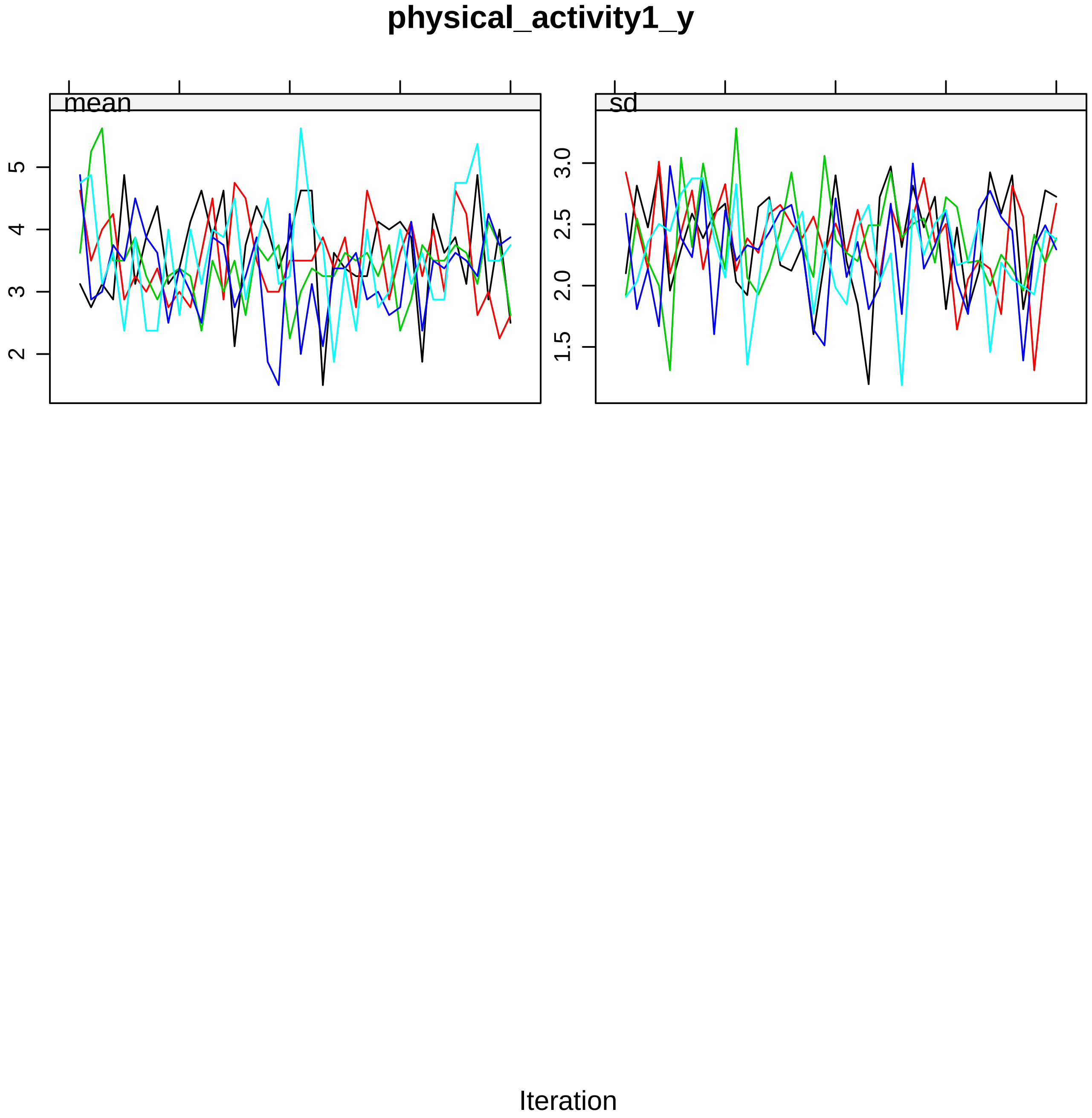
physical_activity1_y
mean
sd
5
4
3
2
3.0
2.5
2.0
1.5
Iteration

physical_activity2_y

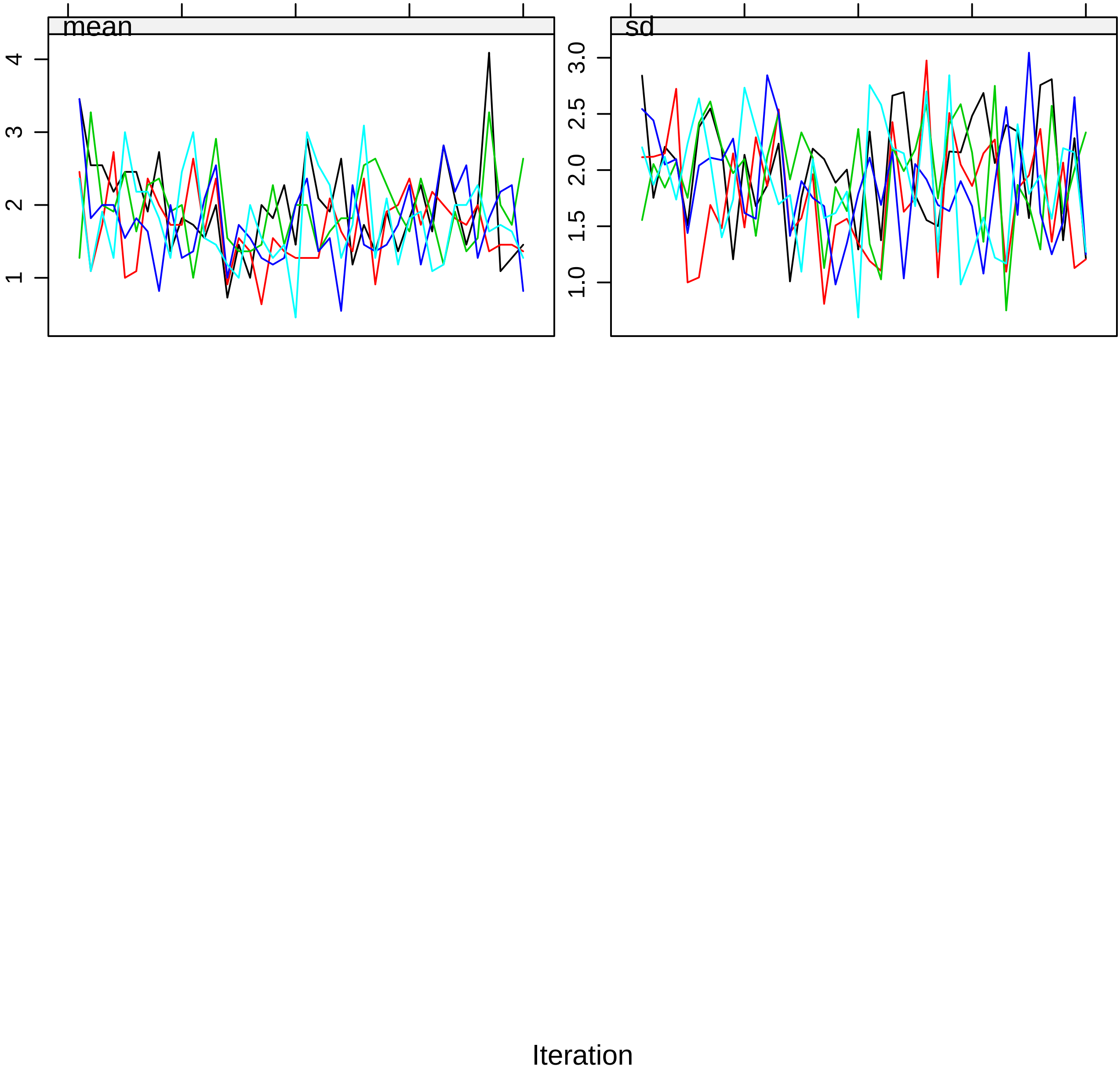

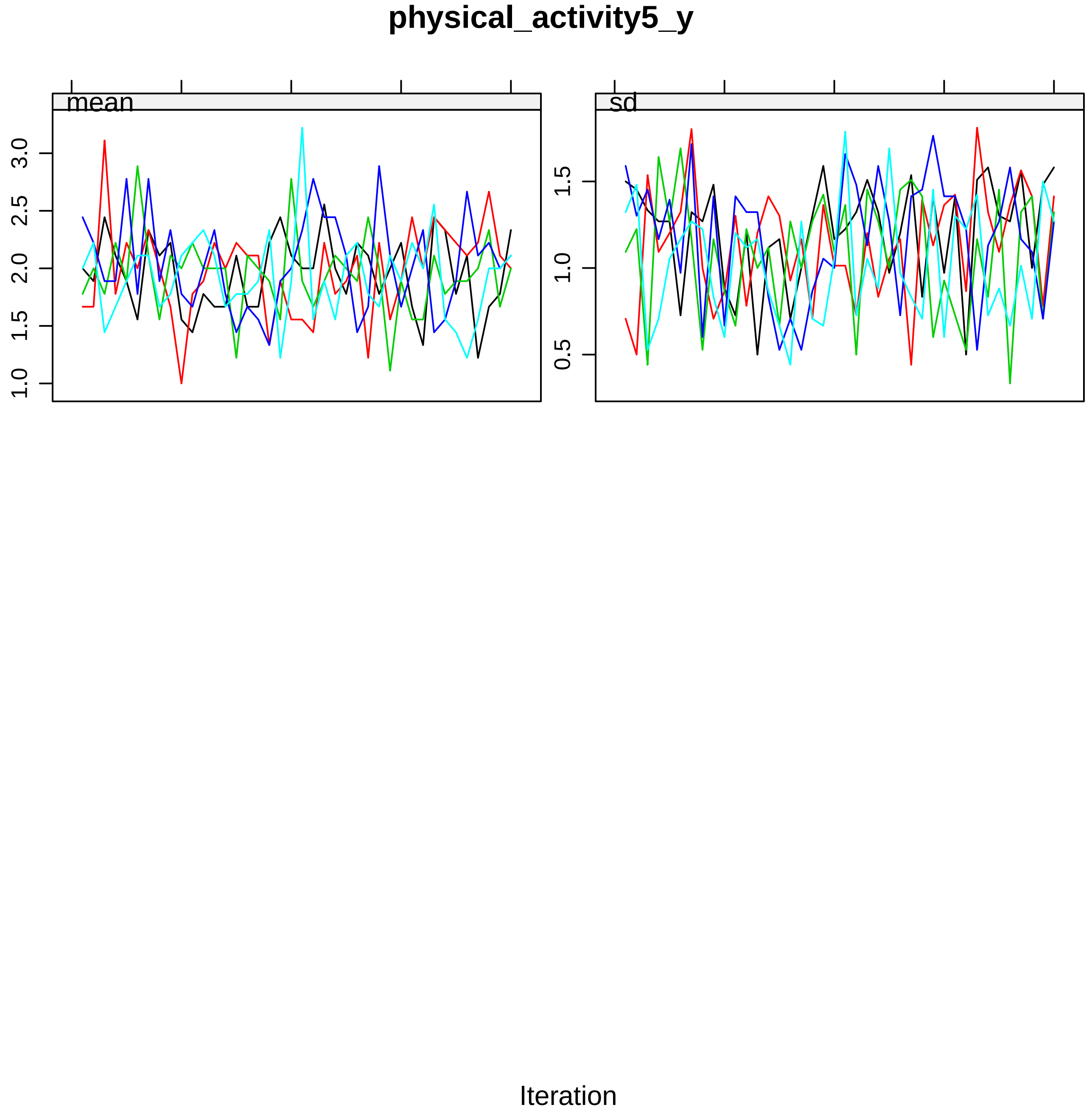
physical_activity5_y
mean
sd
1.0
1.5
2.0
2.5
3.0
0.5
1.0
1.5
Iteration

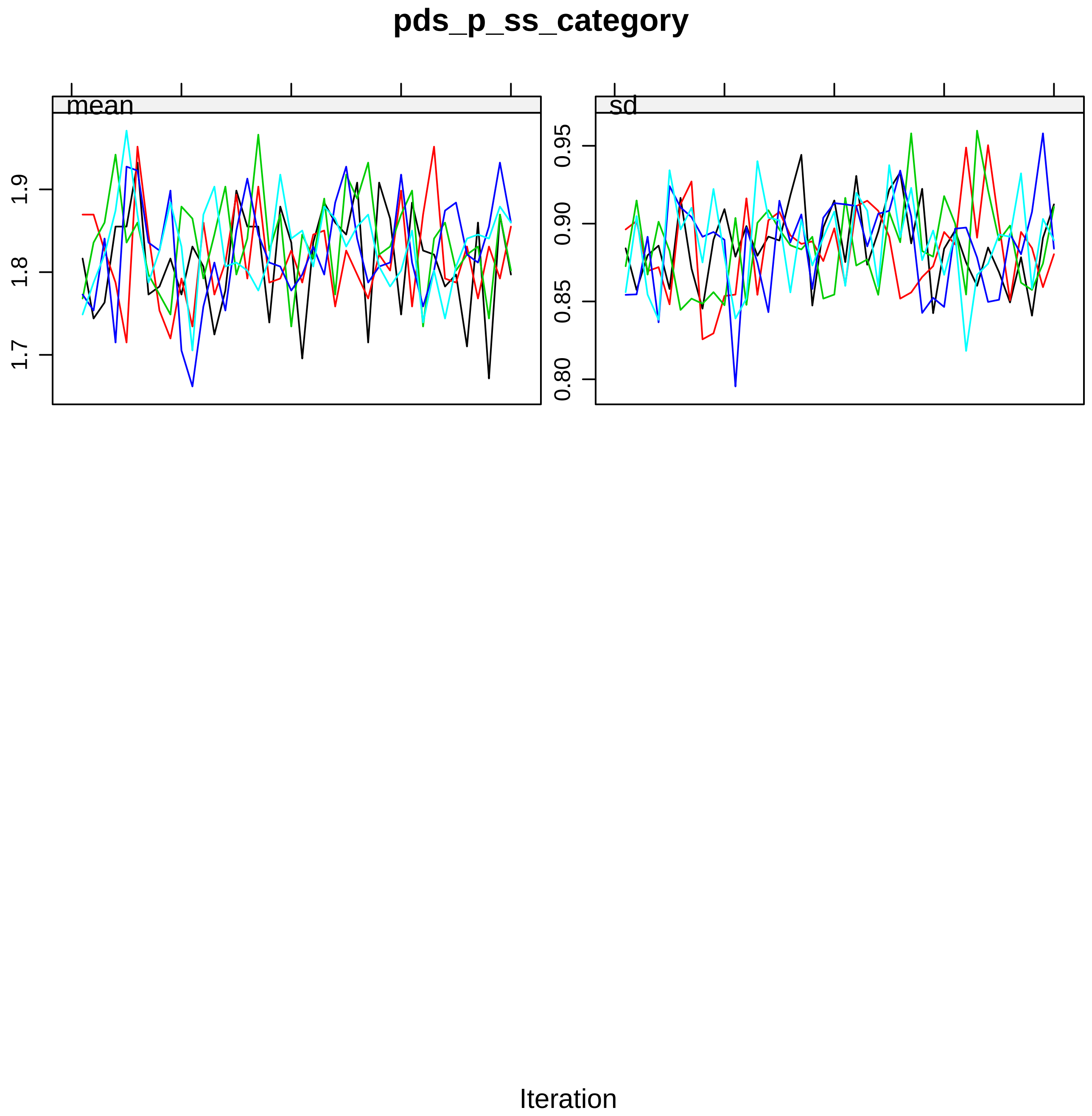

pds_p_ss_category
mean
sd
1.7
1.8
1.9
0.80
0.85
0.90
0.95
Iteration

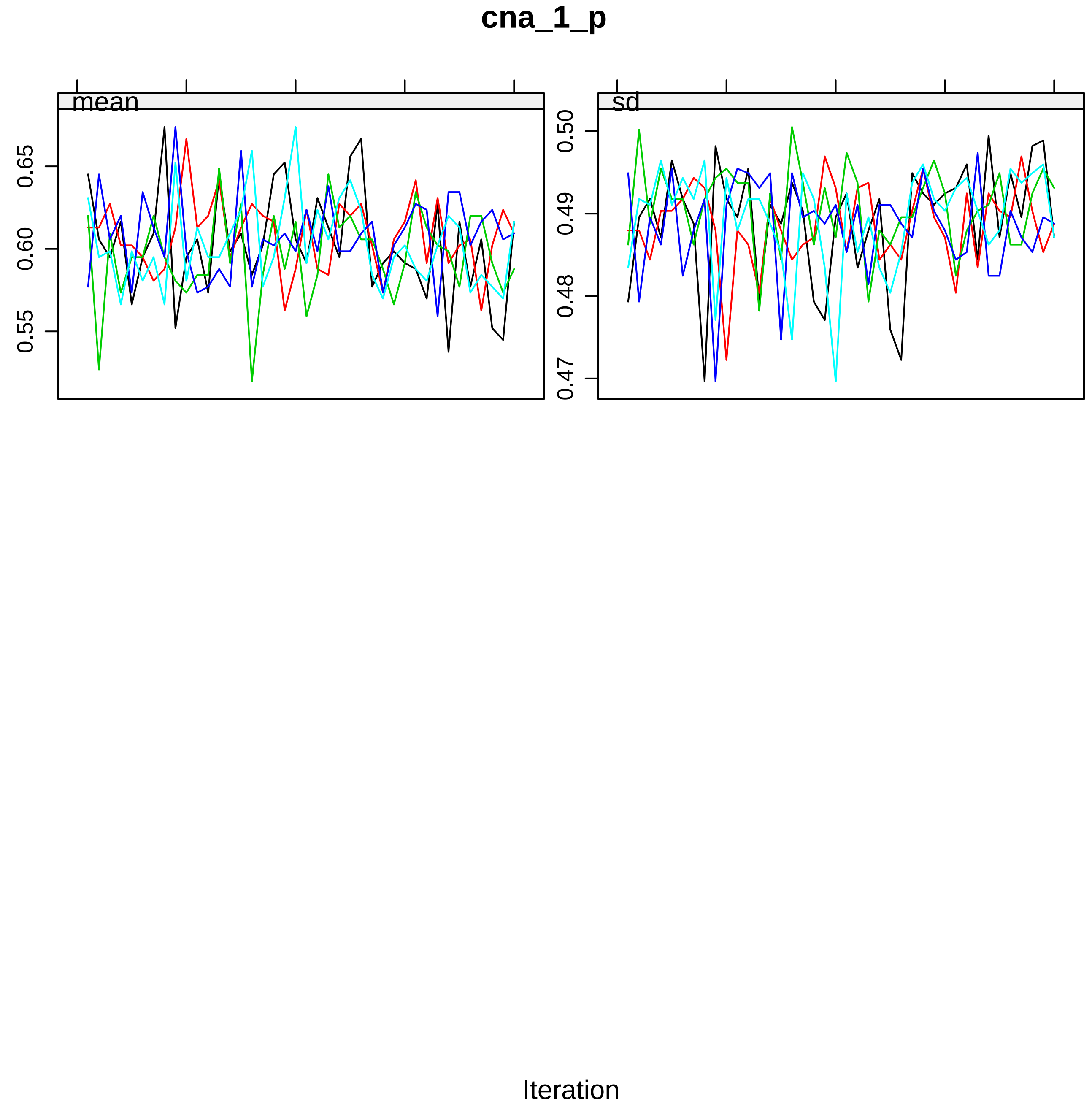

cna_1_p
mean
sd
0.55
0.60
0.65
0.47
0.48
0.49
0.50
Iteration

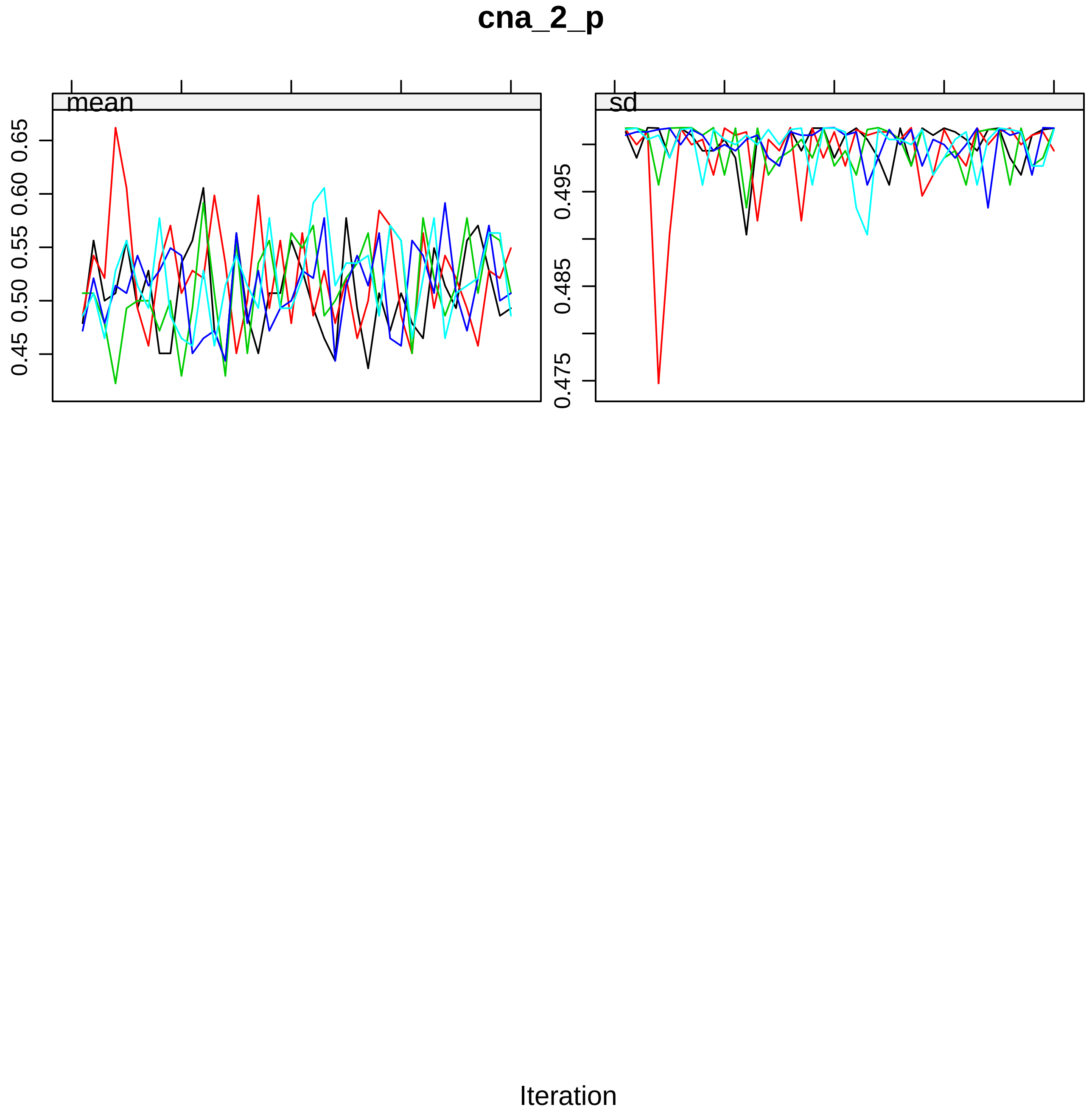

cna_2_p
mean
sd
0.45
0.50
0.55
0.60
0.65
0.475
0.485
0.495
Iteration

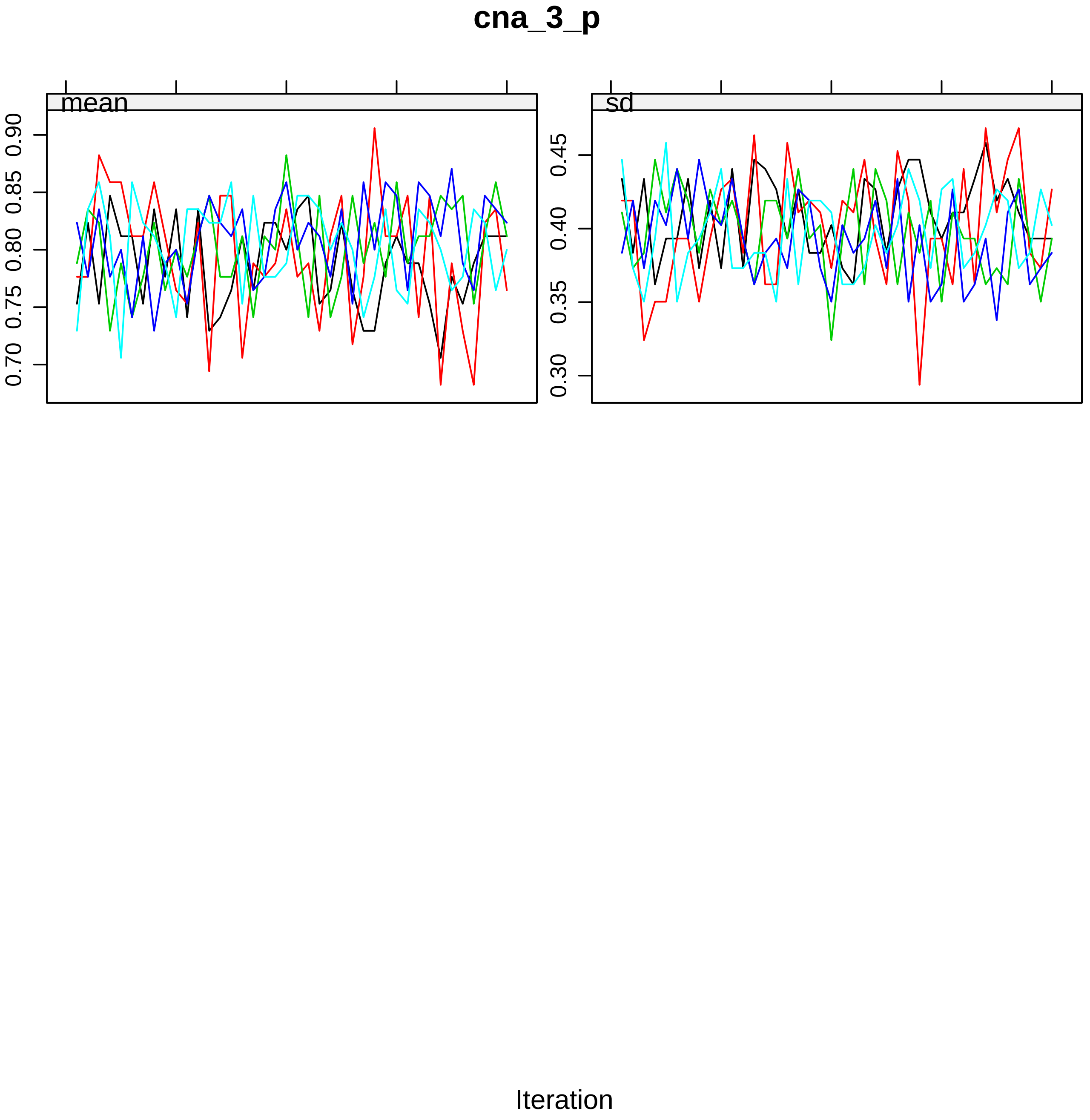

cna_3_p
mean
sd
0.70
0.75
0.80
0.85
0.90
0.30
0.35
0.40
0.45
Iteration

cna_4_p

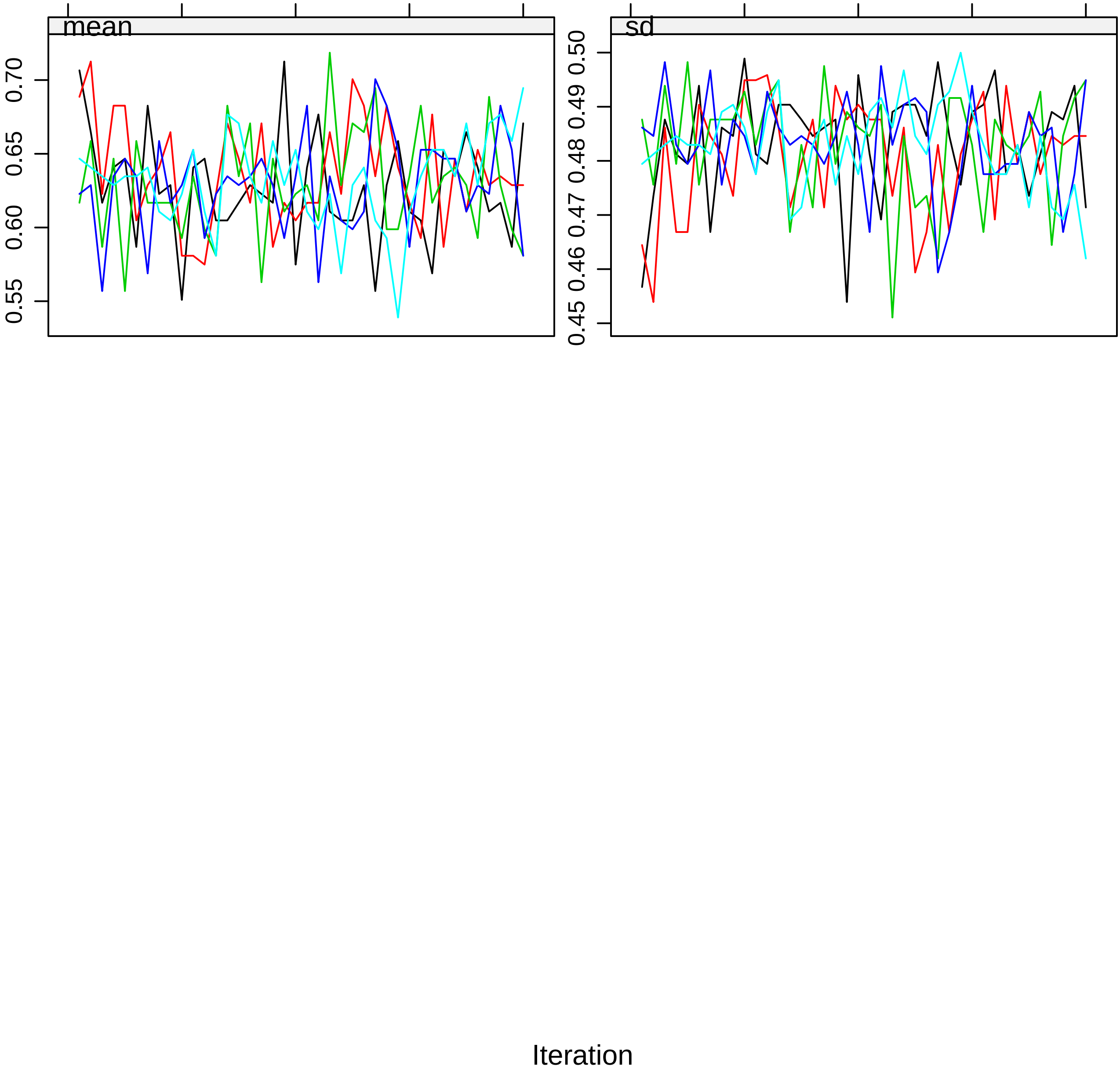

cna_5_p

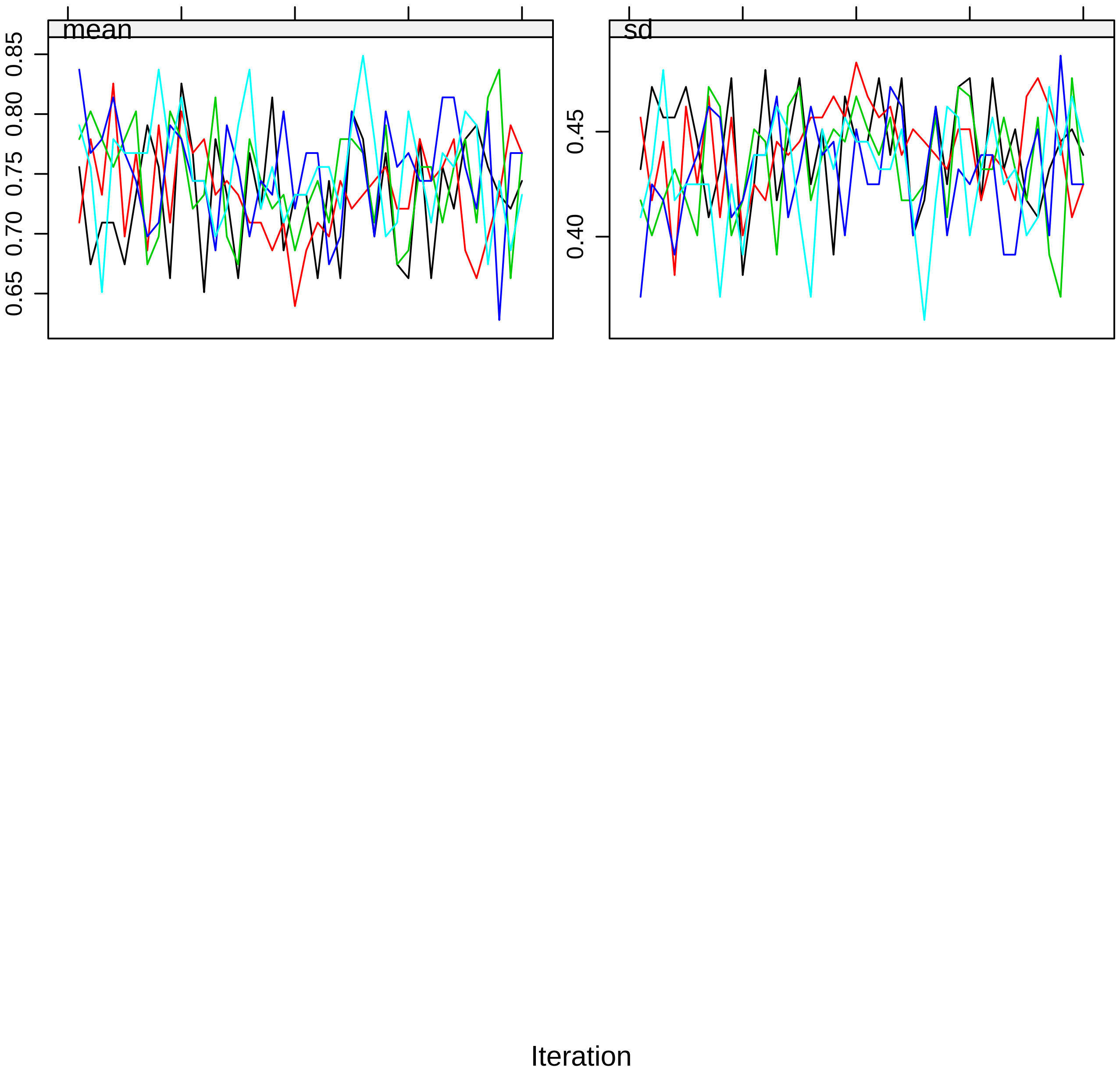

cna_6_p

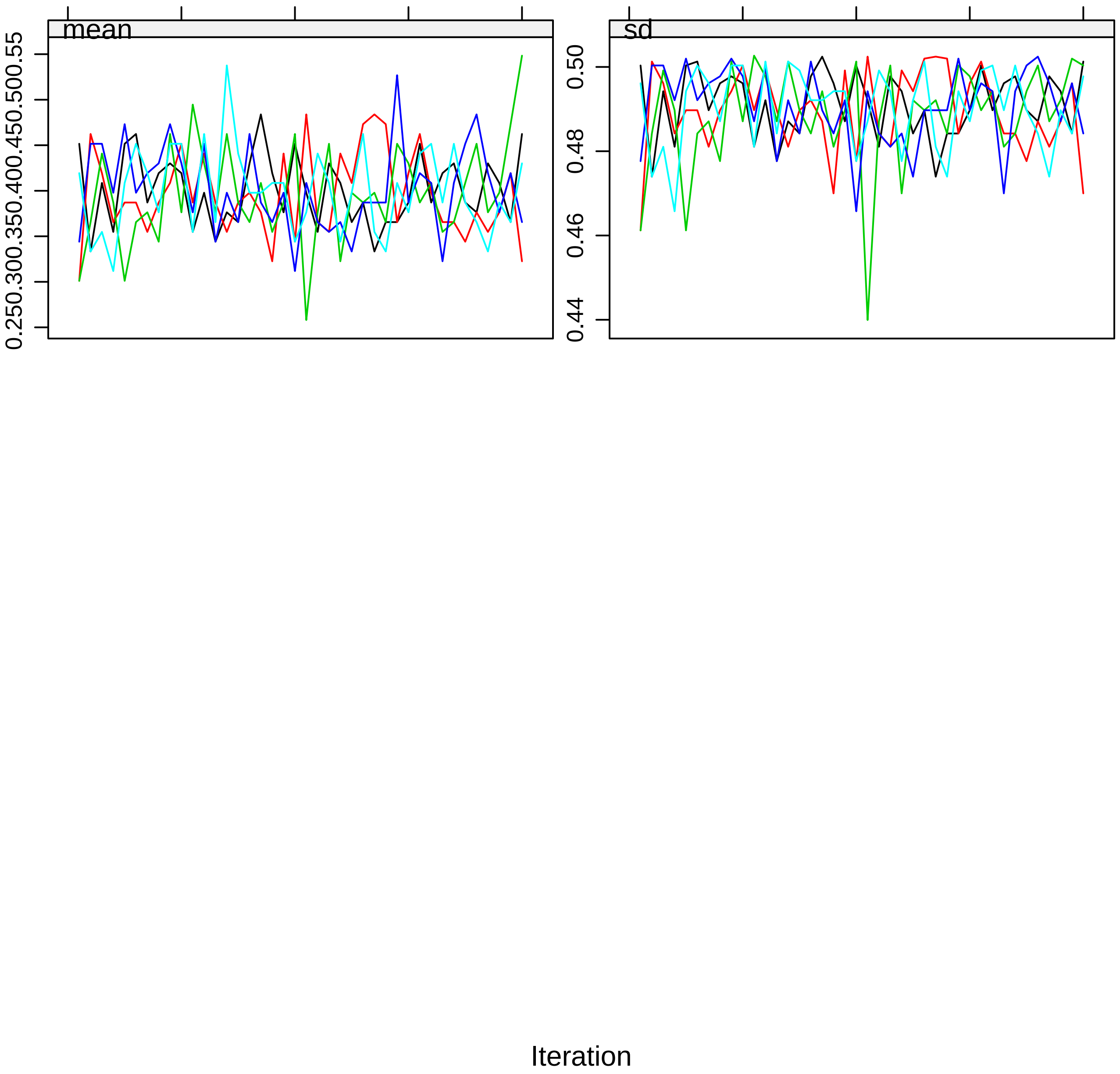

cna_7_p

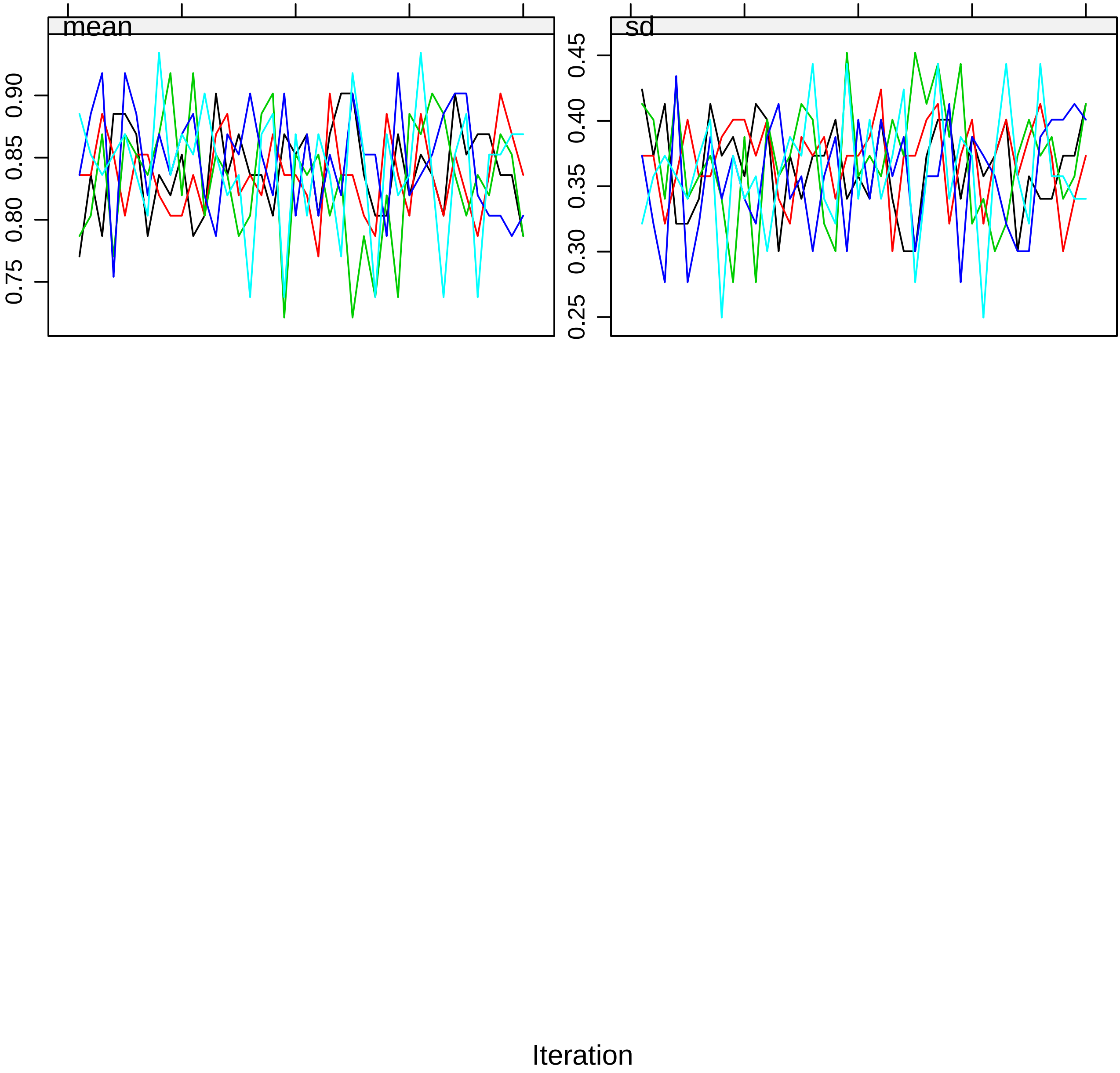

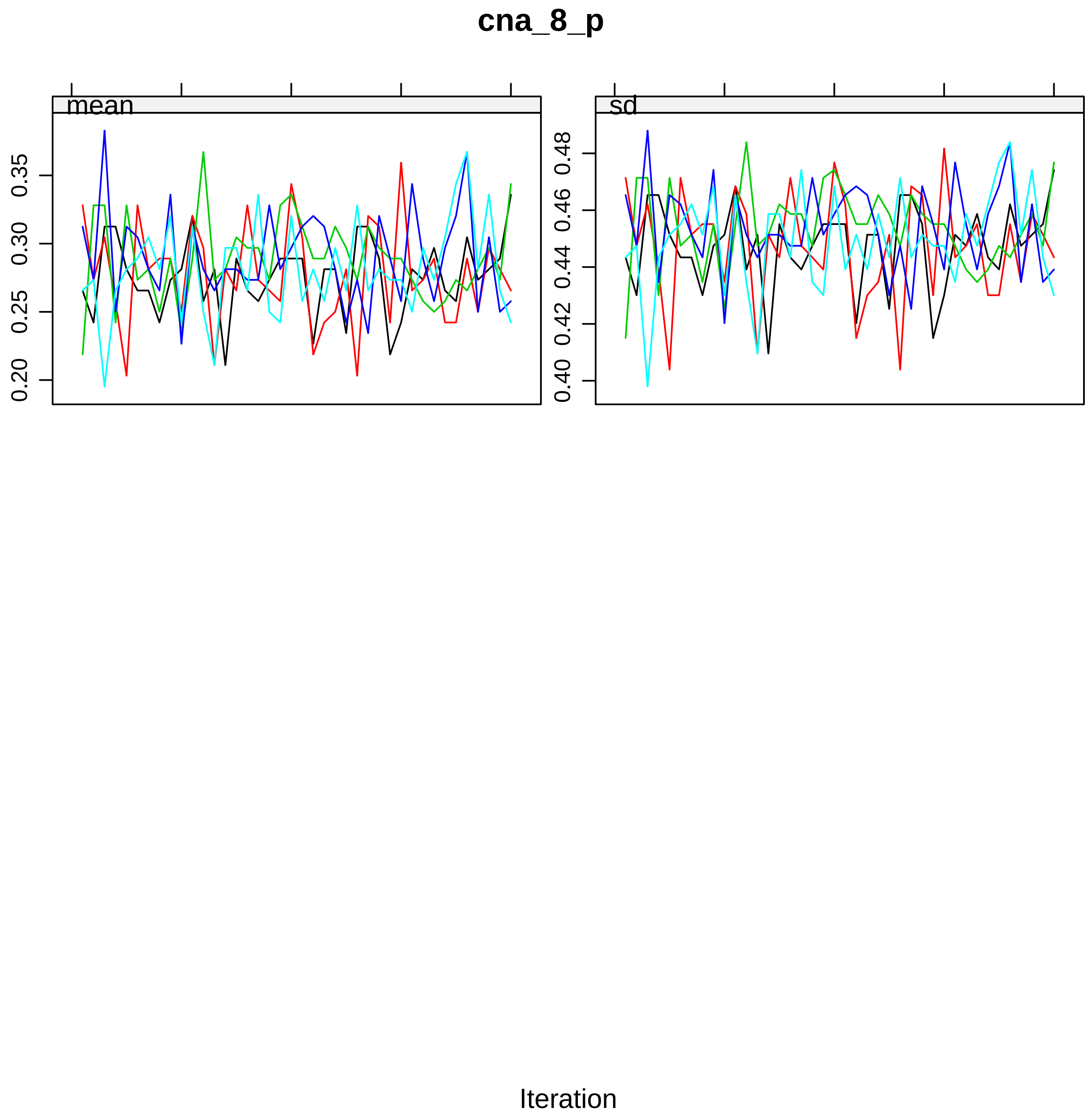

cna_8_p
mean
sd
0.20
0.25
0.30
0.35
0.40
0.42
0.44
0.46
0.48
Iteration

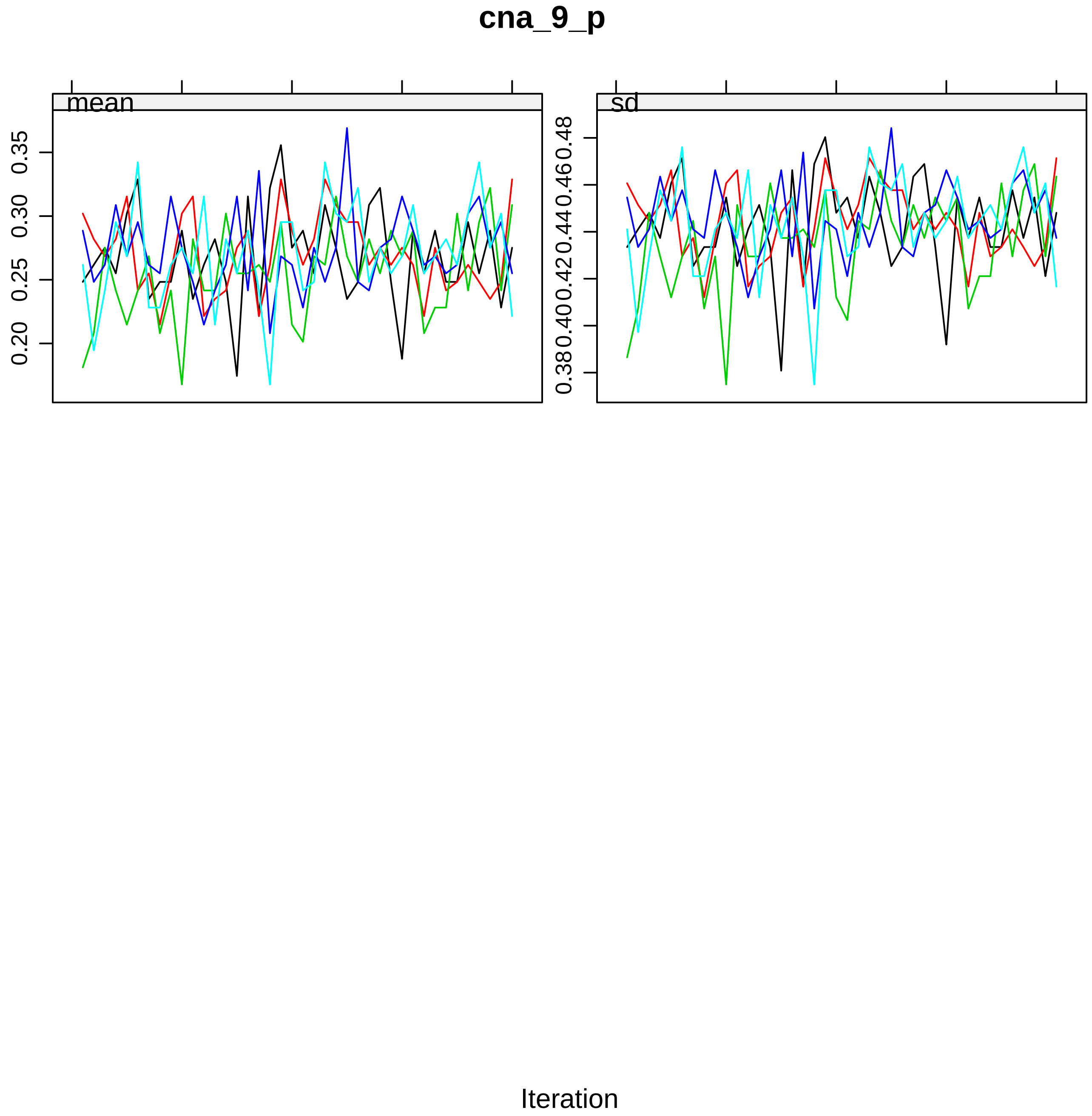
cna_9_p
mean
sd
0.20
0.25
0.30
0.35
0.380
0.400
0.420
0.440
0.460
0.48
Iteration

cna_10_p

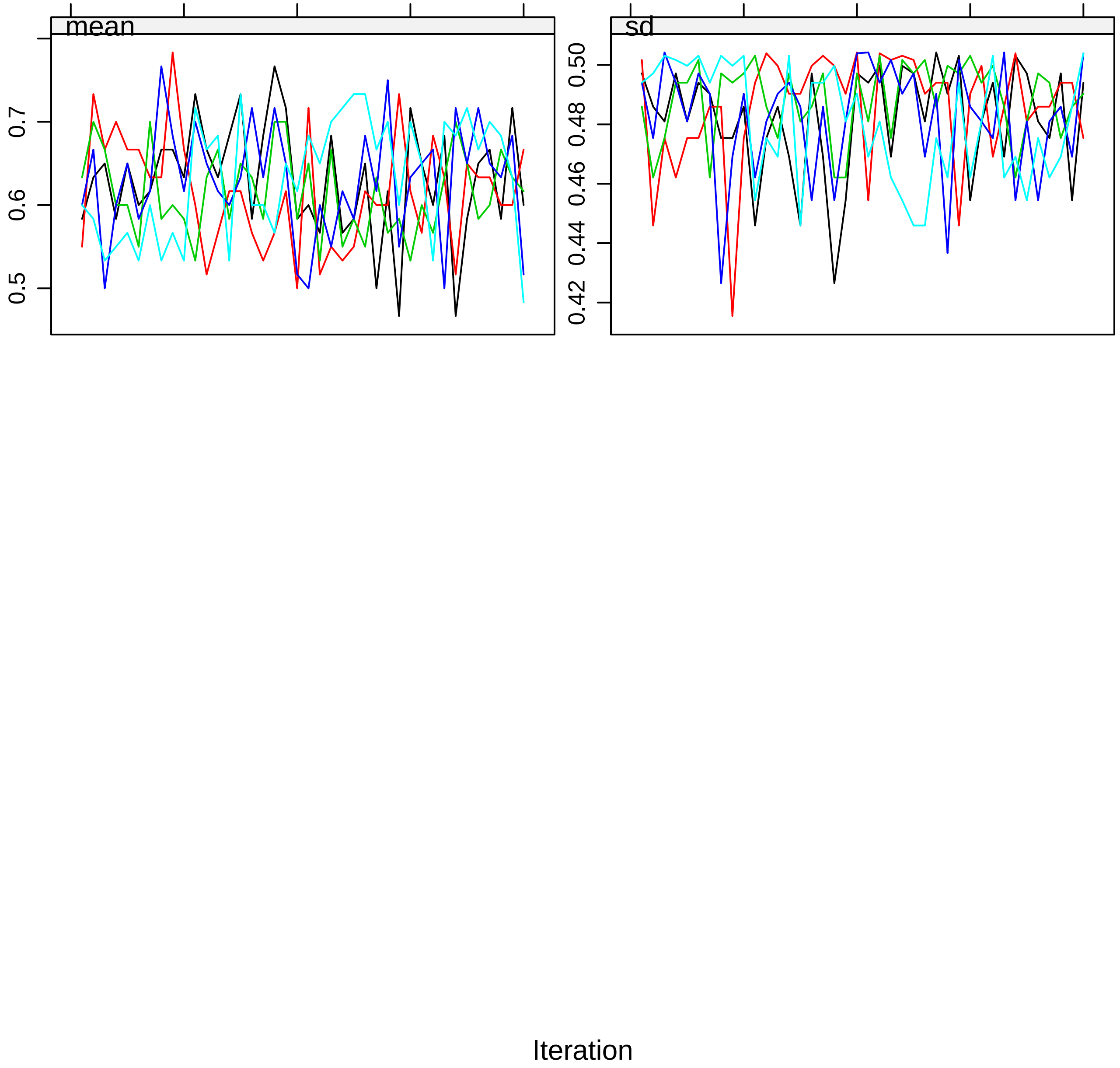

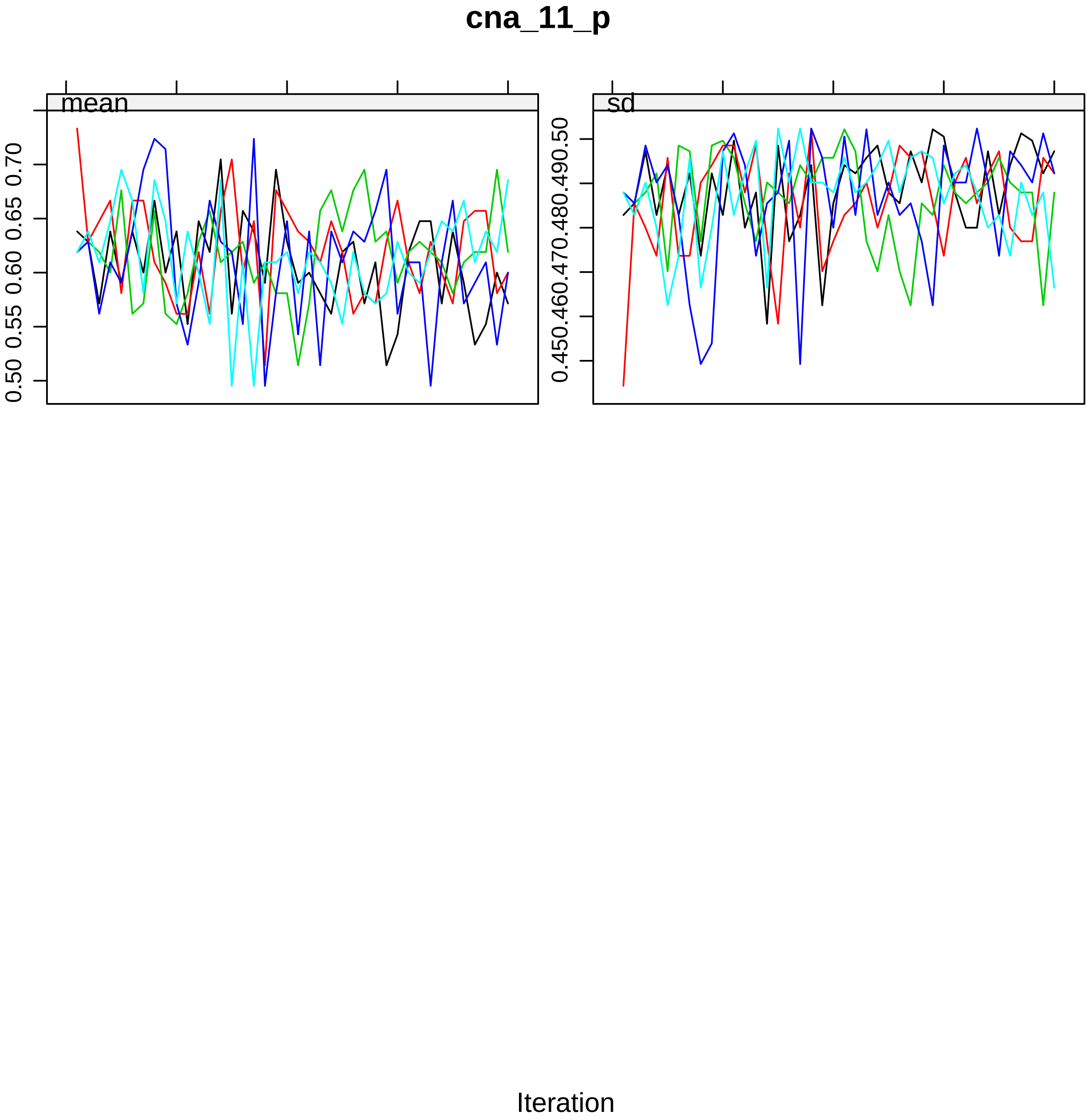
cna_11_p
mean
sd
0.50
0.55
0.60
0.65
0.70
0.45
0.46
0.47
0.48
0.49
0.50
Iteration

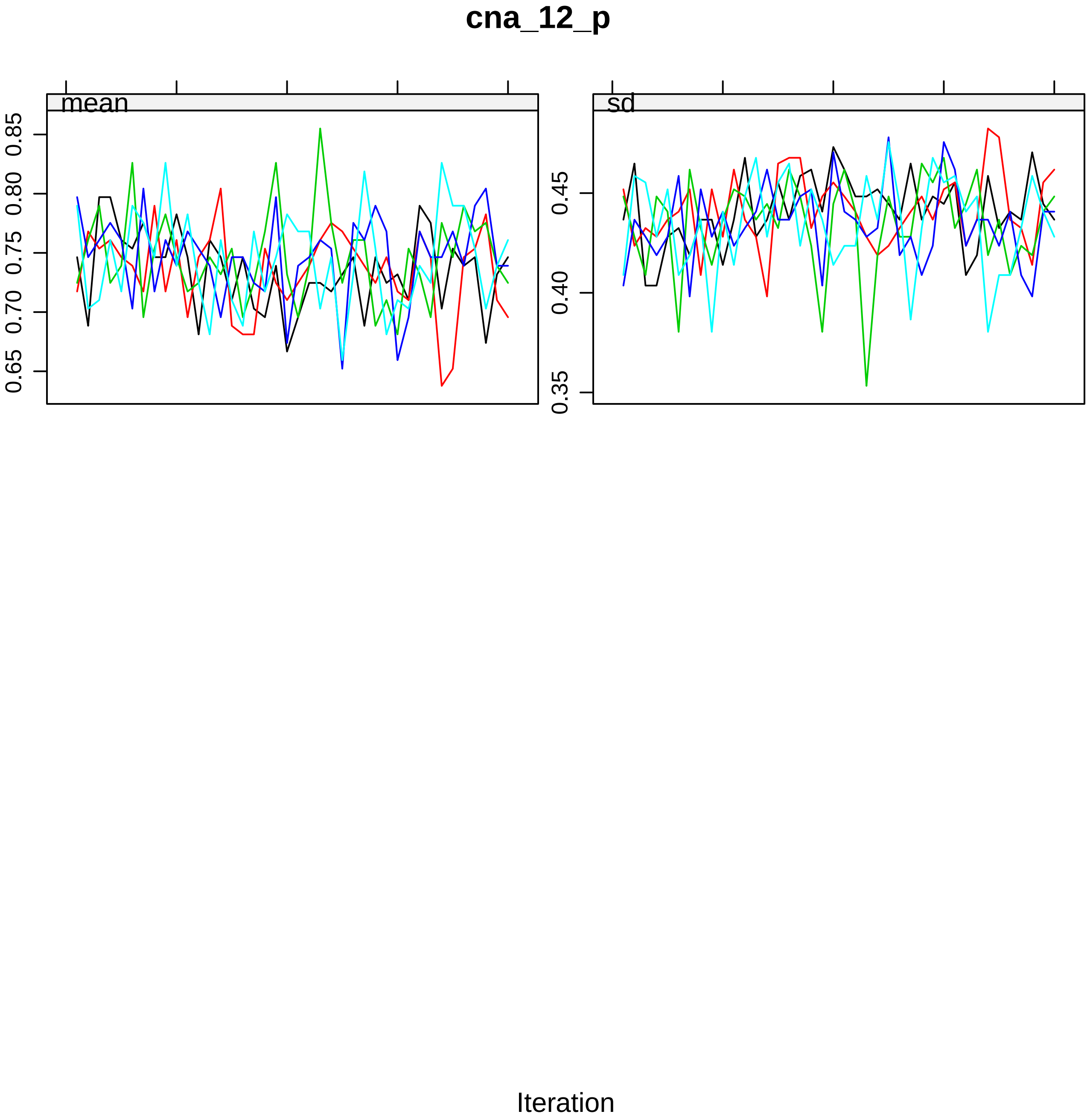

cna_12_p
mean
sd
0.65
0.70
0.75
0.80
0.85
0.35
0.40
0.45
Iteration

cna_13_p

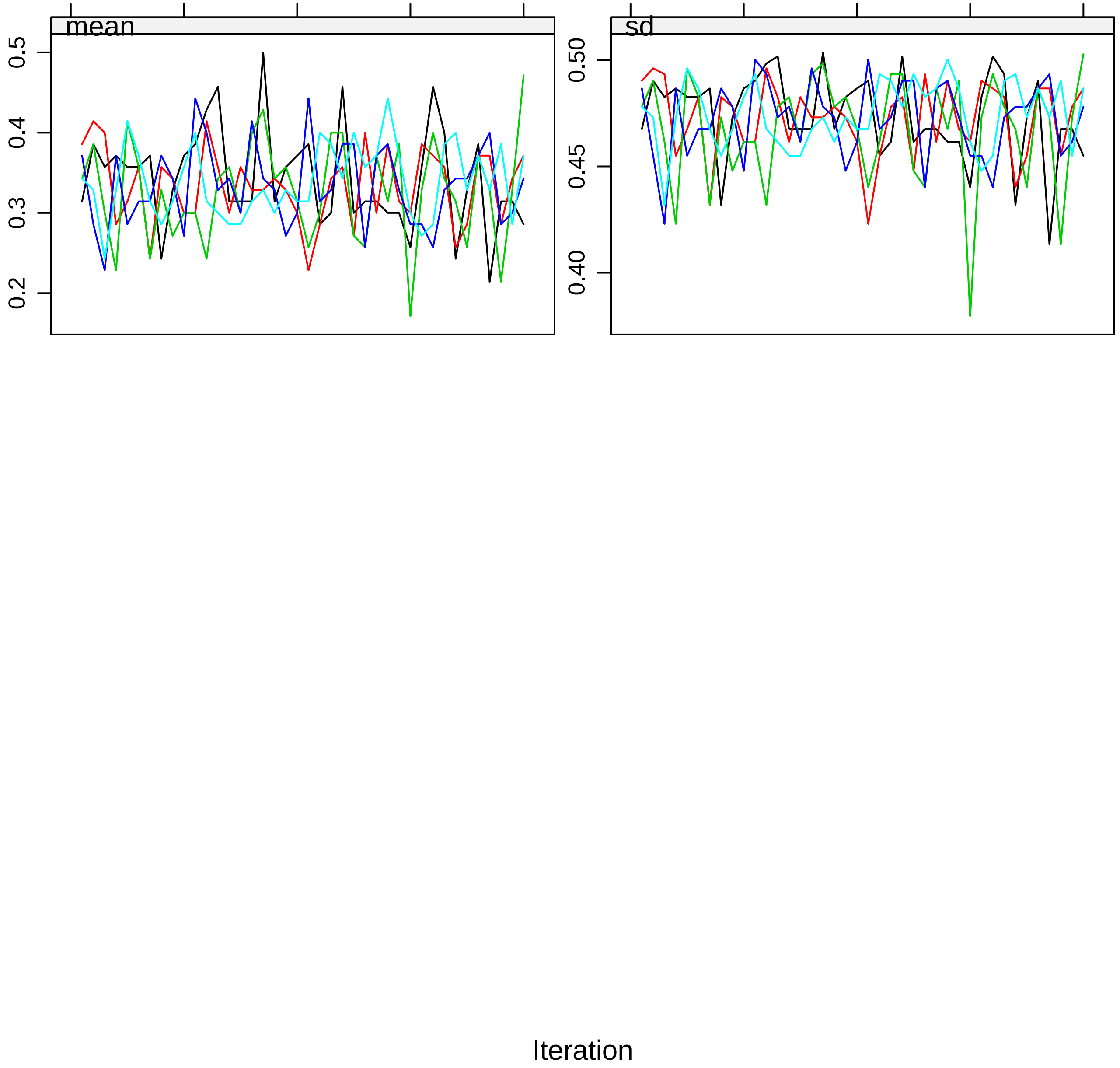

cna_14_p

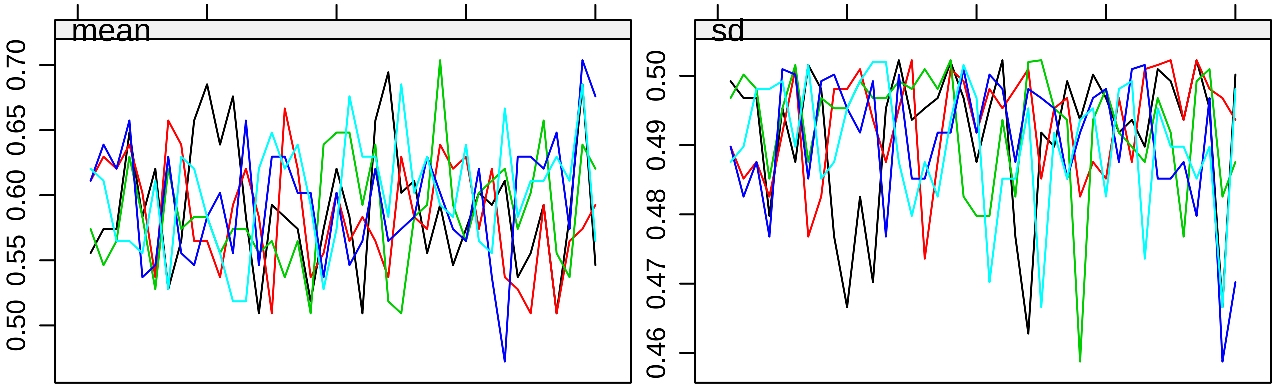

Iteration

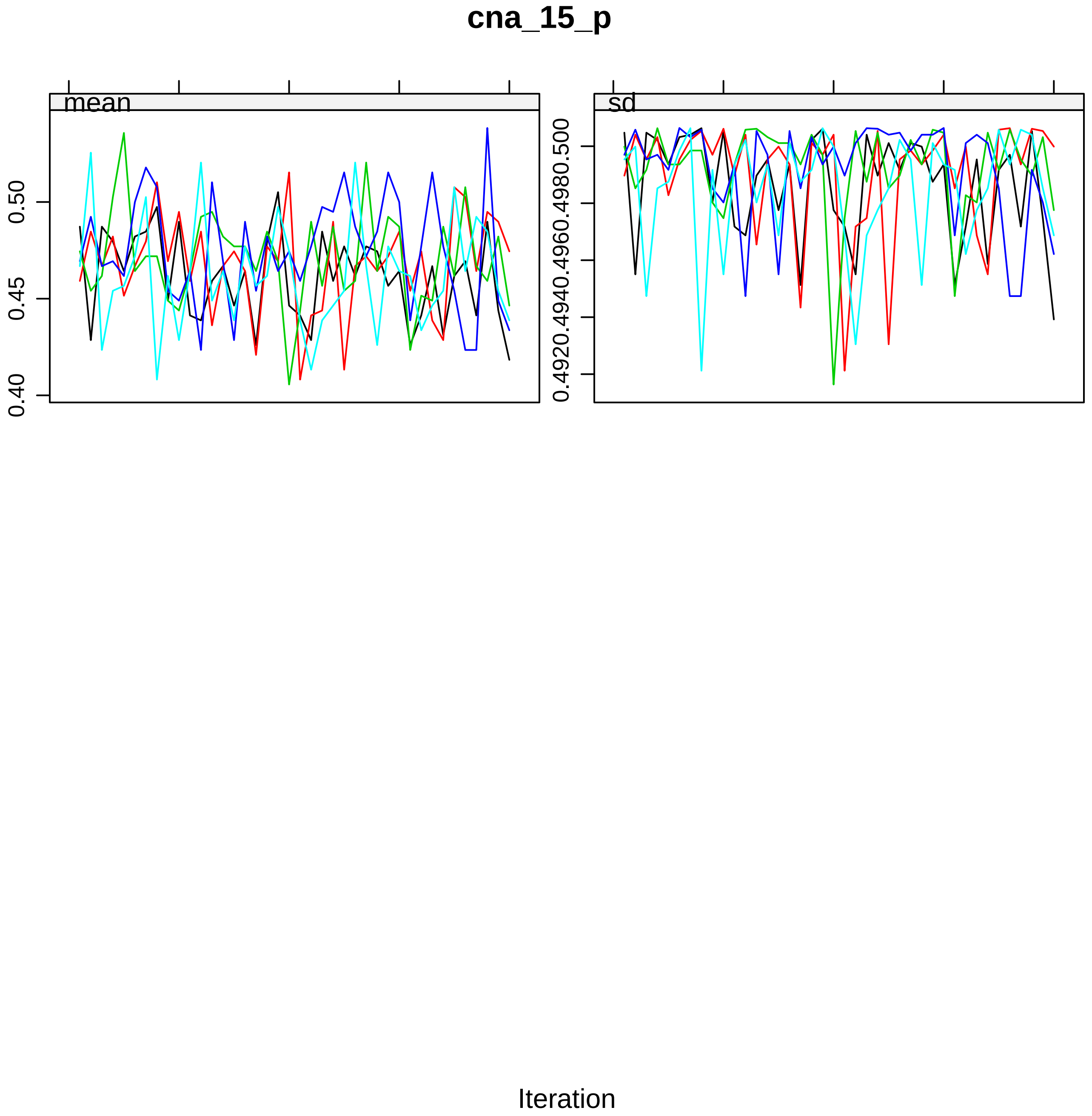
cna_15_p
mean
sd
0.40
0.45
0.50
0.4920
0.4940
0.4960
0.4980
0.500
Iteration

cna_16_p

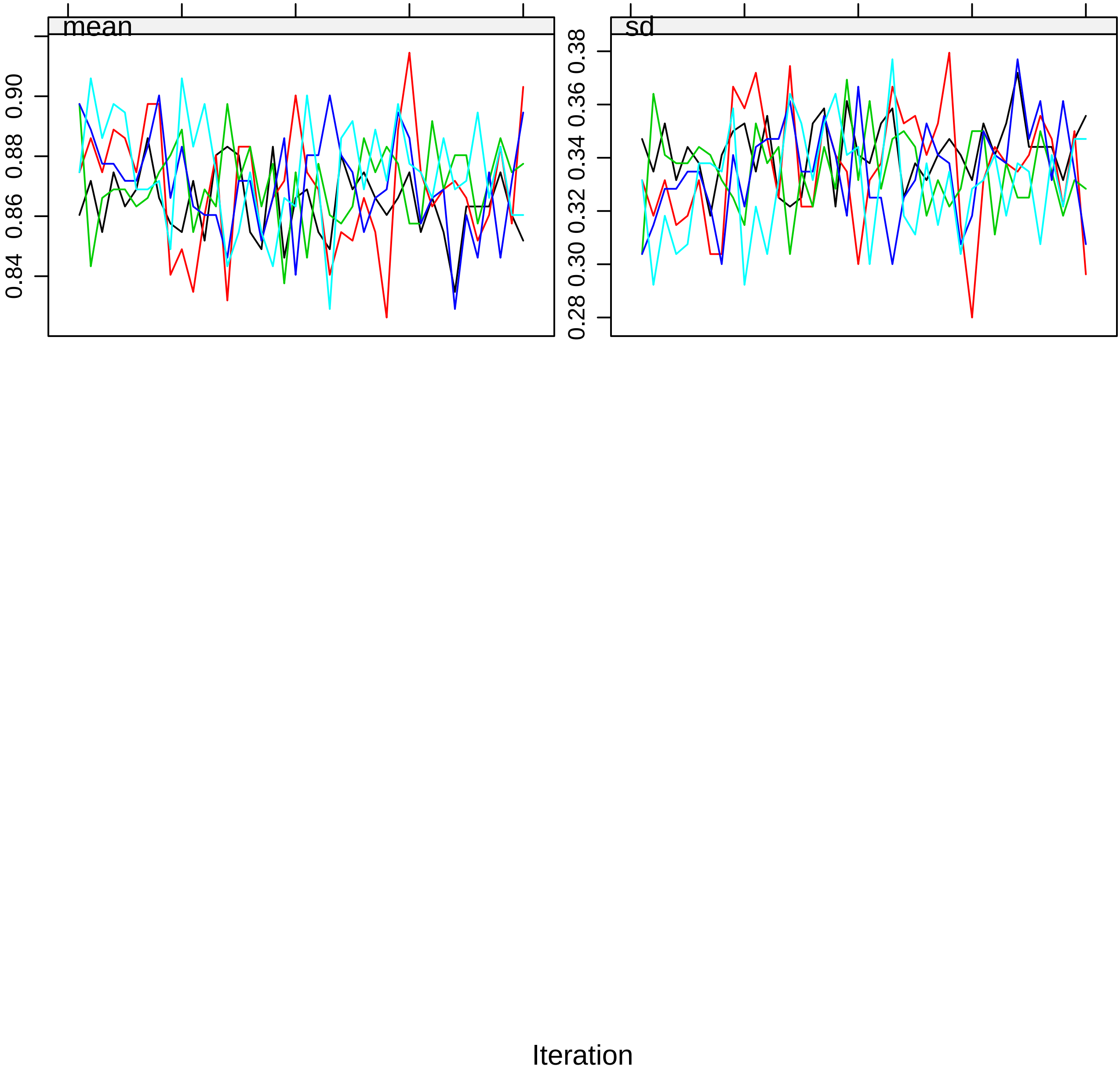

sports_hours

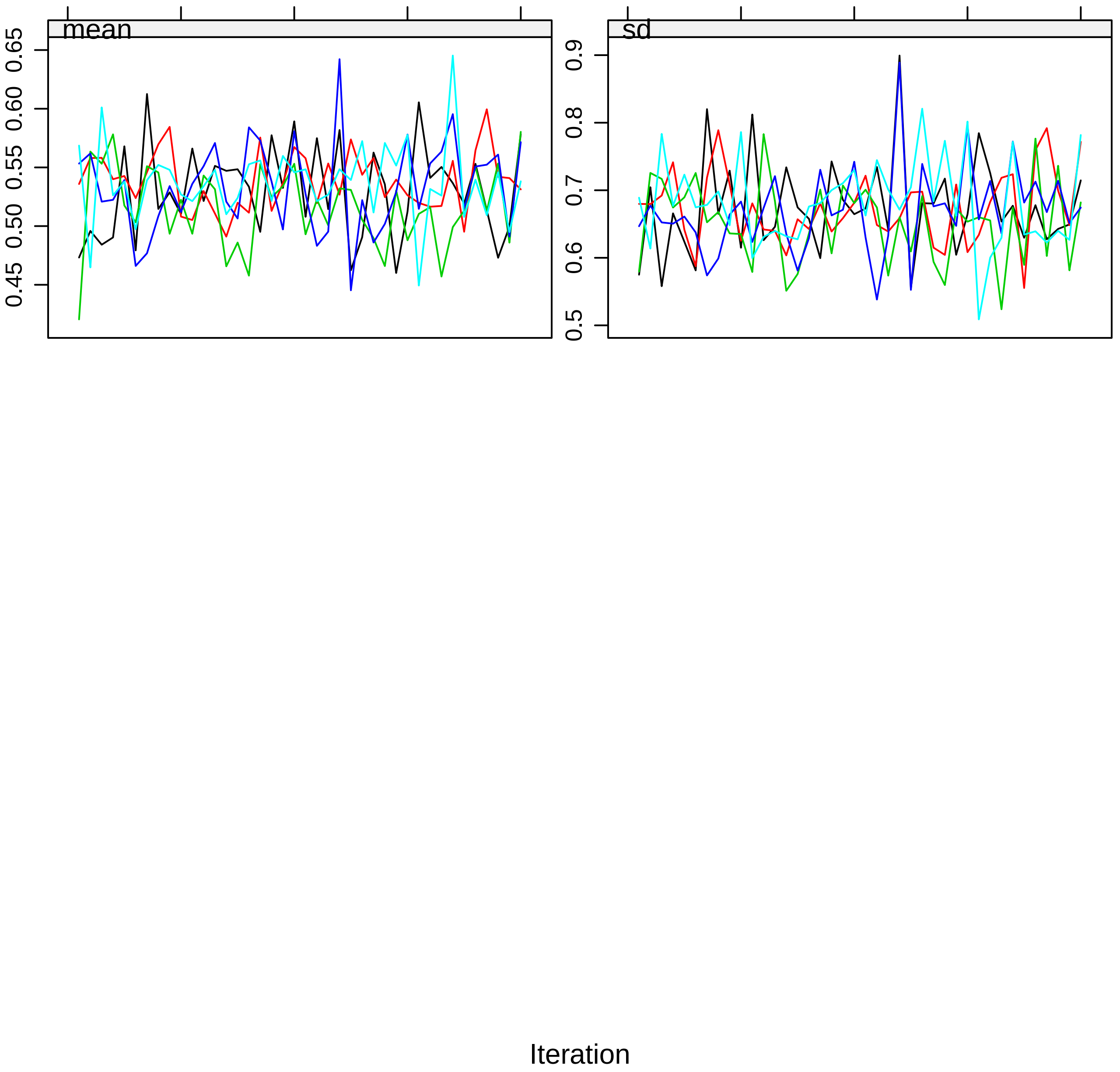

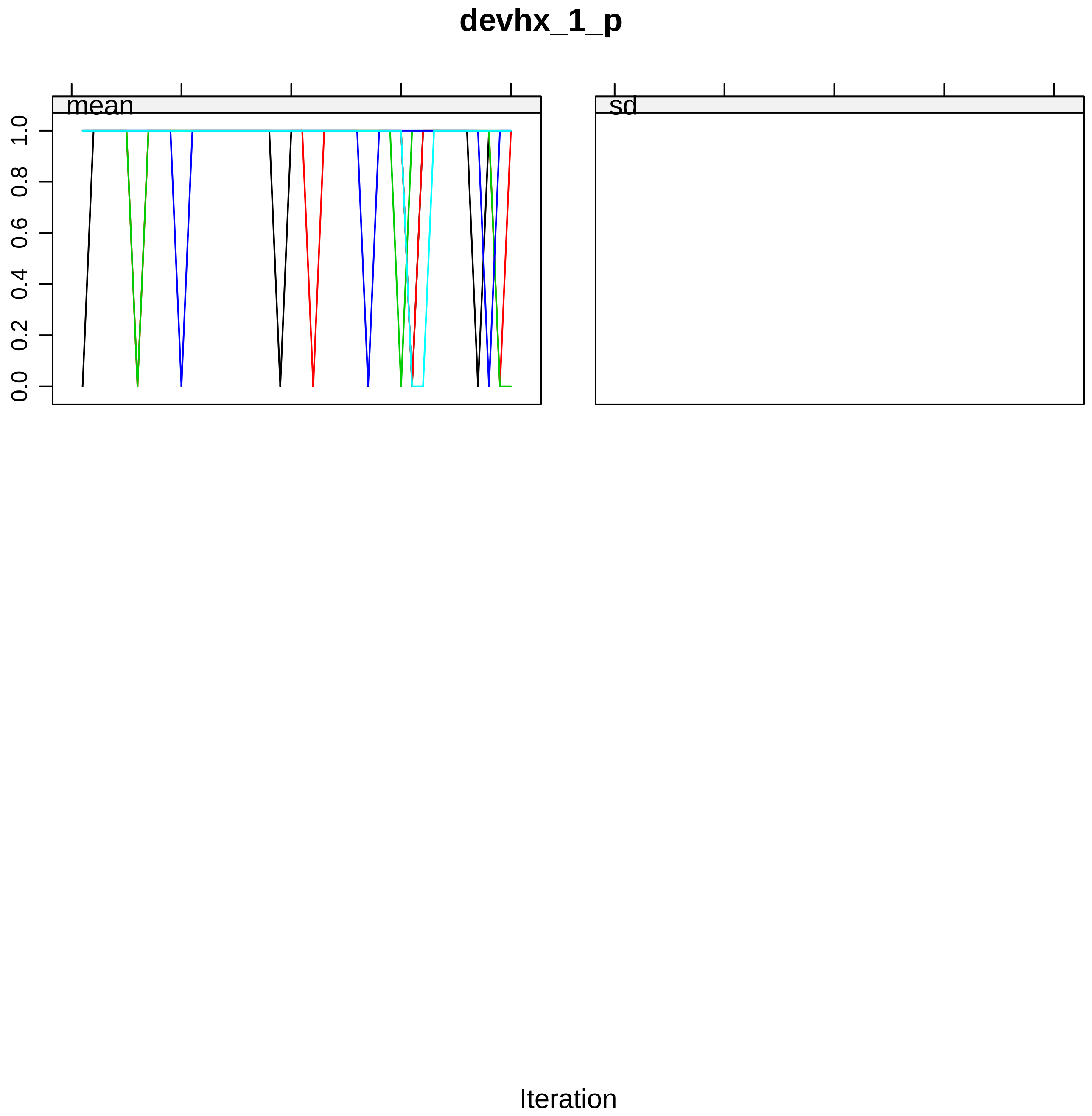
devhx_1_p
mean
sd
1.0
0.8
0.6
0.4
0.2
0.0
Iteration

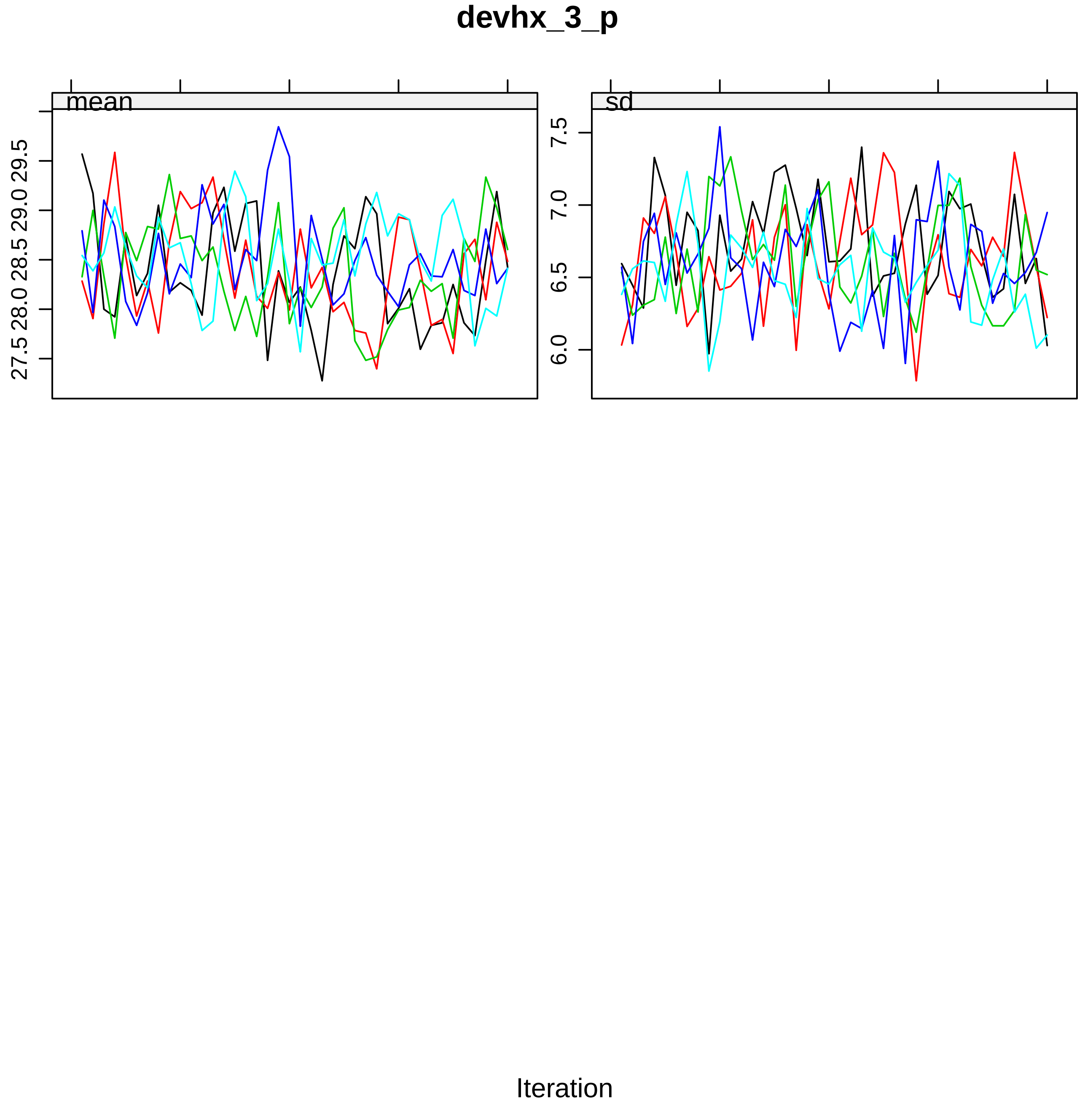
devhx_3_p
mean
sd
27.5 28.0 28.5 29.0 29.5
6.0 6.5 7.0 7.5
Iteration

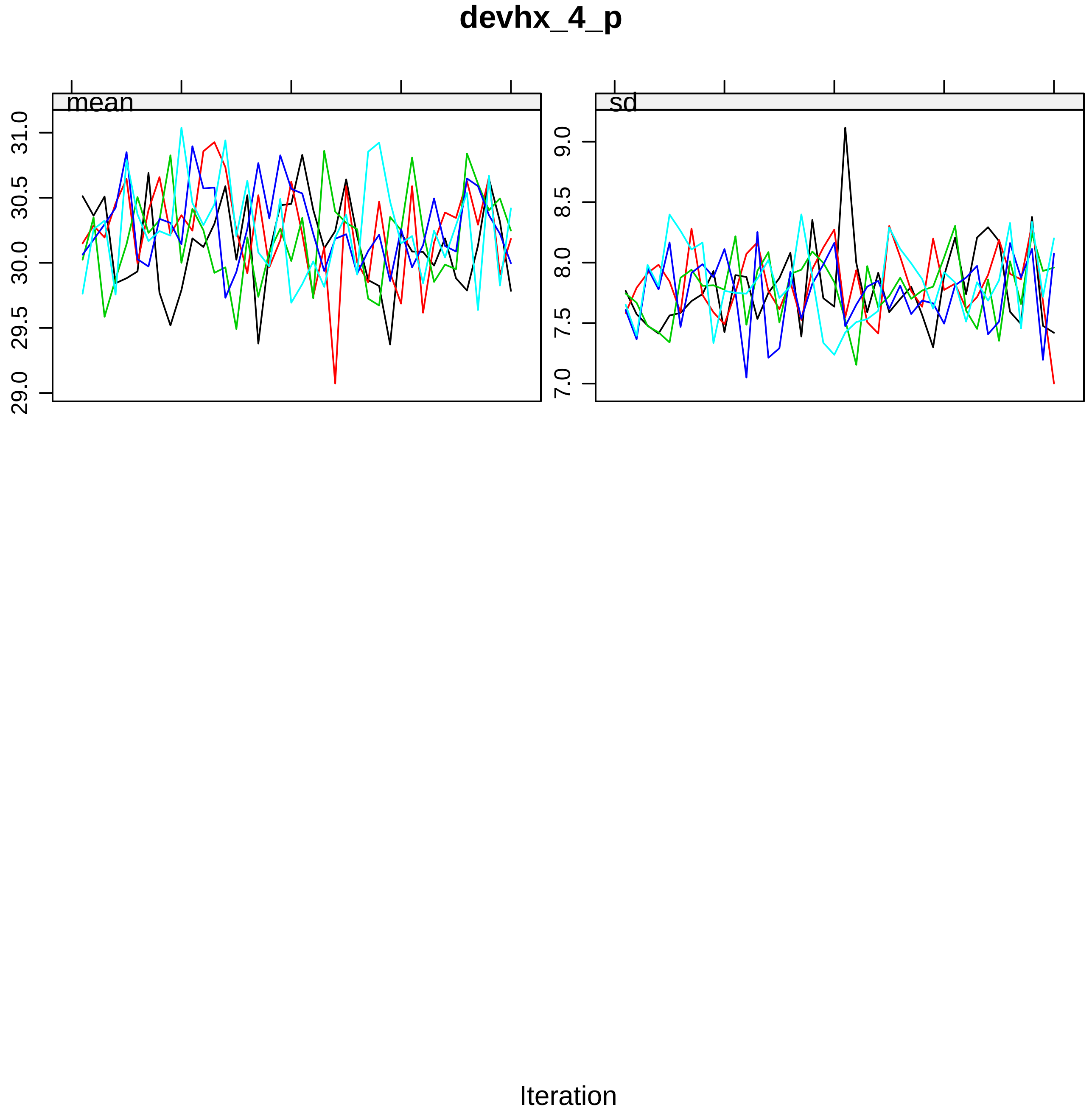
devhx_4_p
mean
sd
31.0
30.5
30.0
29.5
29.0
9.0
8.5
8.0
7.5
7.0
Iteration

devhx_5_p

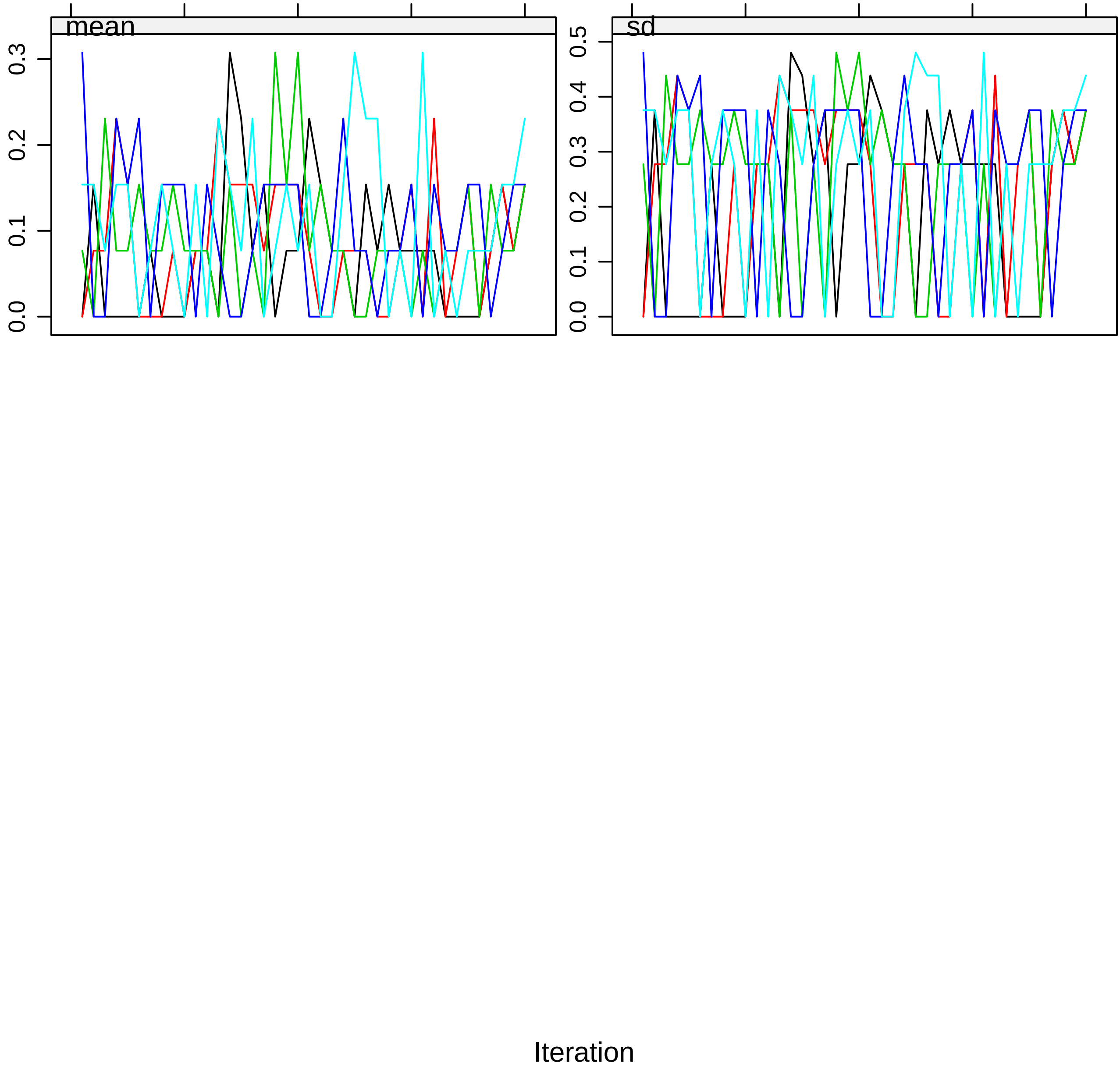

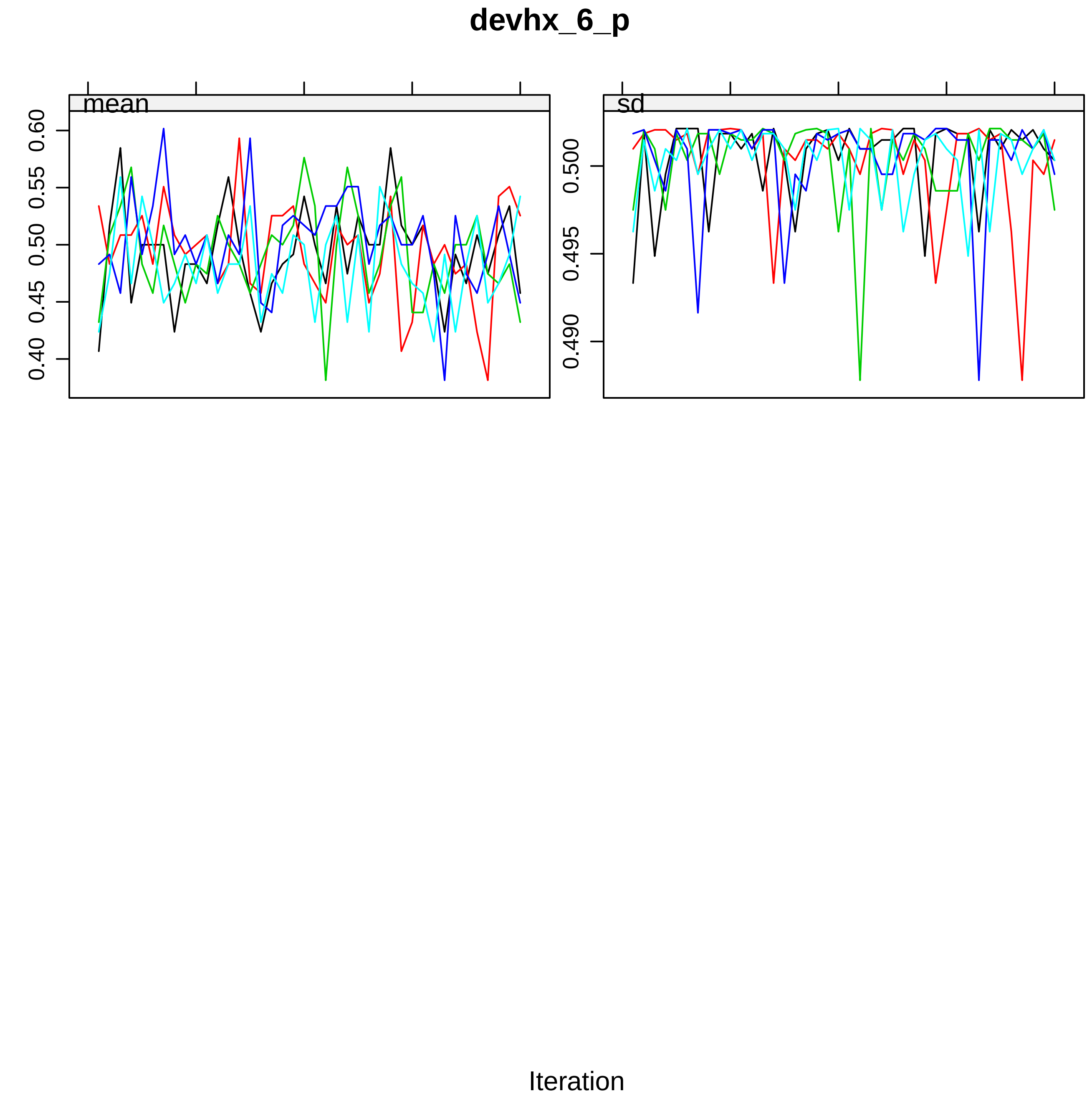
devhx_6_p
mean
sd
0.40
0.45
0.50
0.55
0.60
0.490
0.495
0.500
Iteration

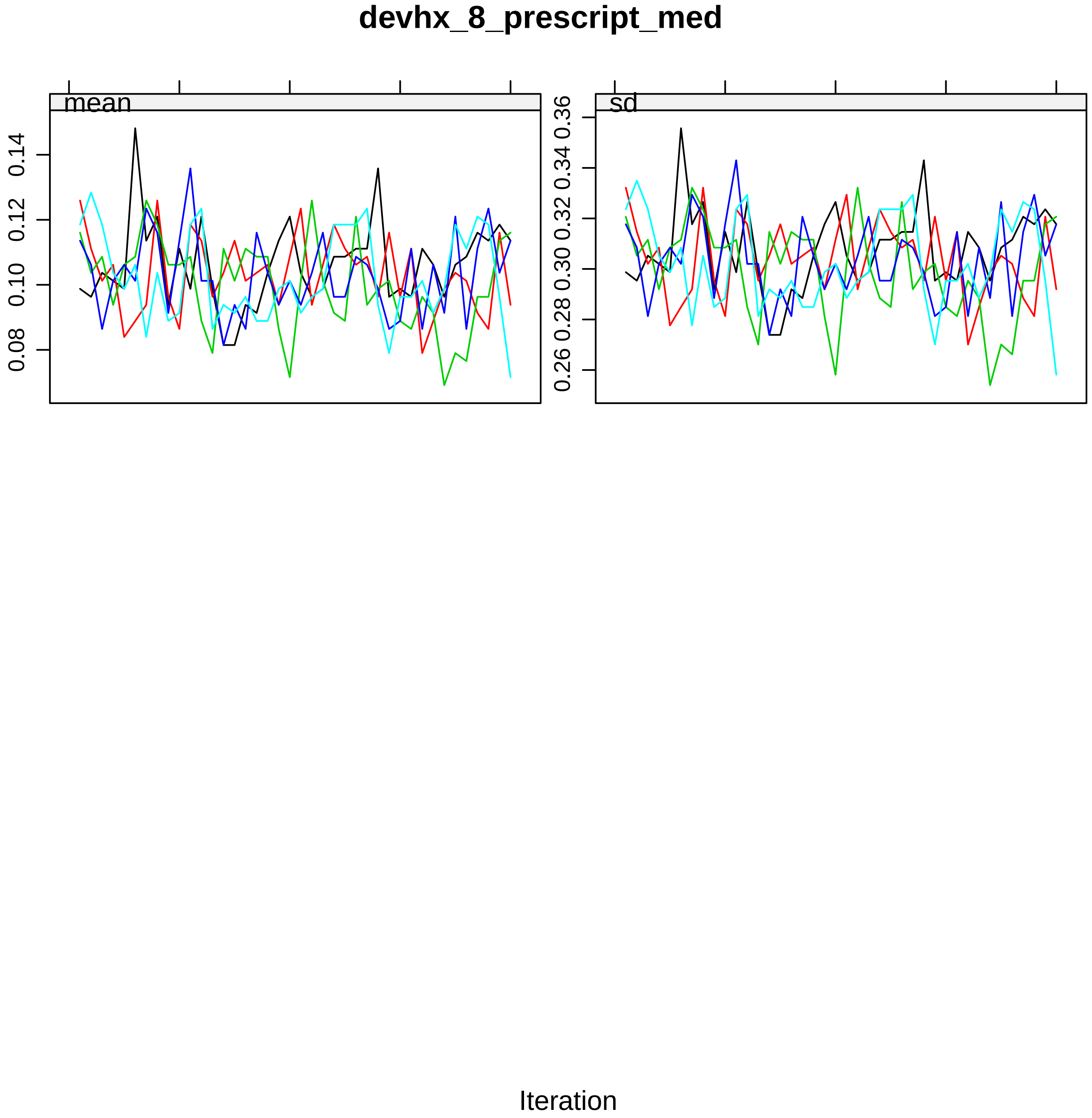
devhx_8_prescript_med
mean
sd
0.08
0.10
0.12
0.14
0.26
0.28
0.30
0.32
0.34
0.36
Iteration

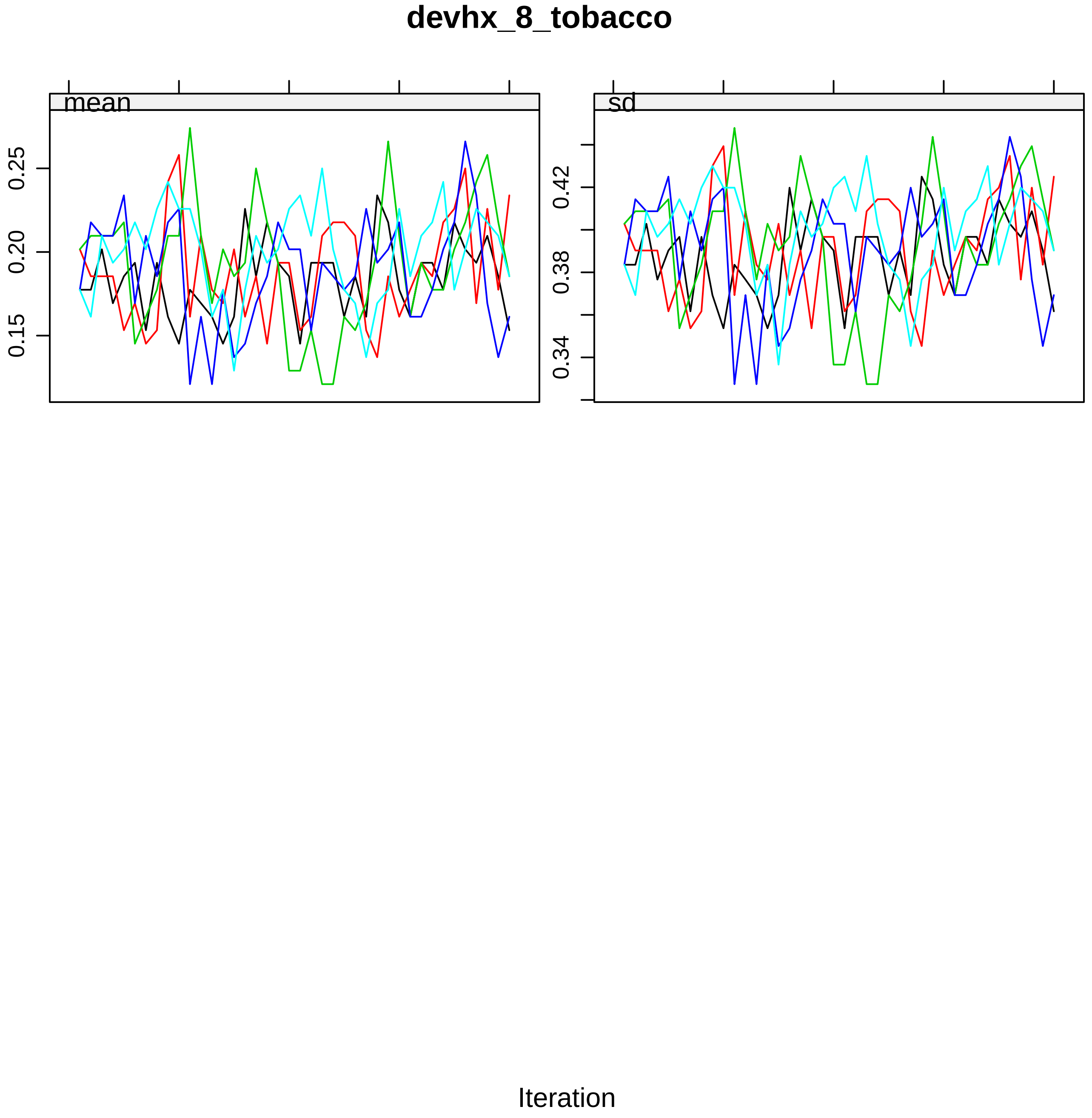
devhx_8_tobacco
mean
sd
0.15
0.20
0.25
0.34
0.38
0.42
Iteration

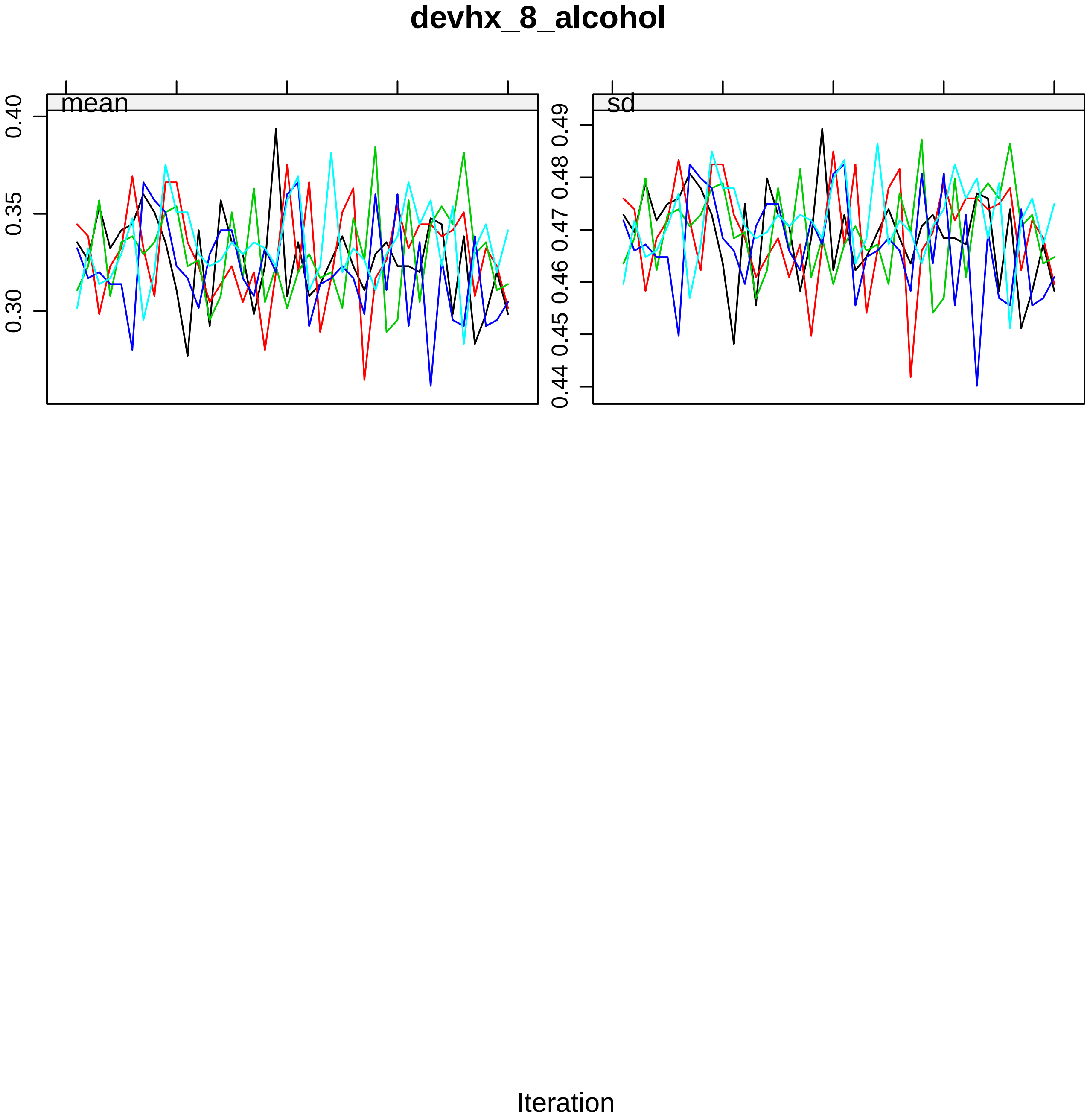
devhx_8_alcohol
mean
sd
0.40
0.35
0.30
0.49
0.48
0.47
0.46
0.45
0.44
Iteration

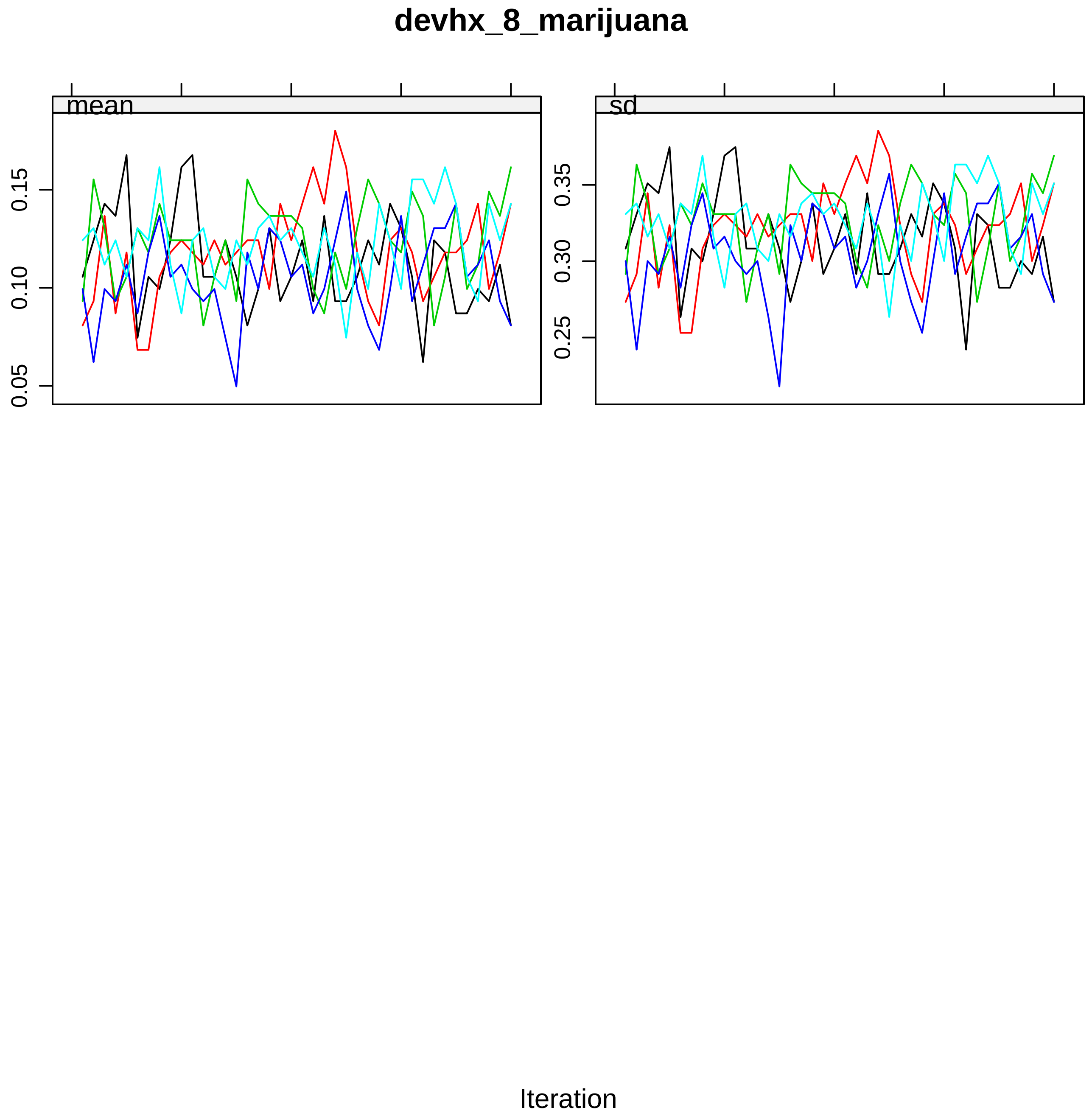

devhx_8_marijuana
mean
sd
0.05
0.10
0.15
0.25
0.30
0.35
Iteration

devhx_9_prescript_med

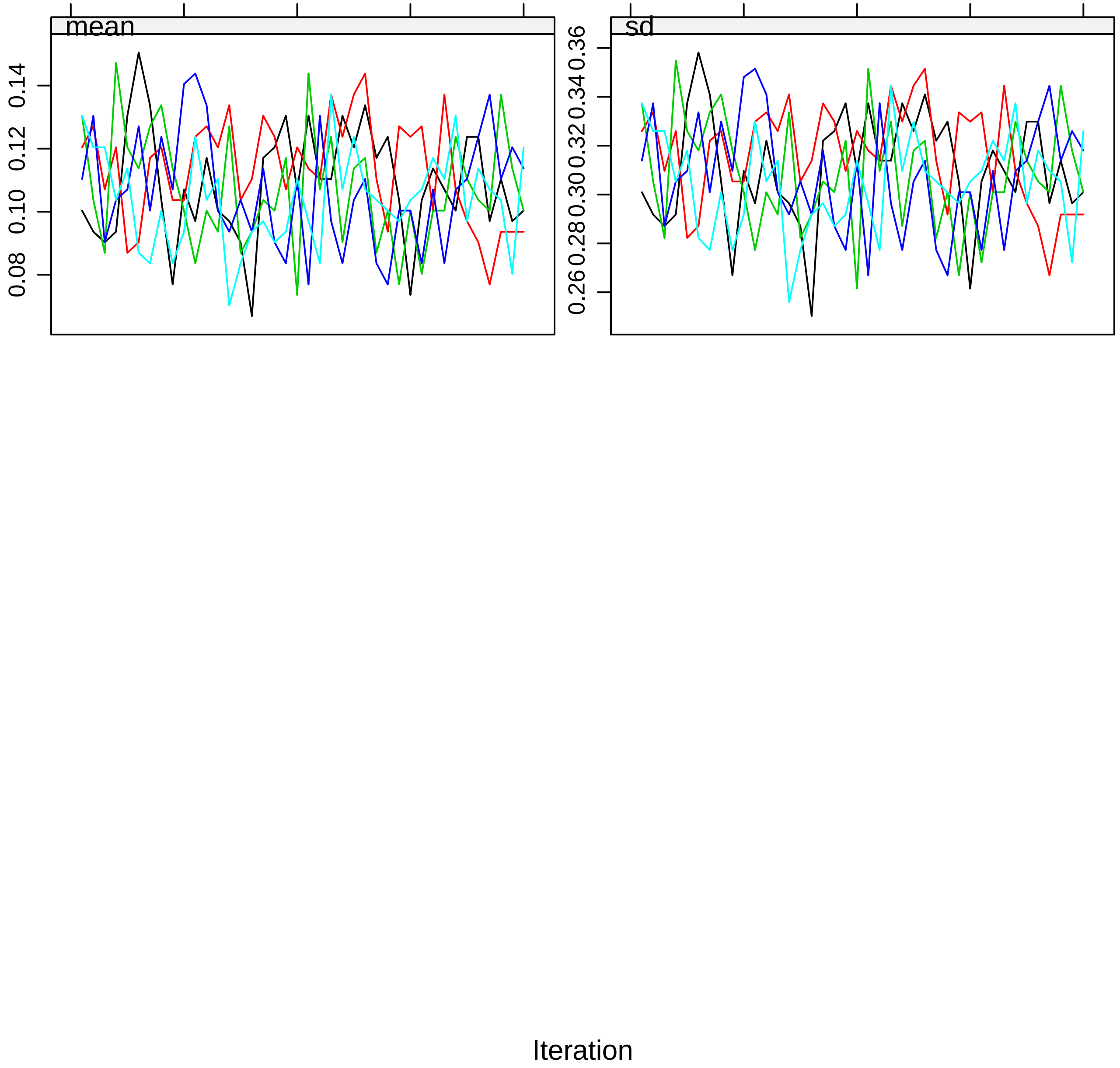

devhx_9_tobacco

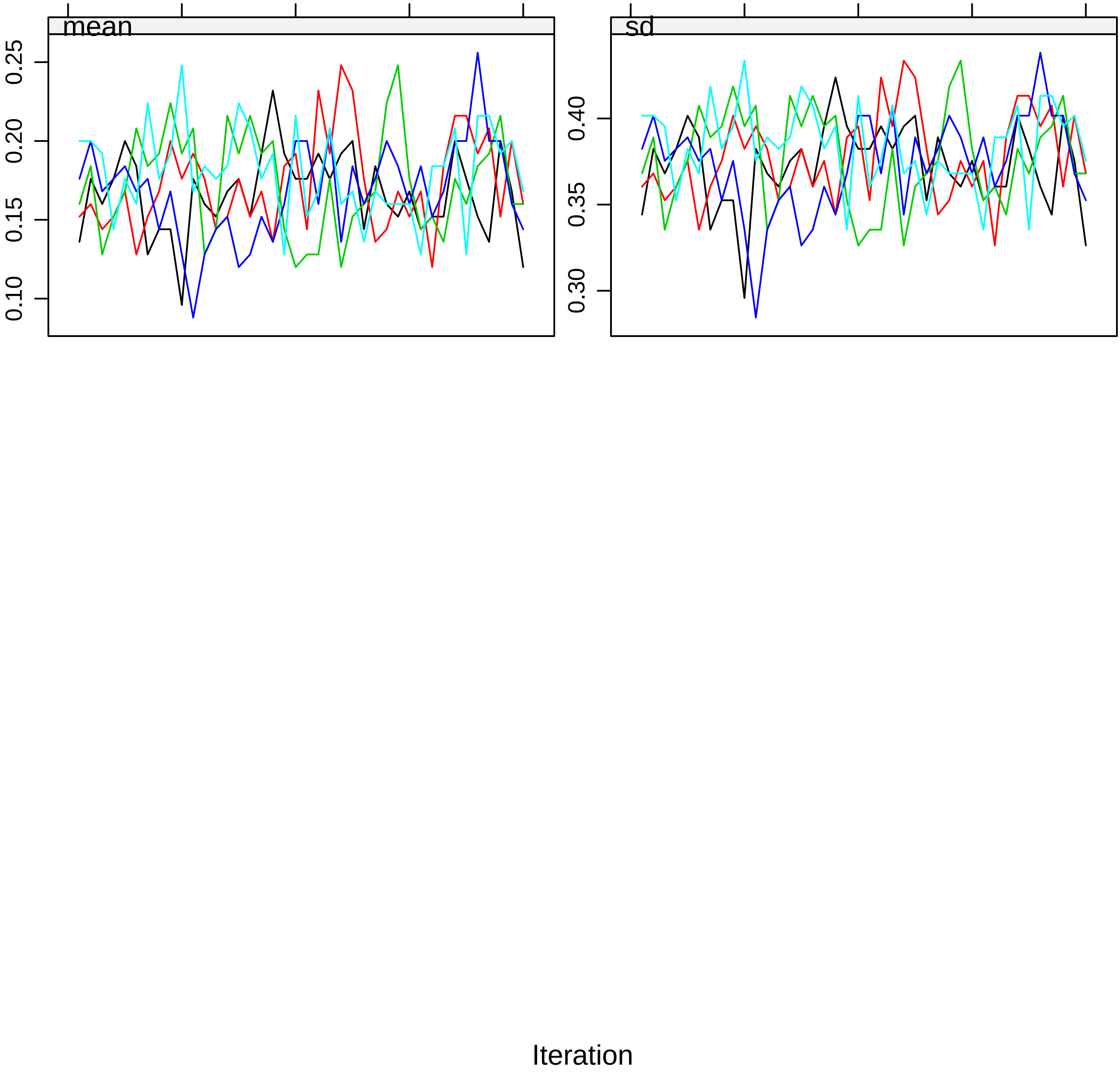

devhx_9_alcohol

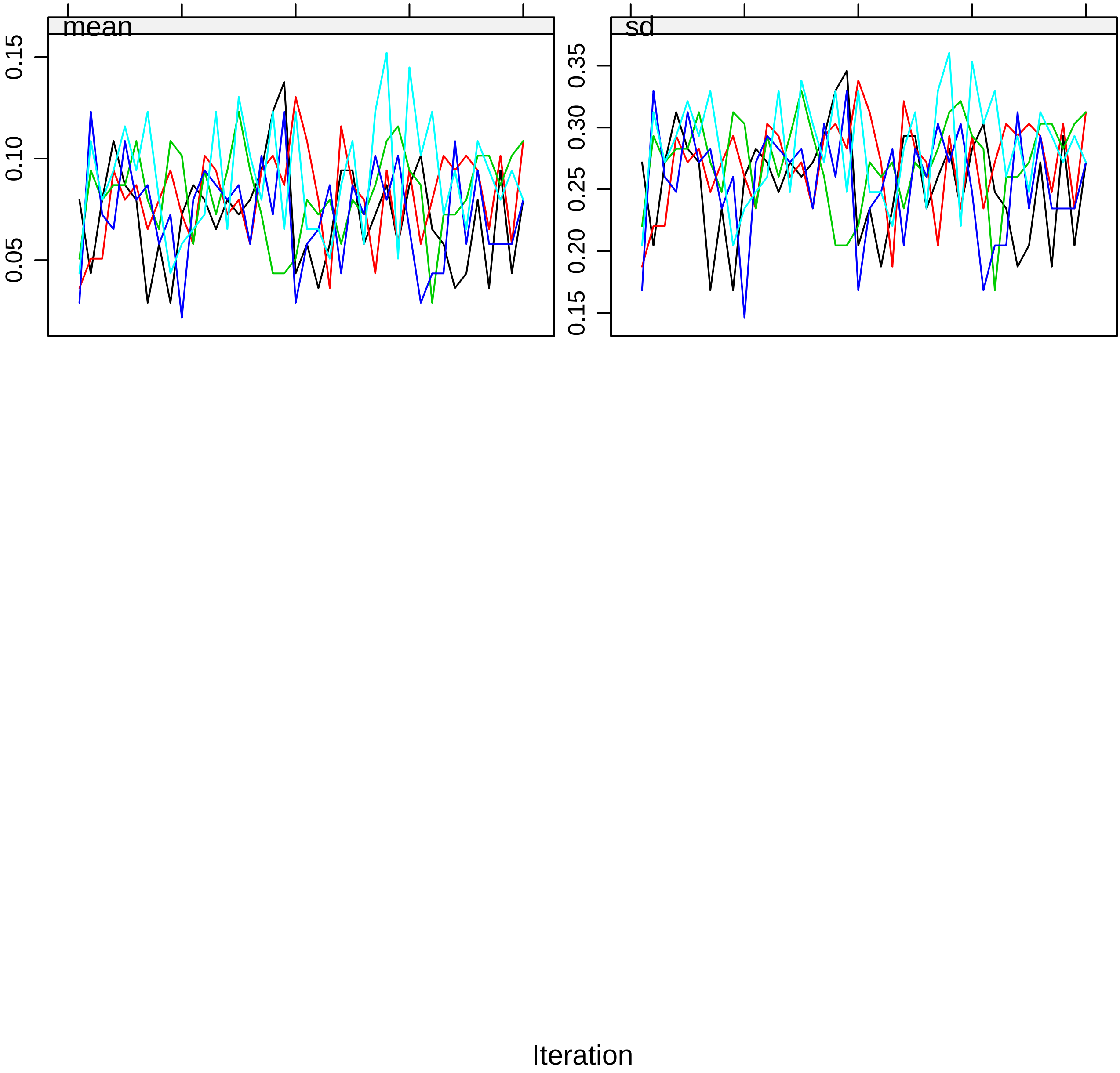

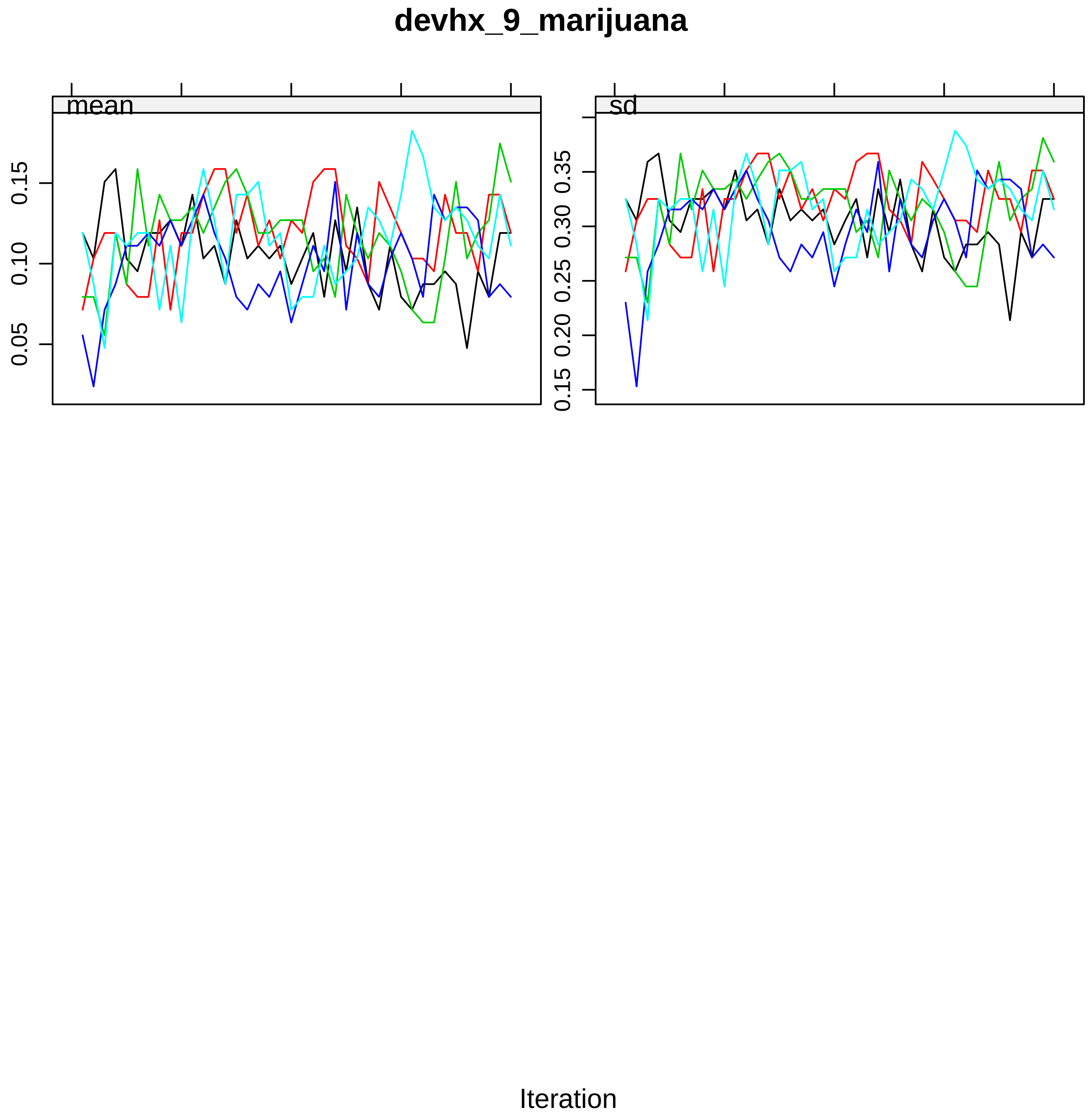
devhx_9_marijuana
mean
sd
0.05
0.10
0.15
0.15
0.20
0.25
0.30
0.35
Iteration

devhx_10

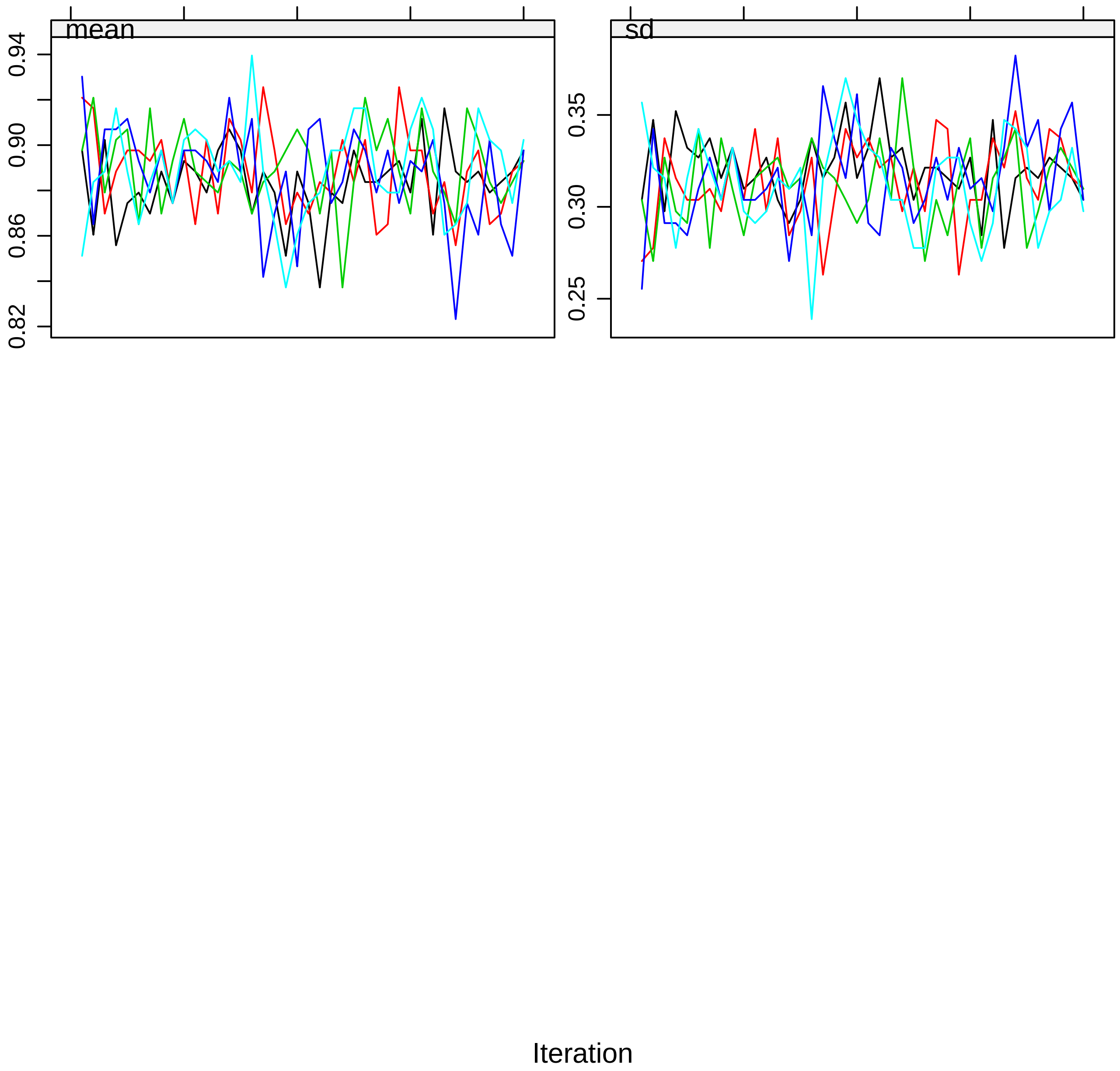

devhx_caffeine_11

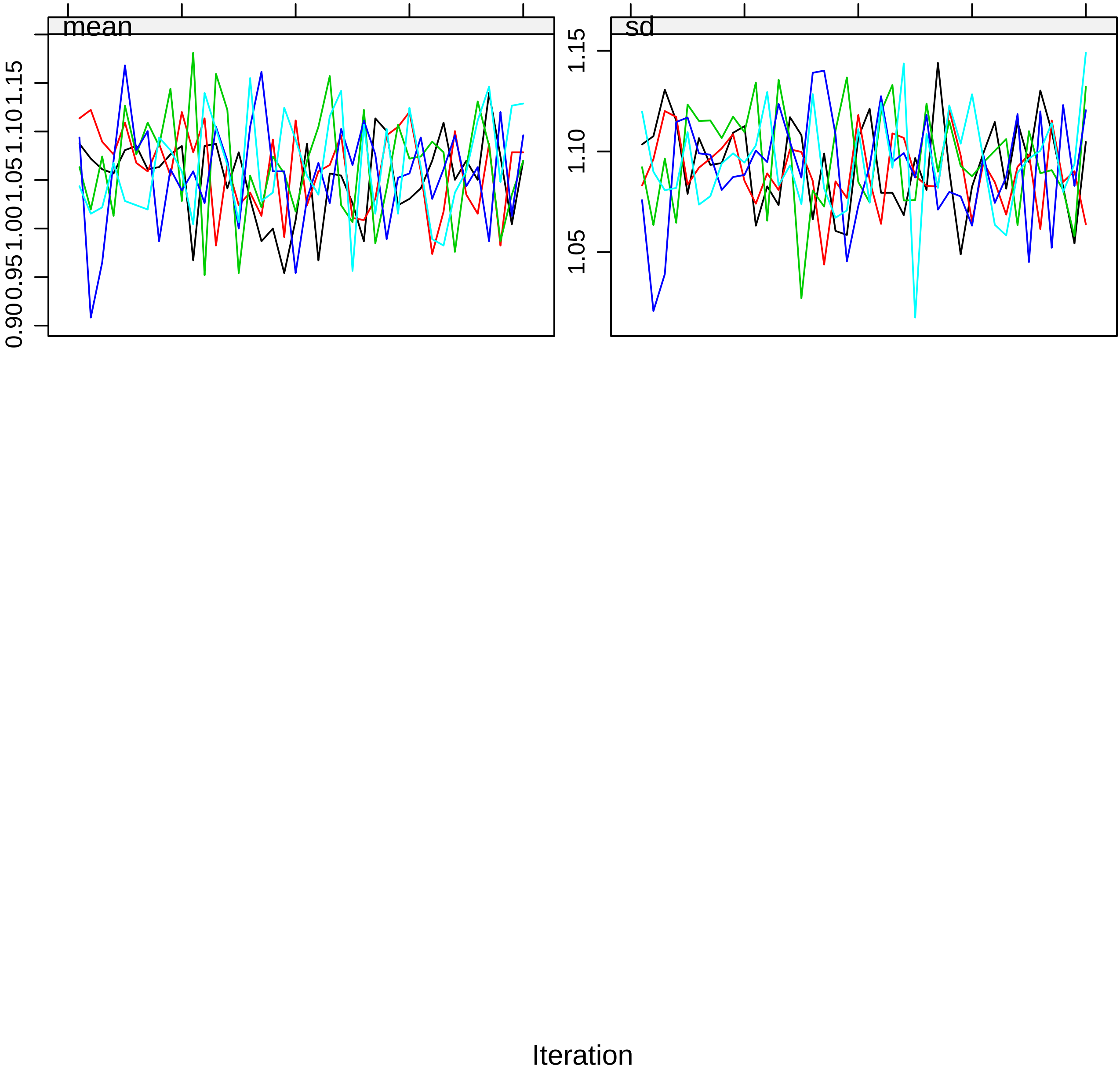

devhx_10a3_p

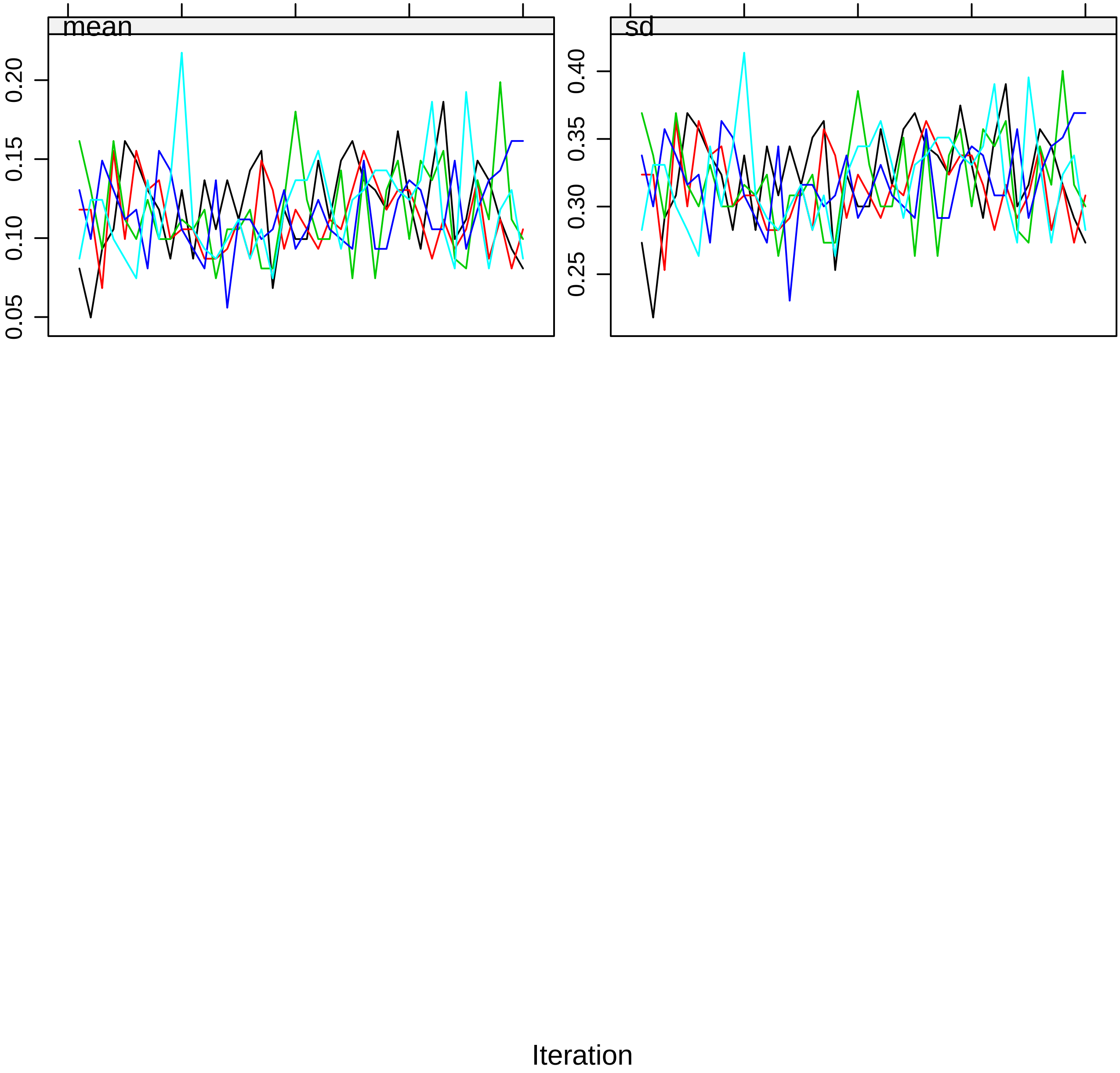

devhx_10b3_p

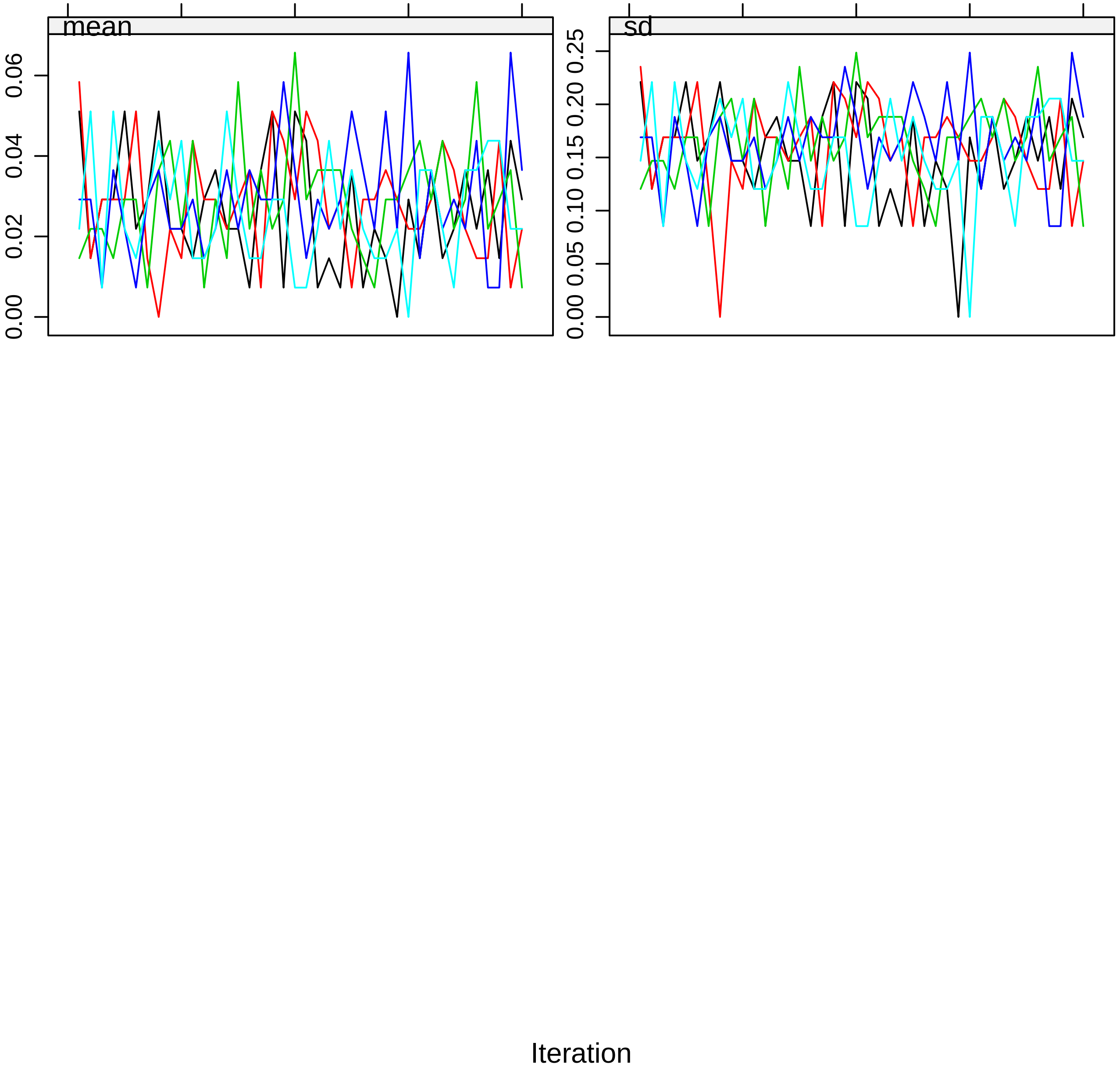

devhx_10c3_p

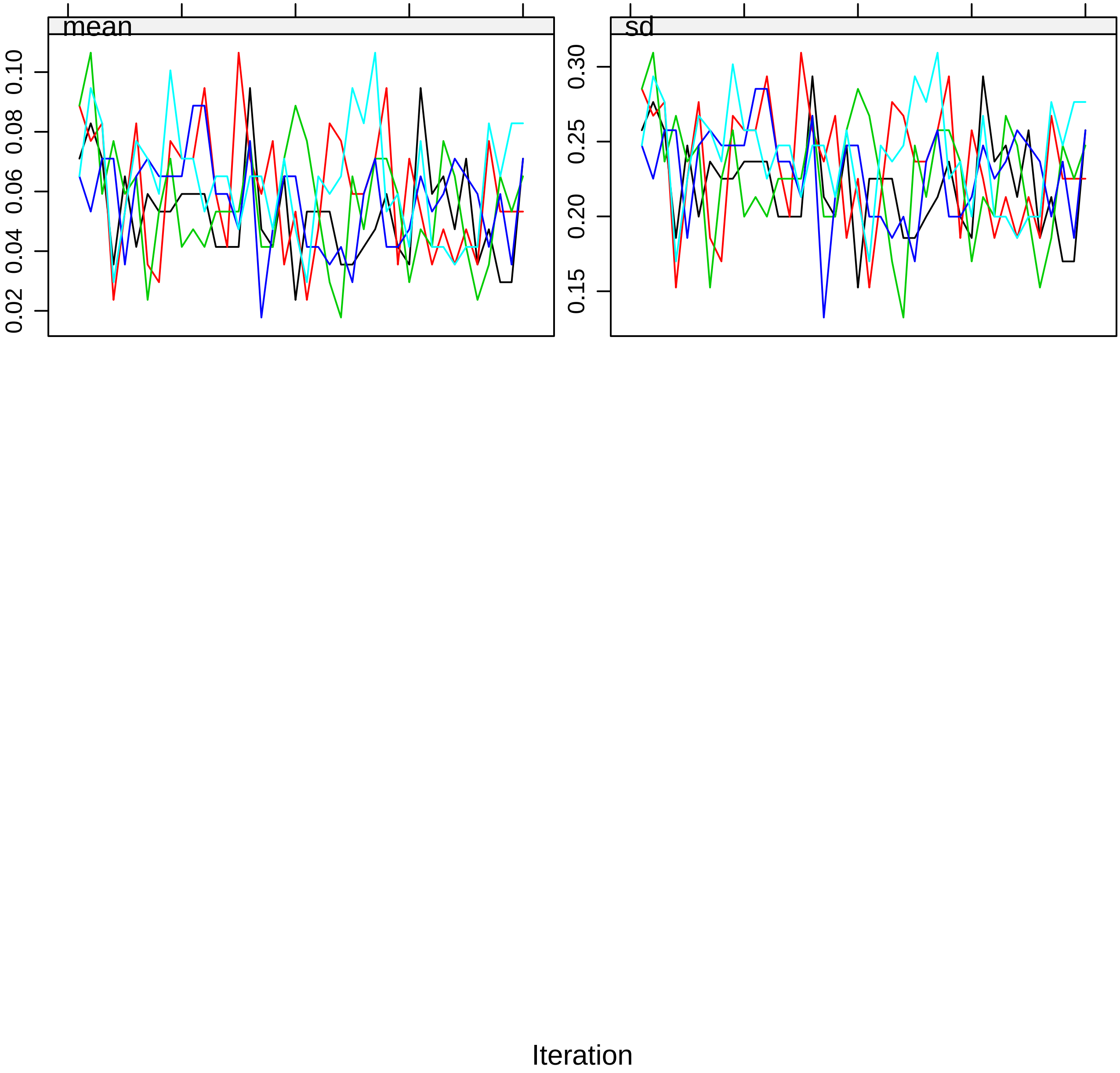

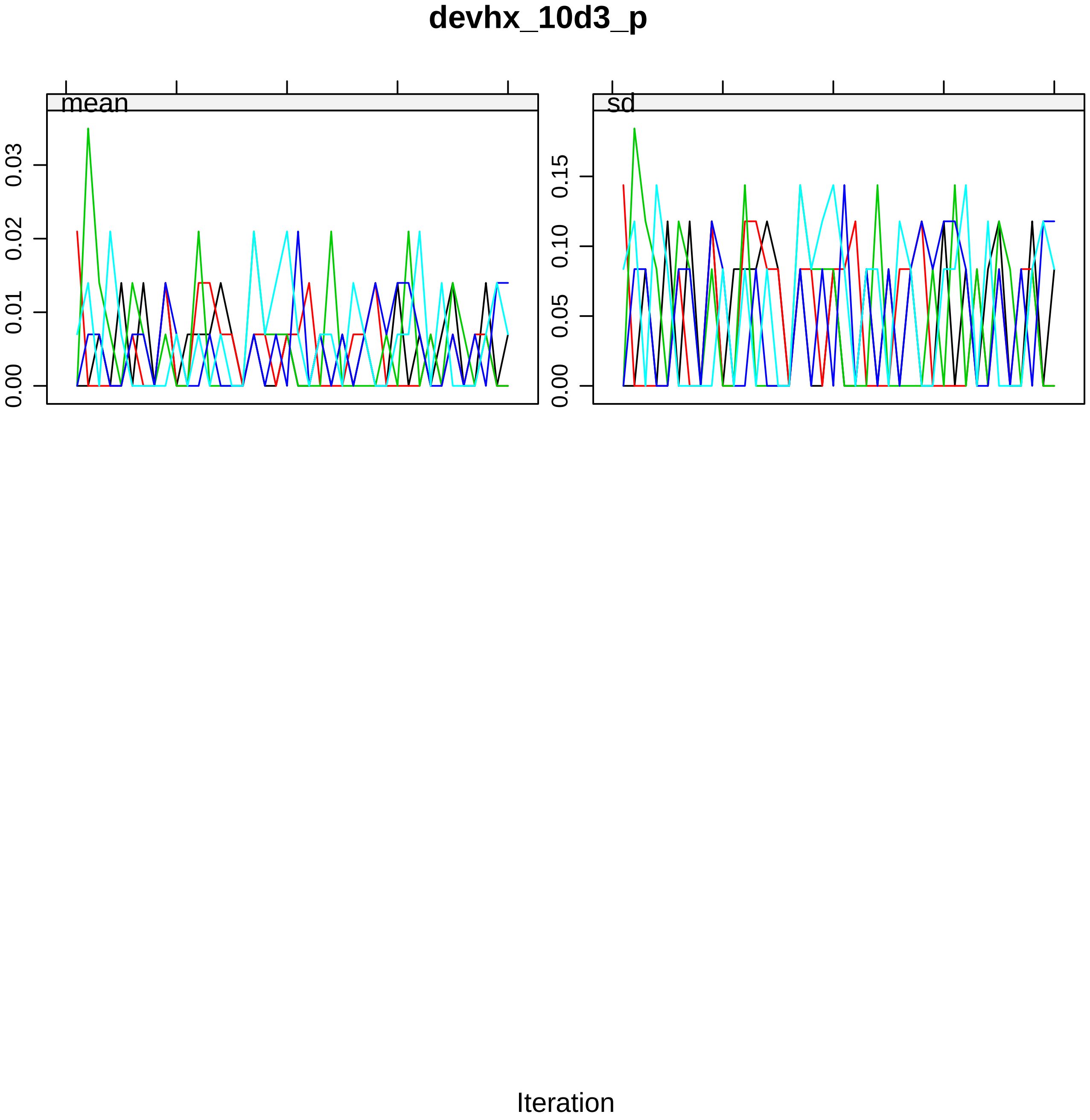

devhx_10d3_p
mean
sd
0.00
0.01
0.02
0.03
0.00
0.05
0.10
0.15
Iteration

devhx_10e3_p

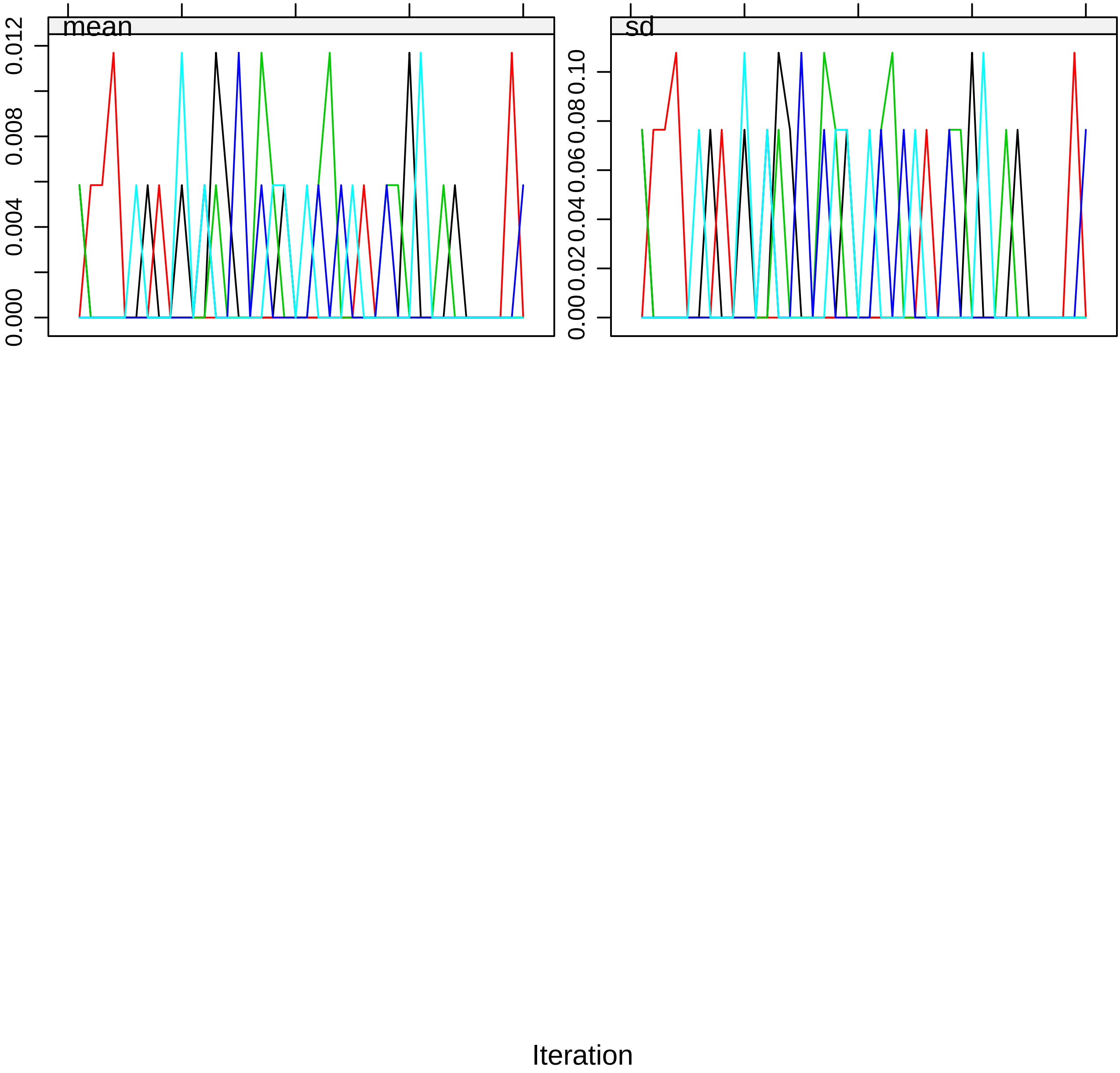

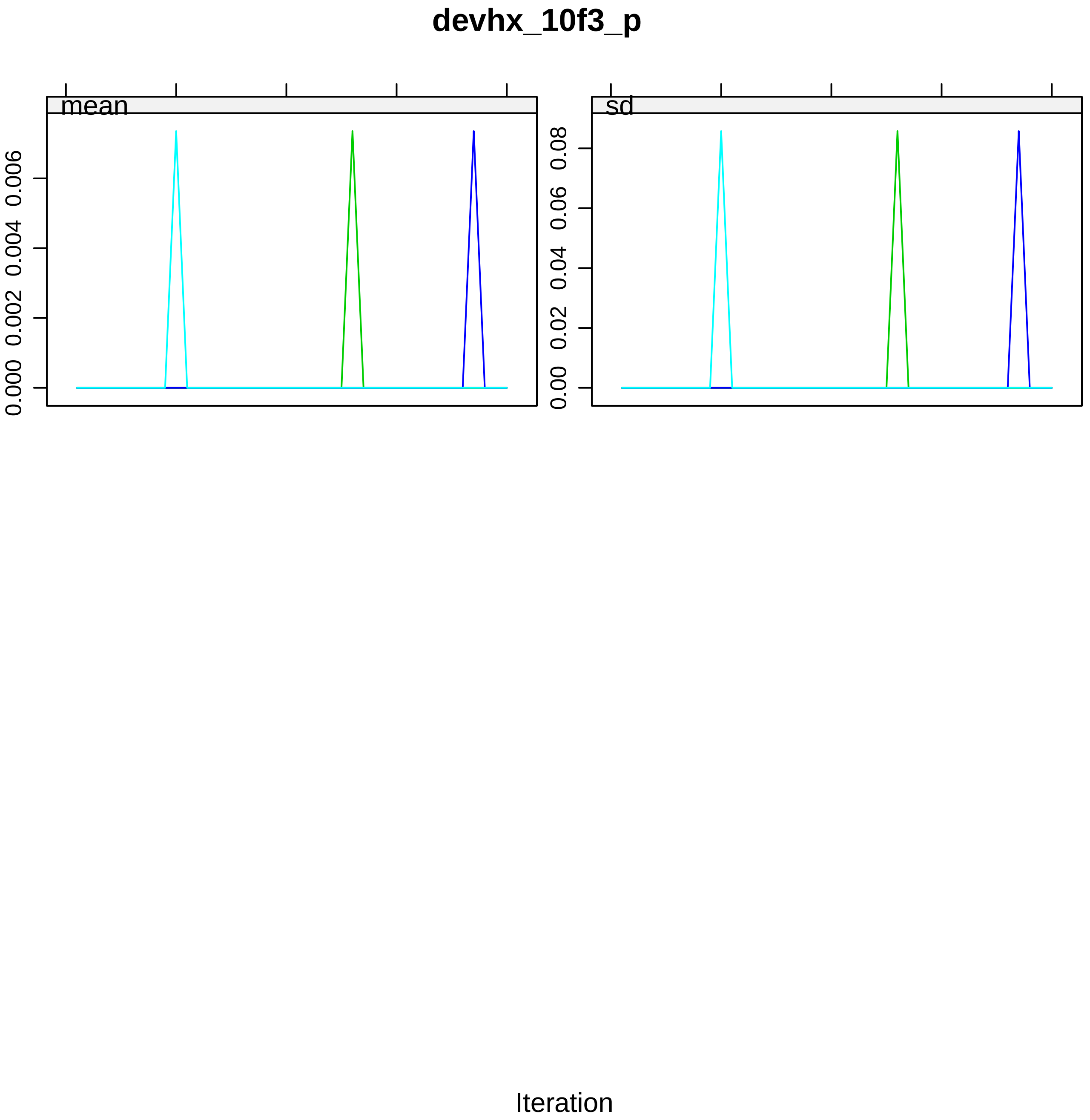
devhx_10f3_p
mean
sd
0.000
0.002
0.004
0.006
0.00
0.02
0.04
0.06
0.08
Iteration

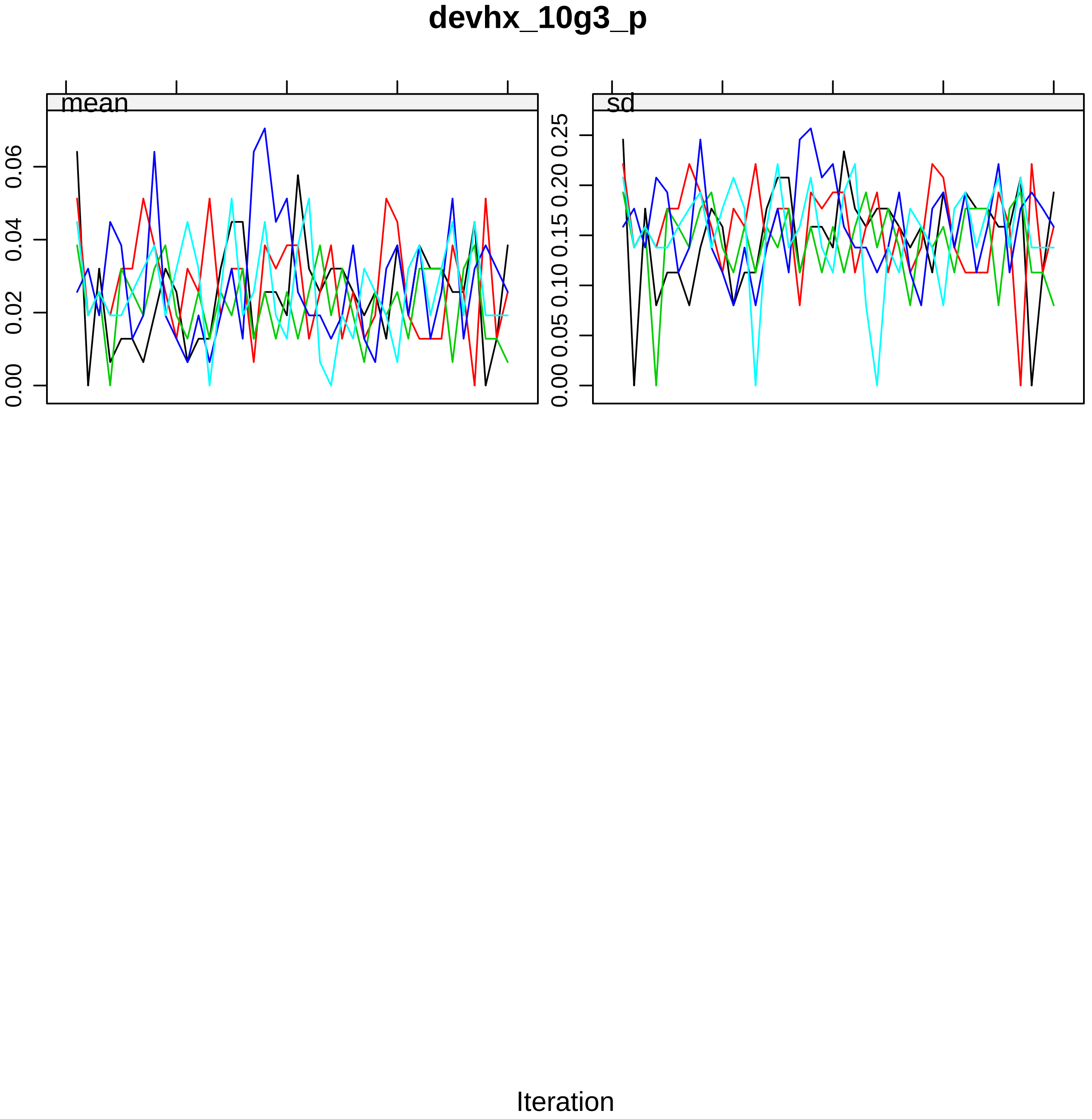
devhx_10g3_p
mean
sd
0.00
0.02
0.04
0.06
0.00
0.05
0.10
0.15
0.20
0.25
Iteration

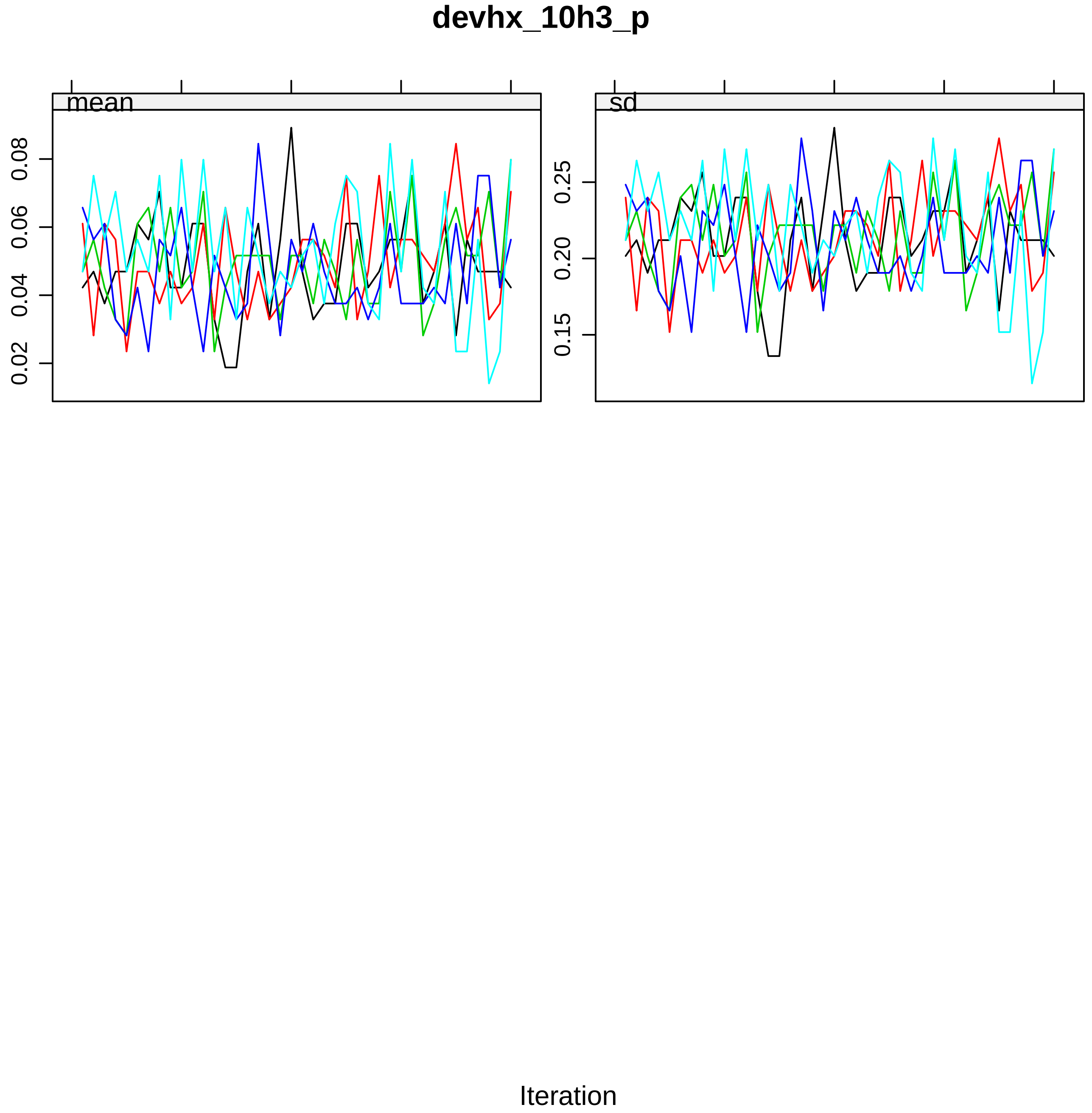
devhx_10h3_p
mean
sd
0.02
0.04
0.06
0.08
0.15
0.20
0.25
Iteration

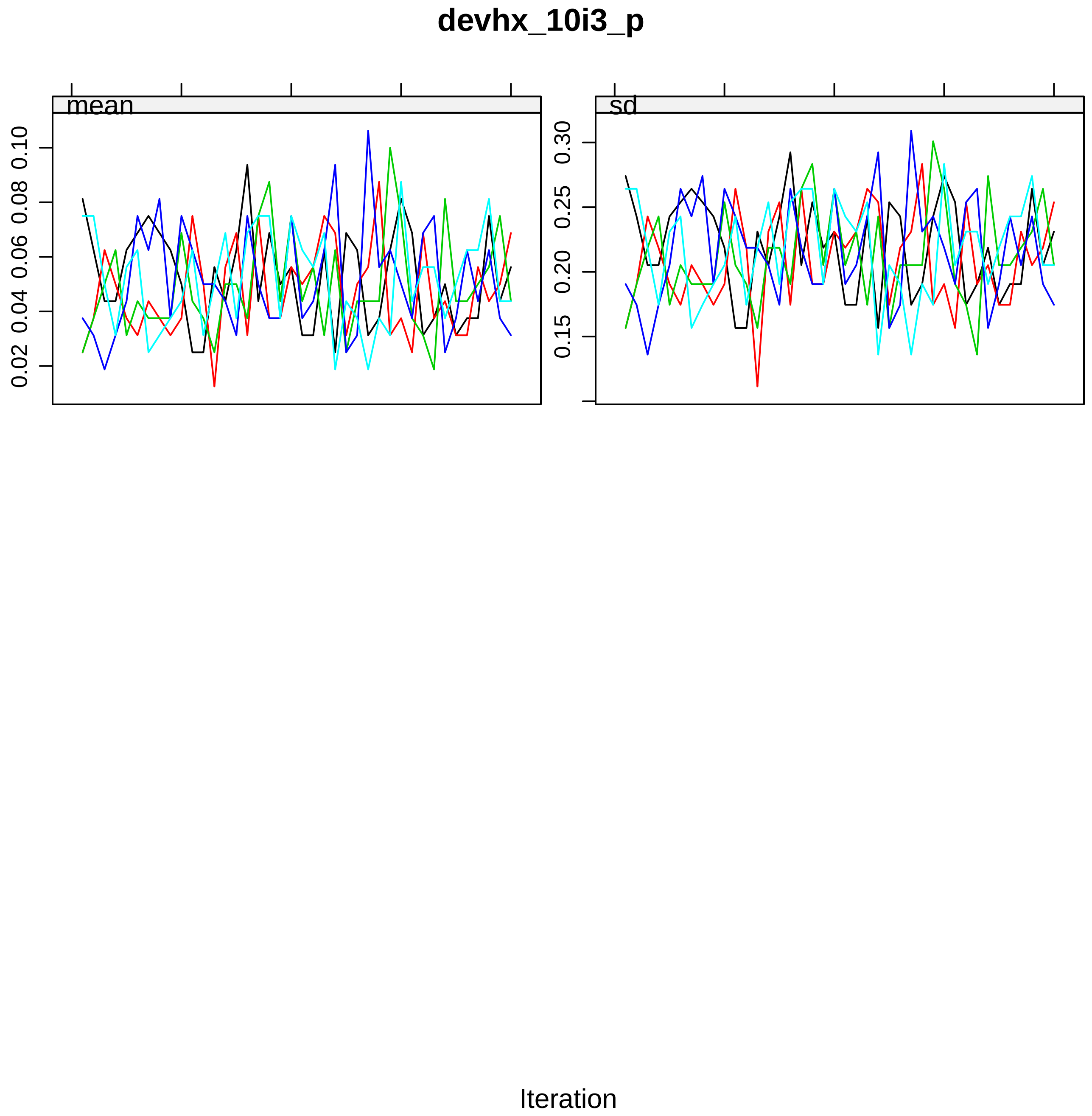
devhx_10i3_p
mean
sd
0.02
0.04
0.06
0.08
0.10
0.15
0.20
0.25
0.30
Iteration

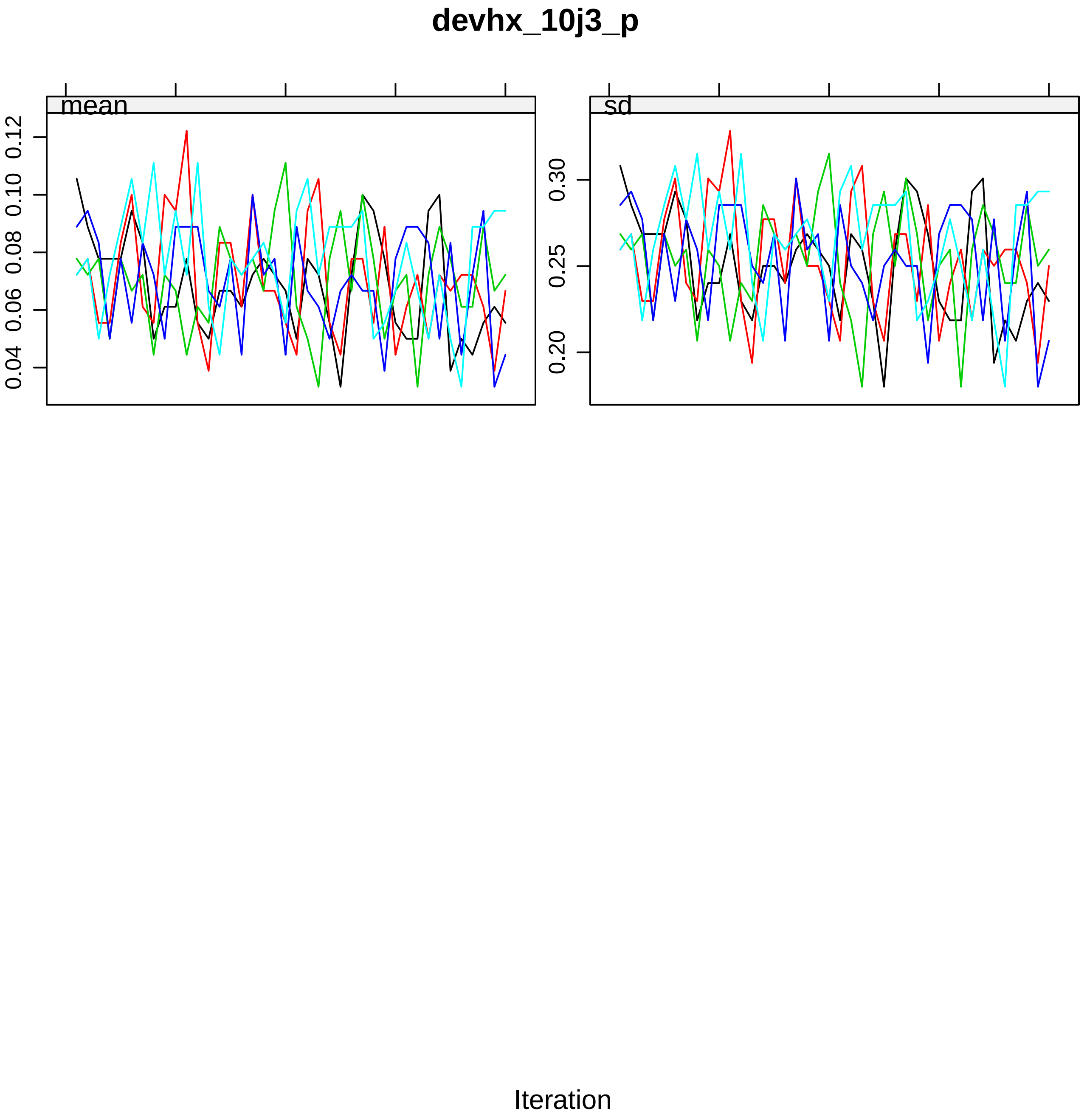
devhx_10j3_p
mean
sd
0.04
0.06
0.08
0.10
0.12
0.20
0.25
0.30
Iteration

devhx_10k3_p

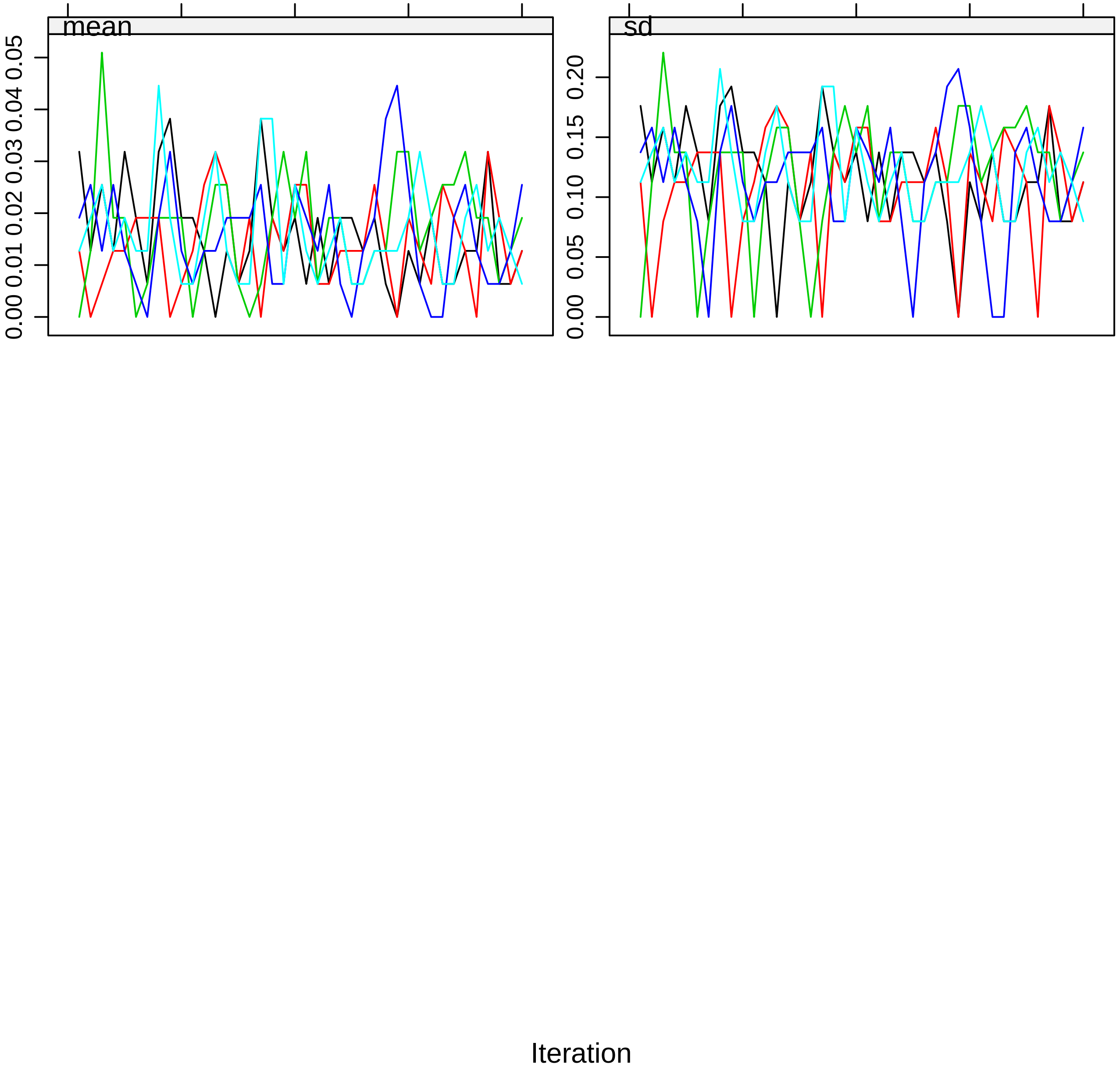

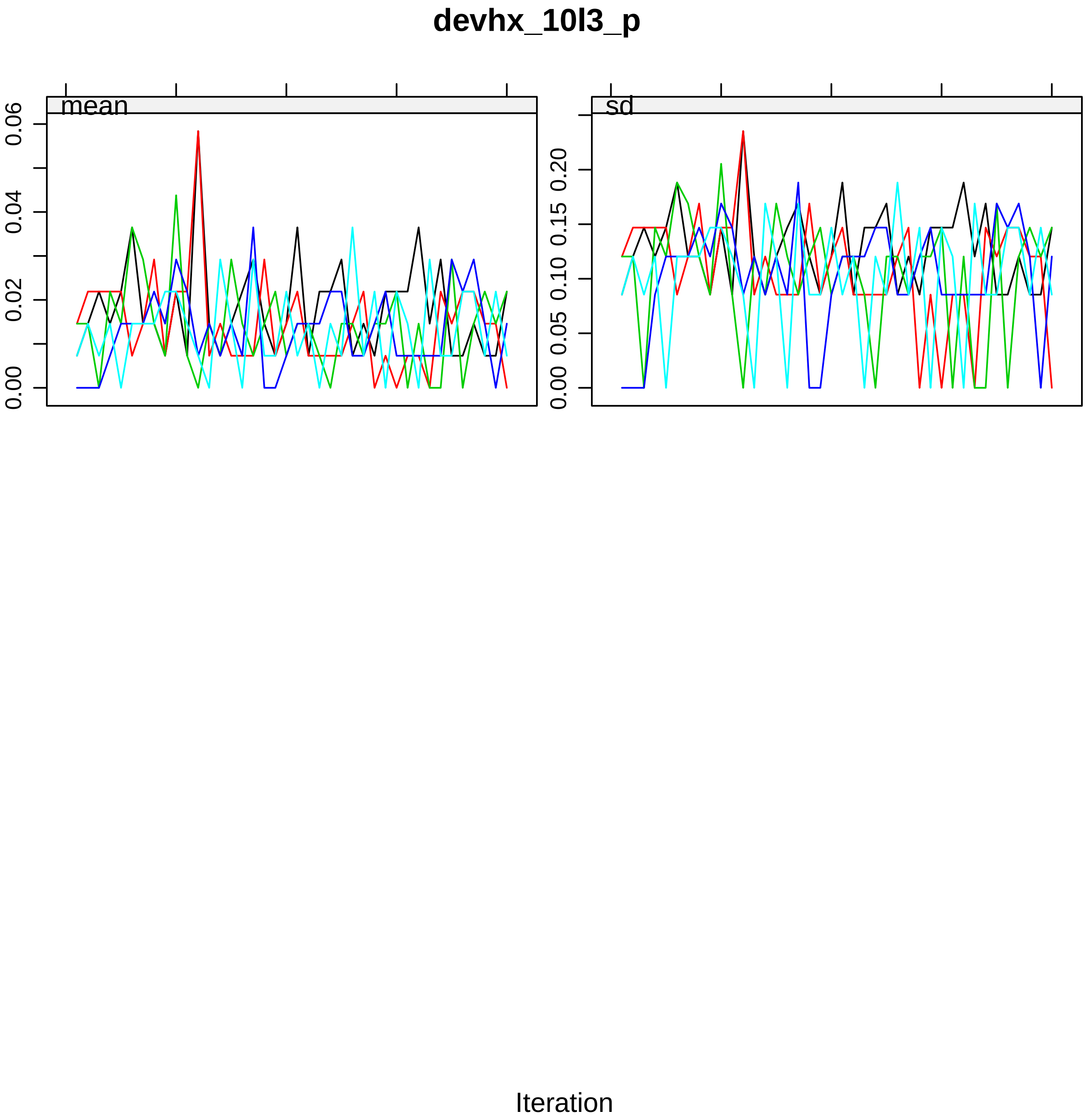

devhx_10l3_p
mean
sd
0.00
0.02
0.04
0.06
0.00
0.05
0.10
0.15
0.20
Iteration

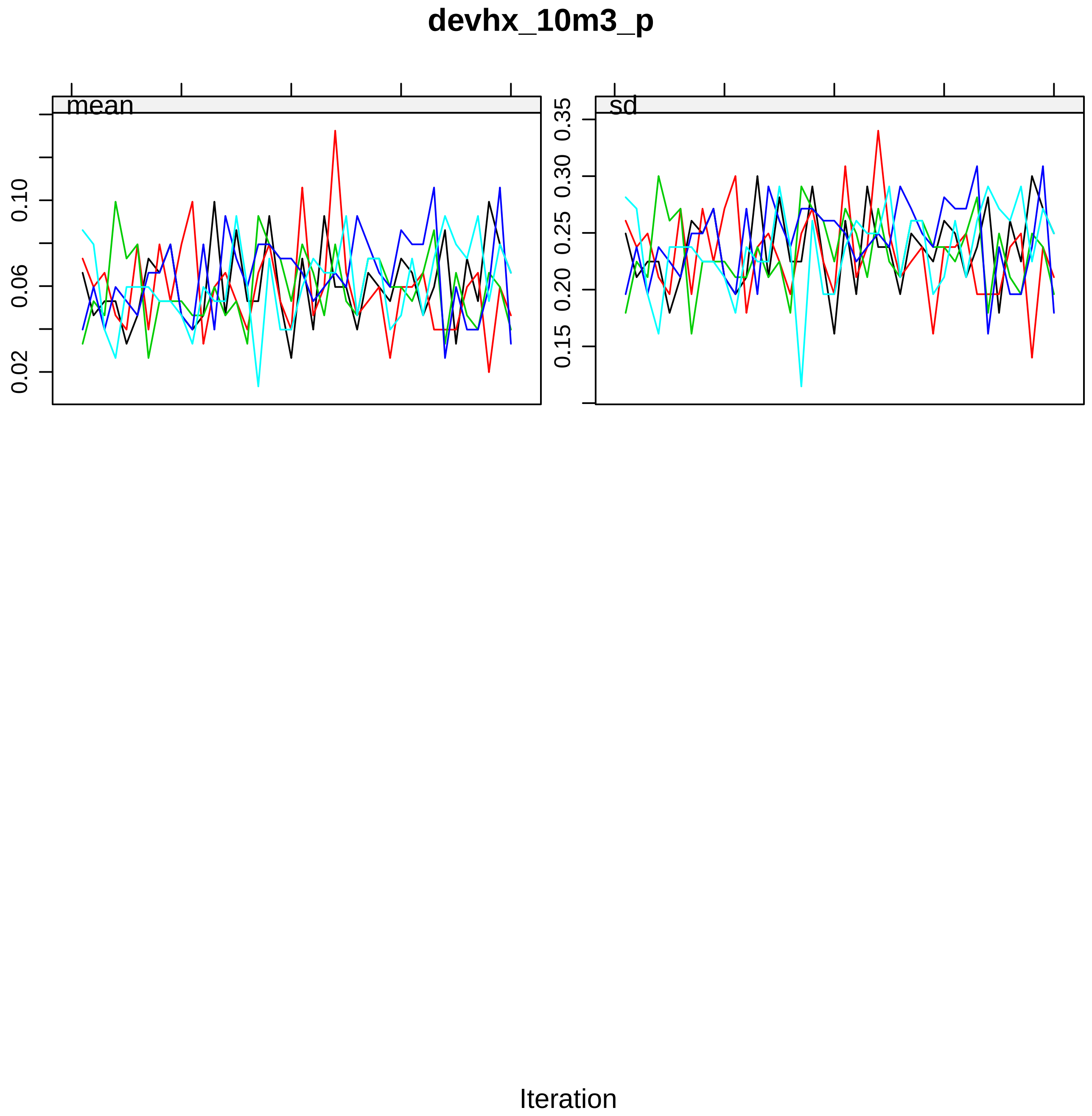
devhx_10m3_p
mean
sd
0.02
0.06
0.10
0.15
0.20
0.25
0.30
0.35
Iteration

devhx_12a_p

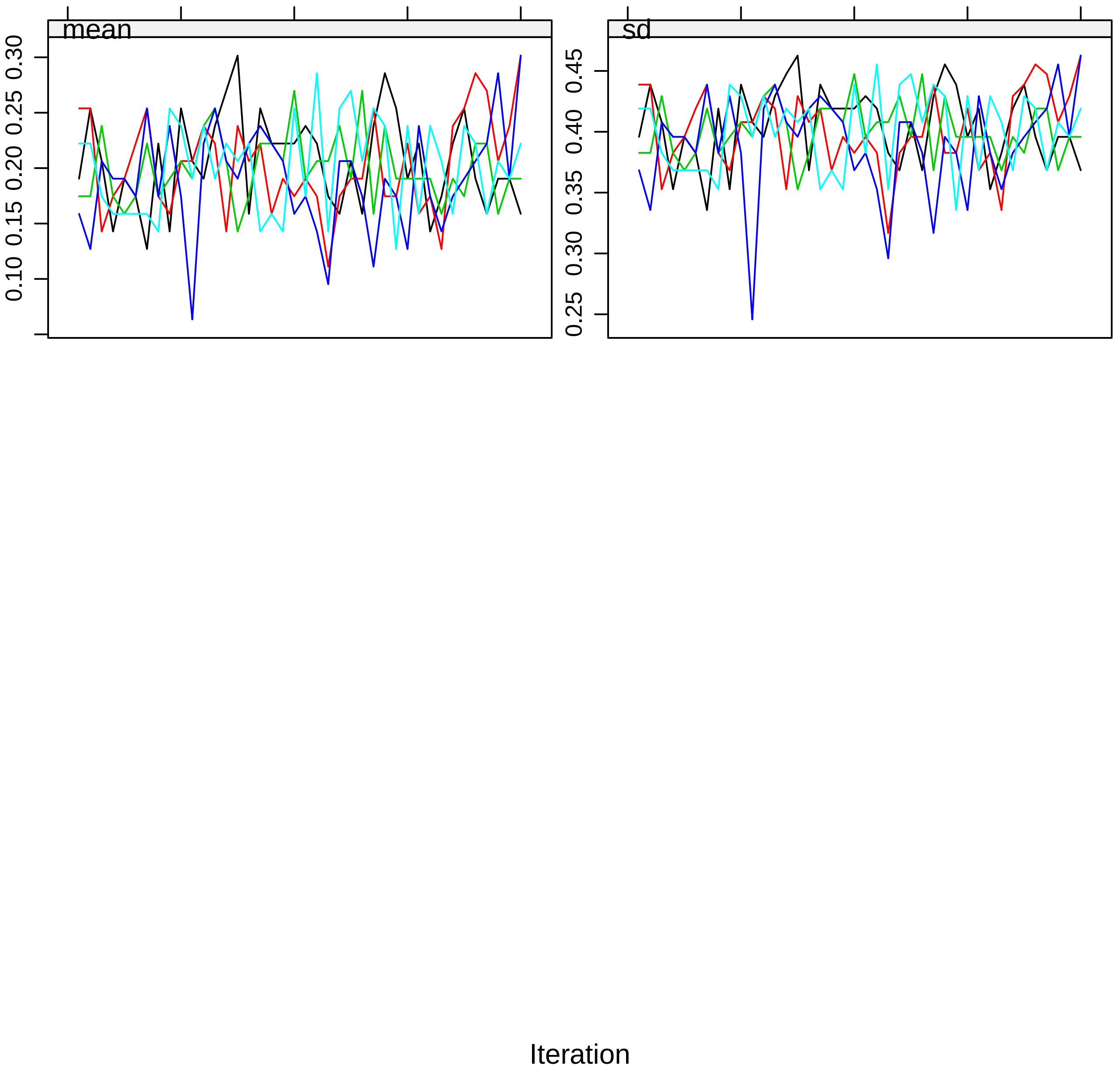

devhx_13_3_p

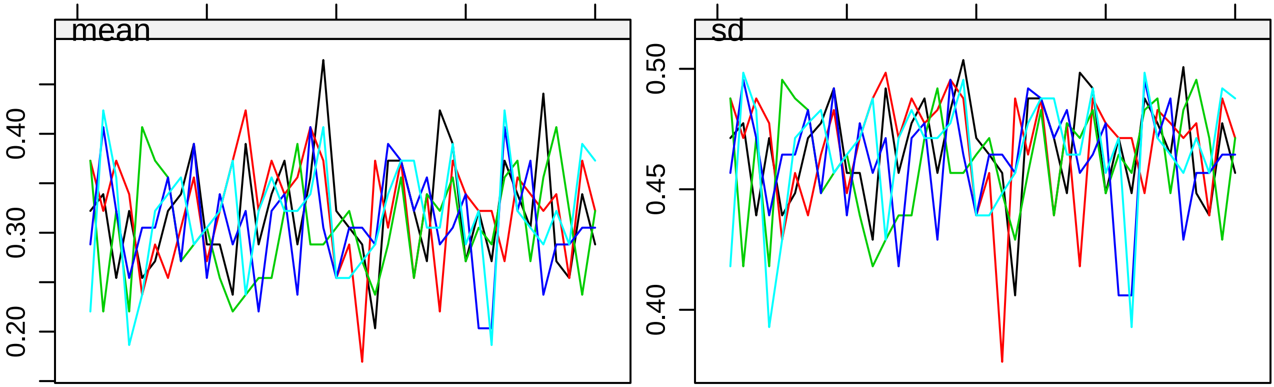

Iteration

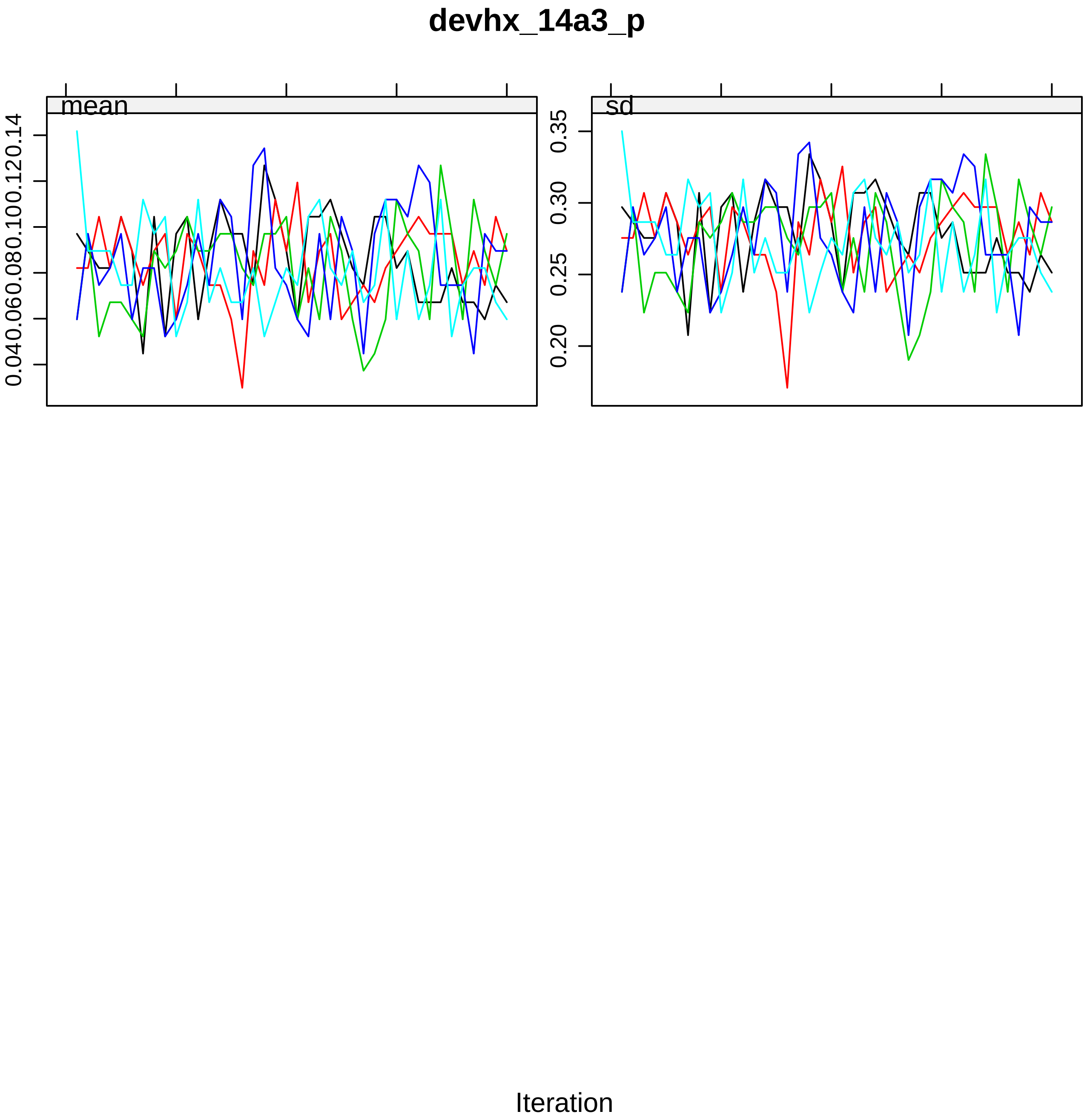
devhx_14a3_p
mean
sd
0.04
0.06
0.08
0.10
0.12
0.14
0.20
0.25
0.30
0.35
Iteration

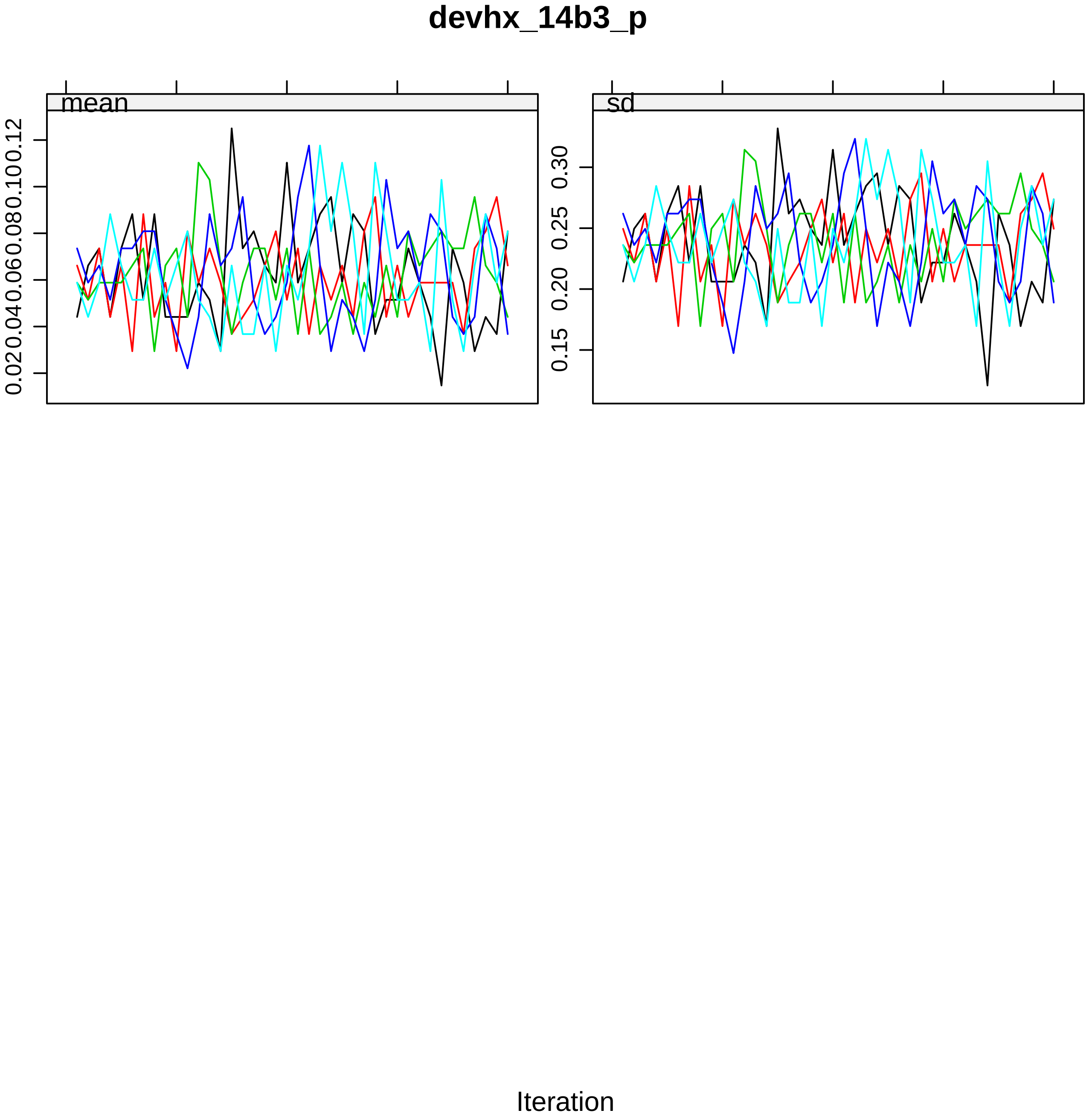
devhx_14b3_p
mean
sd
0.02
0.04
0.06
0.08
0.10
0.12
0.15
0.20
0.25
0.30
Iteration

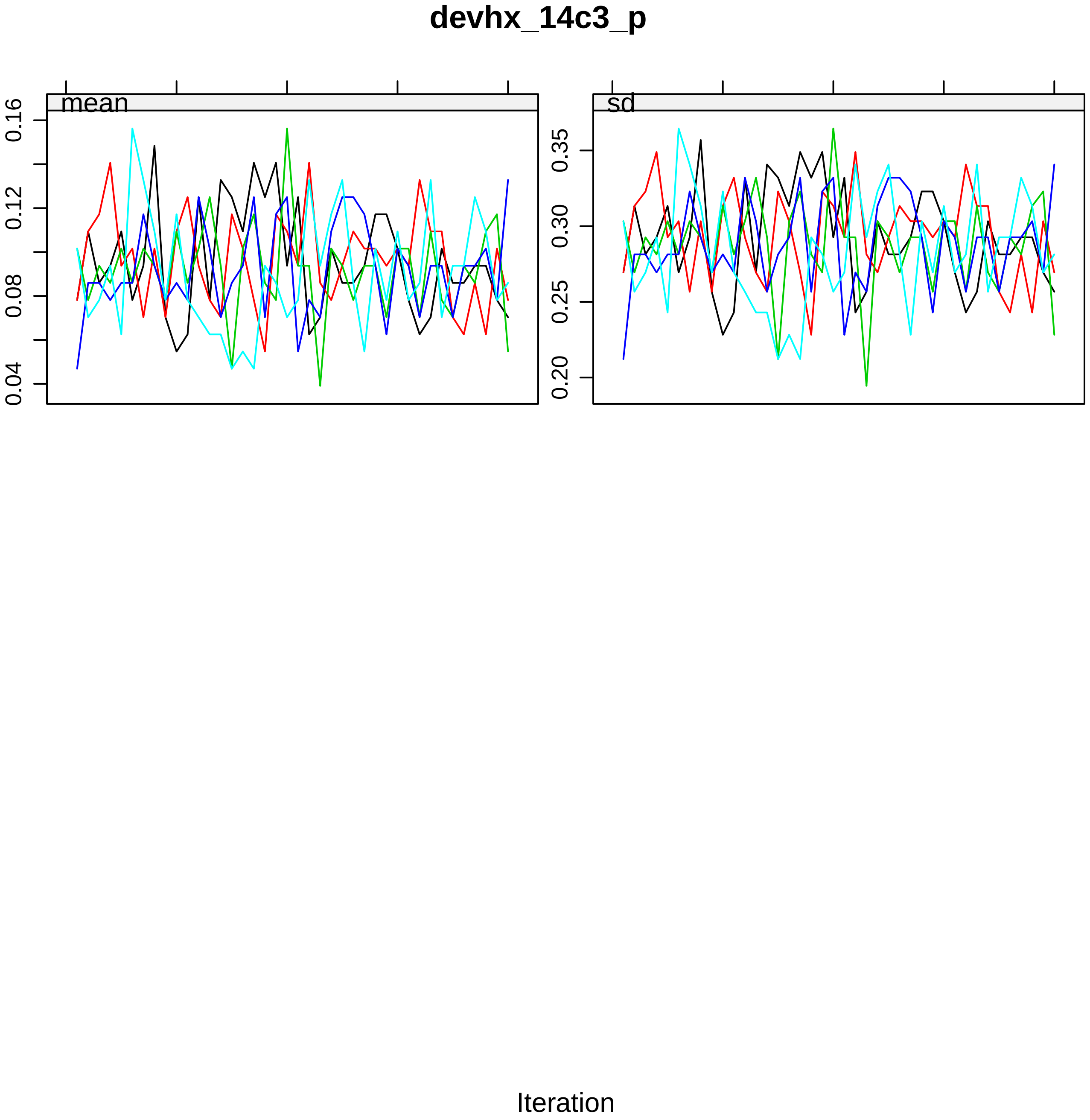
devhx_14c3_p
mean
sd
0.04
0.08
0.12
0.16
0.20
0.25
0.30
0.35
Iteration

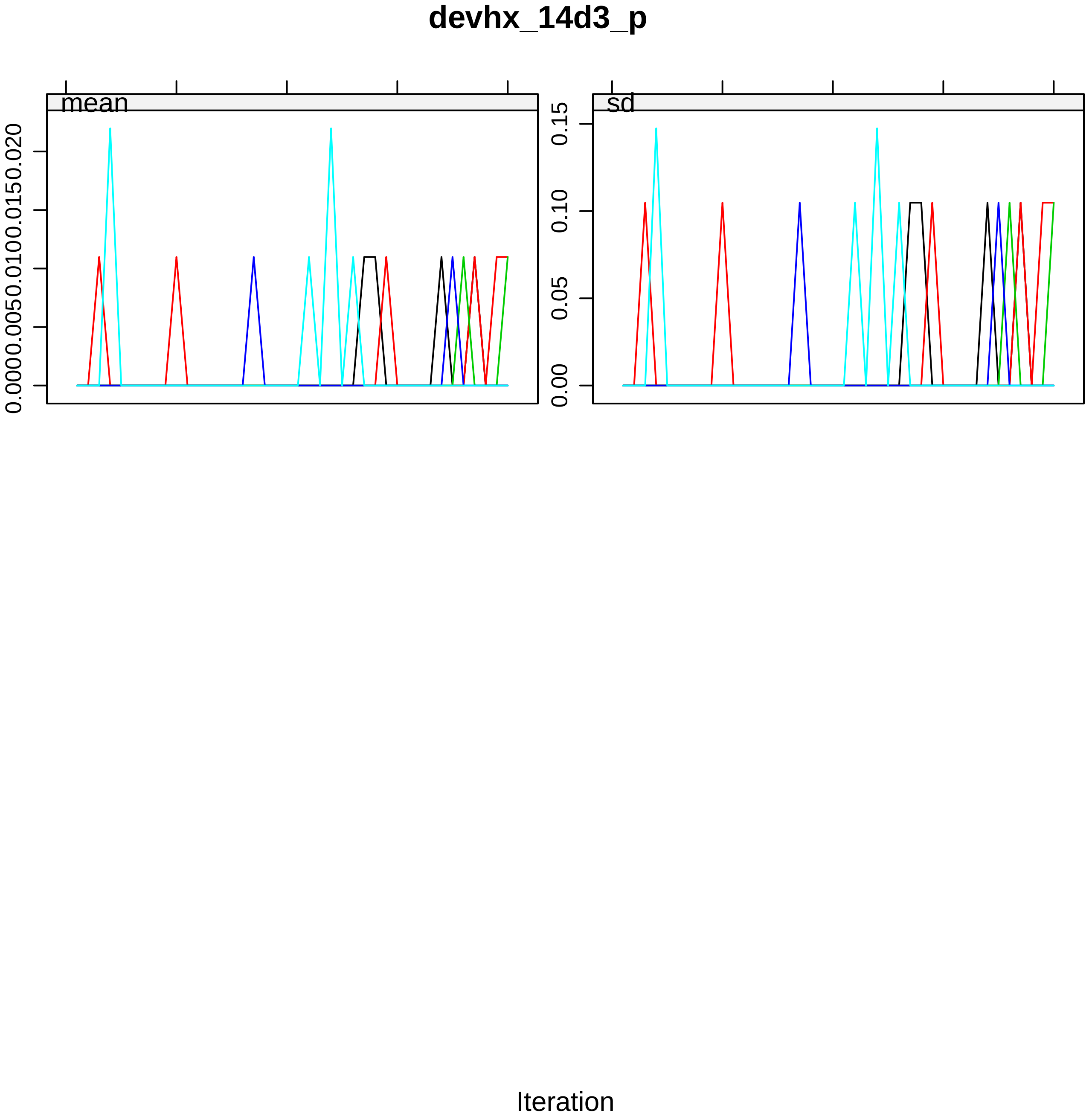

devhx_14d3_p
mean
sd
0.000
0.005
0.010
0.015
0.020
0.00
0.05
0.10
0.15
Iteration

devhx_14e3_p

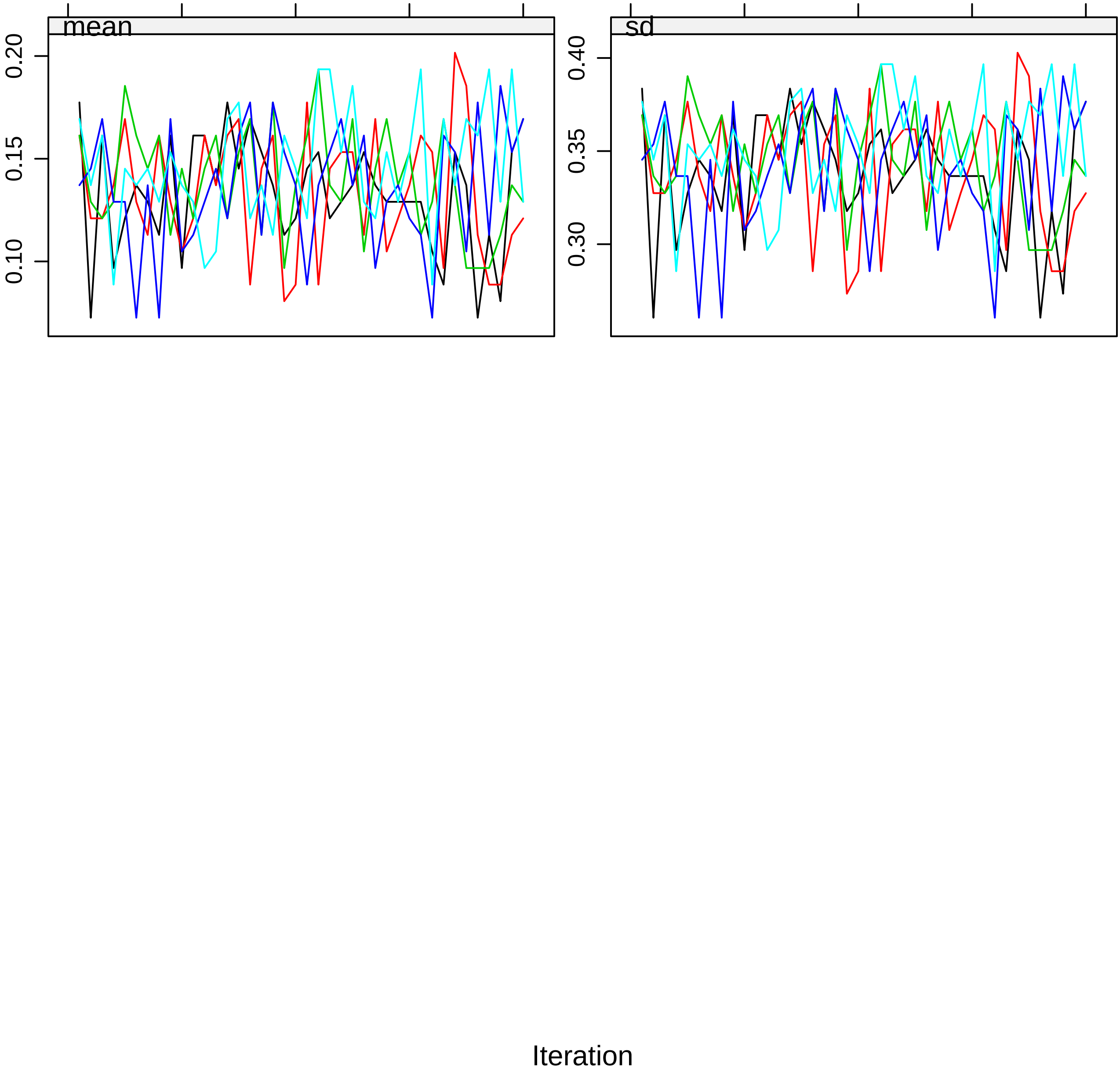

devhx_14f3_p

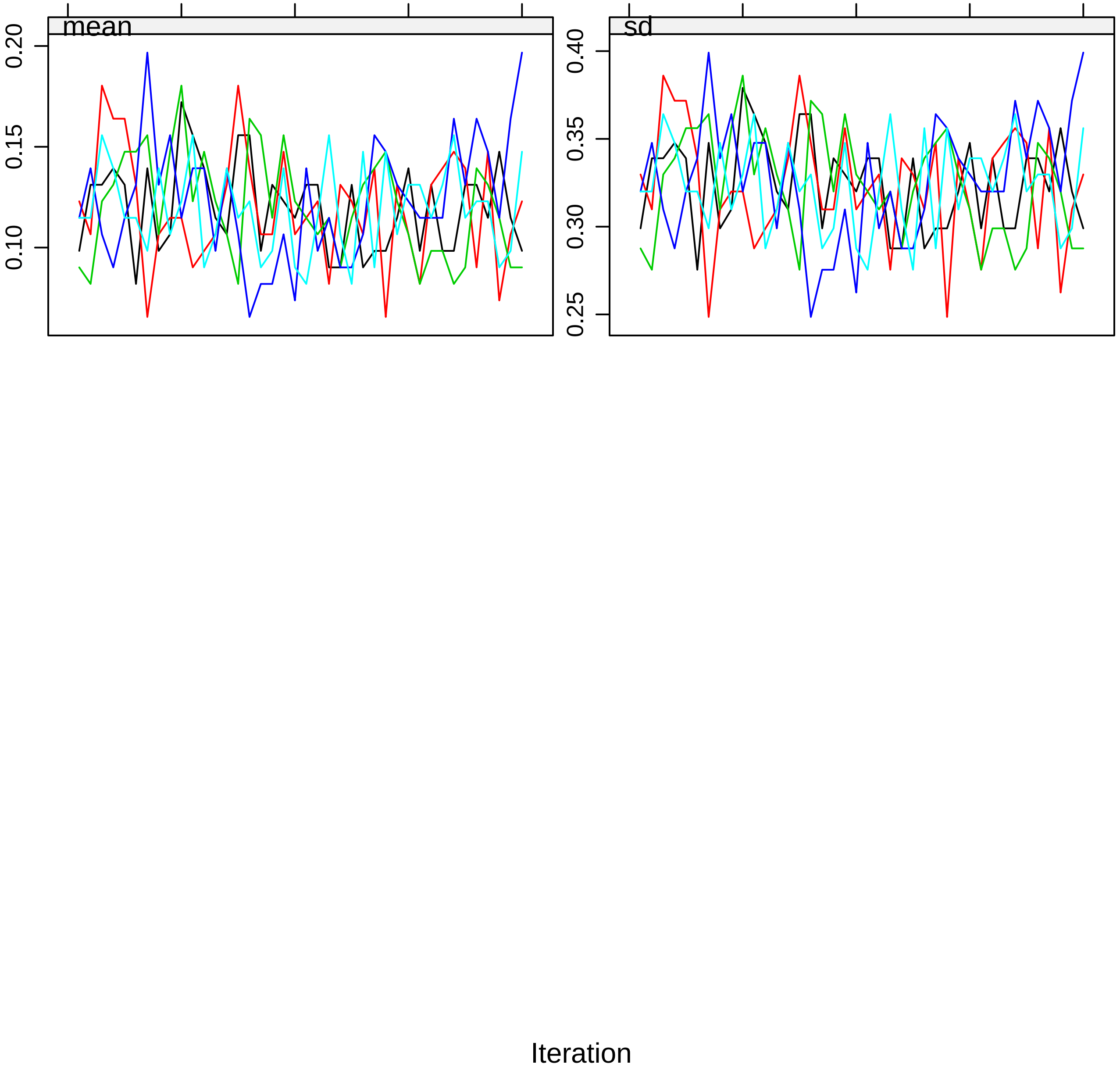

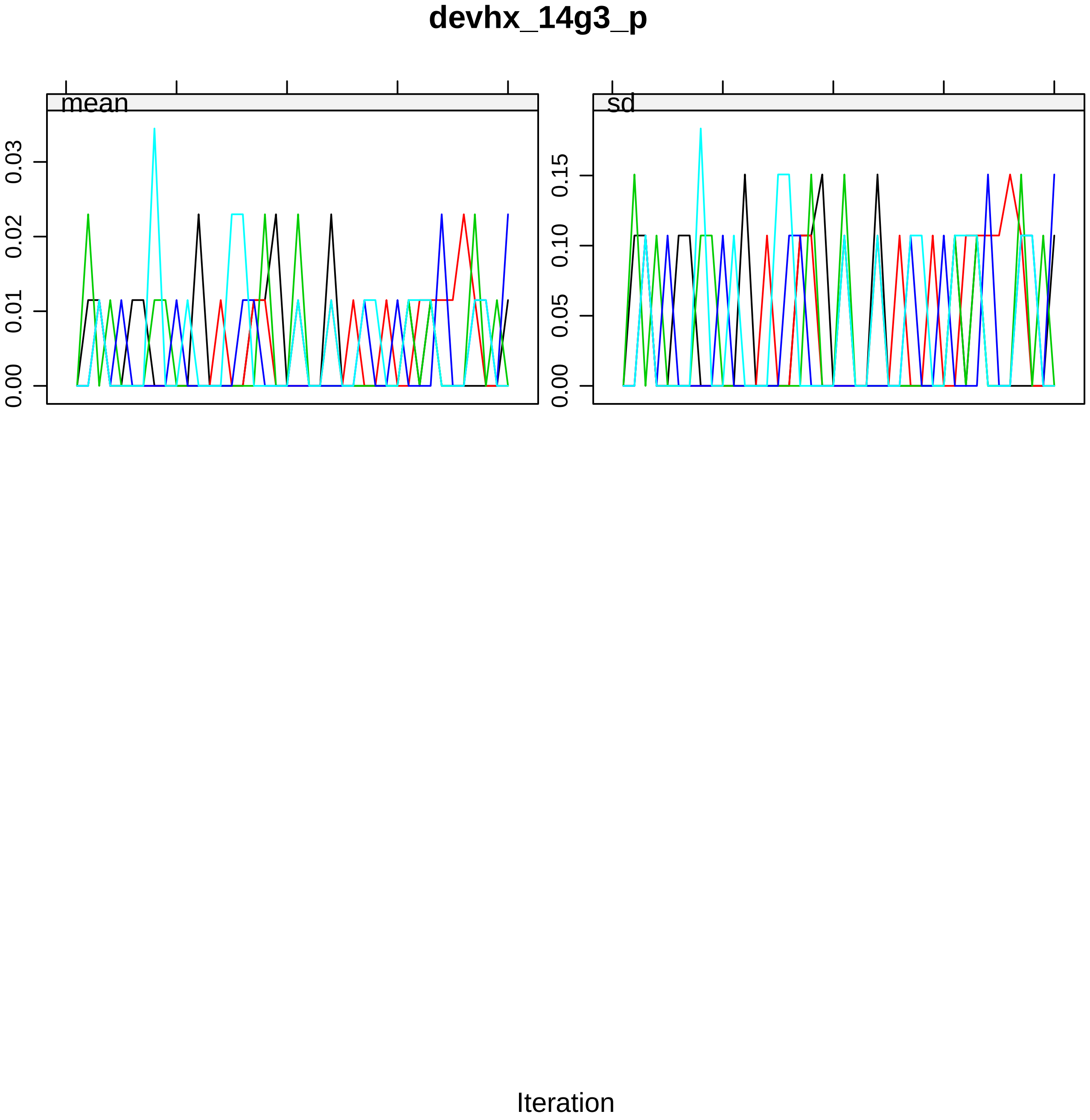
devhx_14g3_p
mean
sd
0.00
0.01
0.02
0.03
0.00
0.05
0.10
0.15
Iteration

devhx_14h3_p

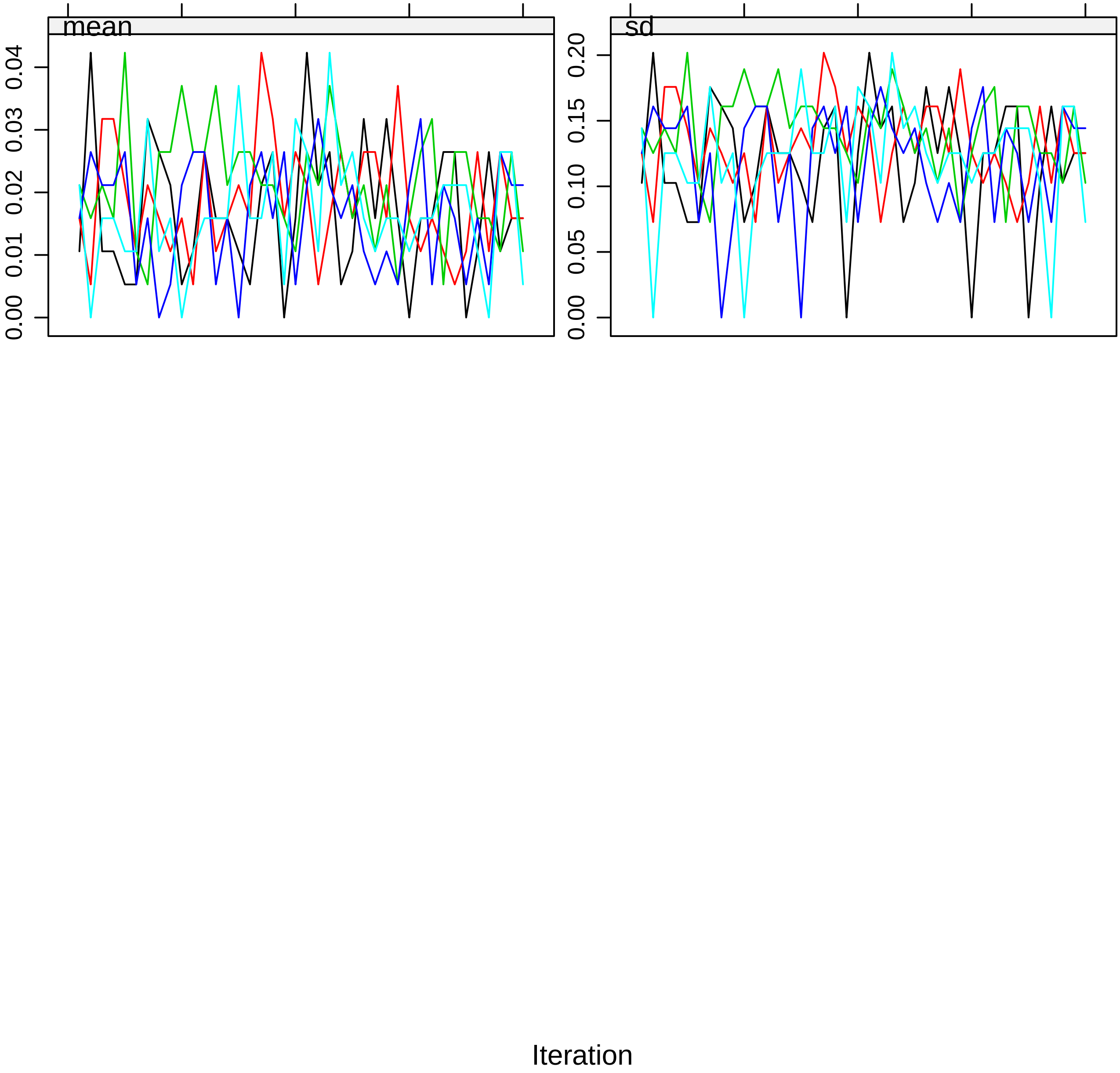

devhx_15

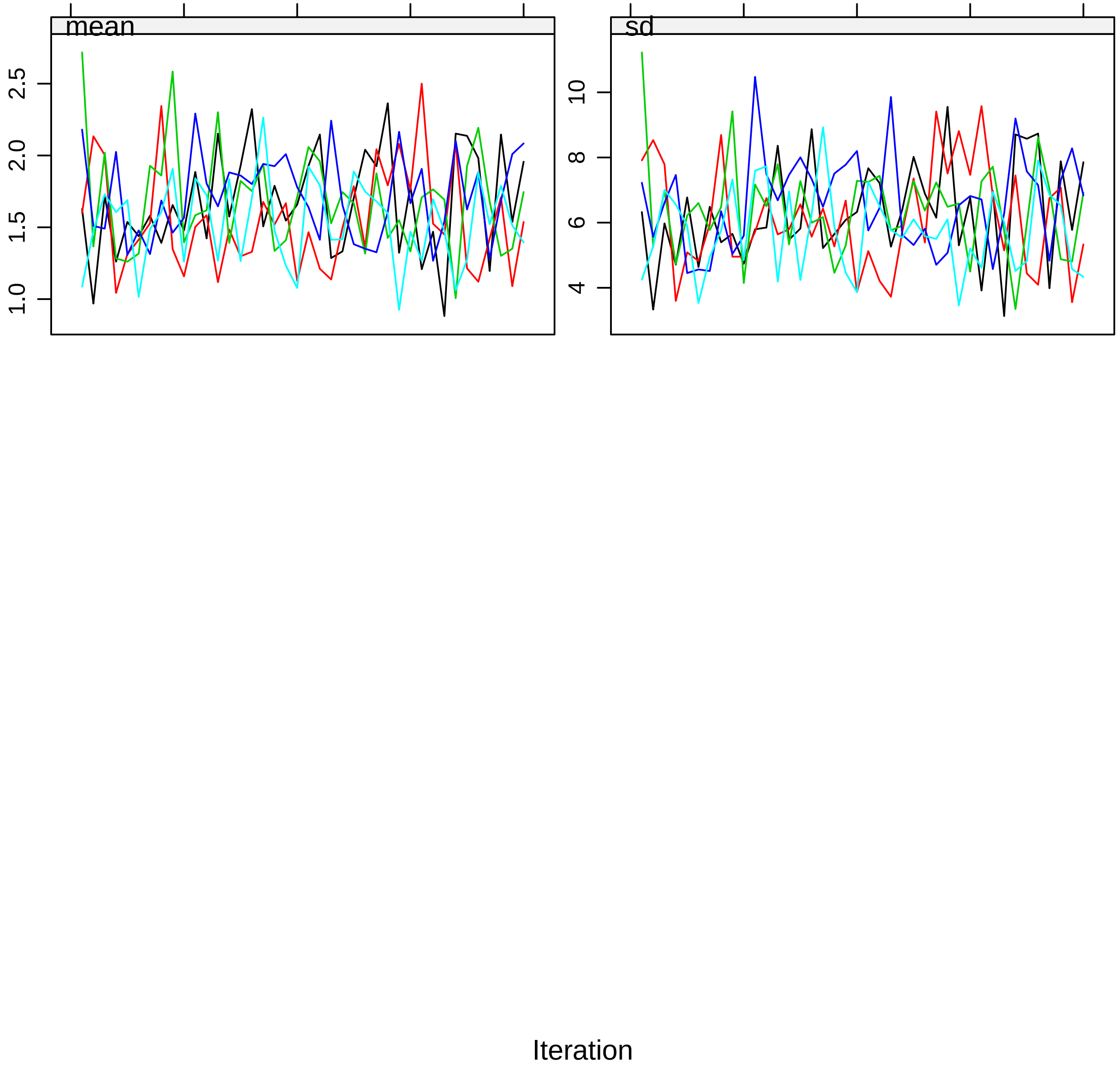

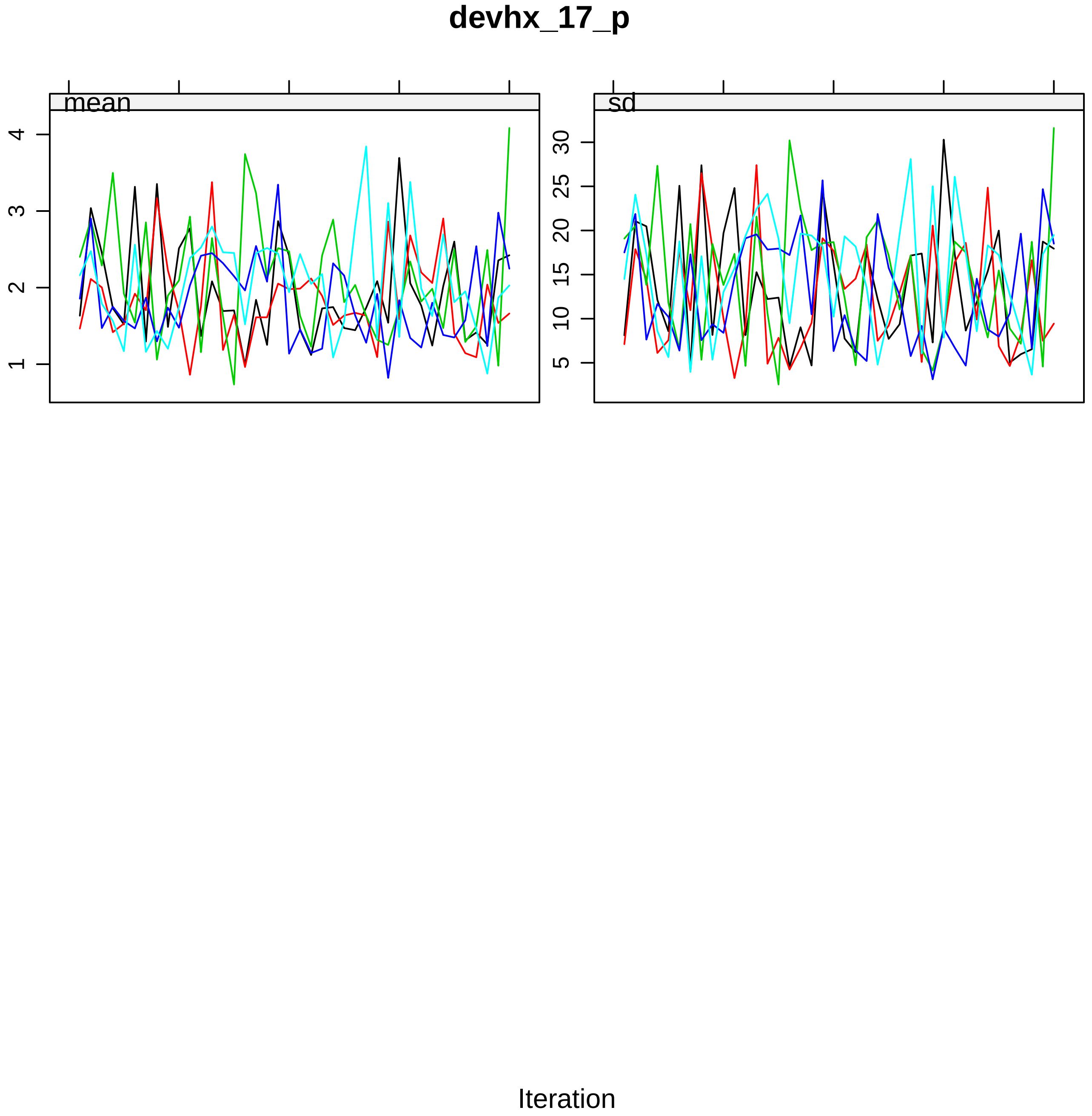

devhx_17_p
mean
sd
1
2
3
4
5
10
15
20
25
30
Iteration

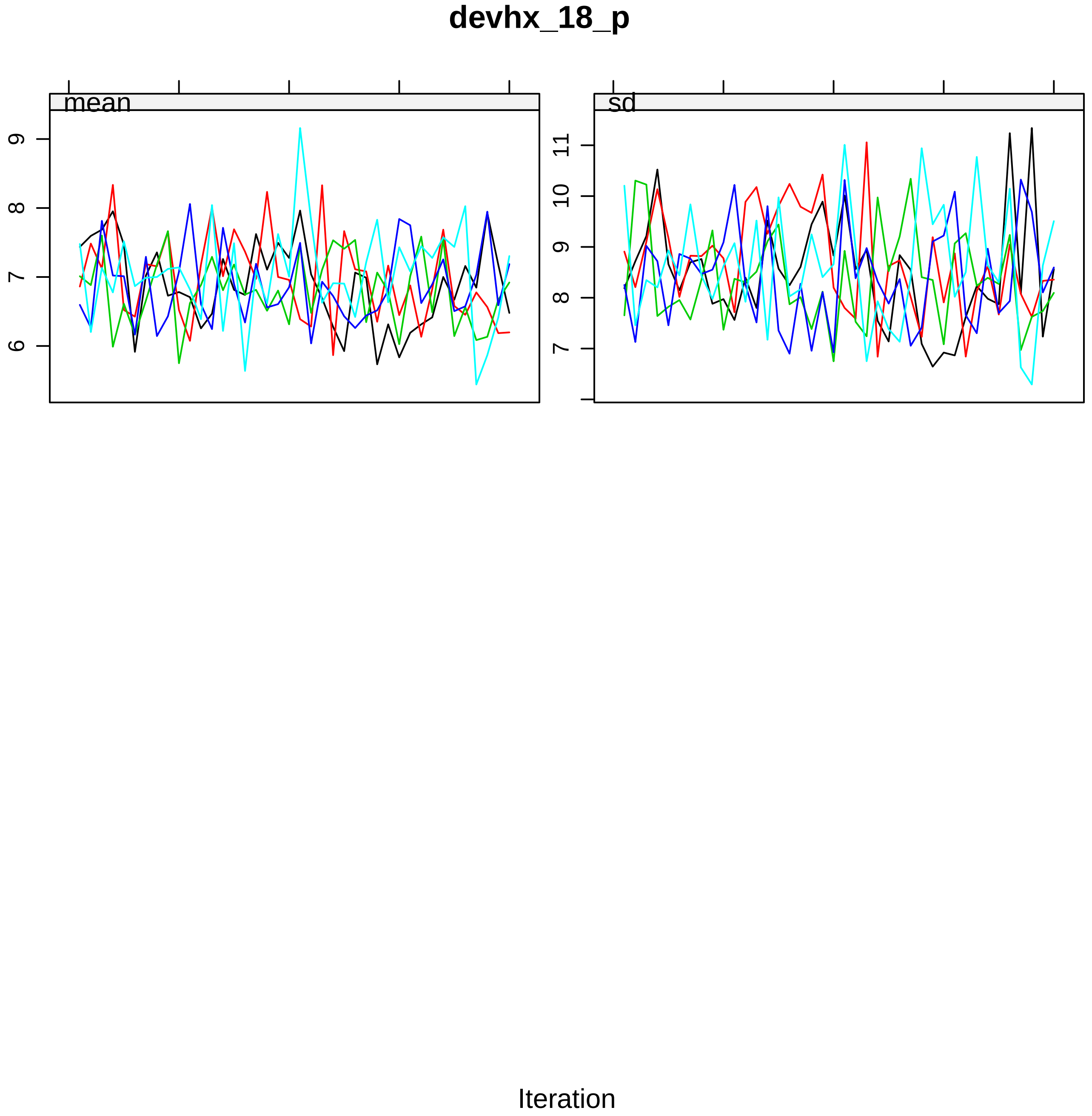

devhx_18_p
mean
sd
6
7
8
9
7
8
9
10
11
Iteration

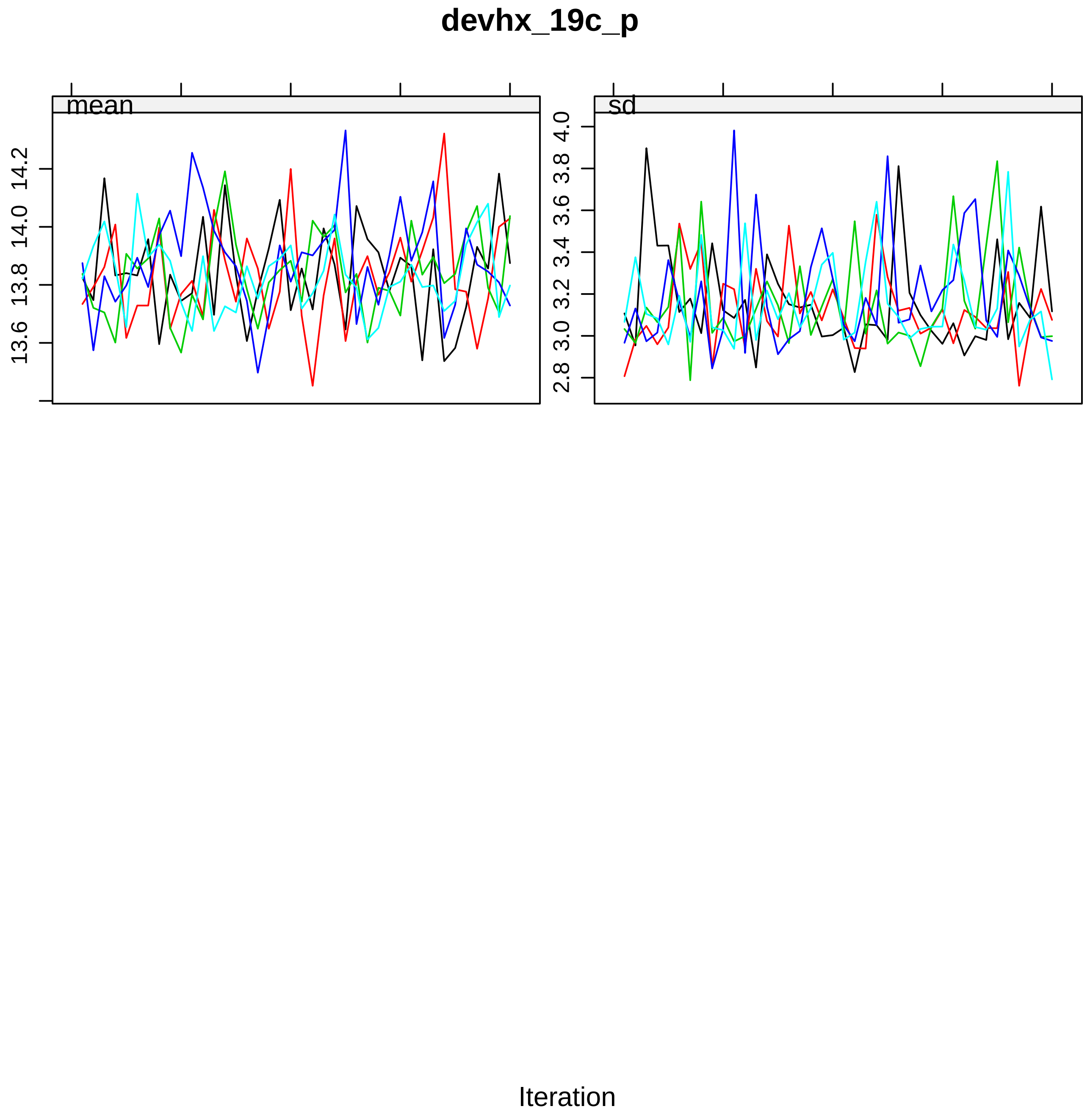
devhx_19c_p
mean
sd
13.6
13.8
14.0
14.2
2.8
3.0
3.2
3.4
3.6
3.8
4.0
Iteration

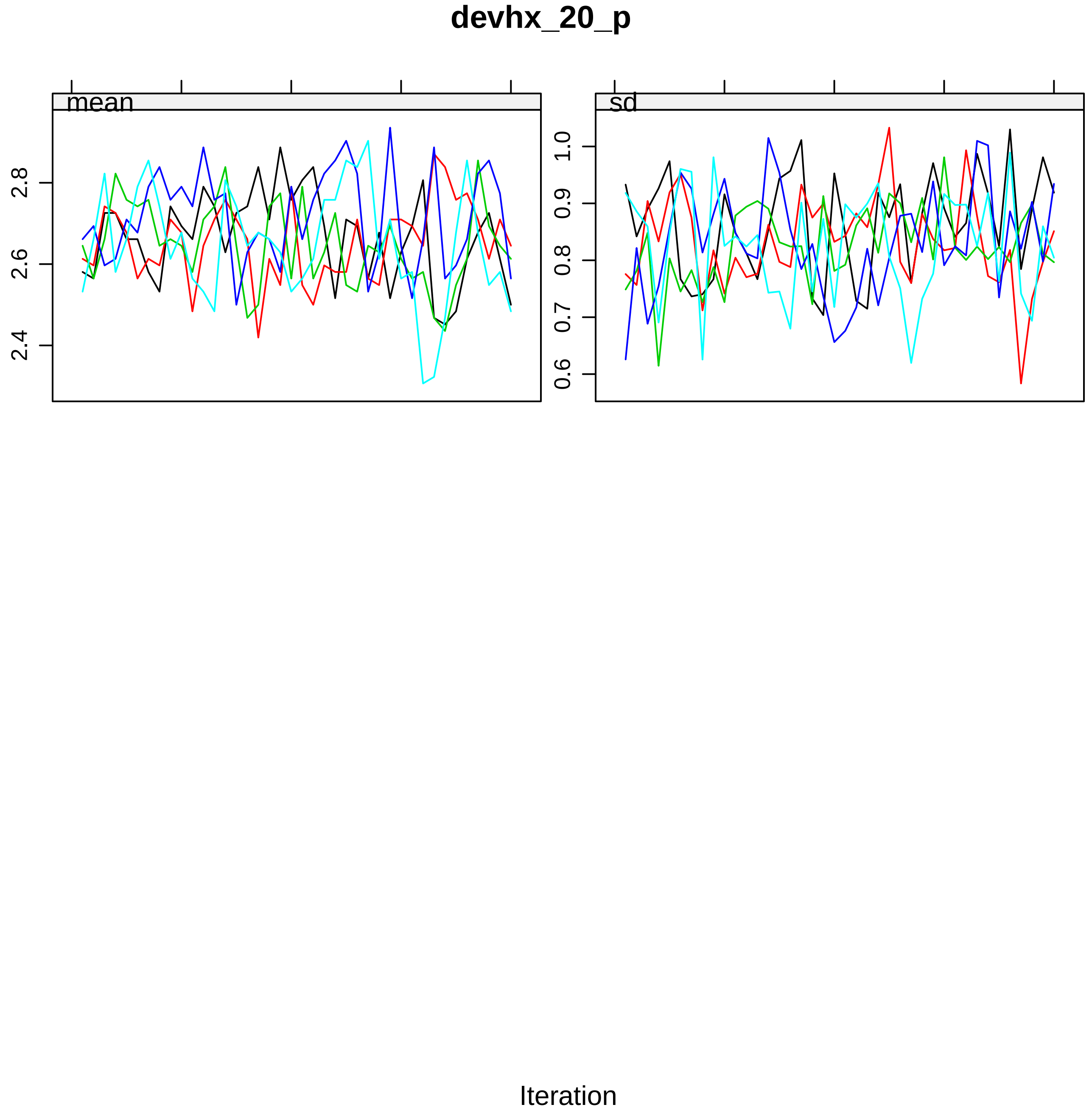
devhx_20_p
mean
sd
2.4
2.6
2.8
0.6
0.7
0.8
0.9
1.0
Iteration

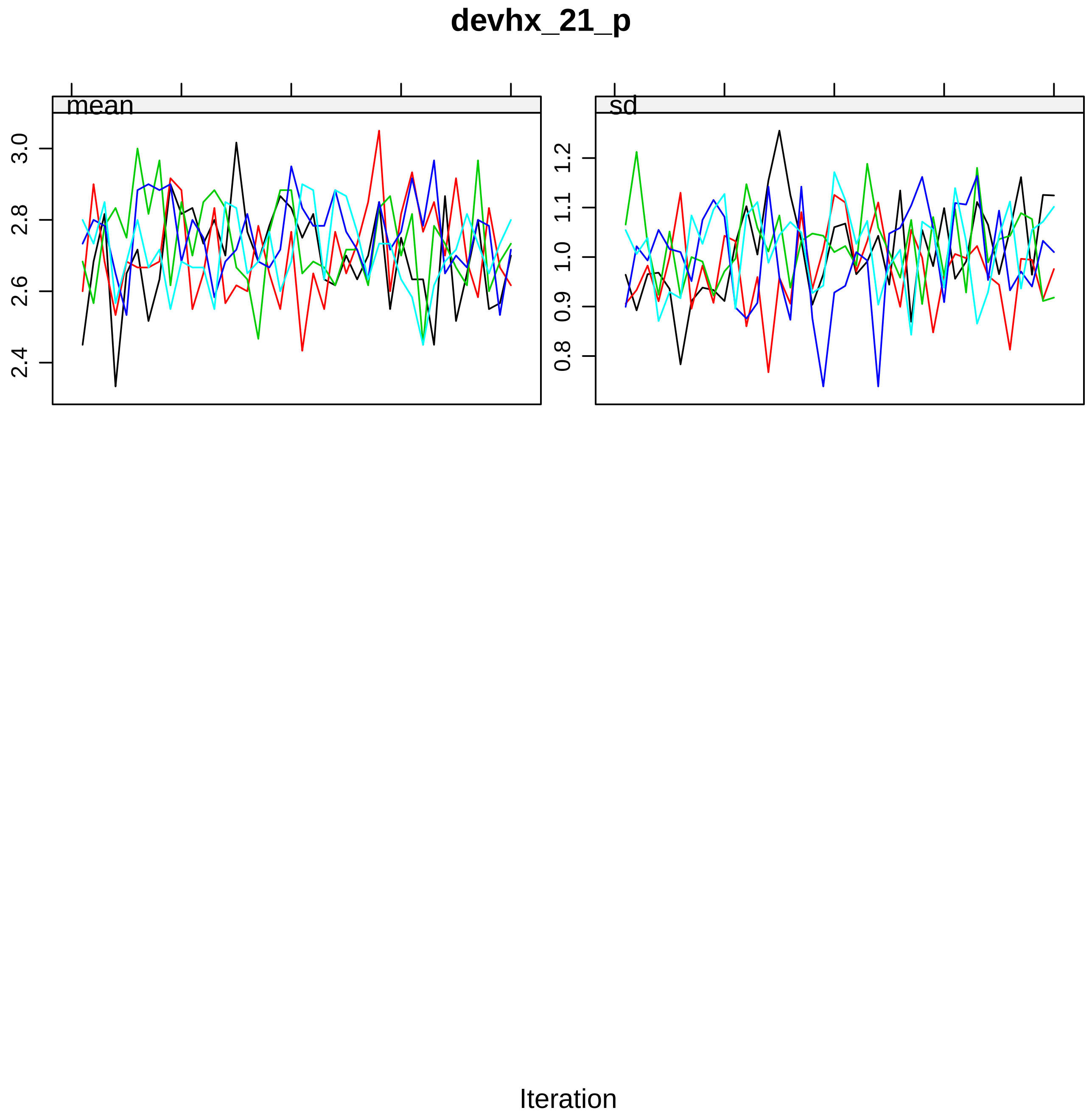
devhx_21_p
mean
sd
3.0
2.8
2.6
2.4
1.2
1.1
1.0
0.9
0.8
Iteration

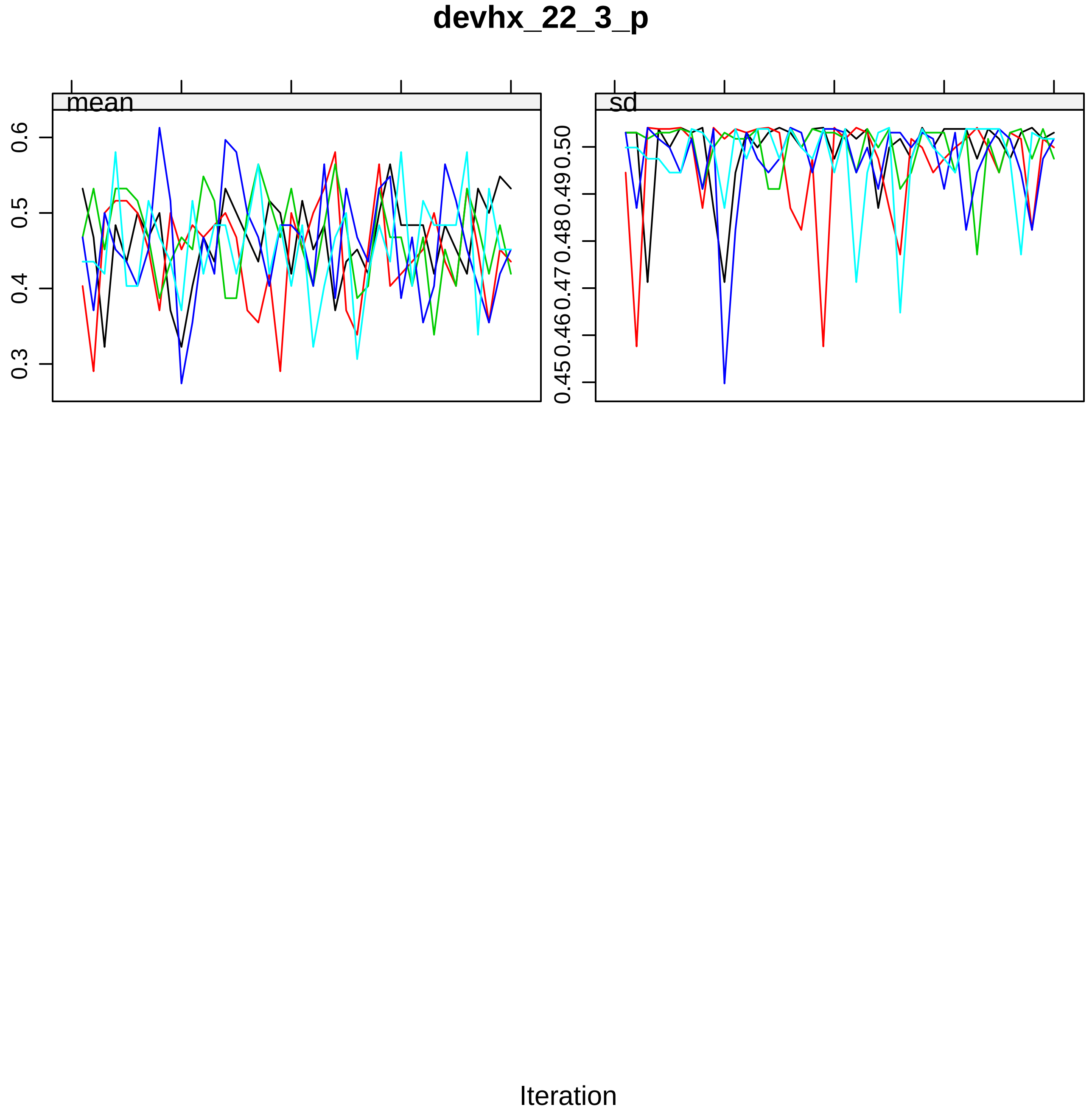
devhx_22_3_p
mean
sd
0.3
0.4
0.5
0.6
0.450
0.460
0.470
0.480
0.490
0.500
Iteration

Data Partition # 2

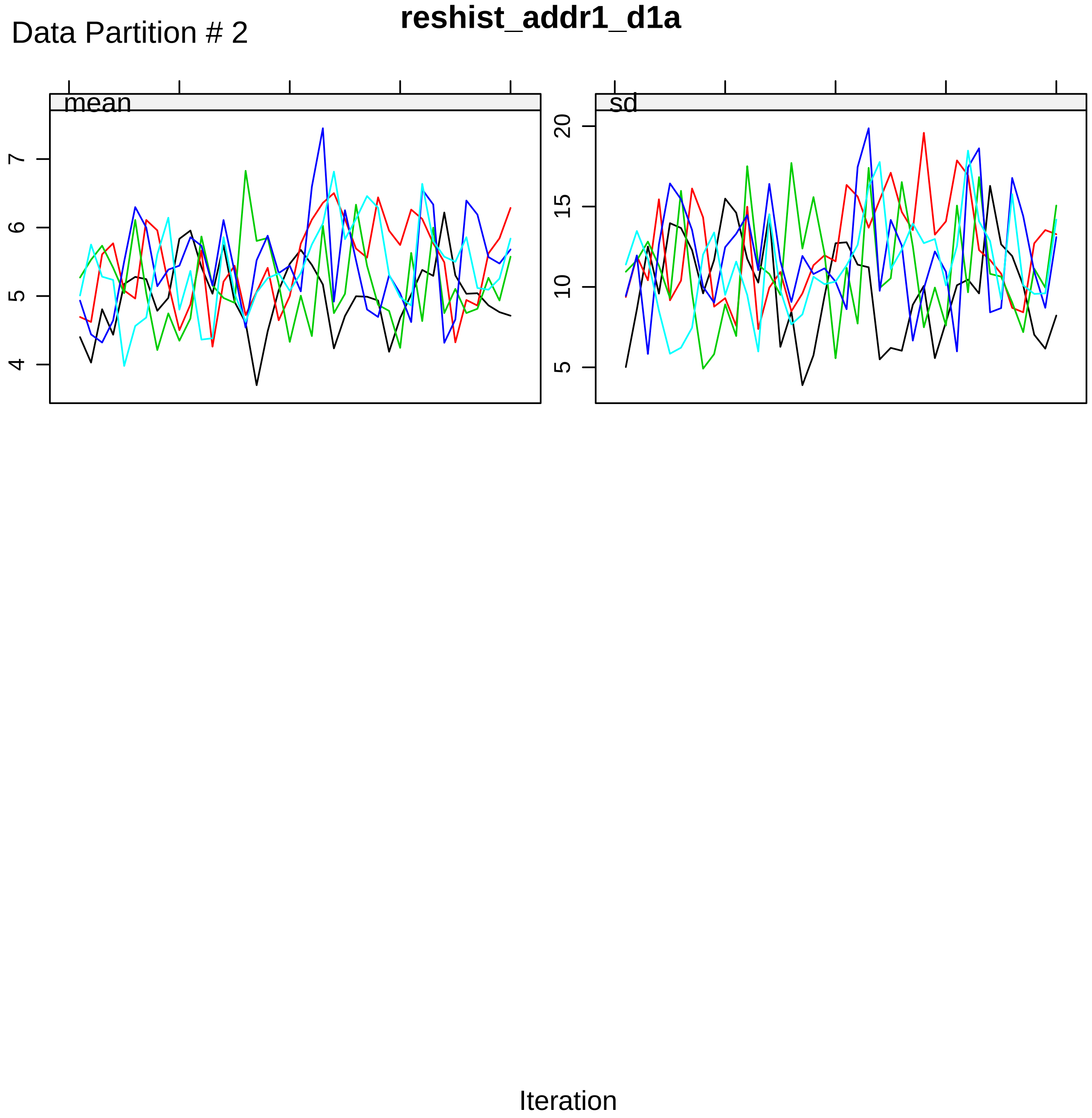

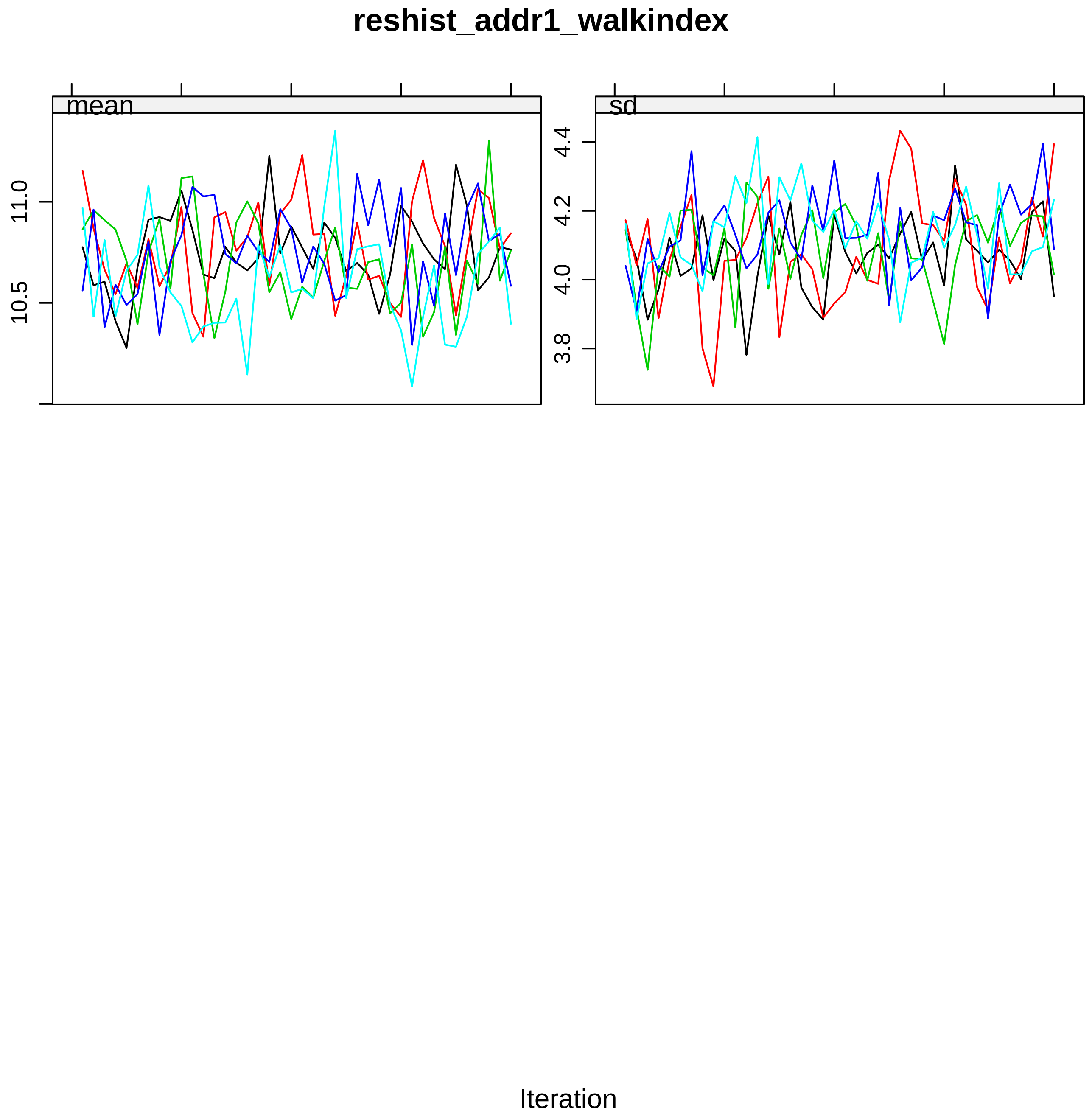
reshist_addr1_walkindex
mean
sd
11.0
10.5
4.4
4.2
4.0
3.8
Iteration

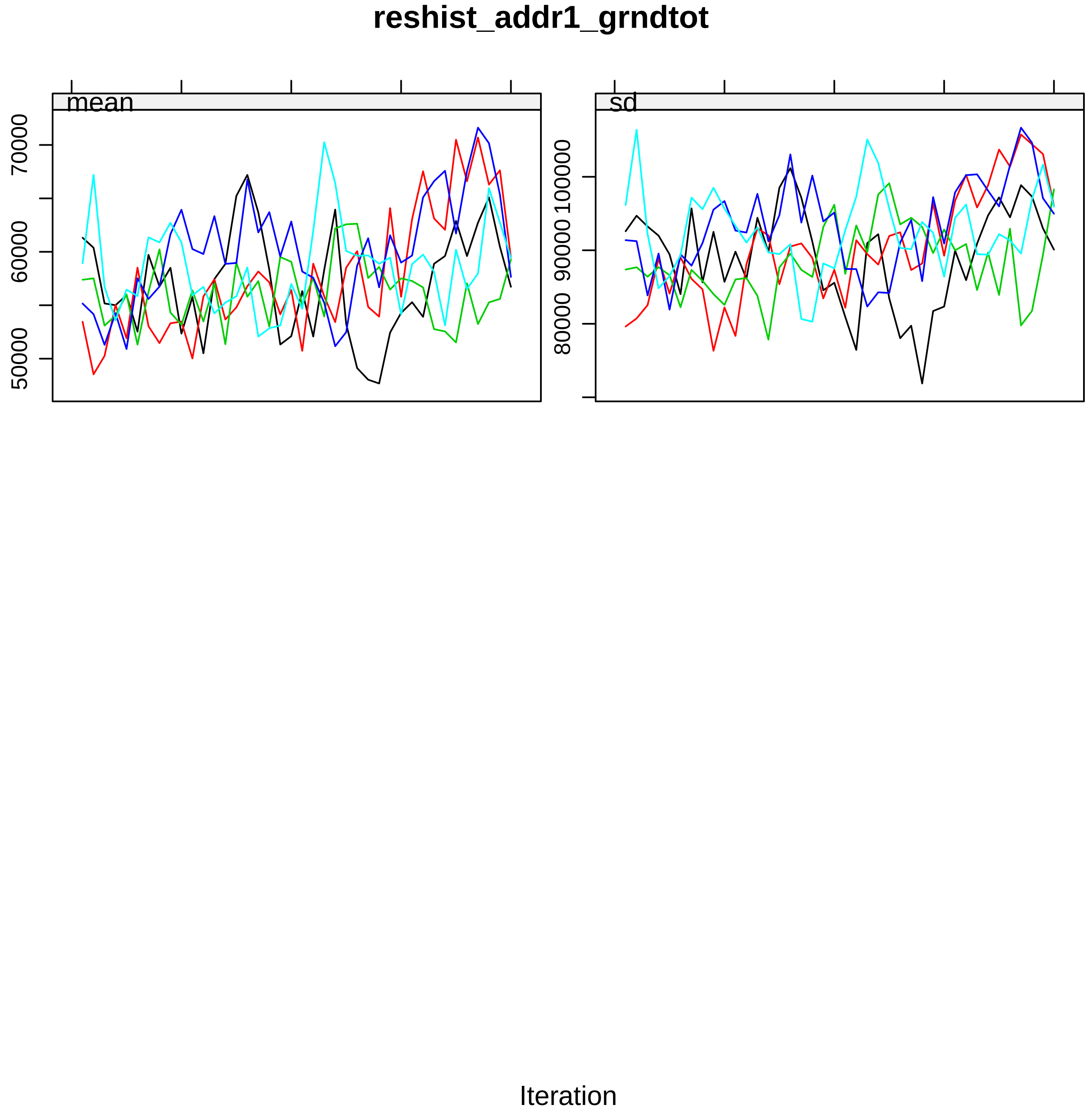
reshist_addr1_grndtot
mean
sd
50000
60000
70000
80000
90000
100000
Iteration

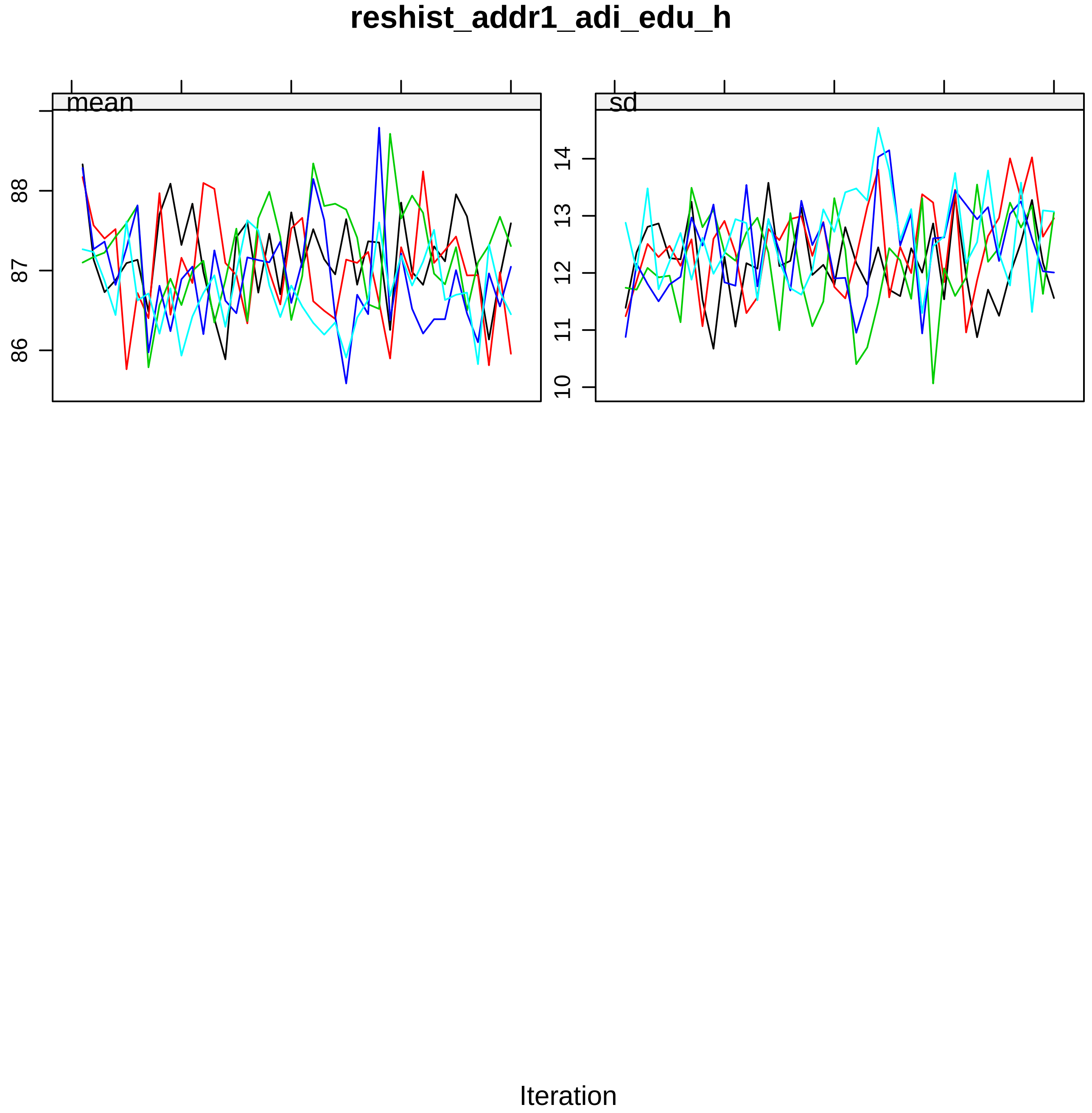

reshist_addr1_adi_edu_h
mean
sd
88
87
86
14
13
12
11
10
Iteration

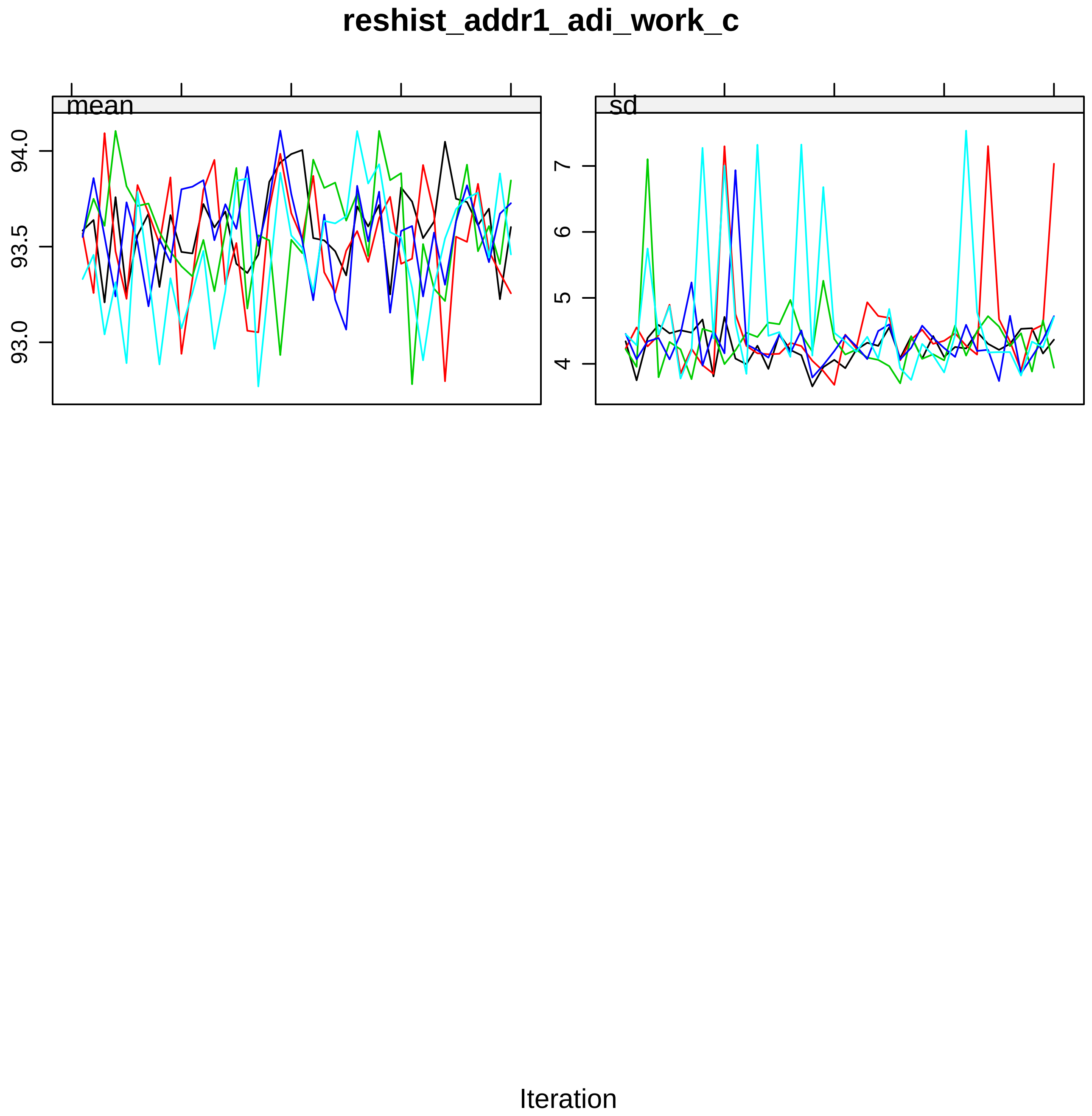
reshist_addr1_adi_work_c
mean
sd
94.0
93.5
93.0
7
6
5
4
Iteration

reshist_addr1_adi_income

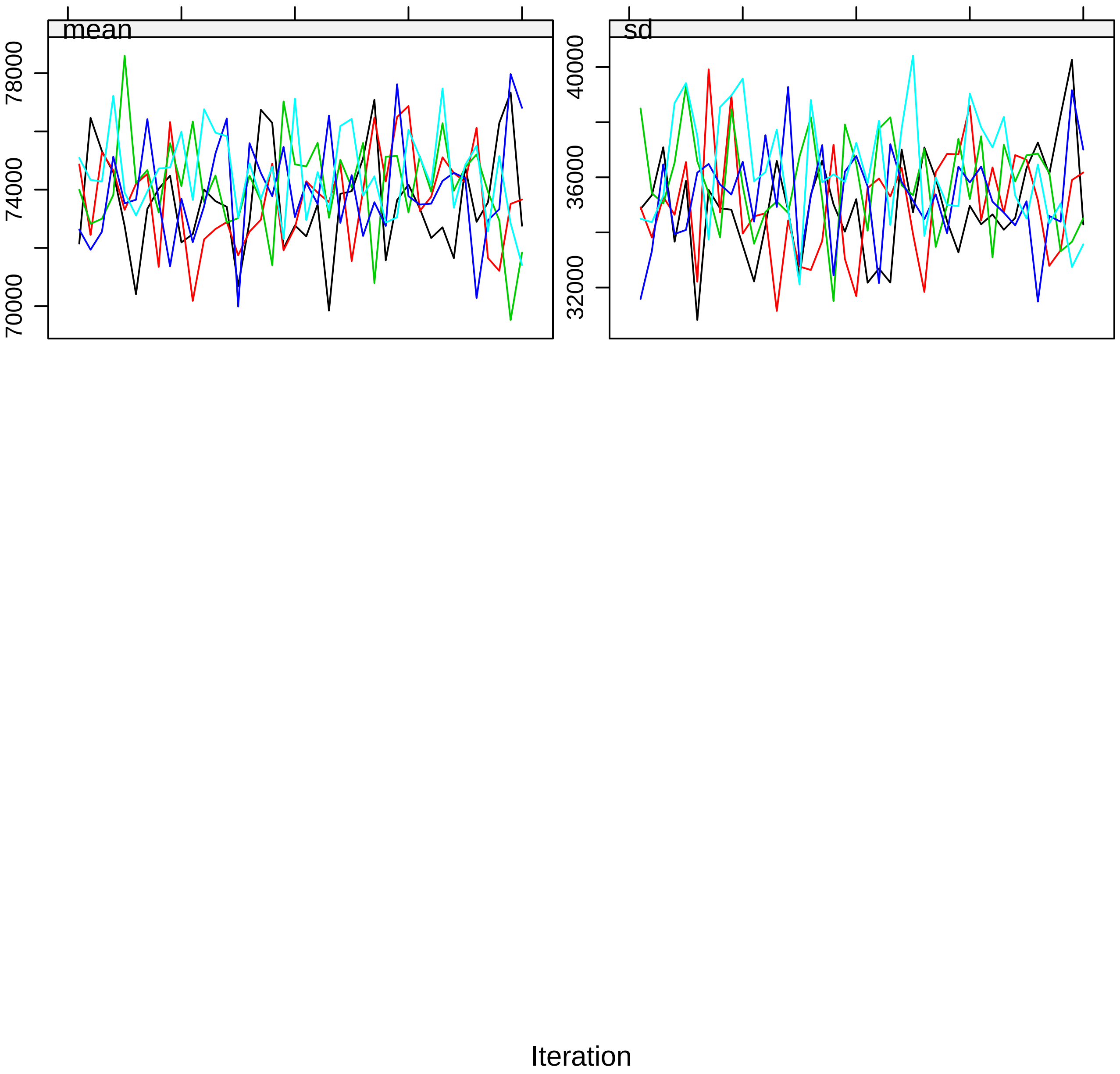

reshist_addr1_adi_home_v

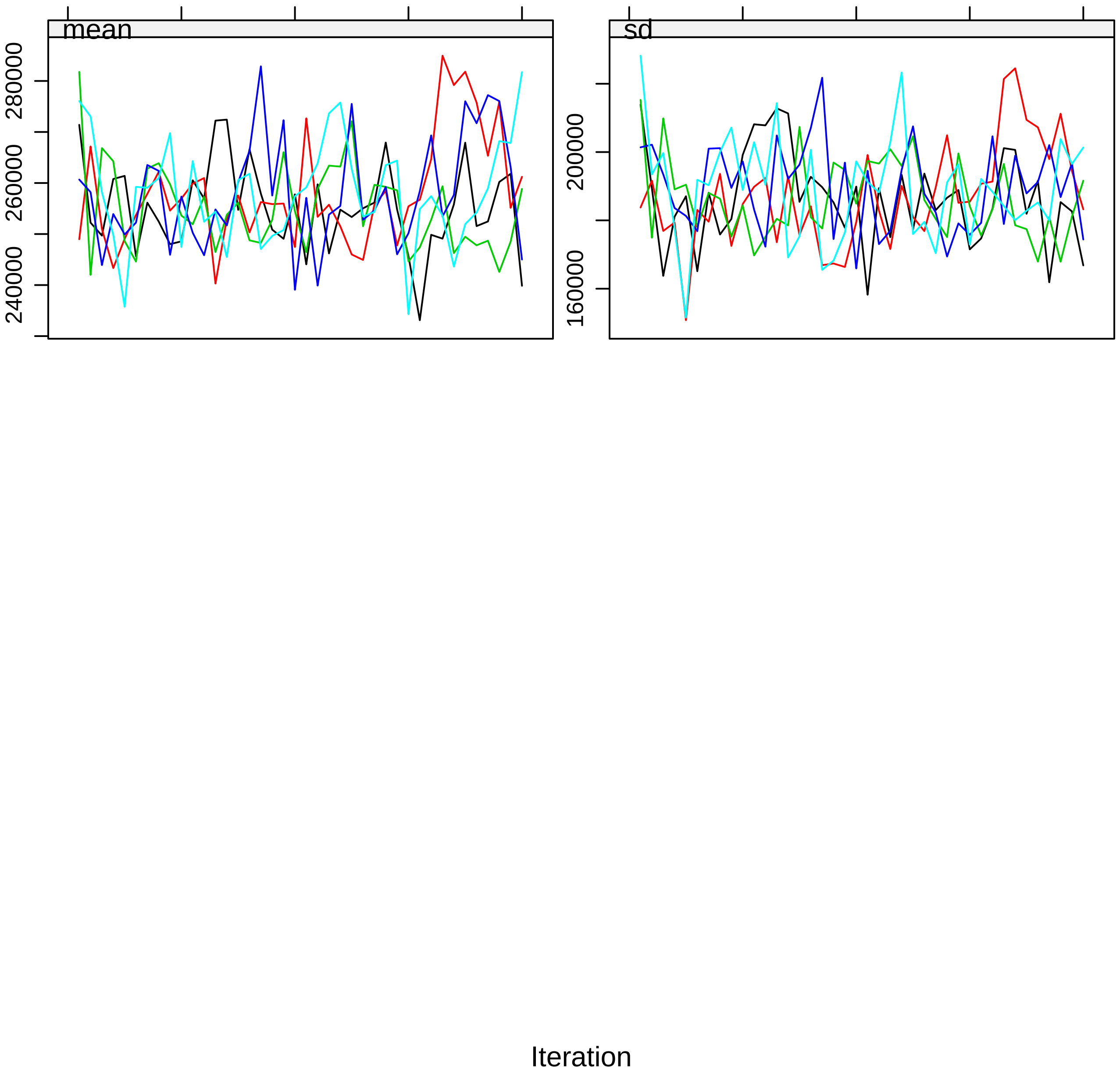

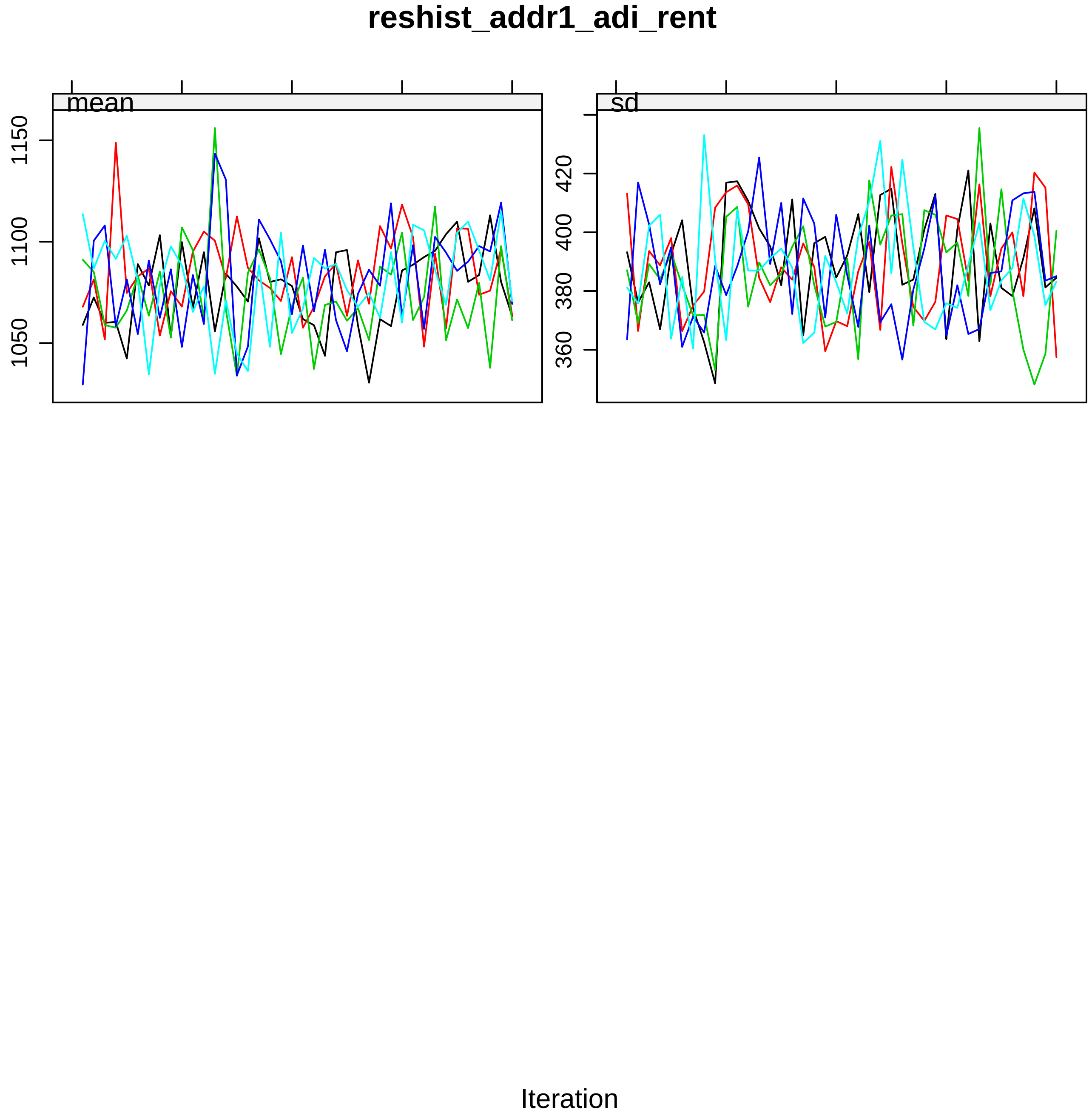
reshist_addr1_adi_rent
mean
sd
1050
1100
1150
360
380
400
420
Iteration

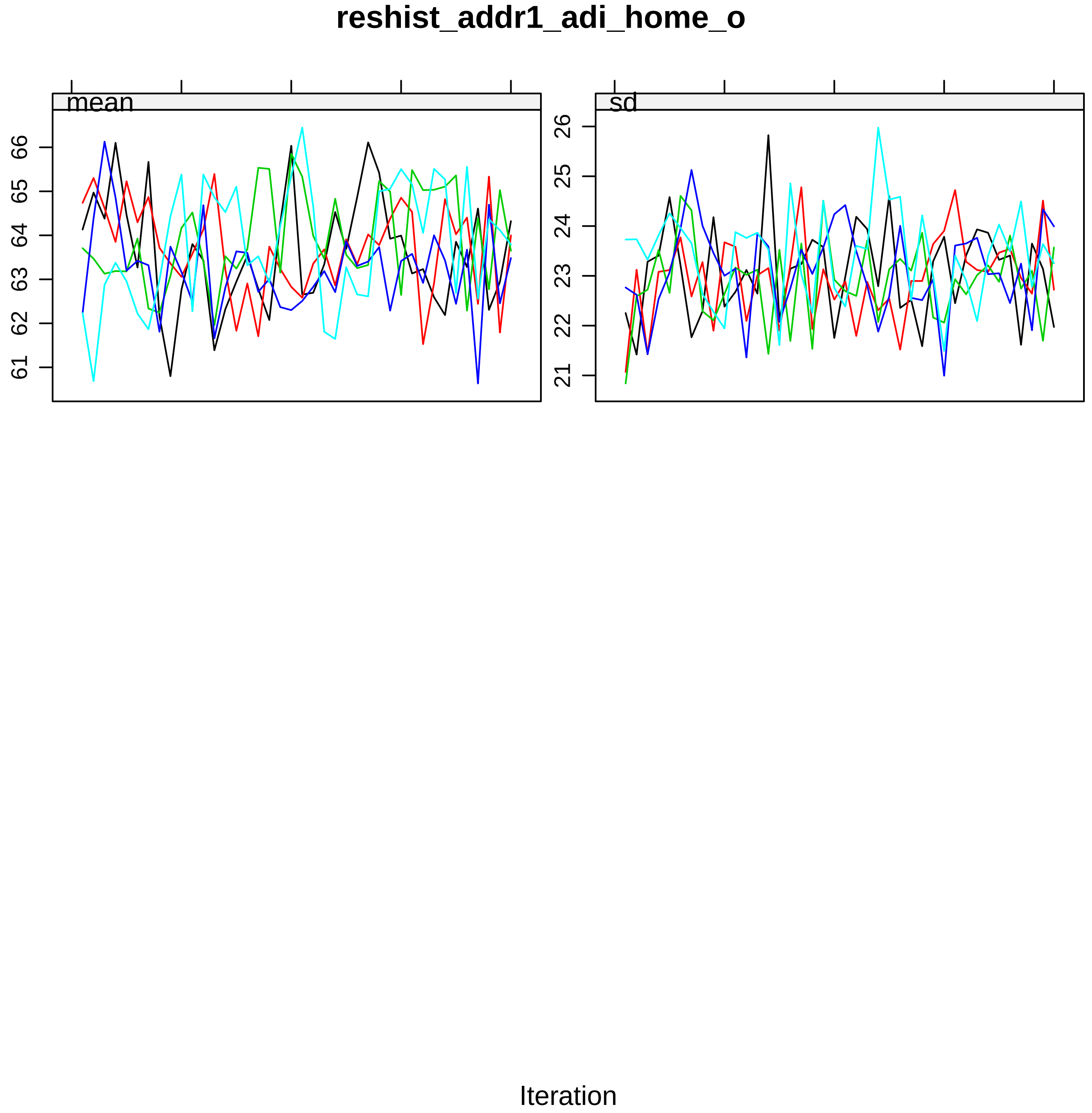

reshist_addr1_adi_home_o
mean
sd
61
62
63
64
65
66
21
22
23
24
25
26
Iteration

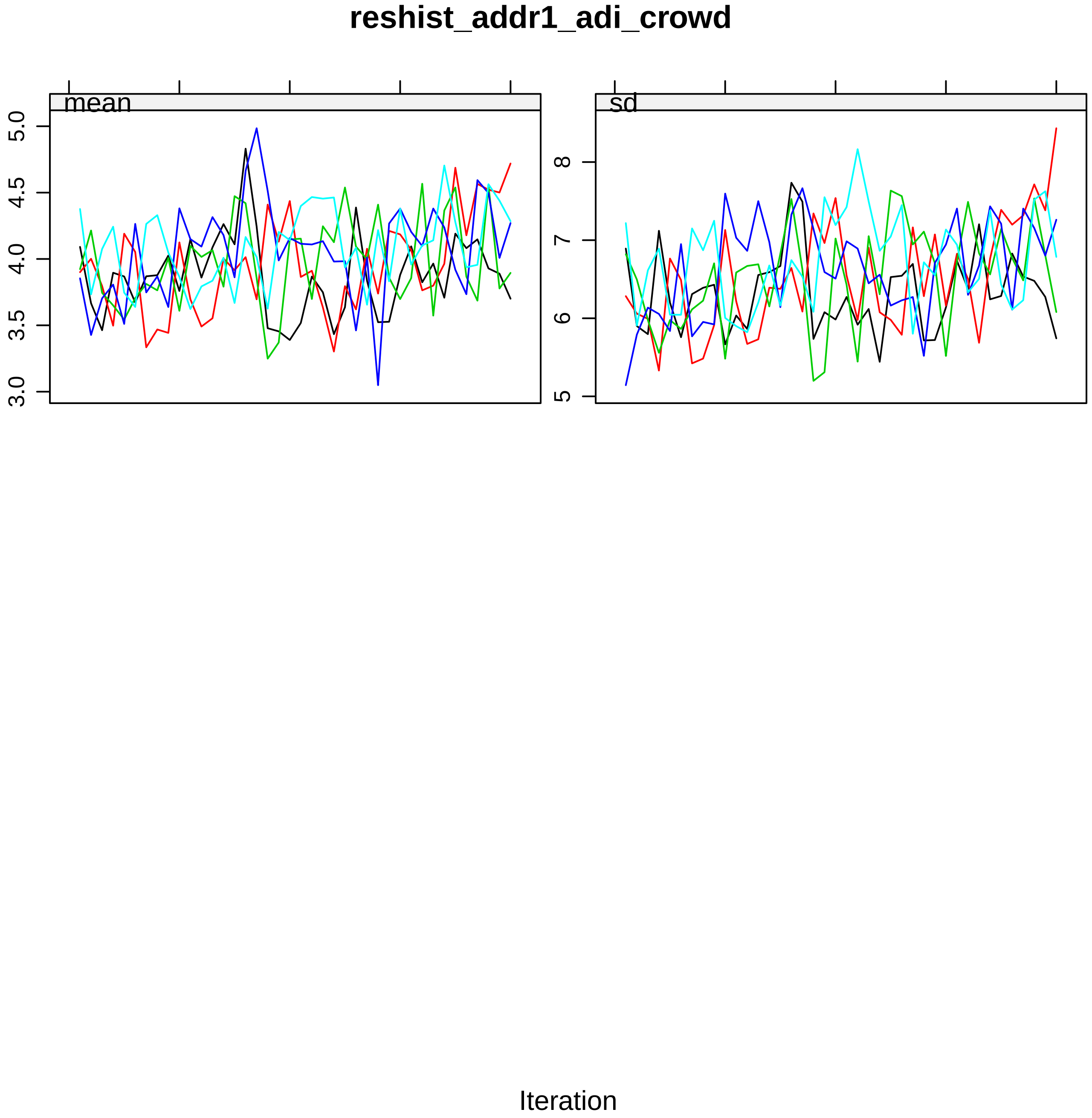
reshist_addr1_adi_crowd
mean
sd
5.0
4.5
4.0
3.5
3.0
8
7
6
5
Iteration

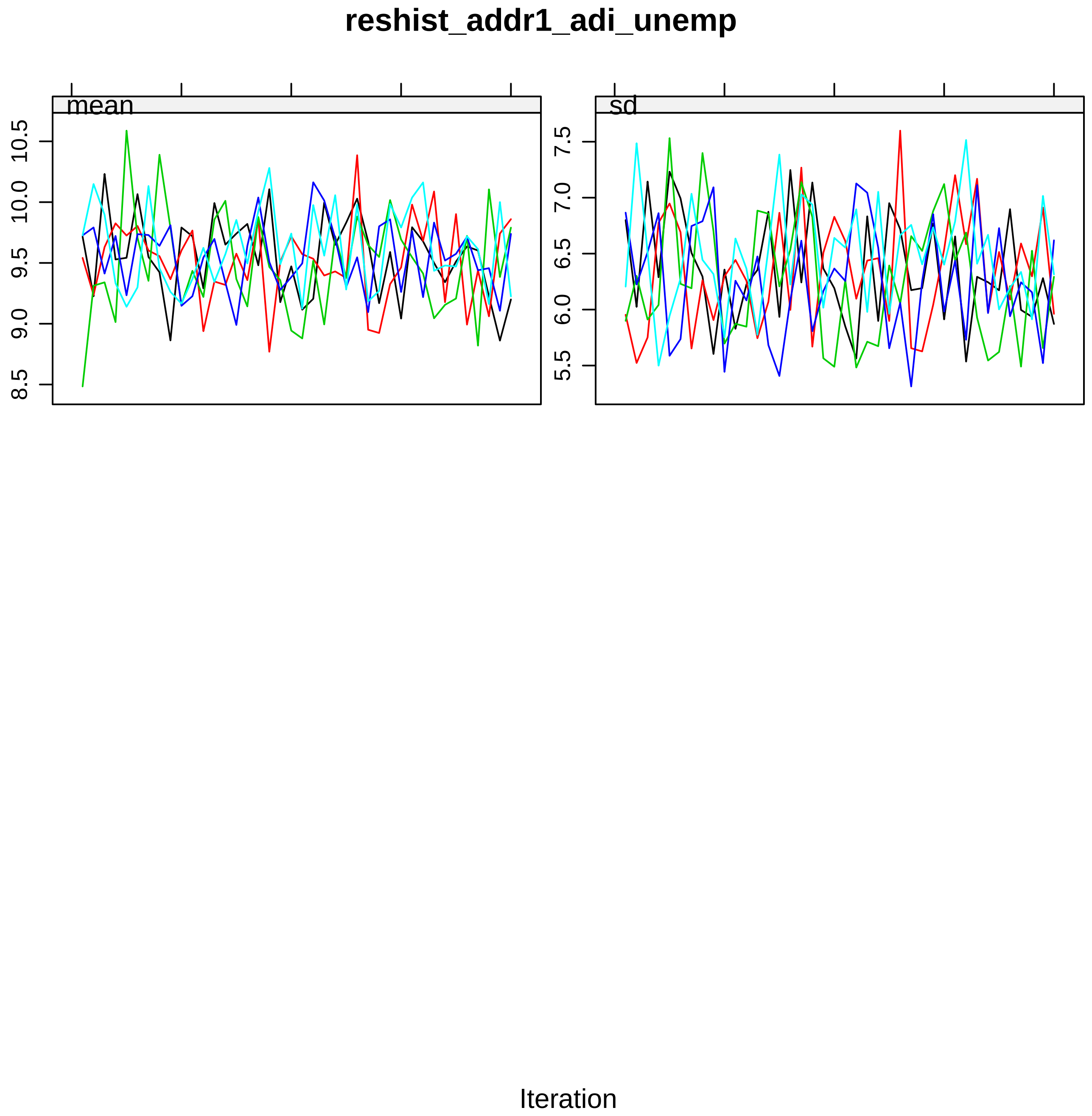
reshist_addr1_adi_unemp
mean
sd
10.5
10.0
9.5
9.0
8.5
7.5
7.0
6.5
6.0
5.5
Iteration

reshist_addr1_adi_b138

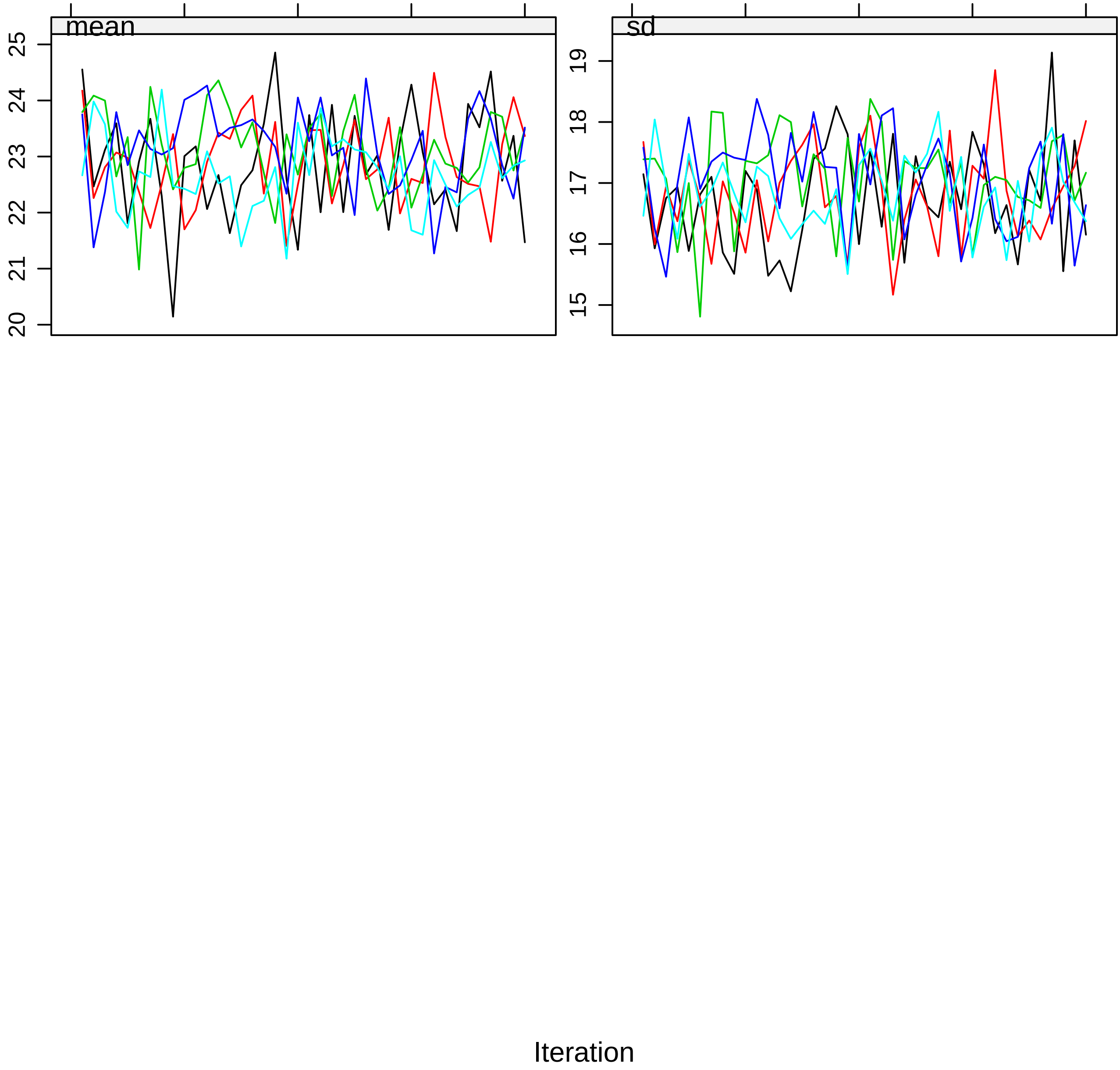

reshist_addr1_adi_sp

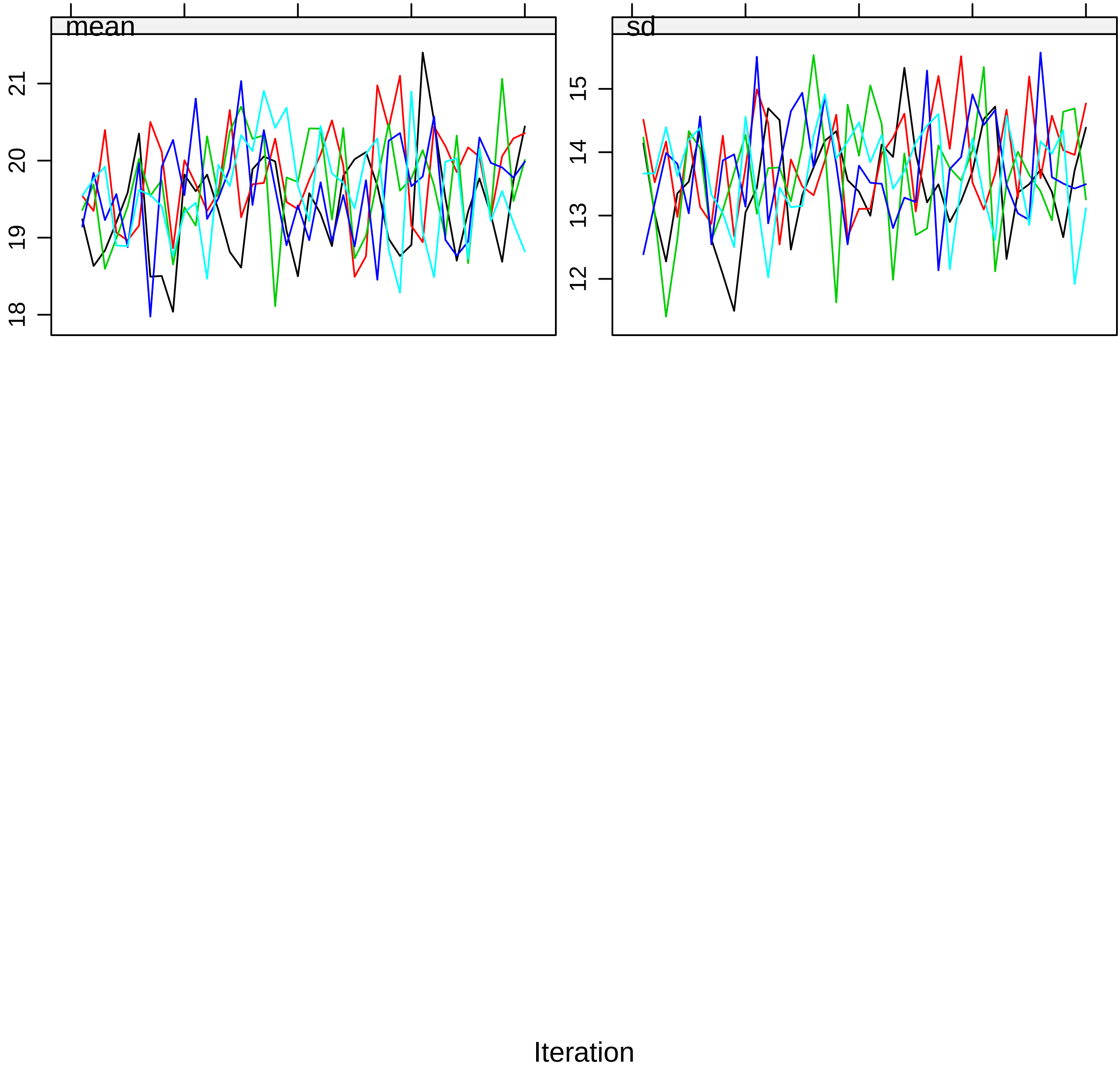

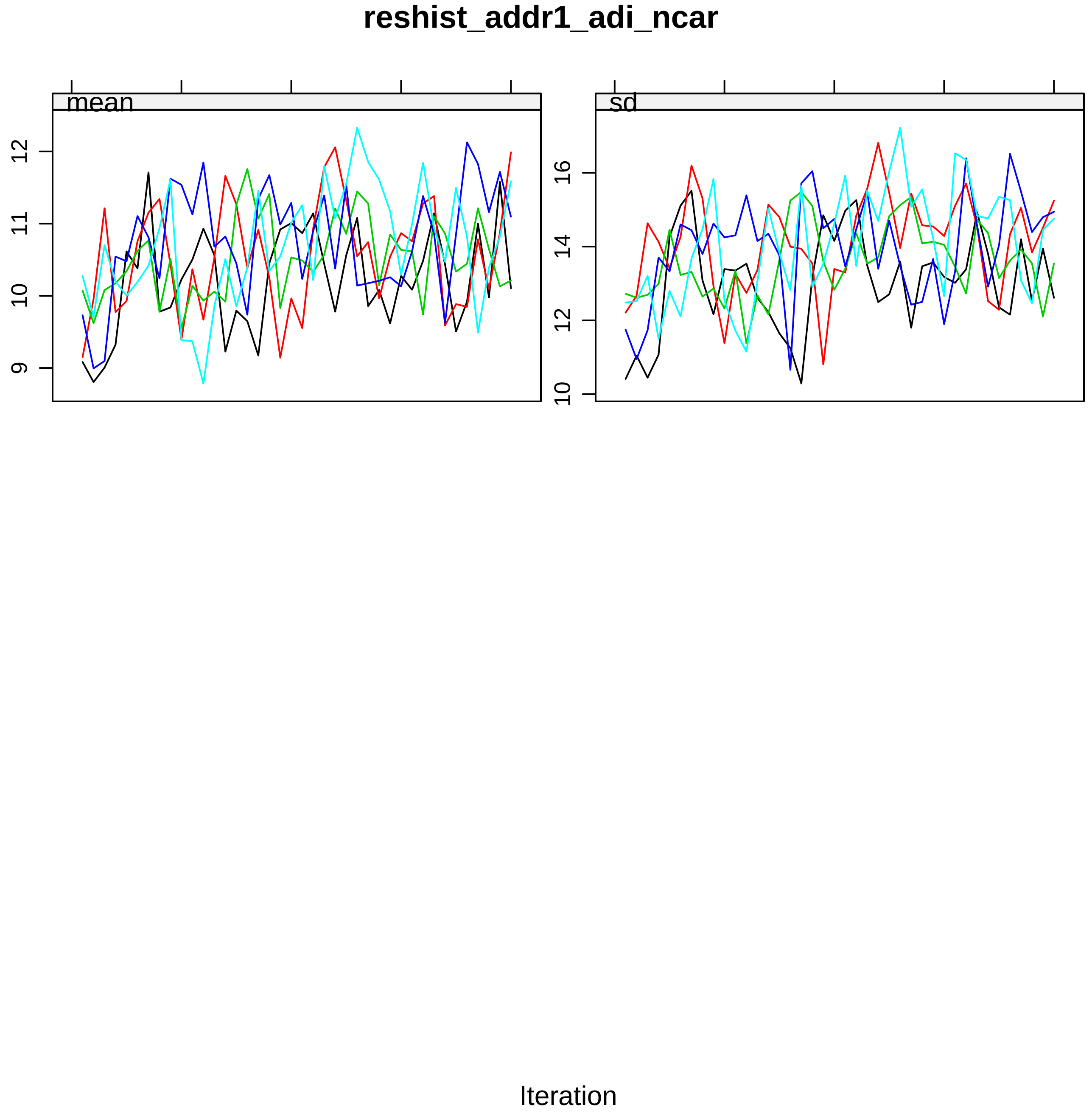
reshist_addr1_adi_ncar
mean
sd
9
10
11
12
10
12
14
16
Iteration

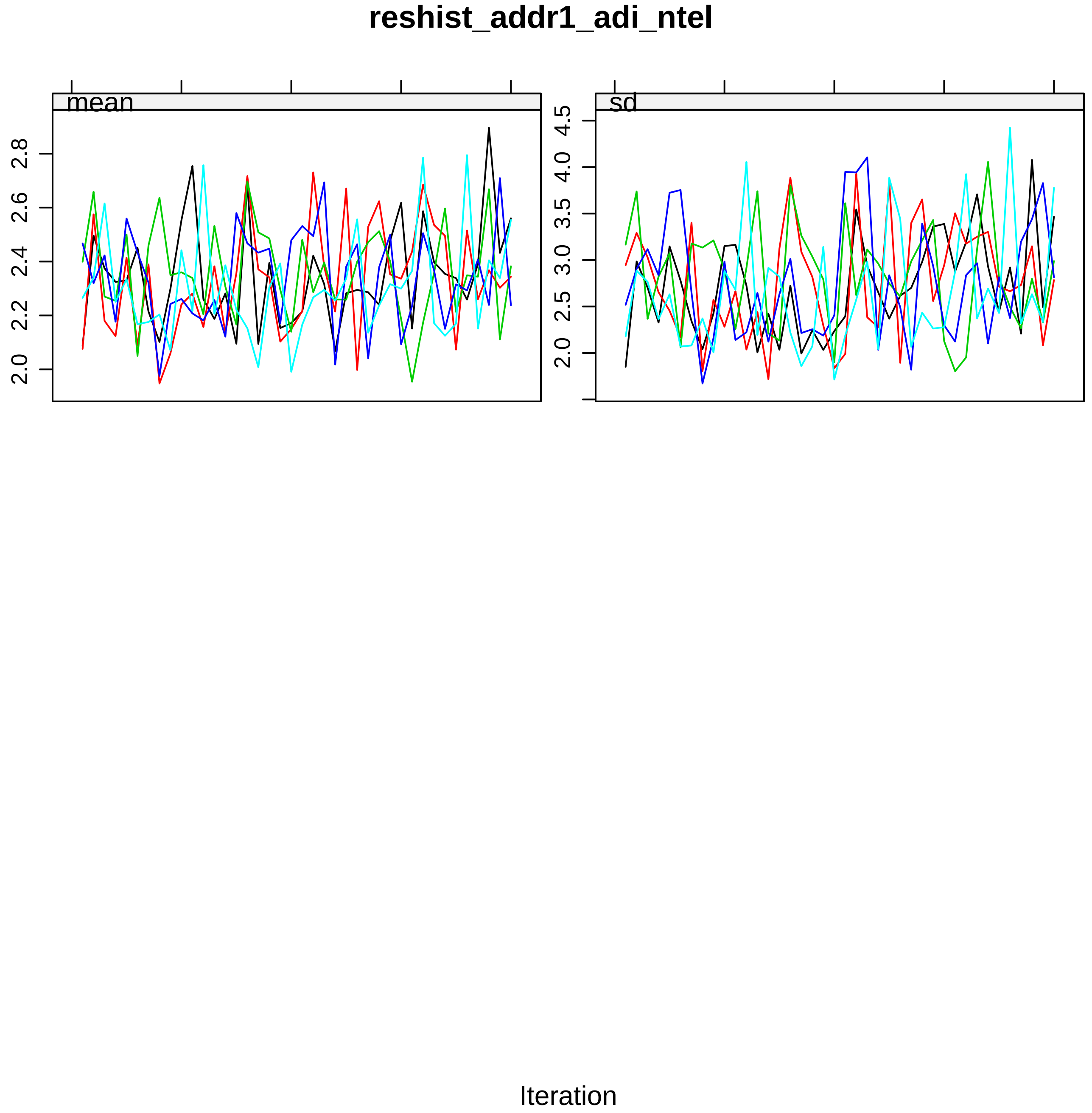
reshist_addr1_adi_ntel
mean
sd
2.0
2.2
2.4
2.6
2.8
2.0
2.5
3.0
3.5
4.0
4.5
Iteration

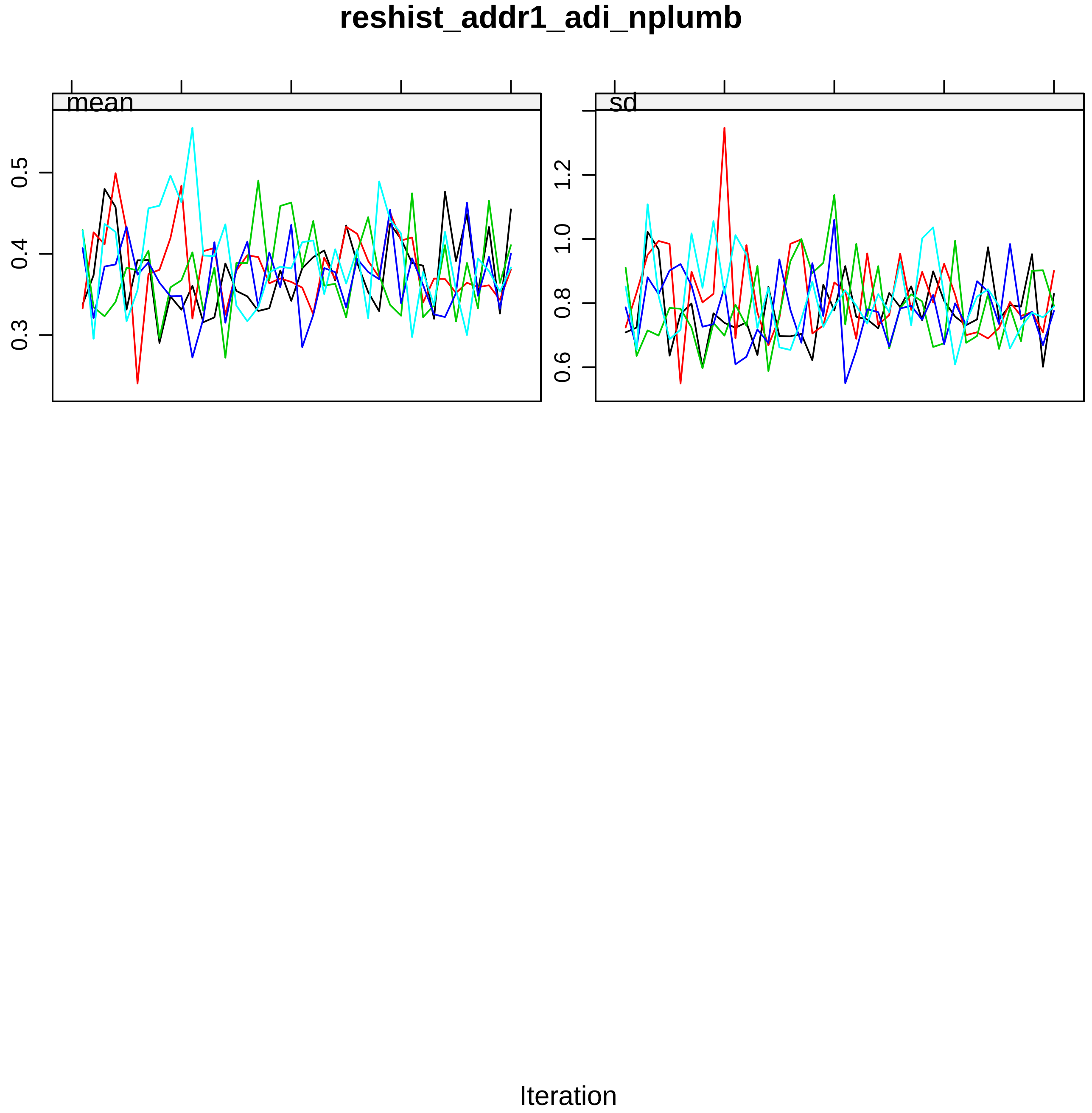
reshist_addr1_adi_nplumb
mean
sd
0.3
0.4
0.5
0.6
0.8
1.0
1.2
Iteration

reshist_addr1_adi_perc

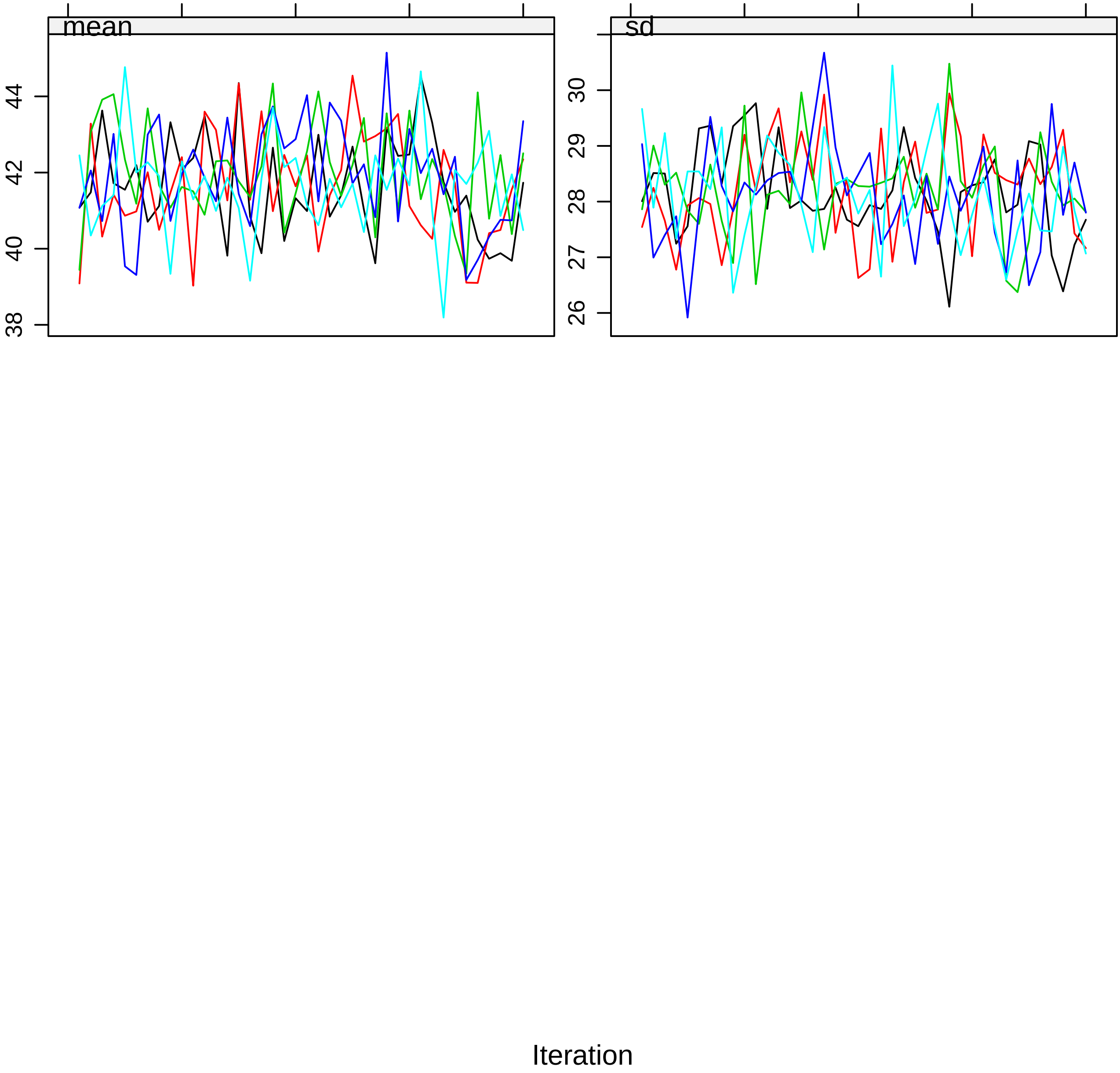

reshist_addr1_no2

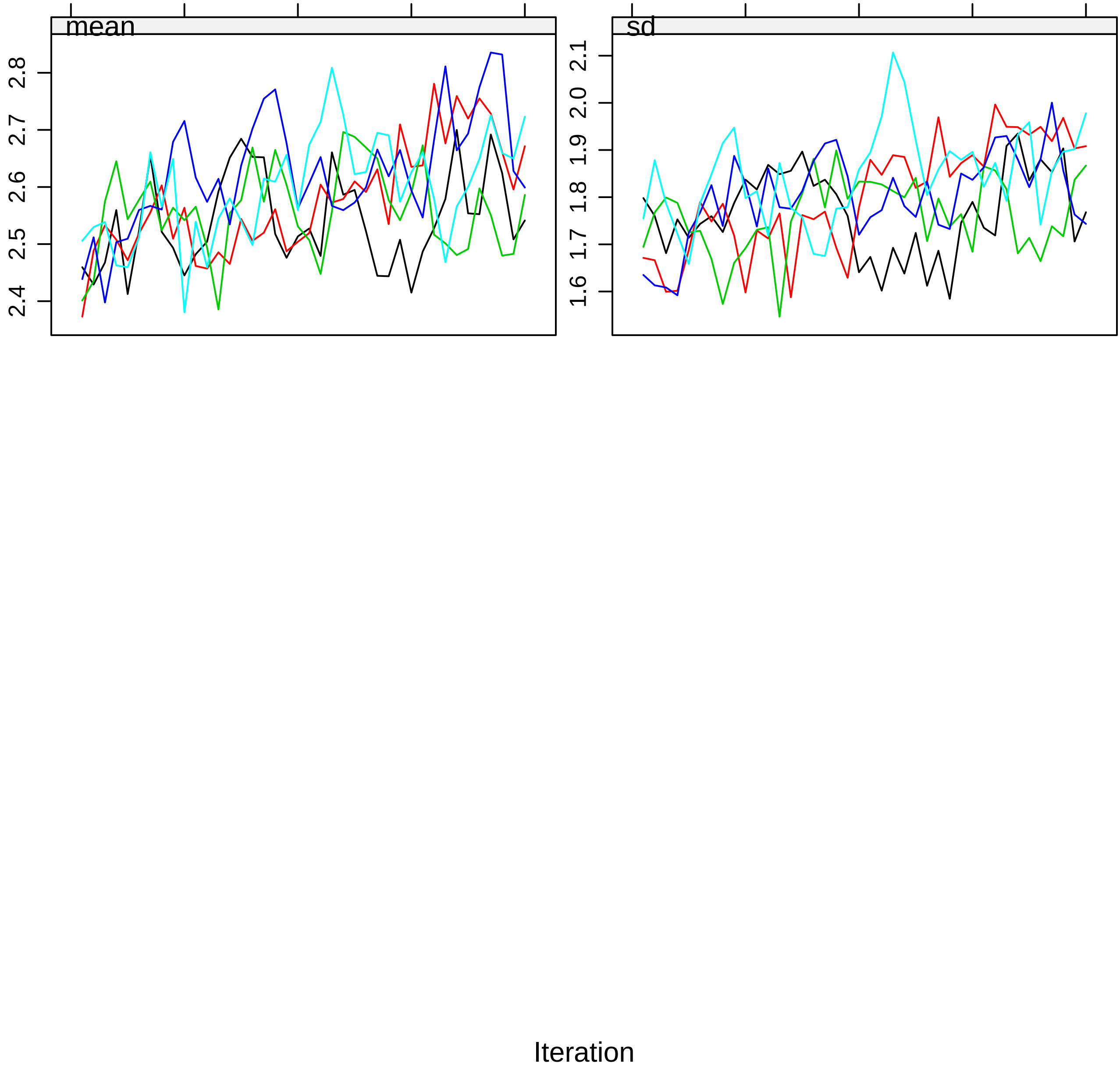

reshist_addr1_pm25

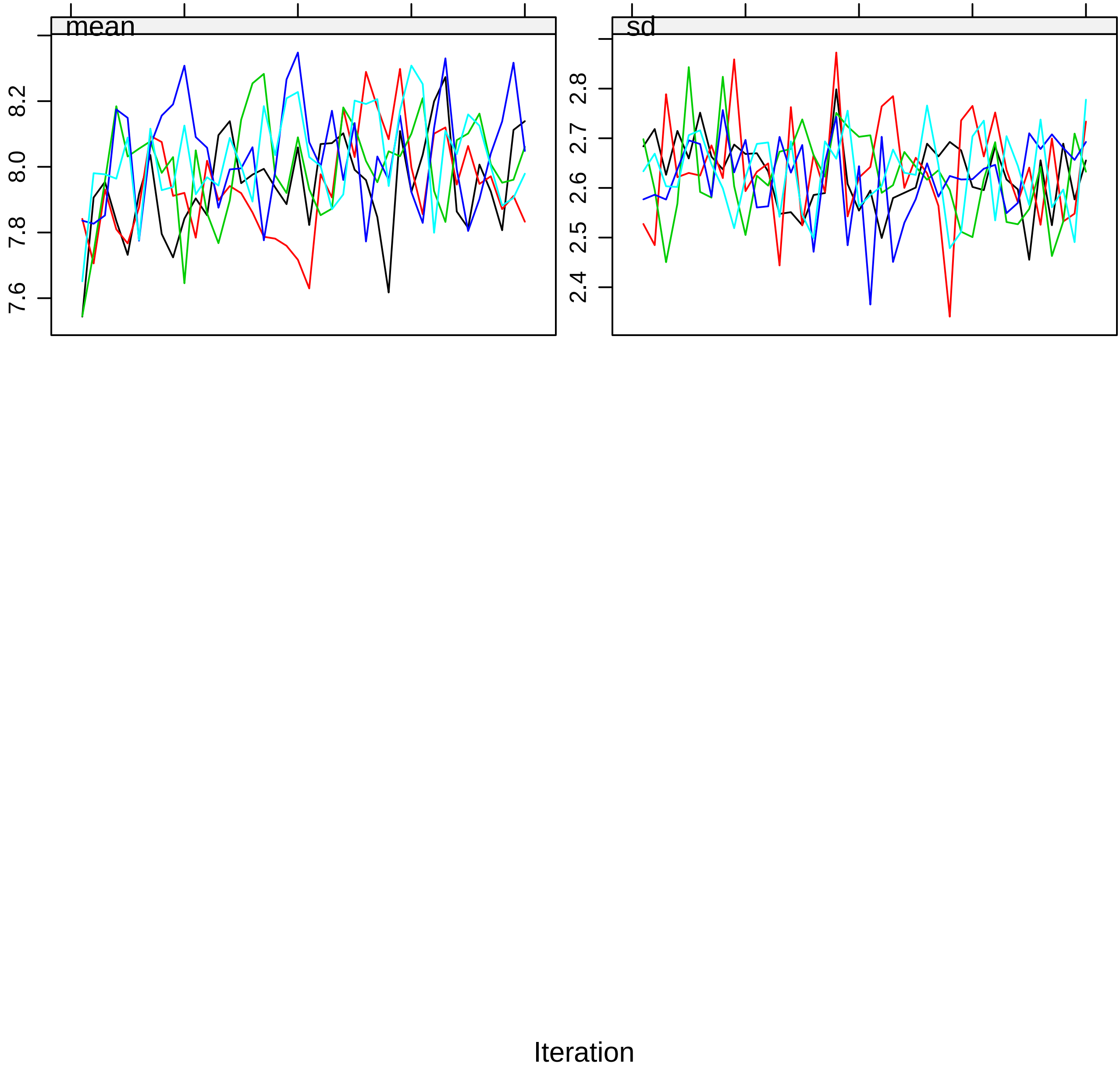

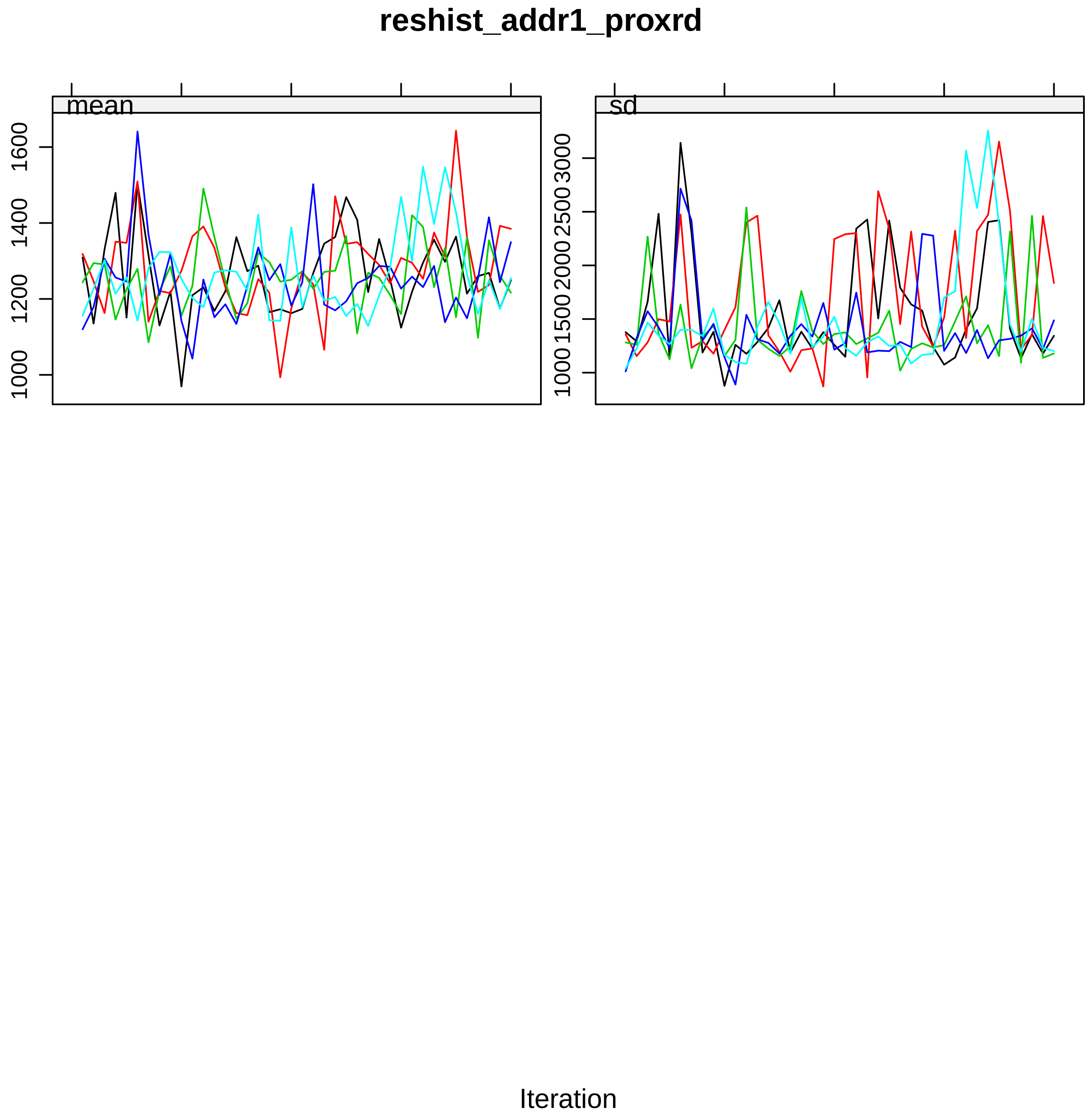
reshist_addr1_proxrd
mean
sd
1000
1200
1400
1600
1000
1500
2000
2500
3000
Iteration

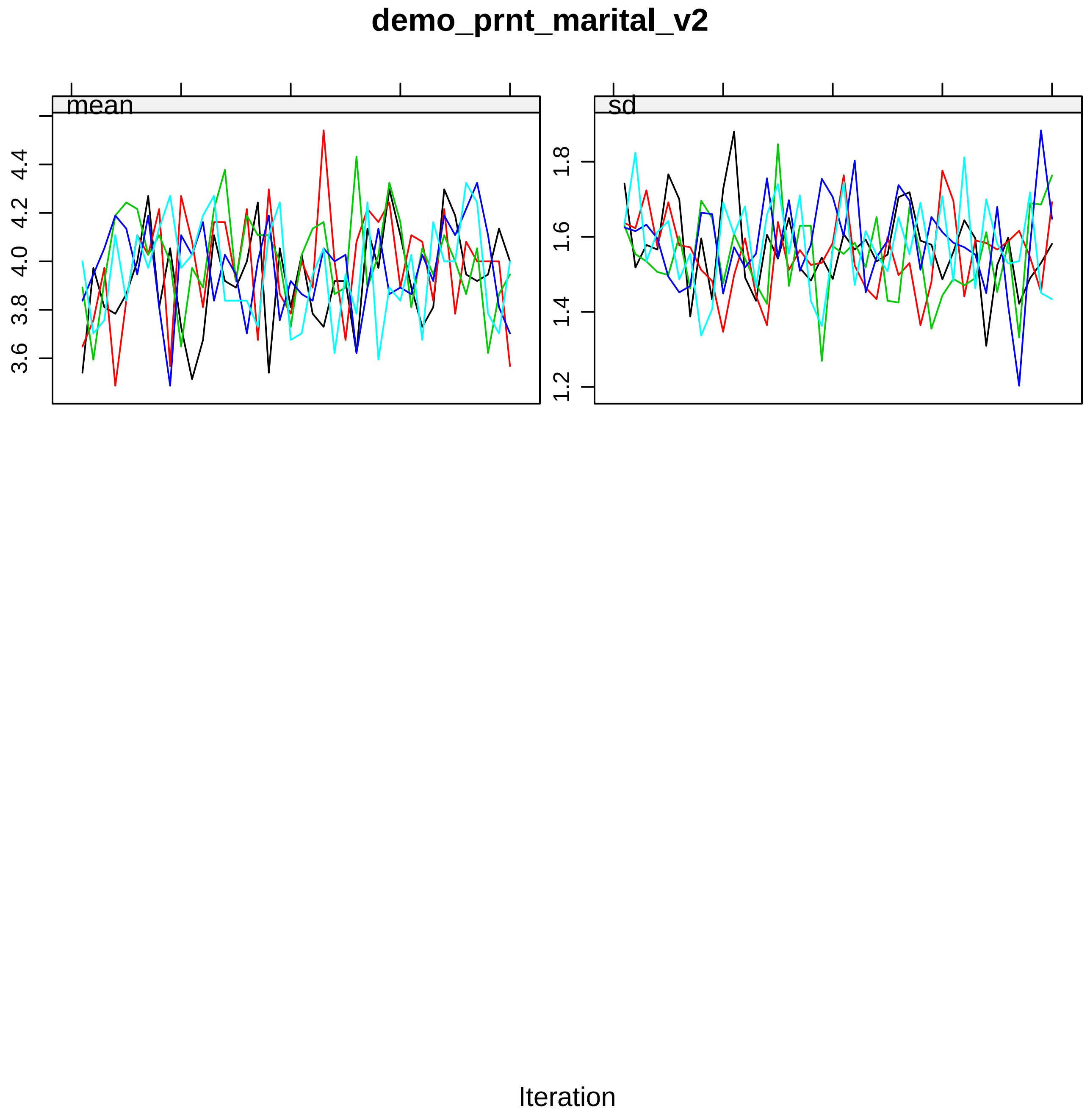

demo_prnt_marital_v2
mean
sd
3.6
3.8
4.0
4.2
4.4
1.2
1.4
1.6
1.8
Iteration

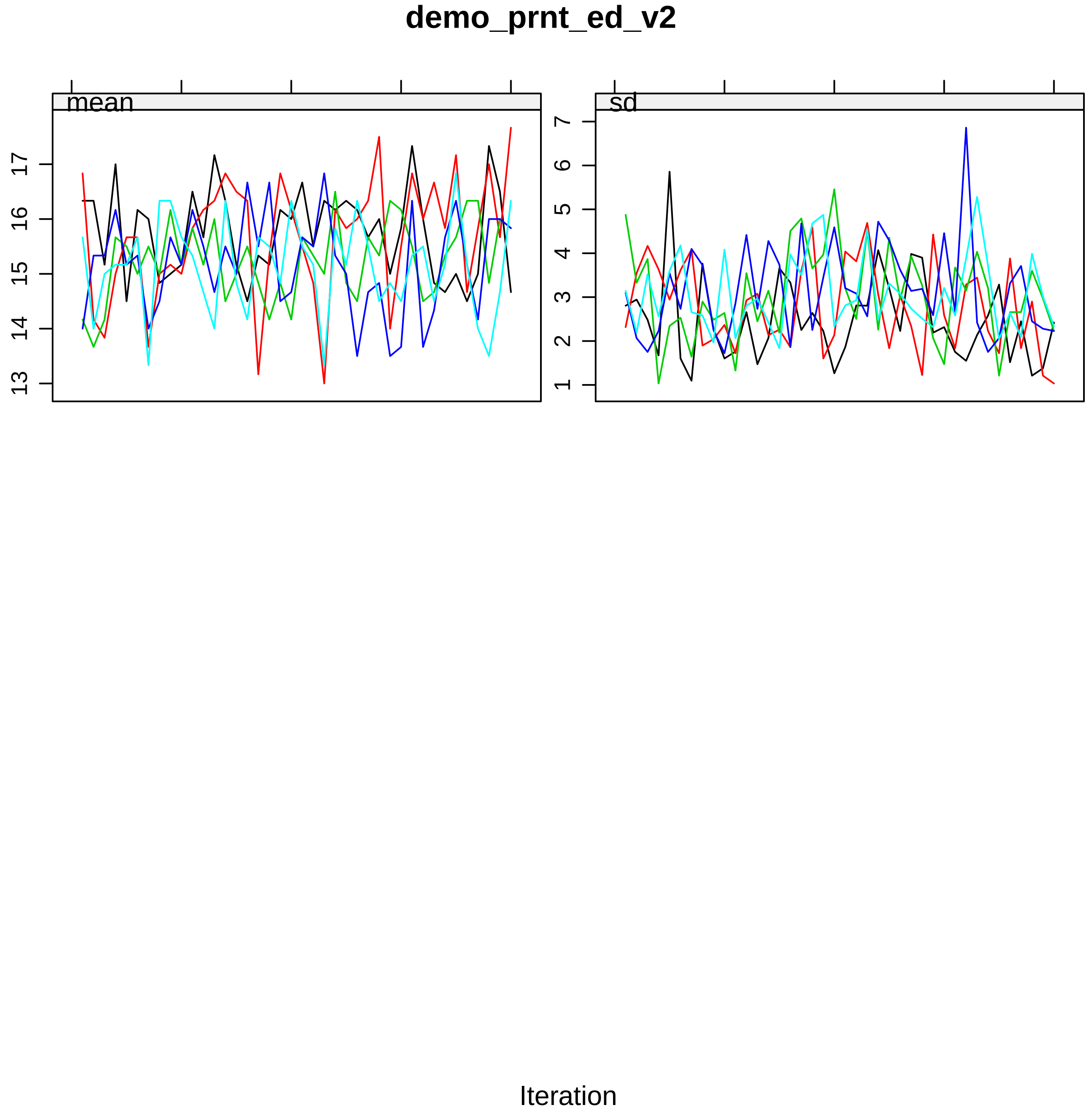
demo_prnt_ed_v2
mean
sd
13
14
15
16
17
1
2
3
4
5
6
7
Iteration

demo_prnt_empl_v2

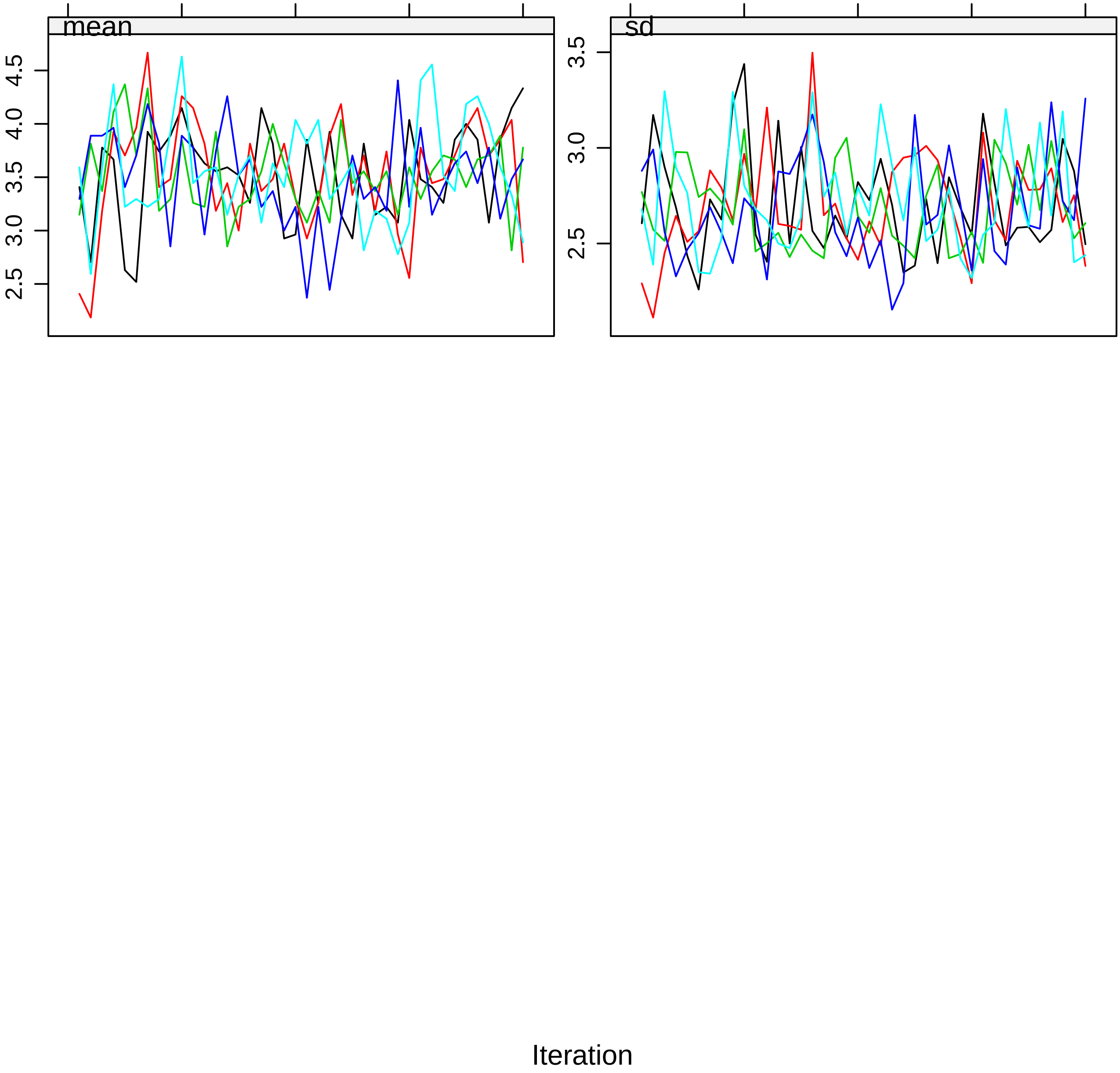

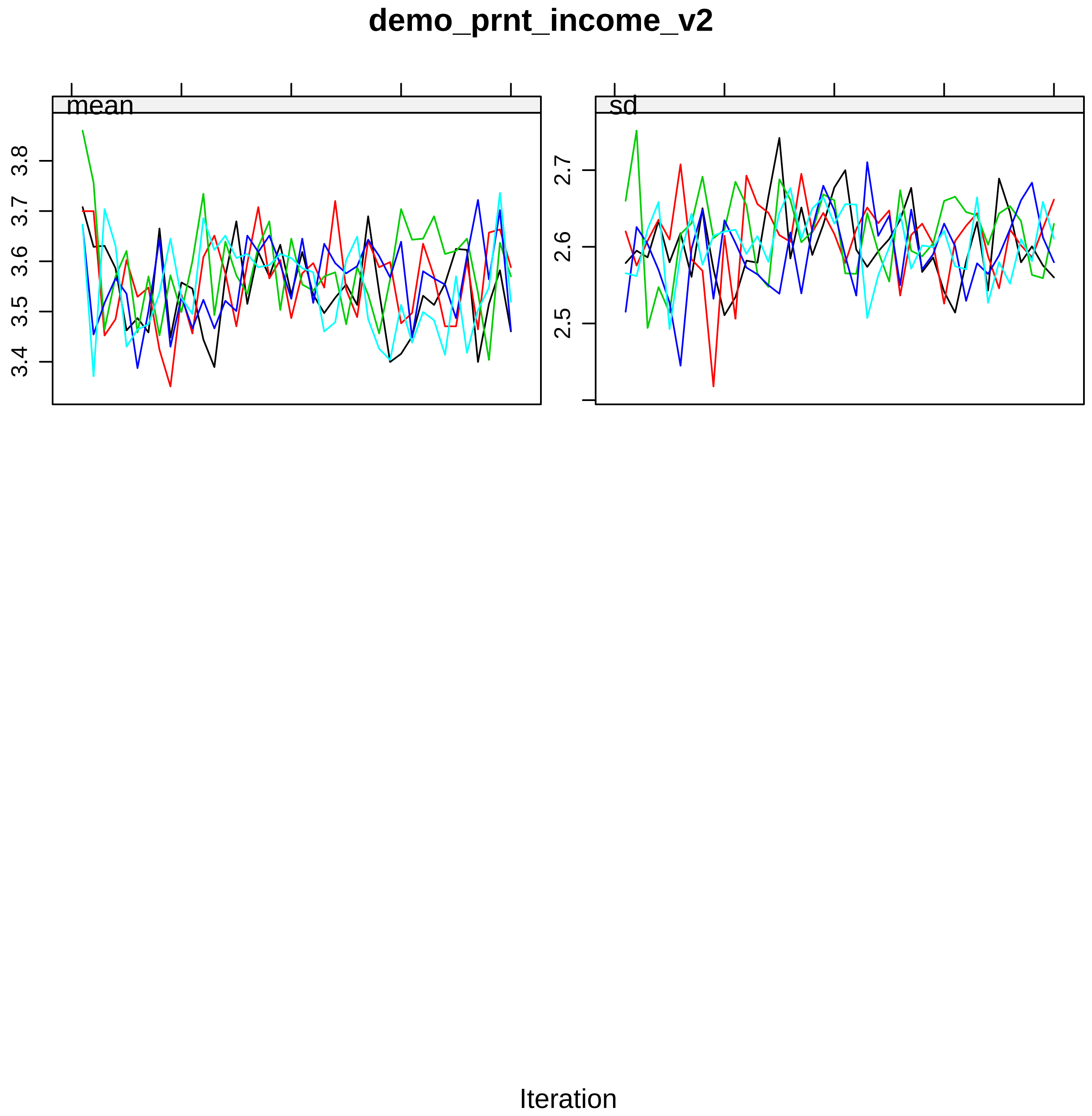
demo_prnt_income_v2
mean
sd
3.4
3.5
3.6
3.7
3.8
2.5
2.6
2.7
Iteration

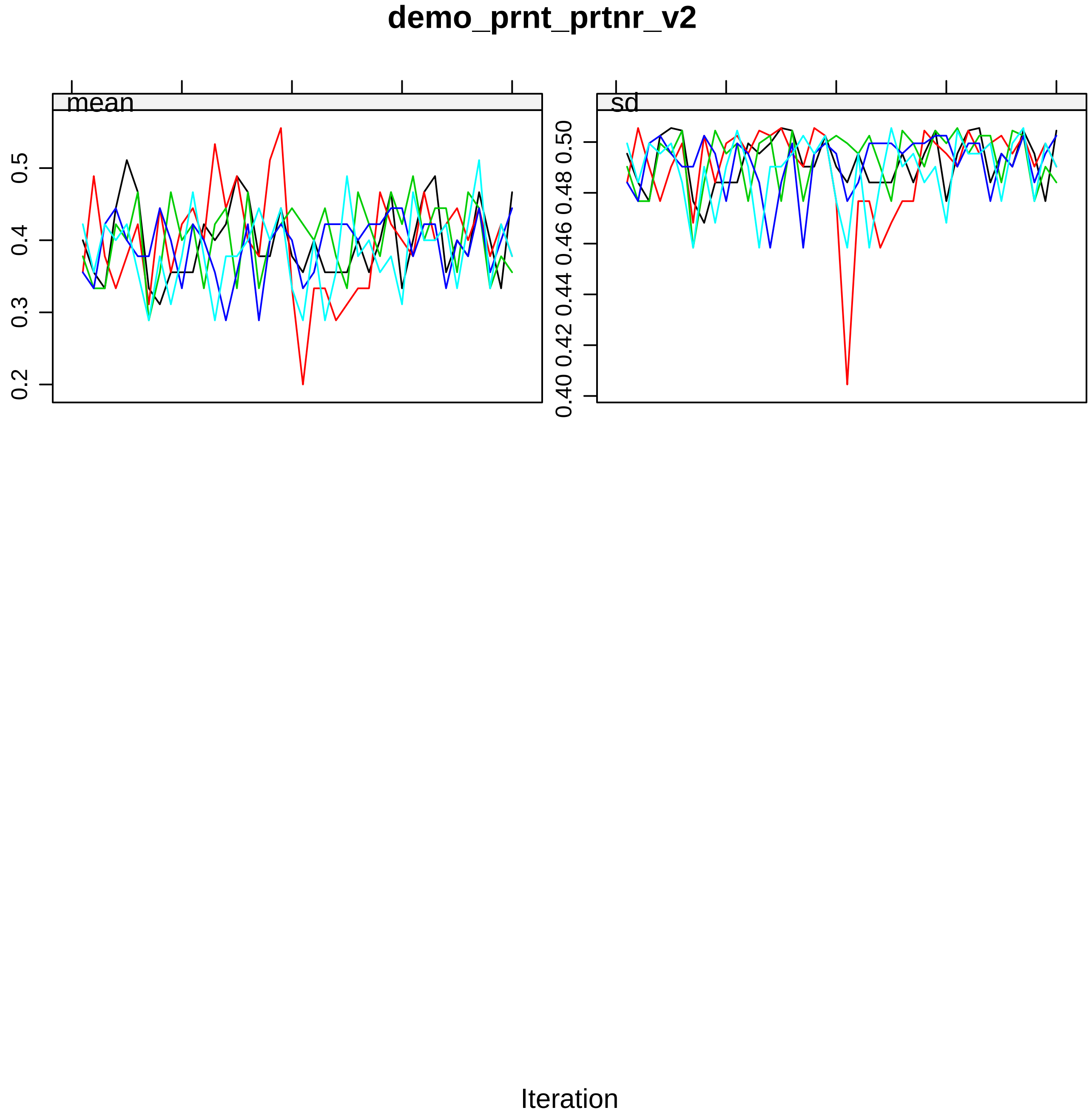

demo_prnt_prtnr_v2
mean
sd
0.2
0.3
0.4
0.5
0.40
0.42
0.44
0.46
0.48
0.50
Iteration

demo_comb_income_v2

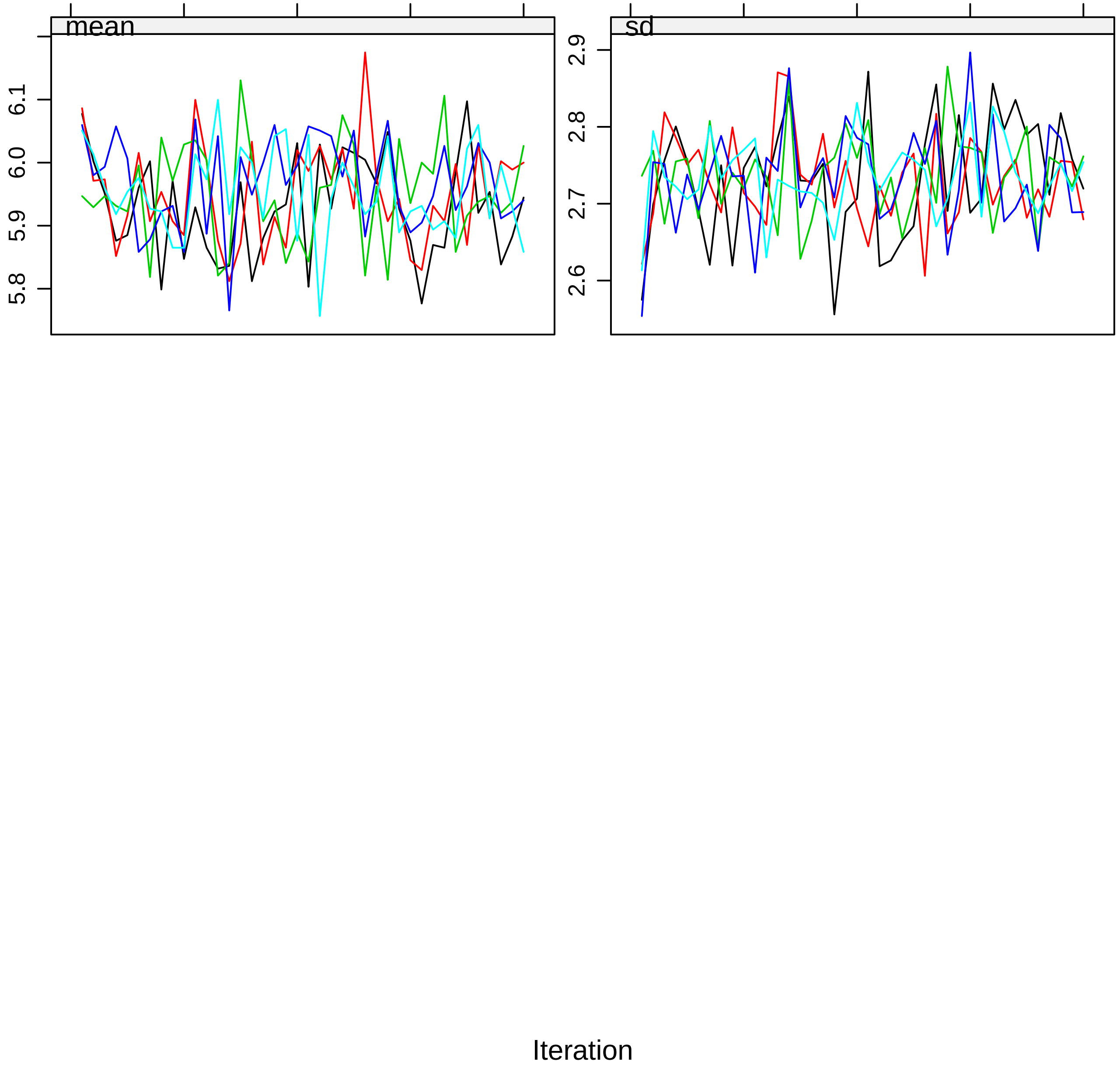

demo_fam_exp1_v2

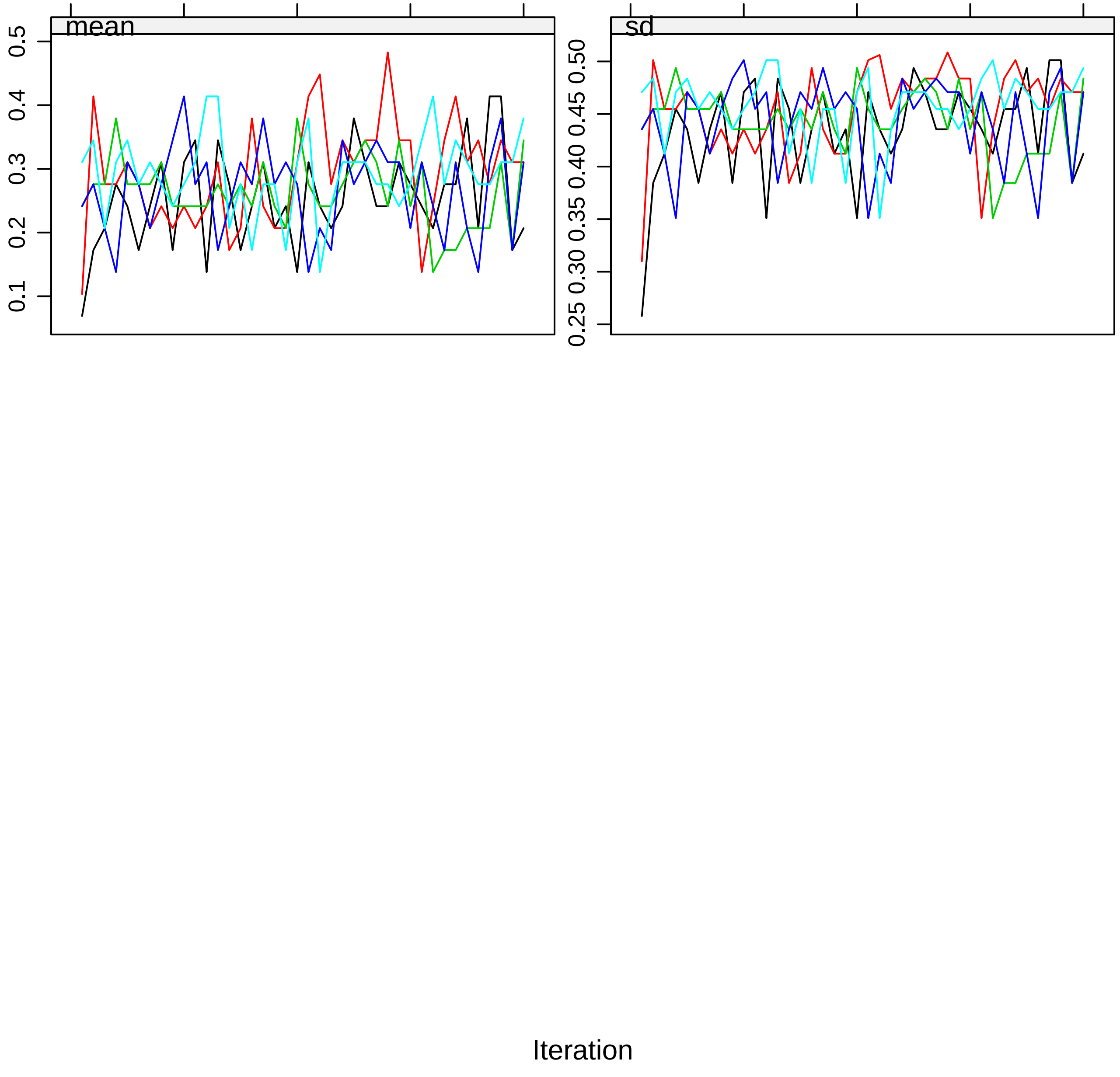

demo_fam_exp2_v2

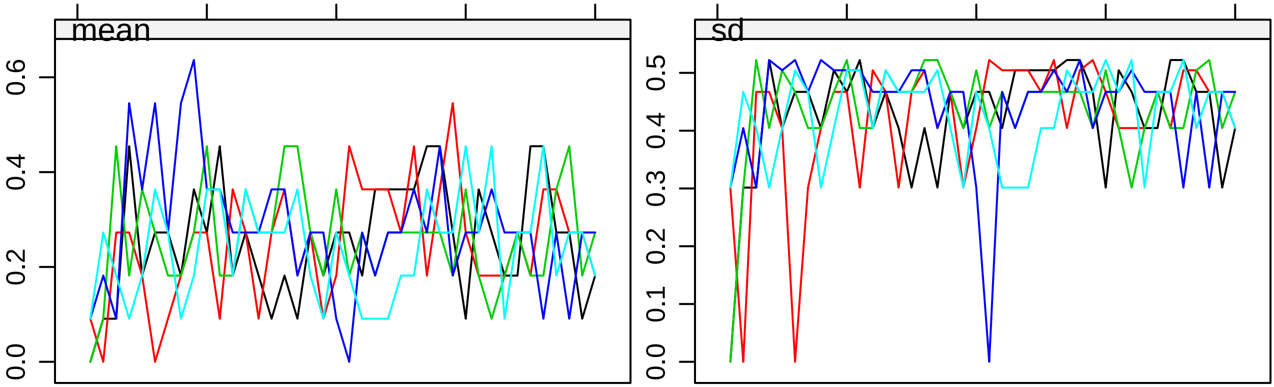

Iteration

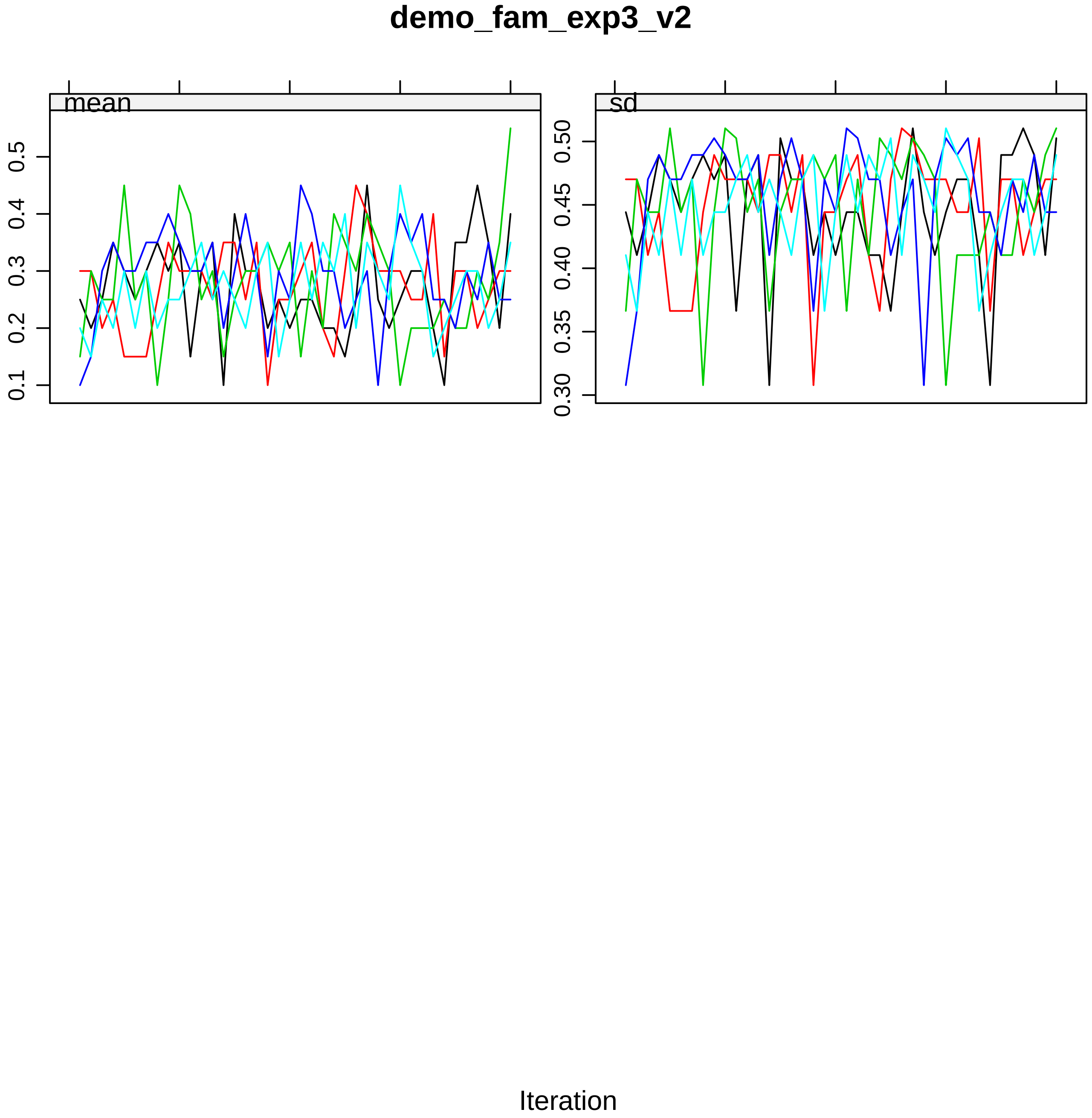
demo_fam_exp3_v2
mean
sd
0.1
0.2
0.3
0.4
0.5
0.30
0.35
0.40
0.45
0.50
Iteration

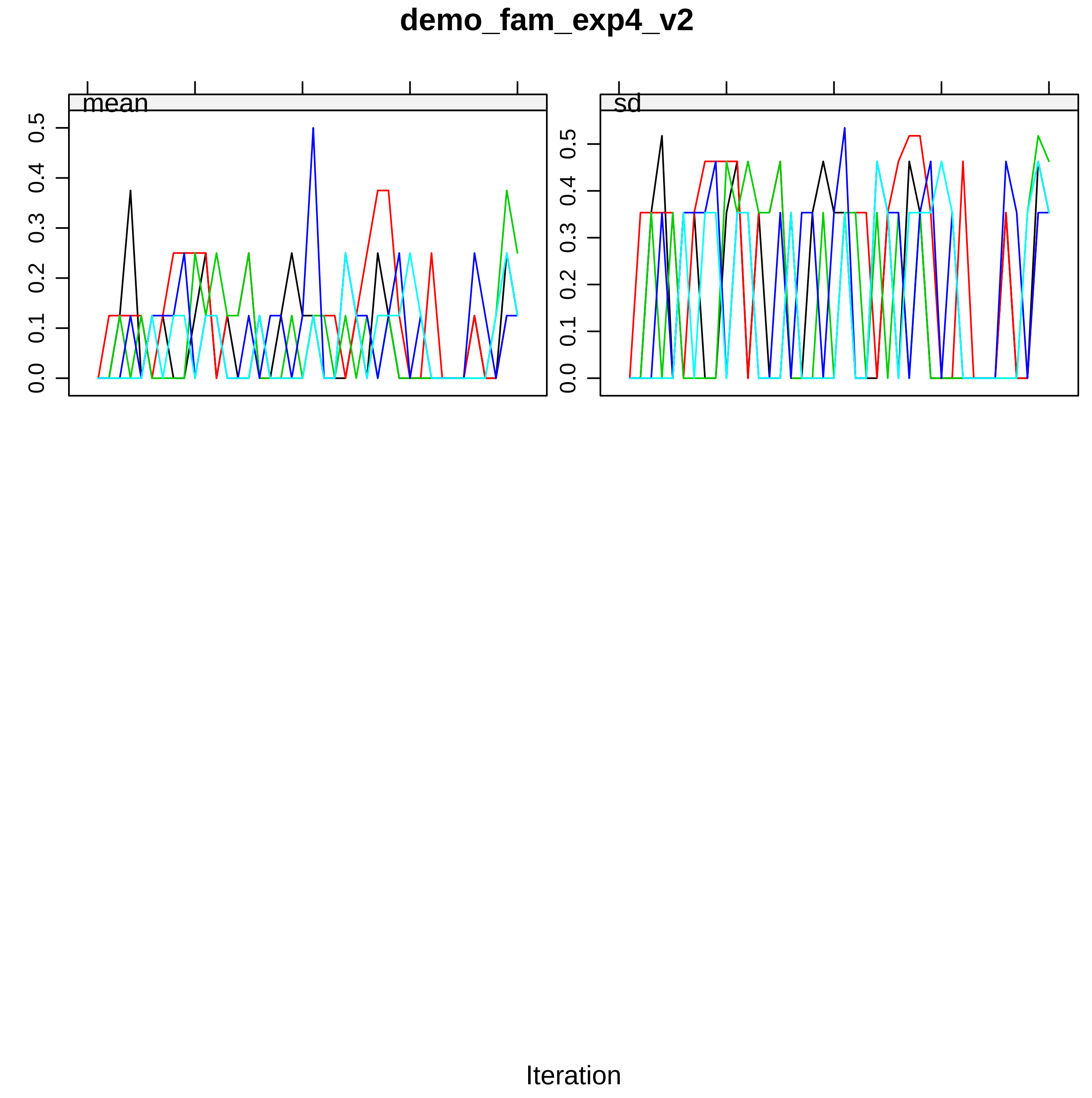
demo_fam_exp4_v2
mean
sd
0.0
0.1
0.2
0.3
0.4
0.5
Iteration

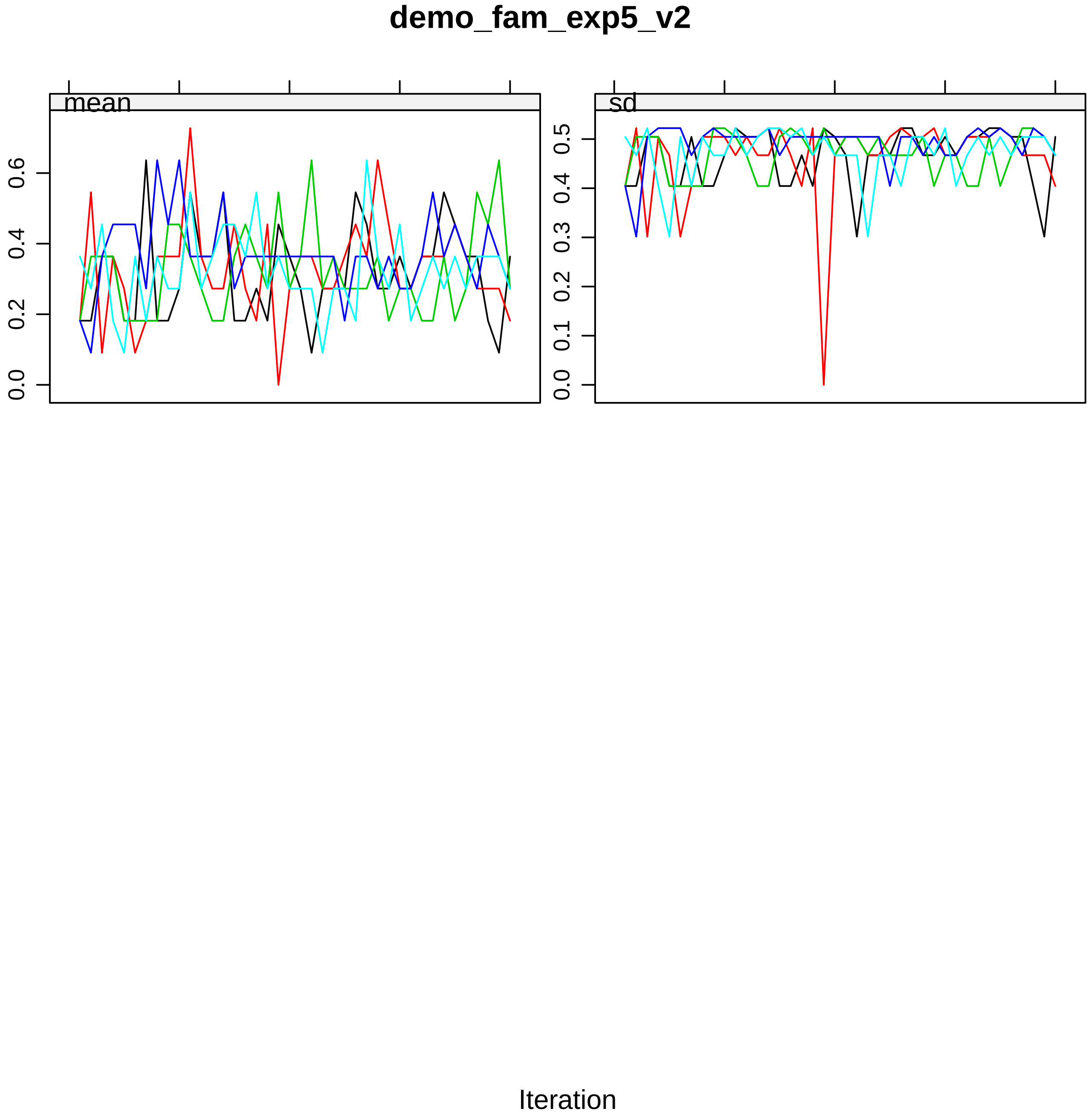
demo_fam_exp5_v2
mean
sd
0.0
0.2
0.4
0.6
0.0
0.1
0.2
0.3
0.4
0.5
Iteration

demo_fam_exp6_v2

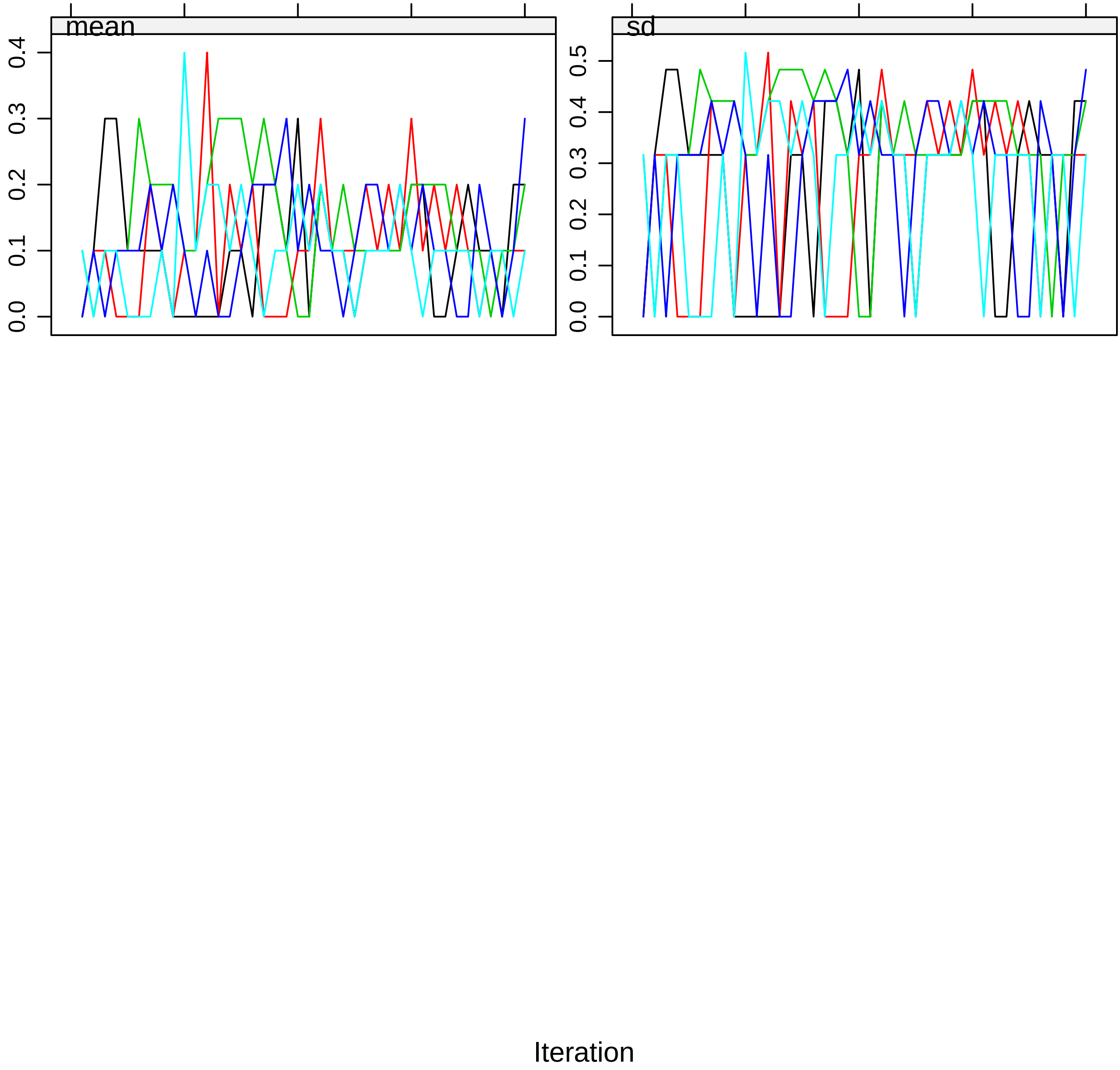

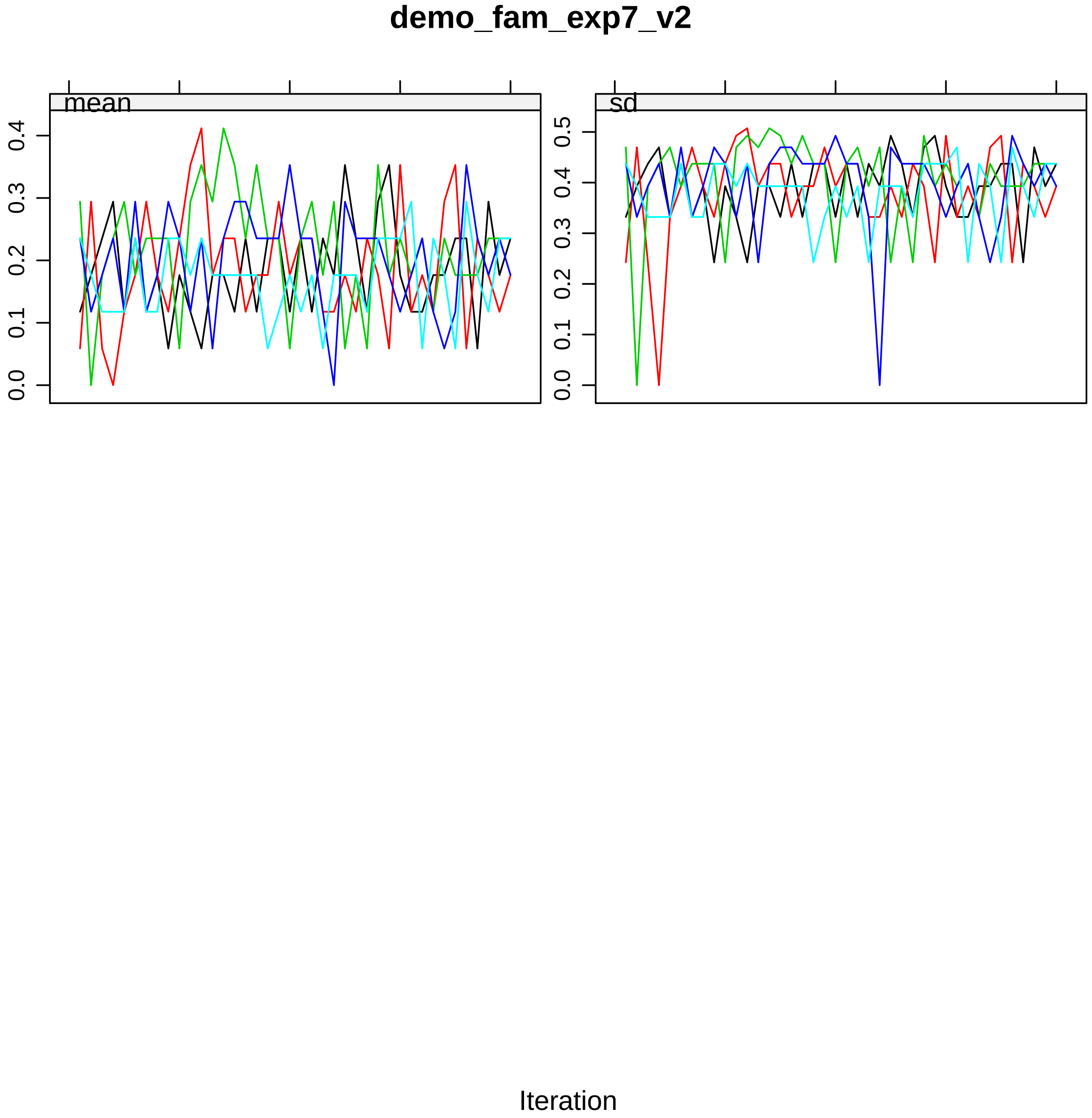
demo_fam_exp7_v2
mean
sd
0.0
0.1
0.2
0.3
0.4
0.5
Iteration

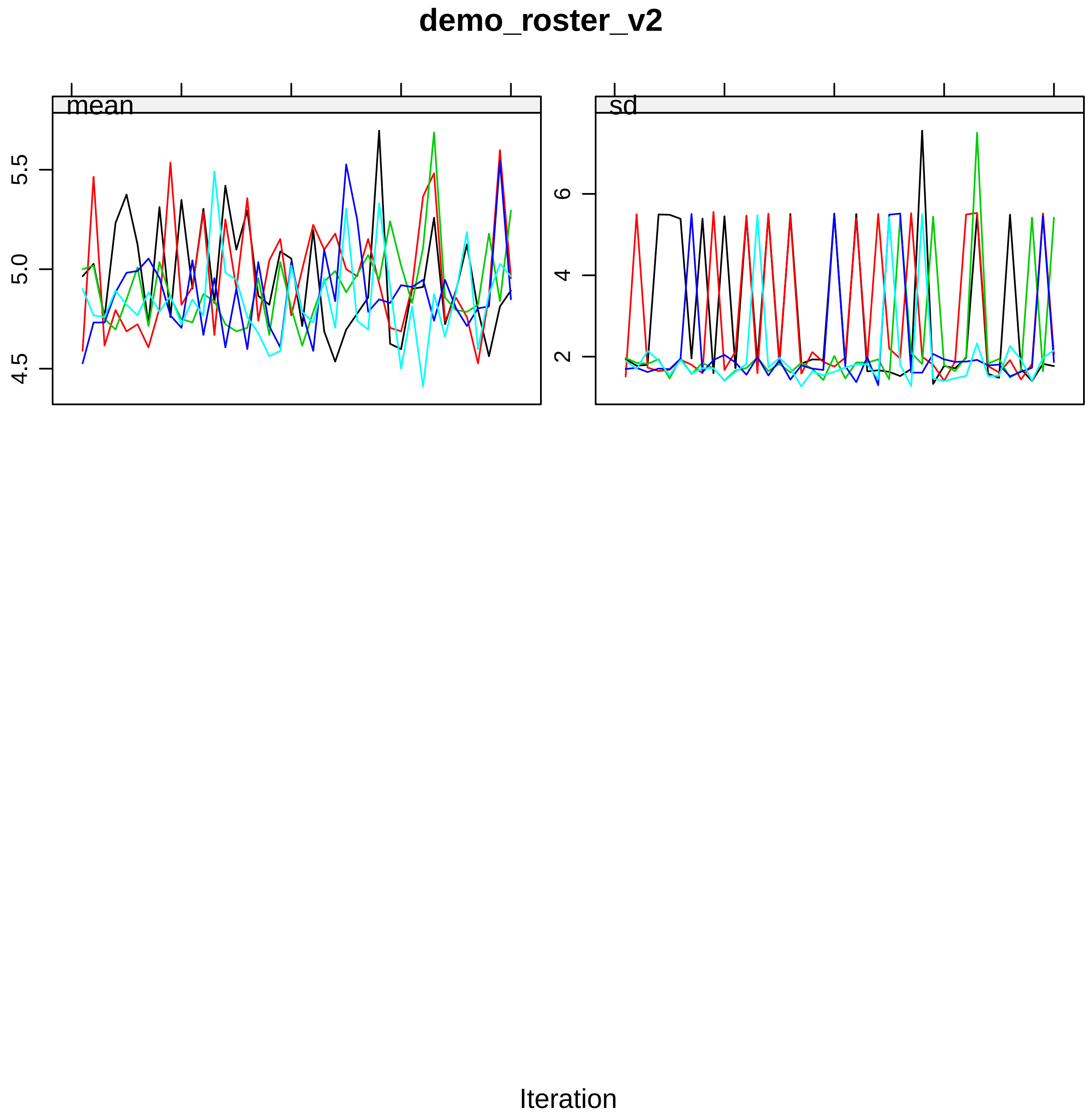
demo_roster_v2
mean
sd
5.5
5.0
4.5
6
4
2
Iteration

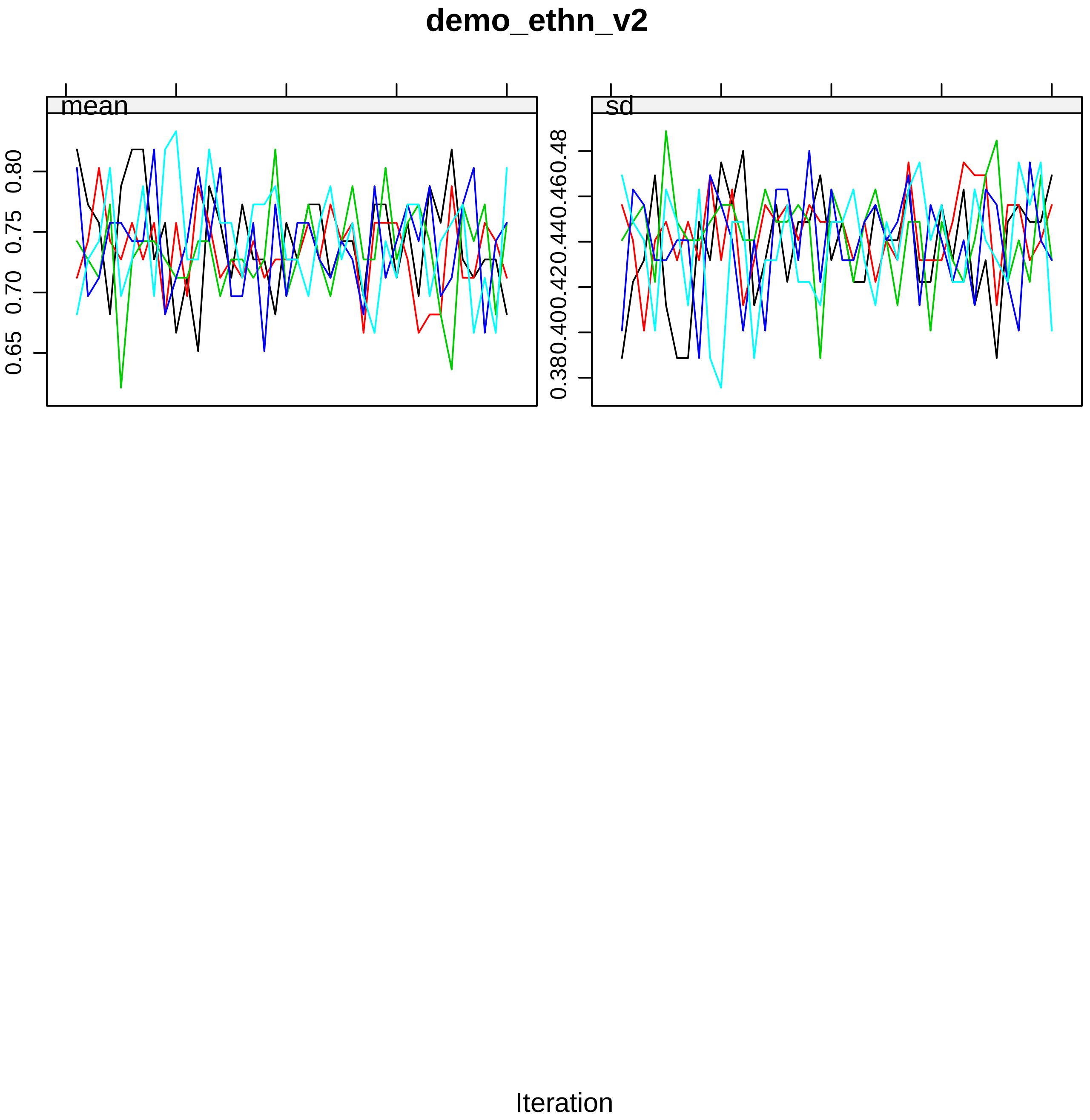
demo_ethn_v2
mean
sd
0.65
0.70
0.75
0.80
0.38
0.40
0.42
0.44
0.46
0.48
Iteration

physical_activity1_y

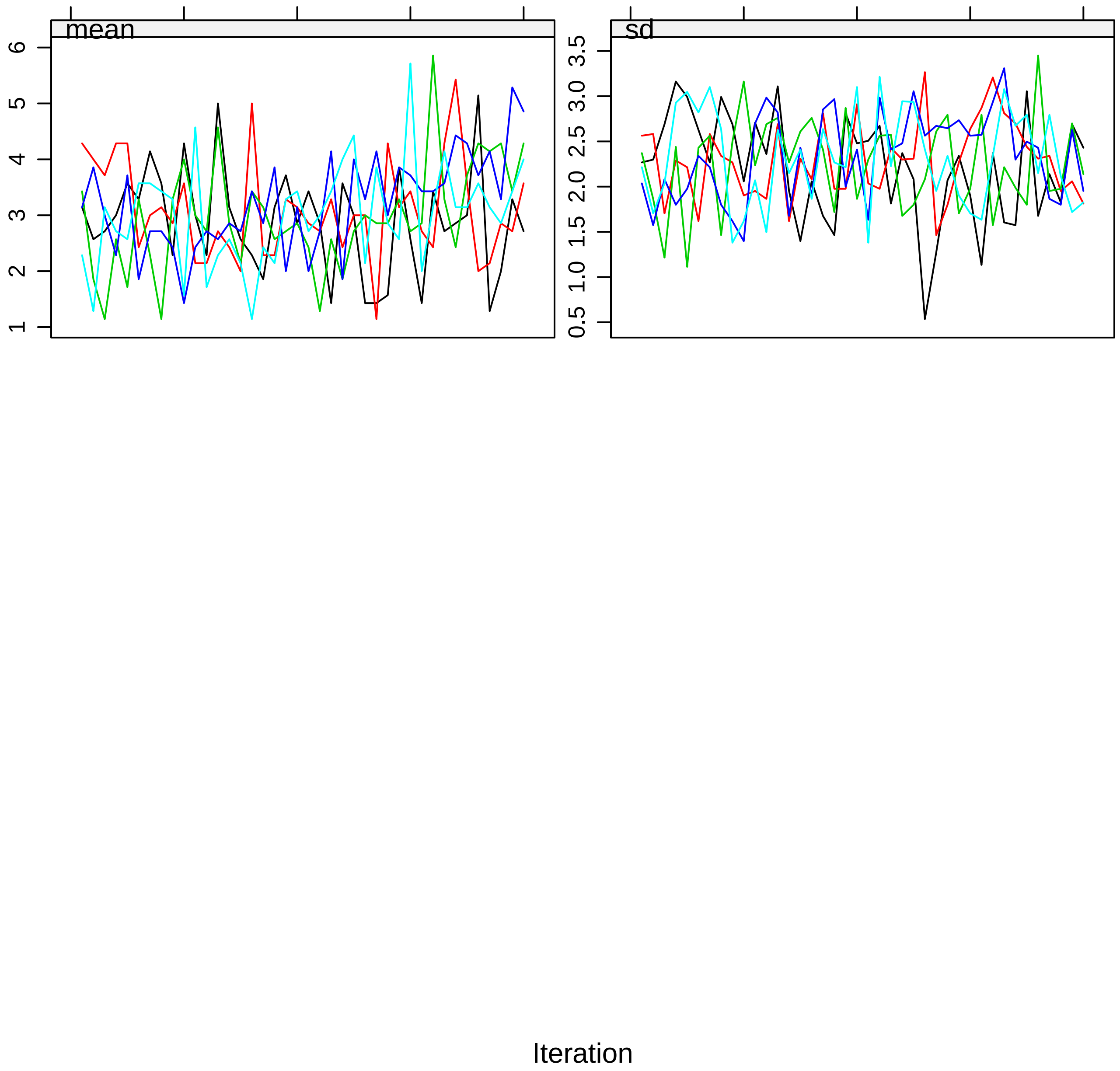

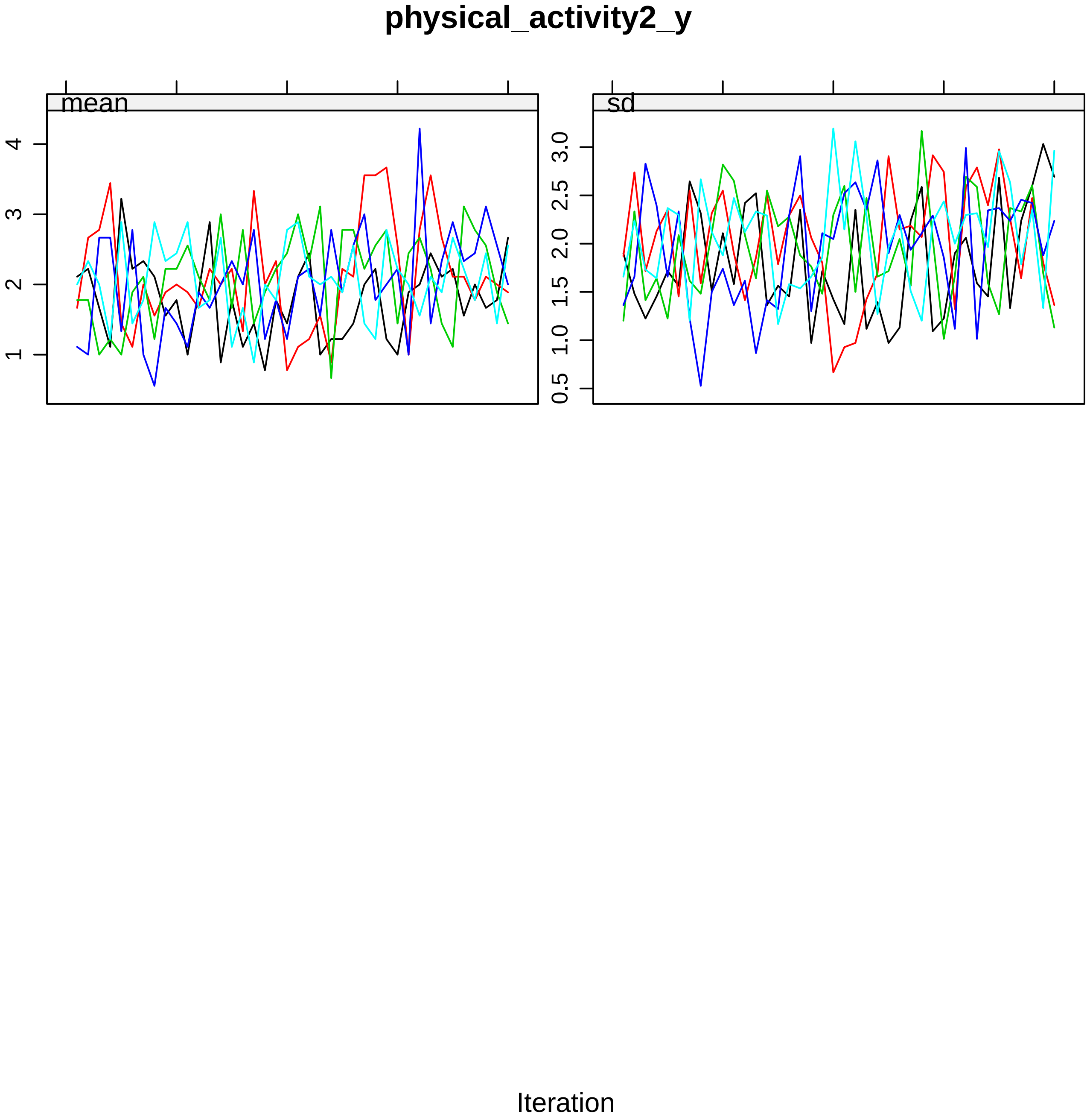

physical_activity2_y
mean
sd
1
2
3
4
0.5
1.0
1.5
2.0
2.5
3.0
Iteration

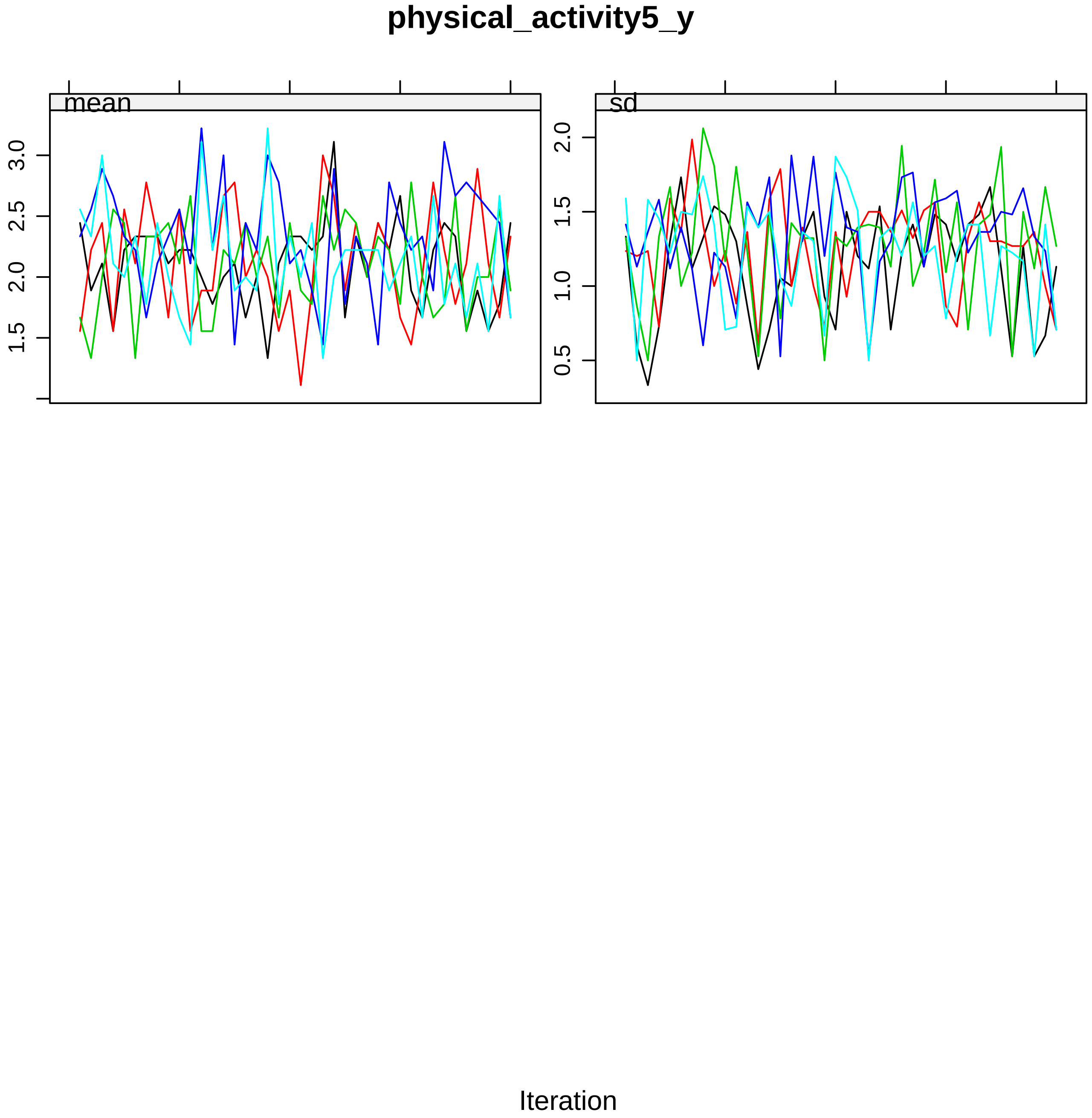

physical_activity5_y
mean
sd
1.5
2.0
2.5
3.0
0.5
1.0
1.5
2.0
Iteration

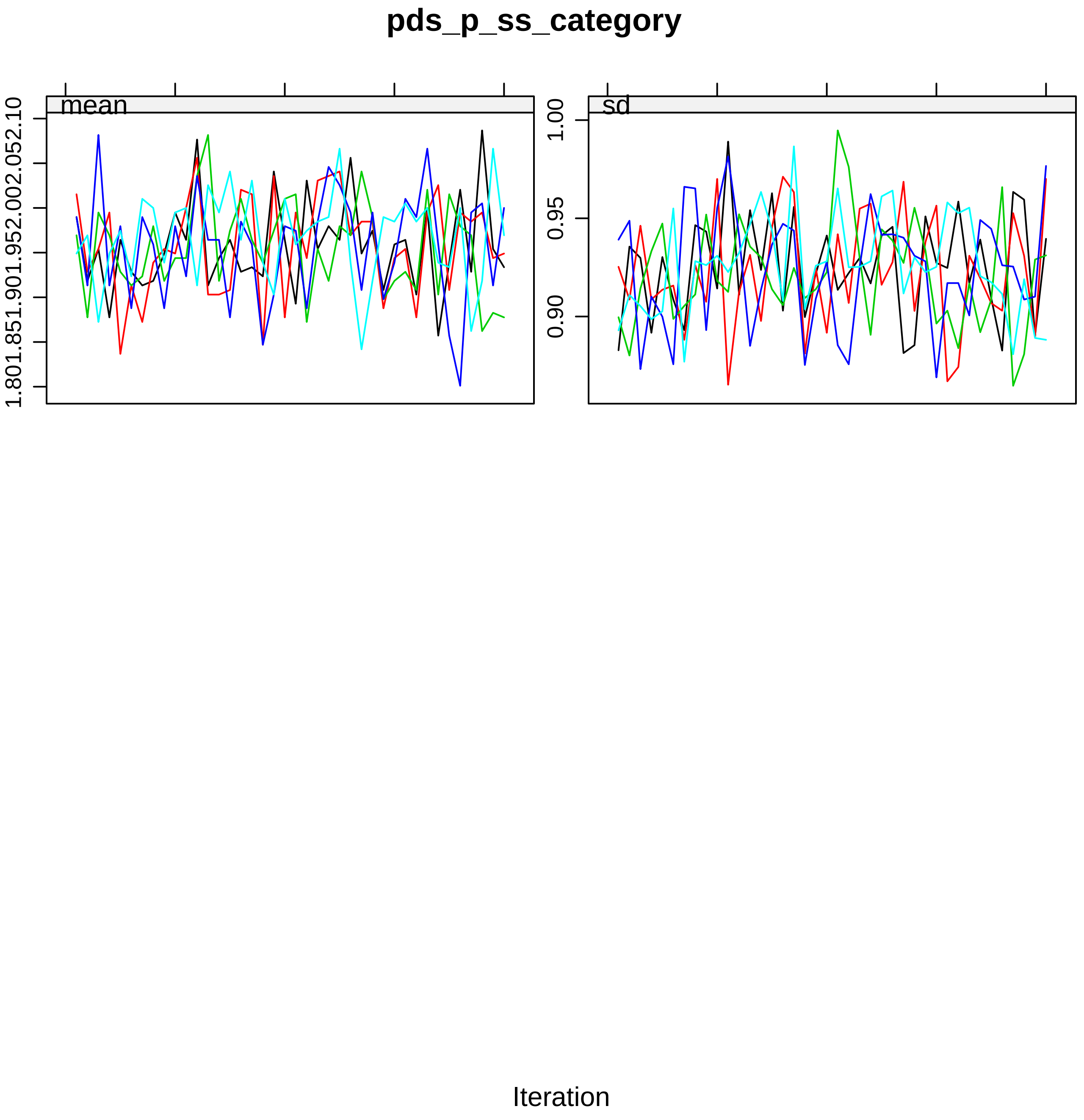
pds_p_ss_category
mean
sd
1.80
1.85
1.90
1.95
2.00
2.05
2.10
0.90
0.95
1.00
Iteration

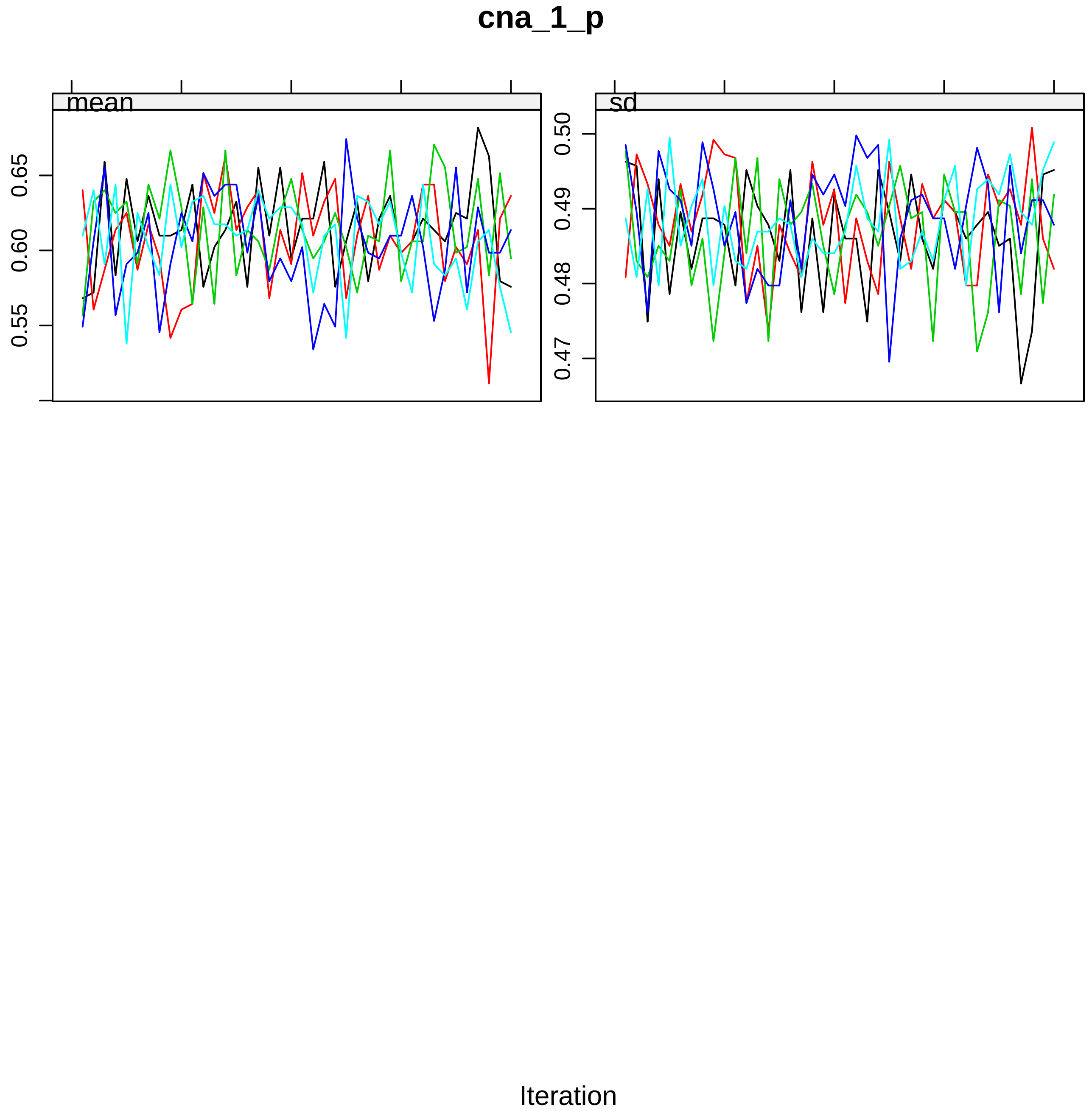
cna_1_p
mean
sd
0.55
0.60
0.65
0.47
0.48
0.49
0.50
Iteration

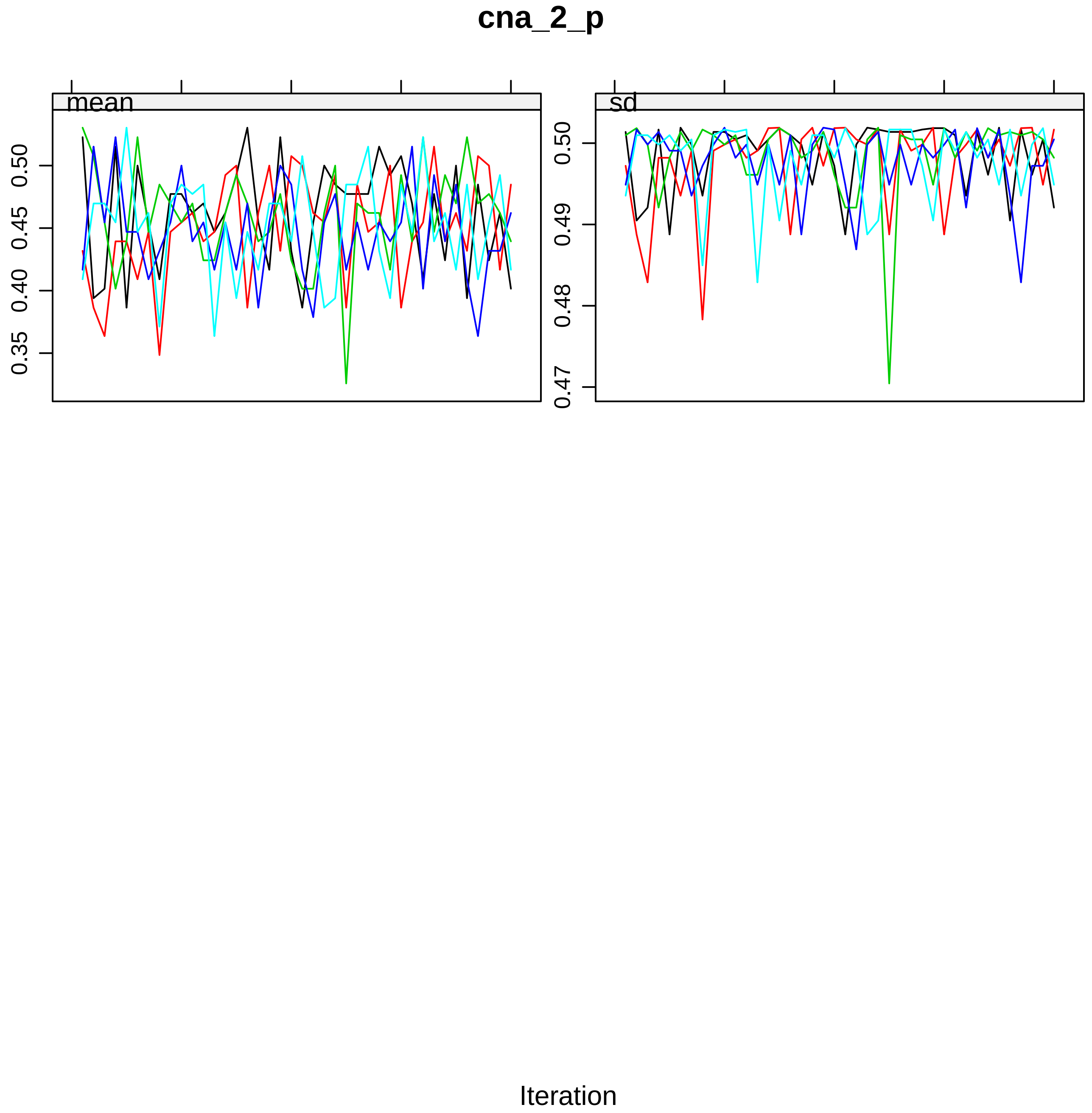
cna_2_p
mean
sd
0.35
0.40
0.45
0.50
0.47
0.48
0.49
0.50
Iteration

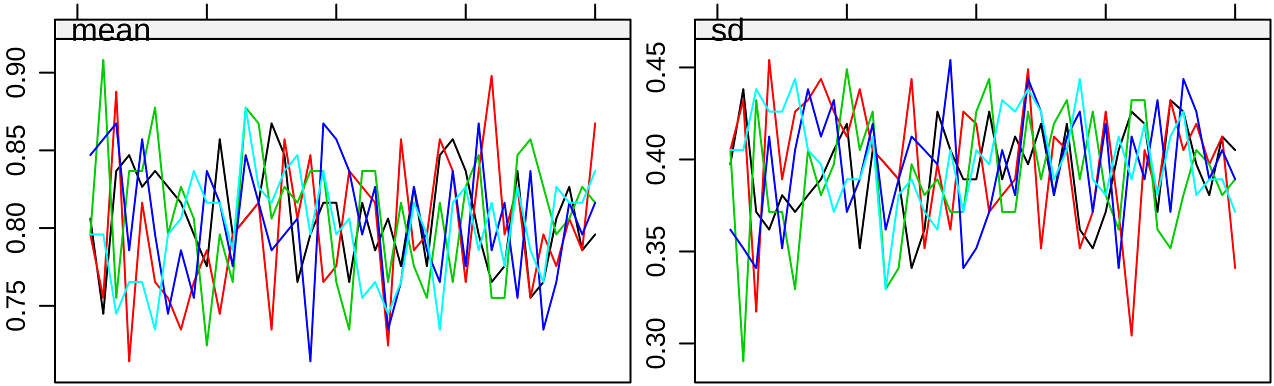

Iteration

cna_4_p

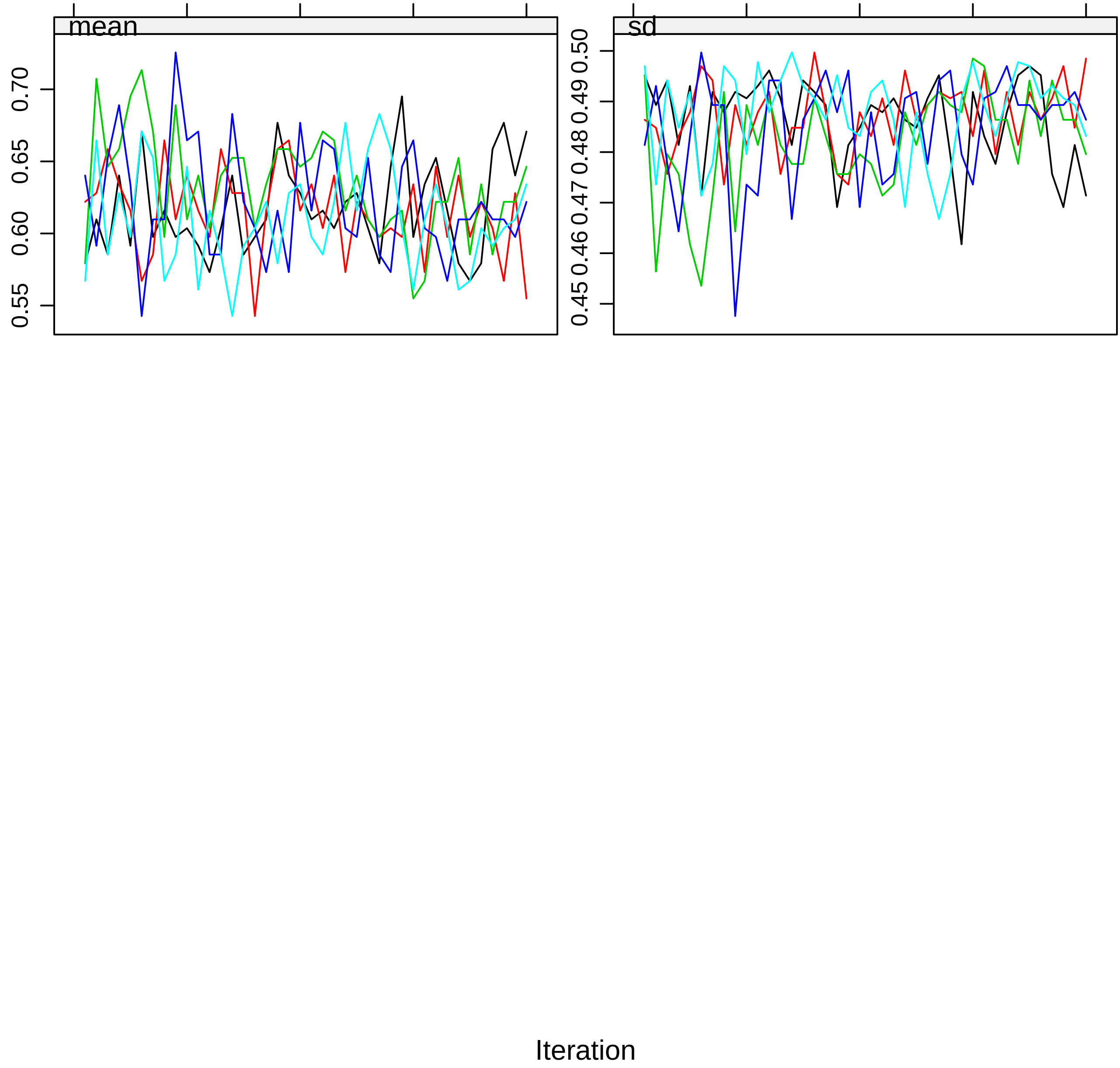

cna_5_p

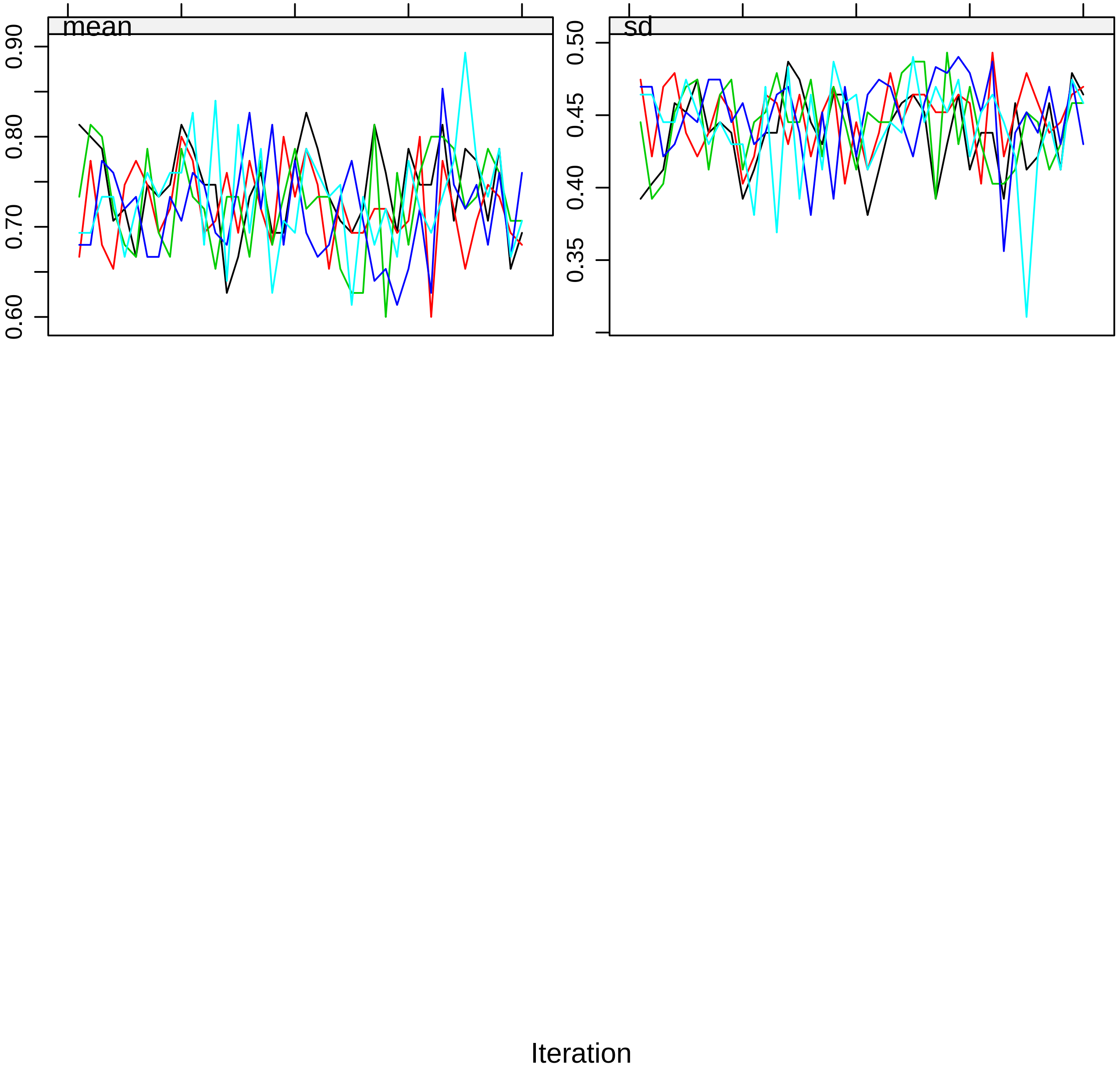

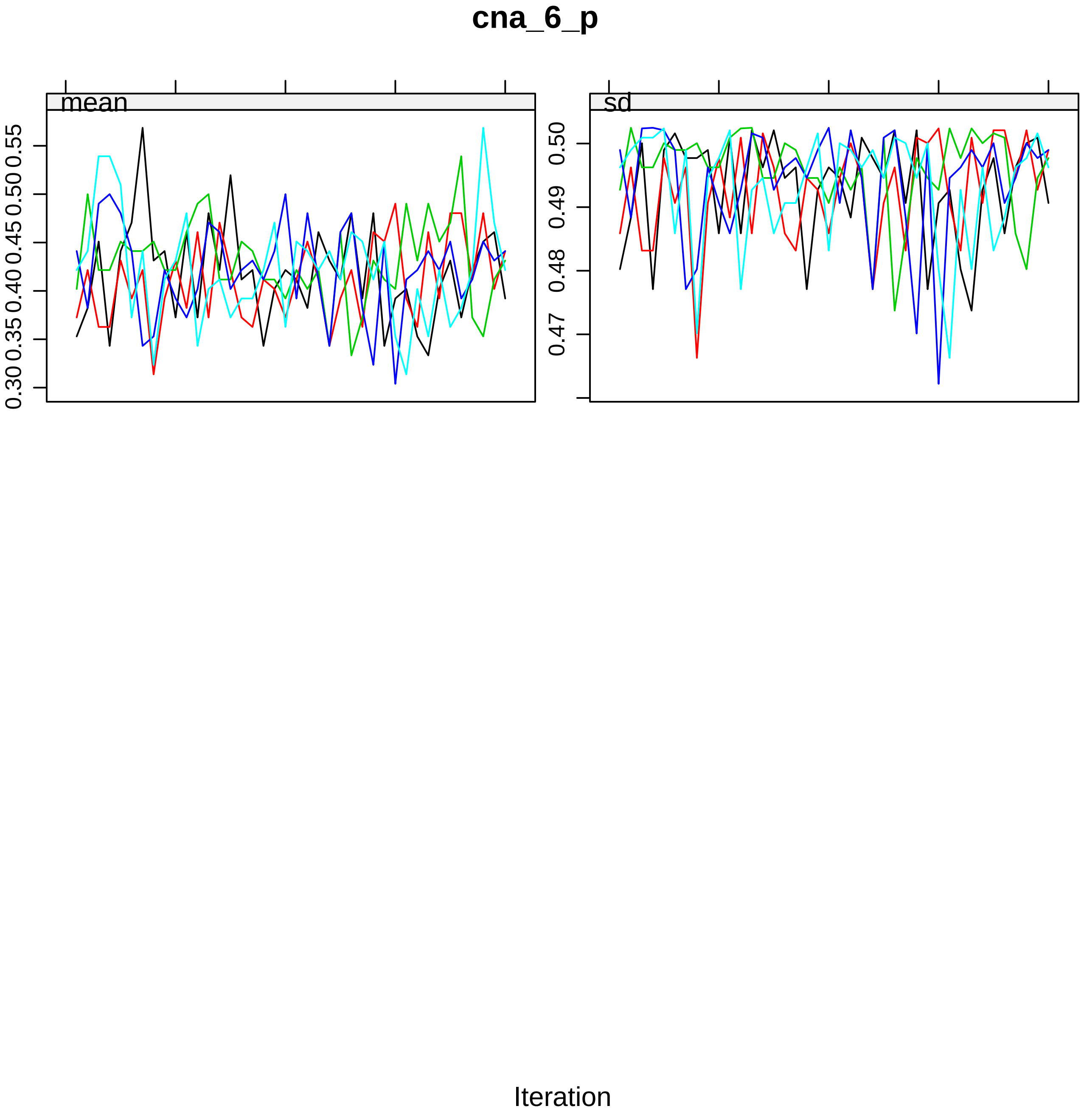
cna_6_p
mean
sd
0.30
0.35
0.40
0.45
0.50
0.55
0.47
0.48
0.49
0.50
Iteration

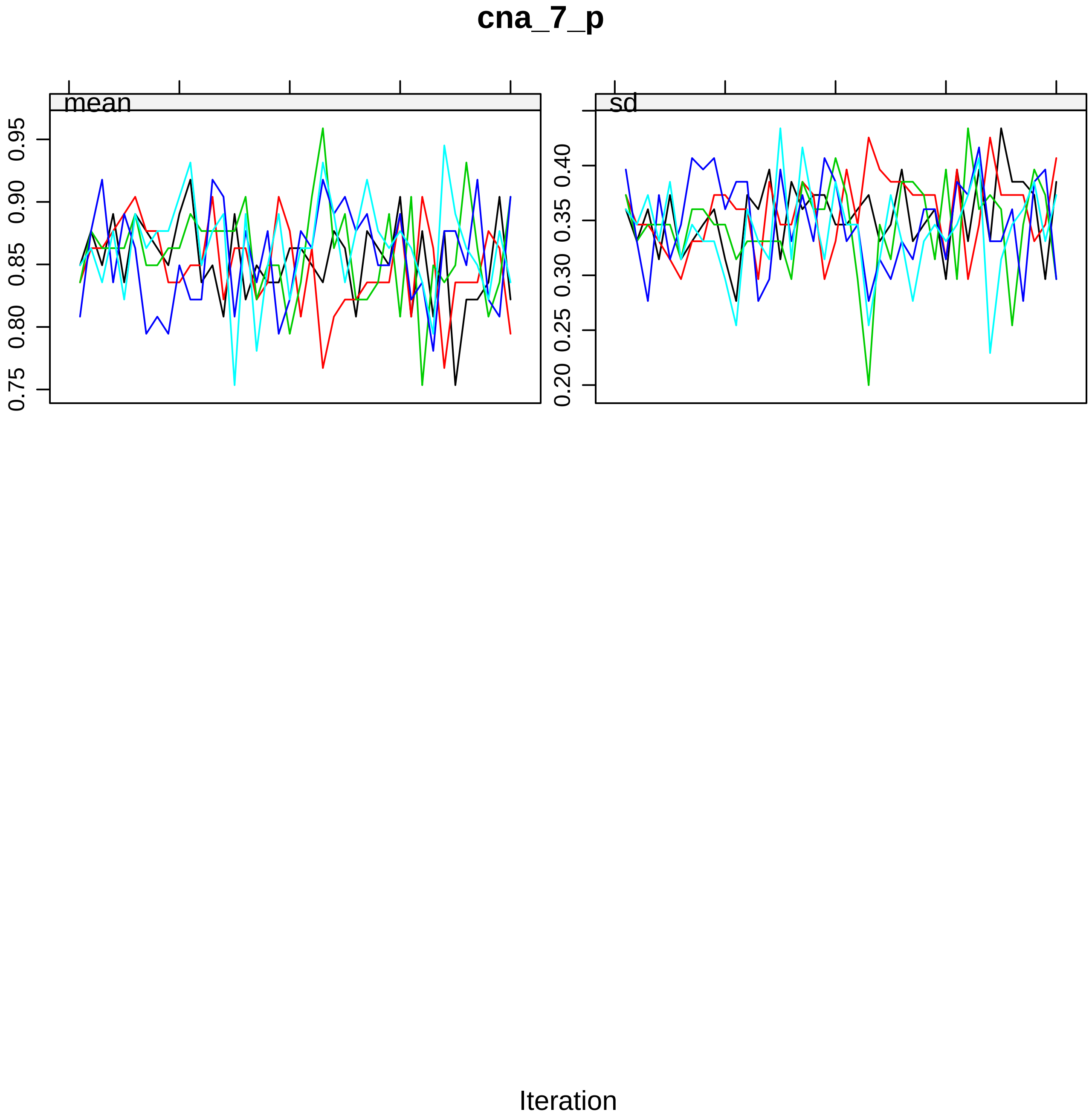
cna_7_p
mean
sd
0.75
0.80
0.85
0.90
0.95
0.20
0.25
0.30
0.35
0.40
Iteration

cna_8_p

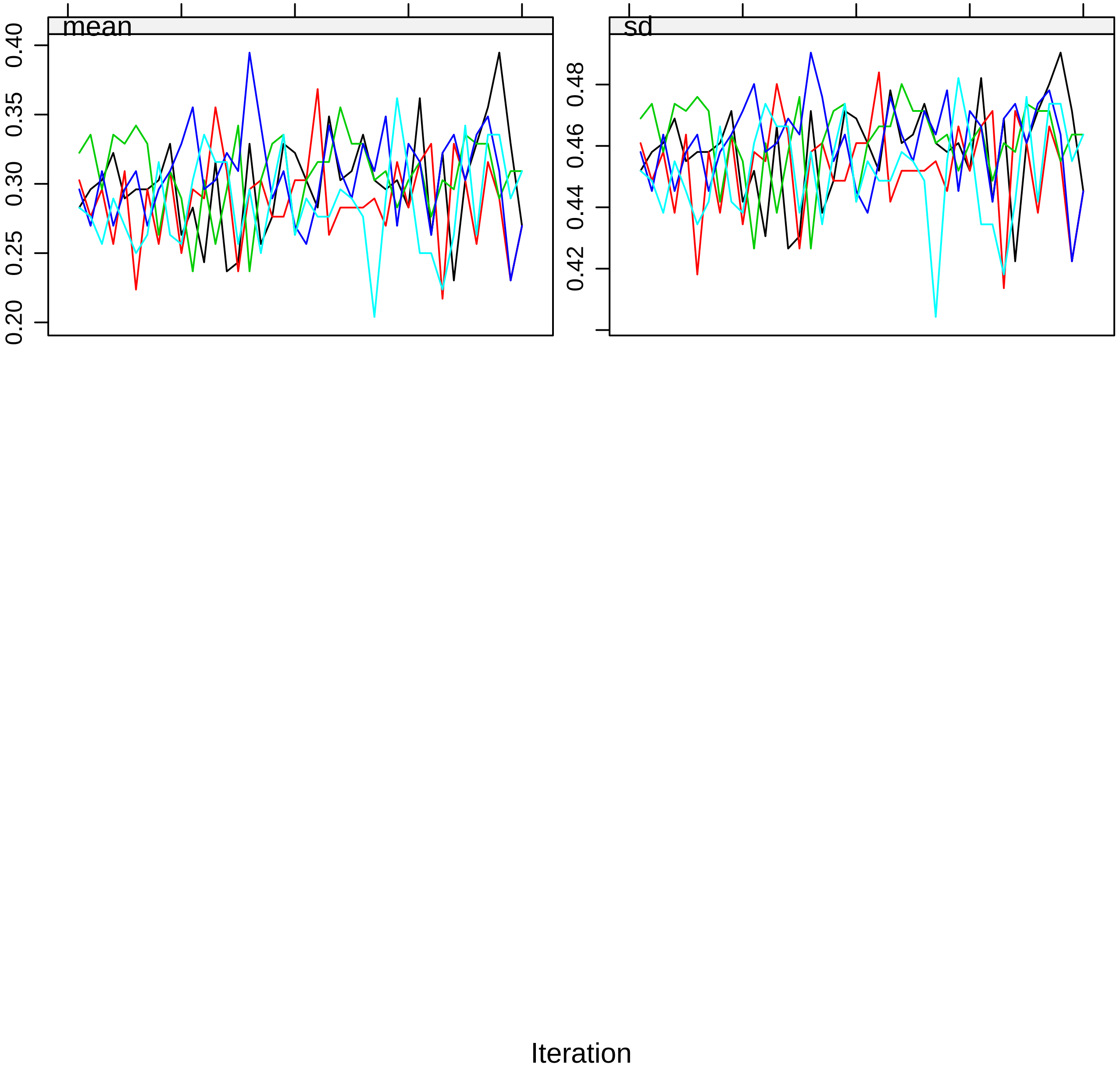

cna_9_p

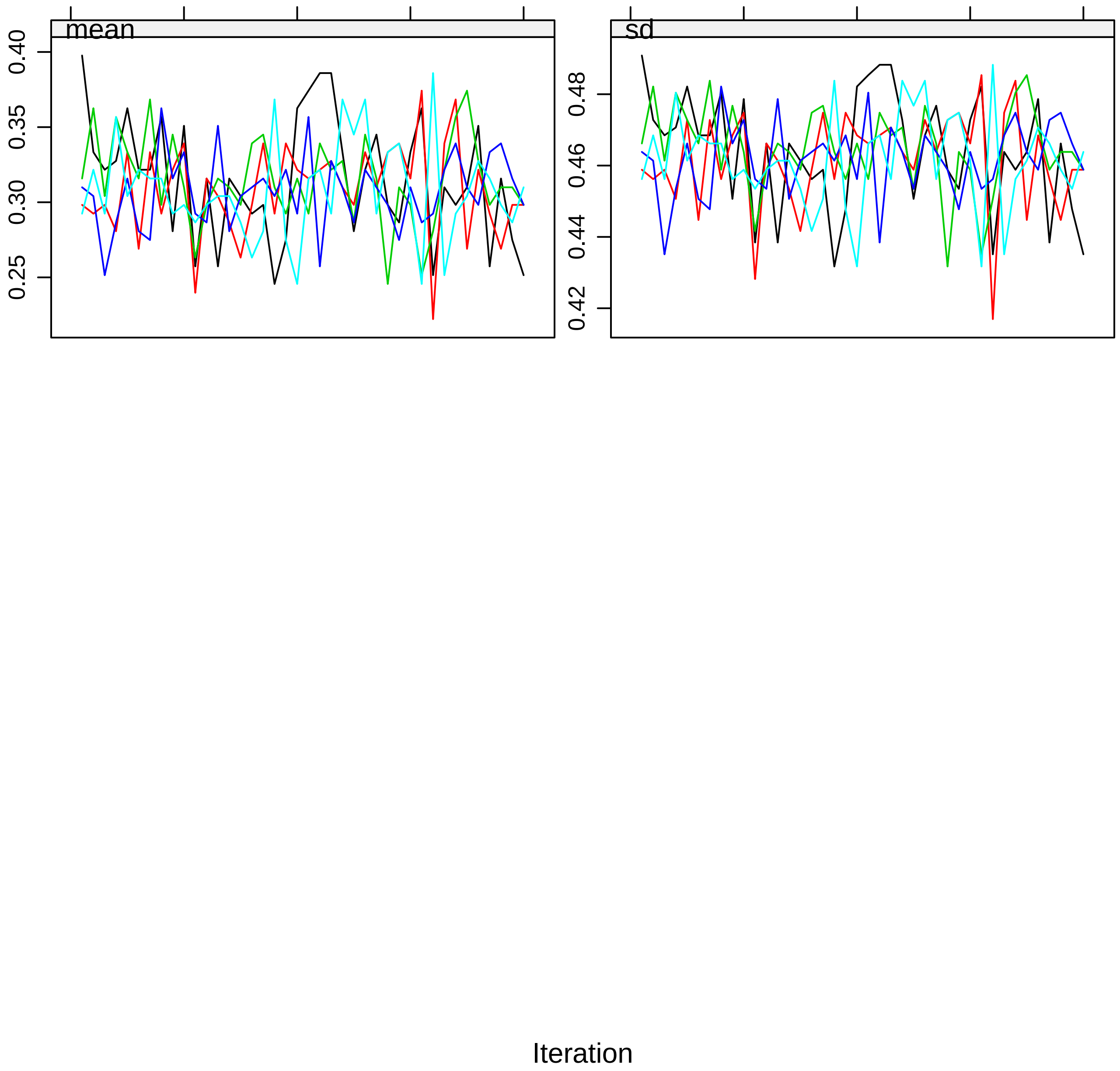

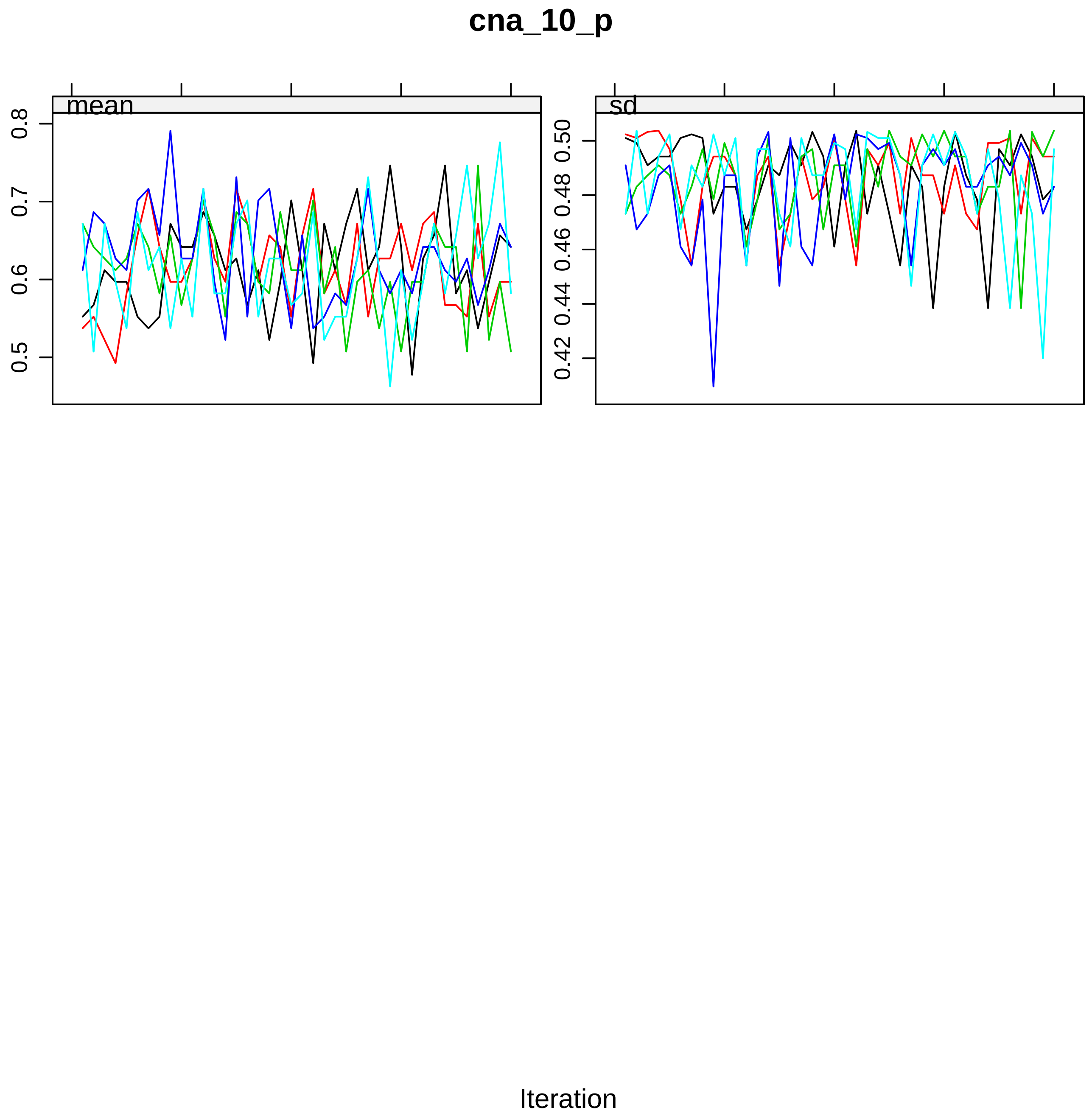
cna_10_p
mean
sd
0.5
0.6
0.7
0.8
0.42
0.44
0.46
0.48
0.50
Iteration

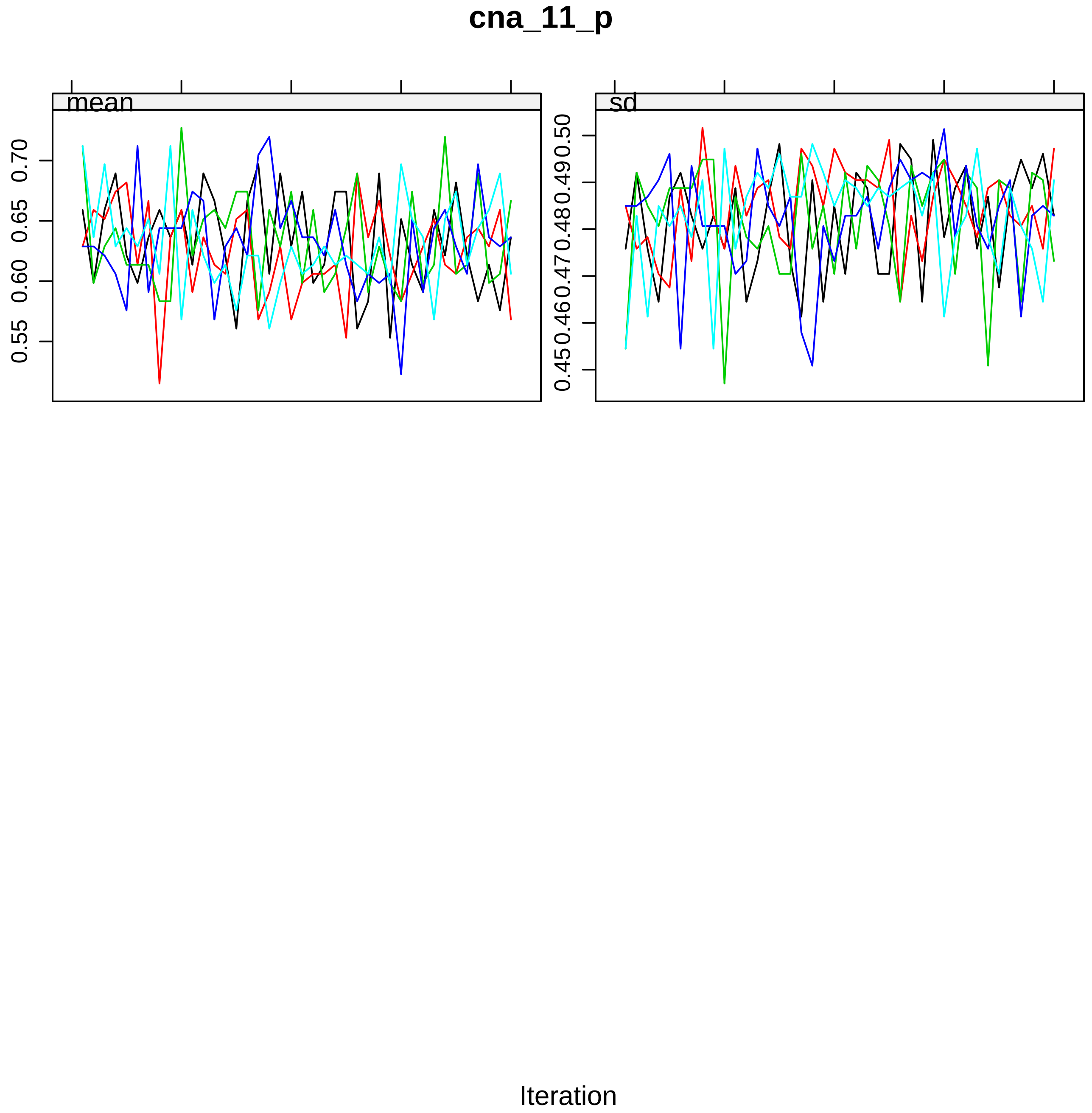

cna_11_p
mean
sd
0.55
0.60
0.65
0.70
0.45
0.46
0.47
0.48
0.49
0.50
Iteration

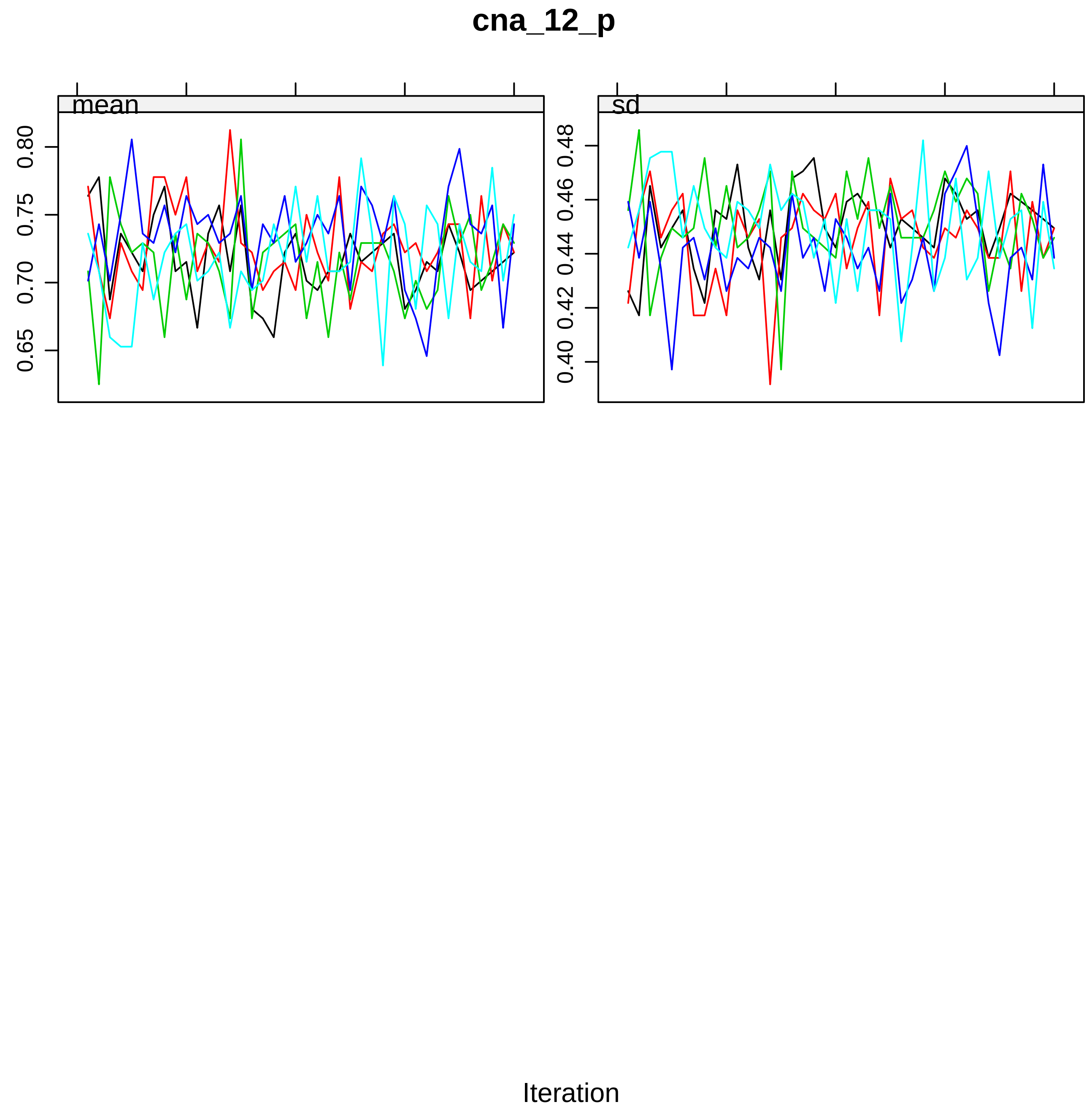
cna_12_p
mean
sd
0.80
0.75
0.70
0.65
0.48
0.46
0.44
0.42
0.40
Iteration

cna_13_p

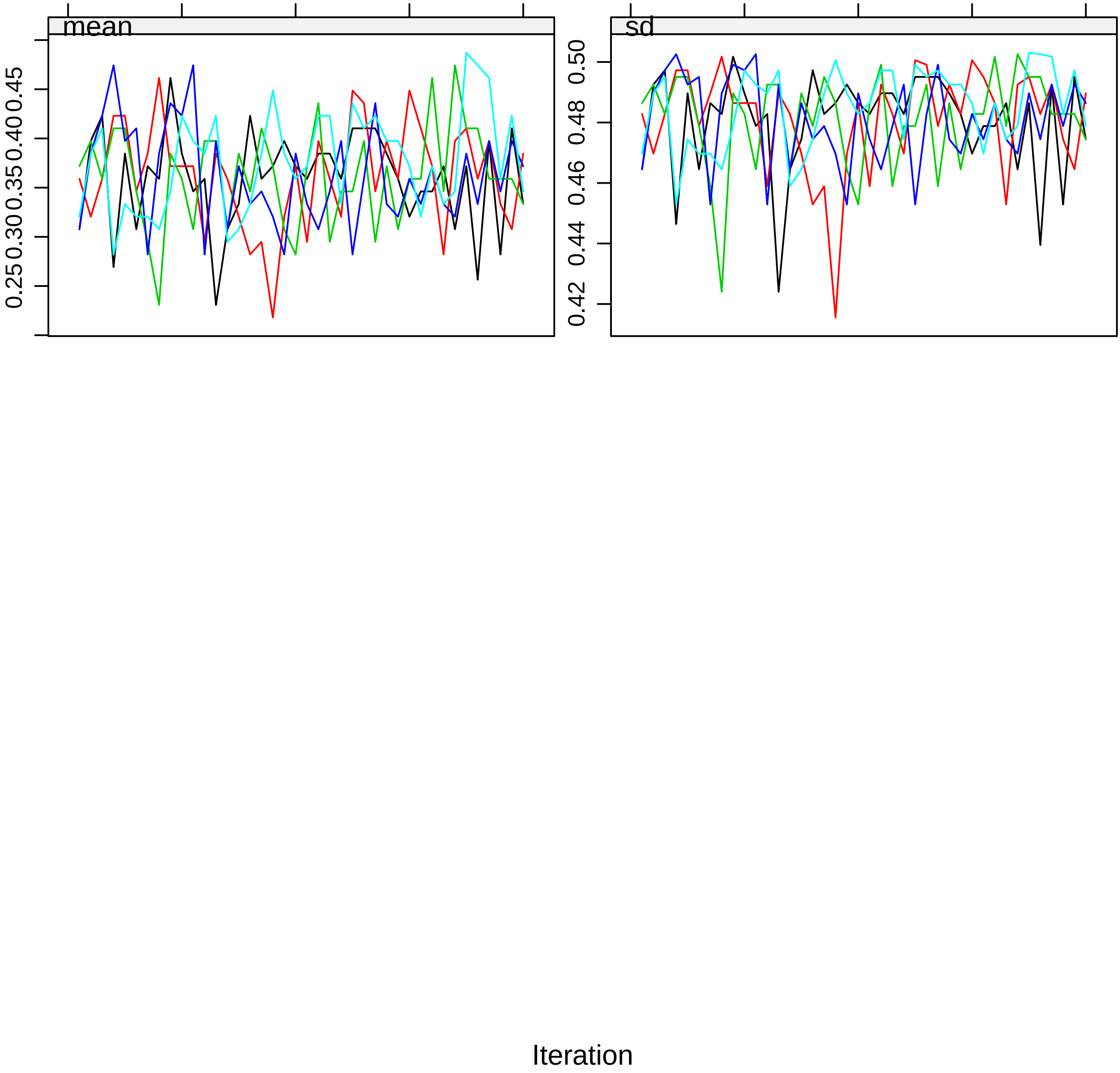

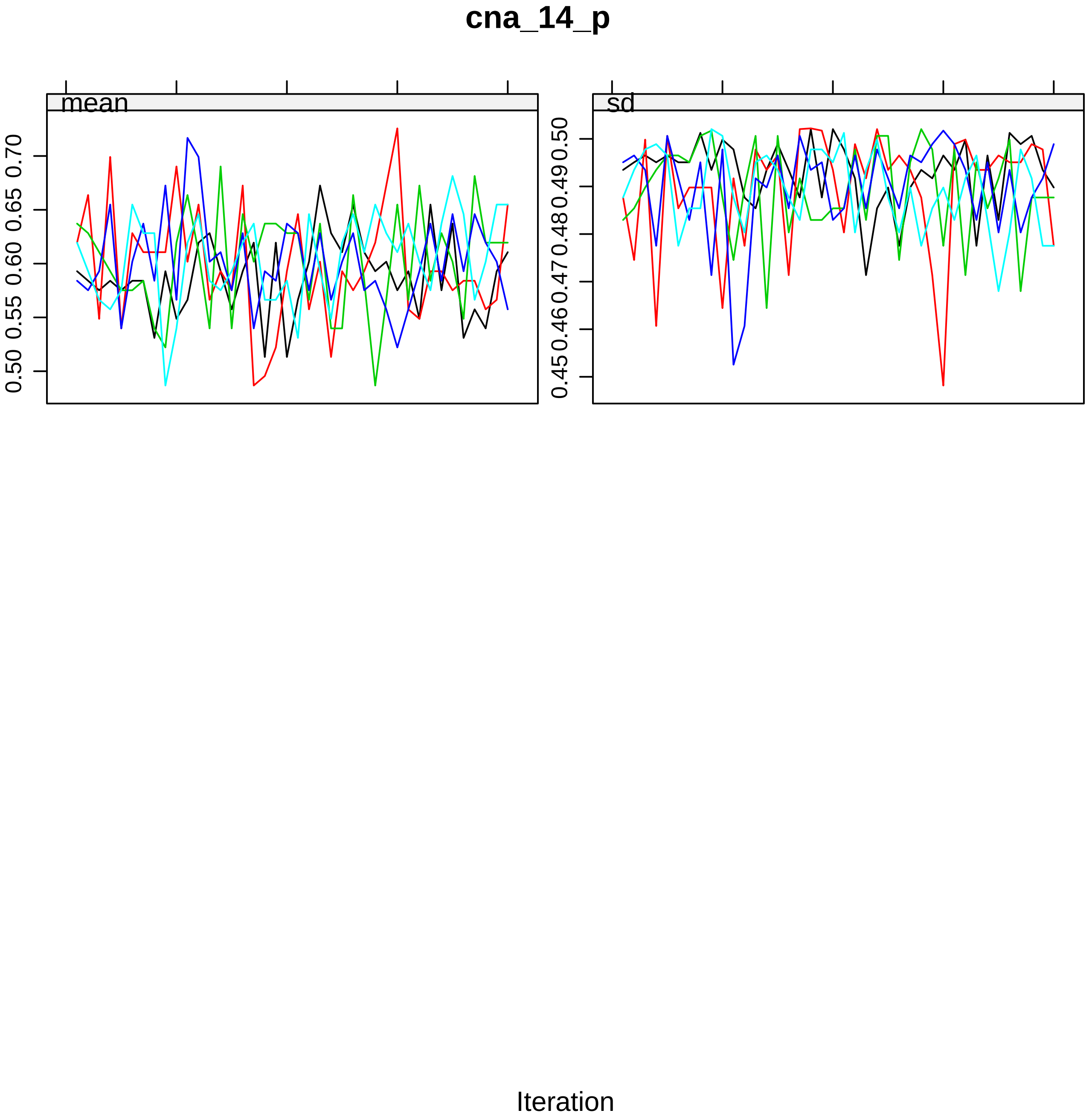

cna_14_p
mean
sd
0.50 0.55 0.60 0.65 0.70
0.45 0.46 0.47 0.48 0.49 0.50
Iteration

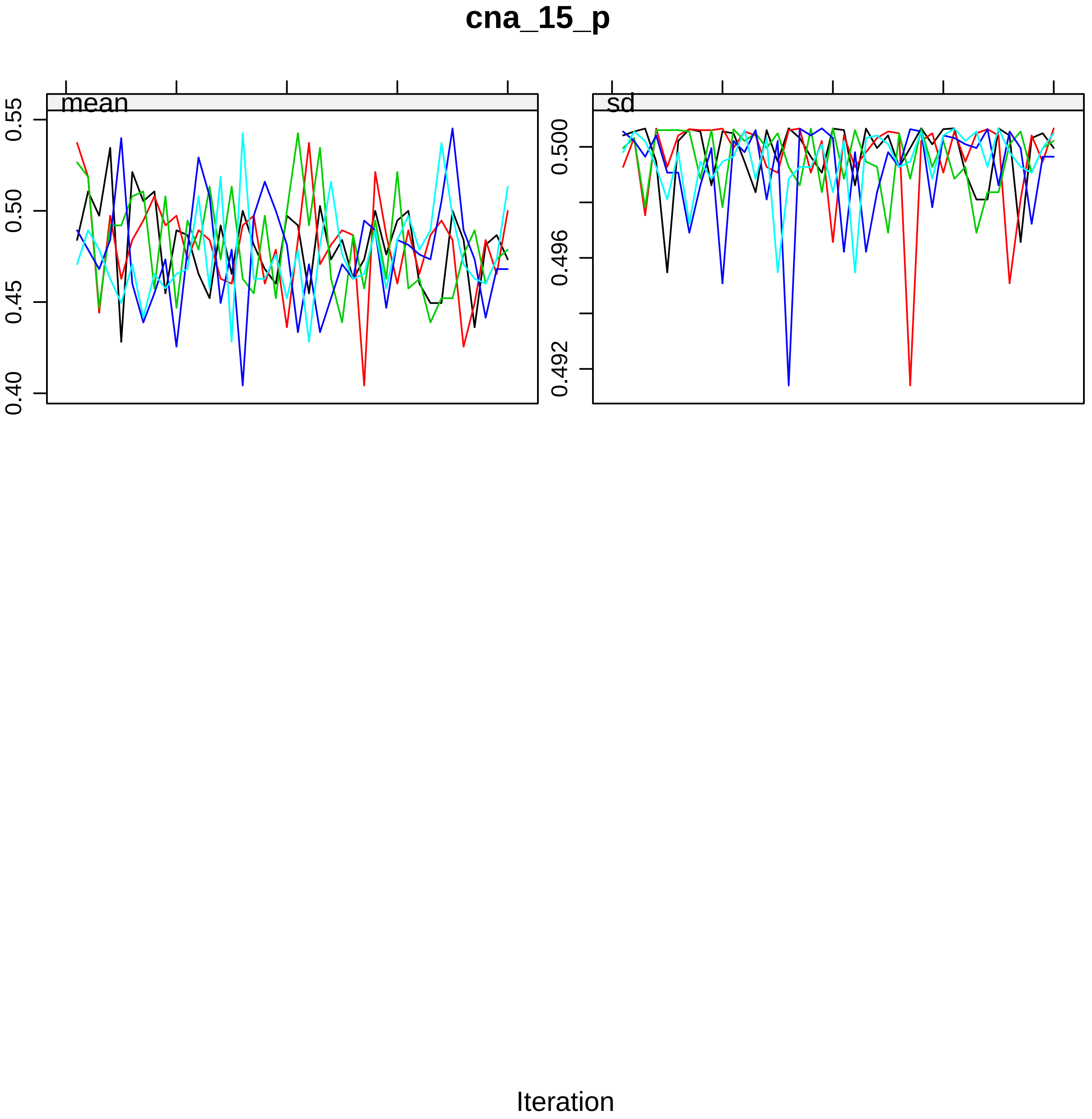
cna_15_p
mean
sd
0.40
0.45
0.50
0.55
0.492
0.496
0.500
Iteration

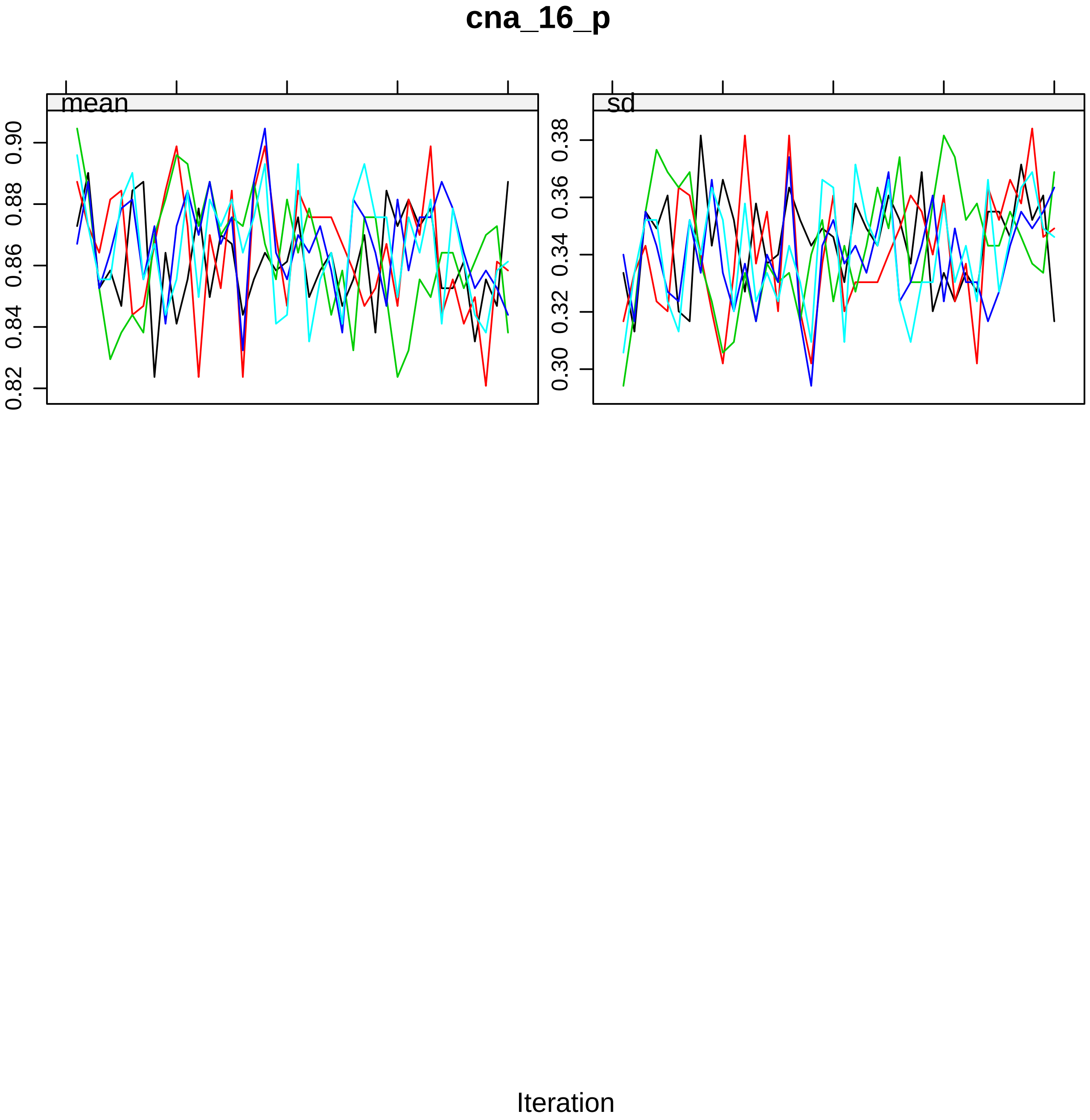

cna_16_p
mean
sd
0.82
0.84
0.86
0.88
0.90
0.30
0.32
0.34
0.36
0.38
Iteration

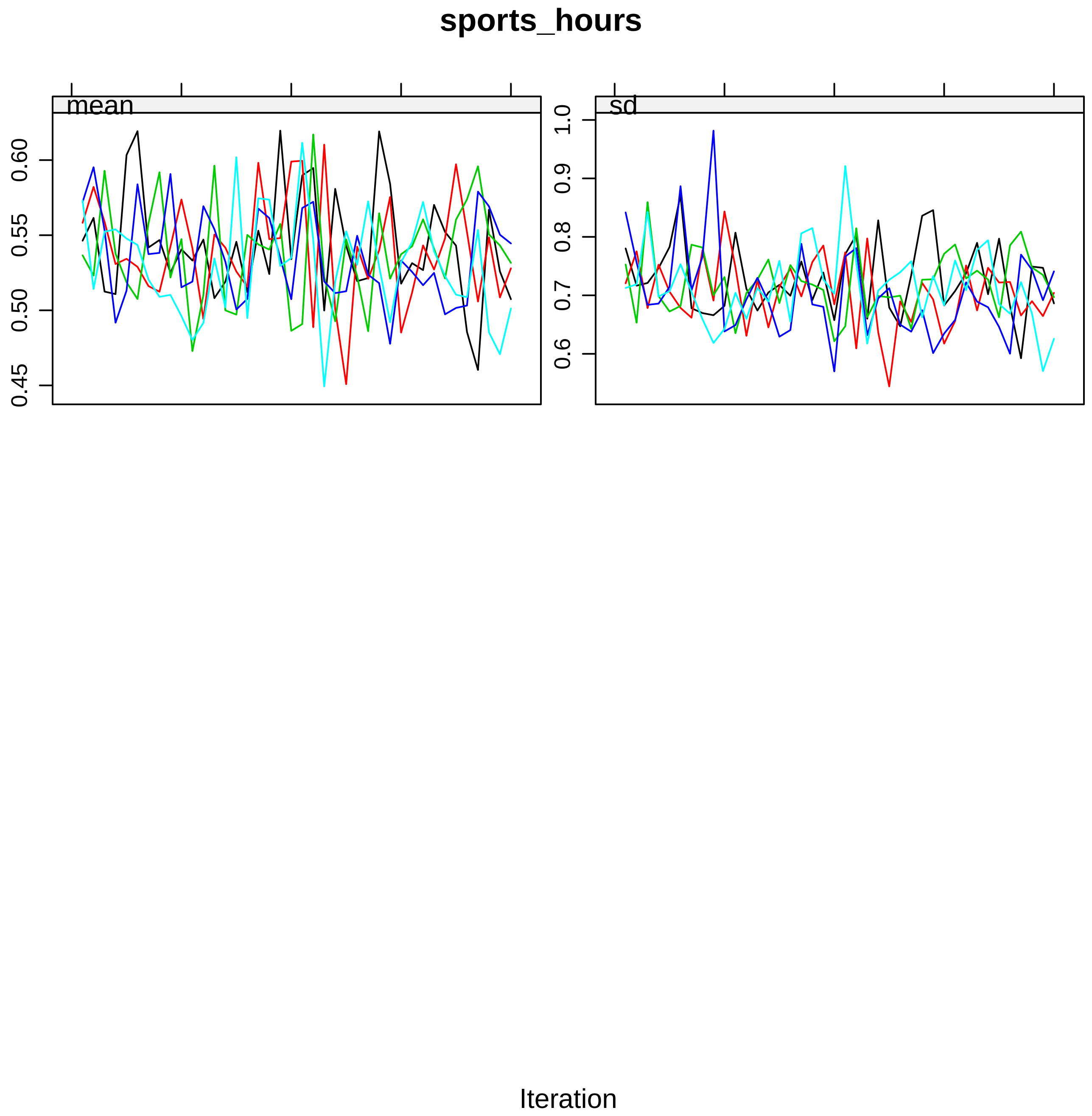
sports_hours
mean
sd
0.45
0.50
0.55
0.60
0.6
0.7
0.8
0.9
1.0
Iteration

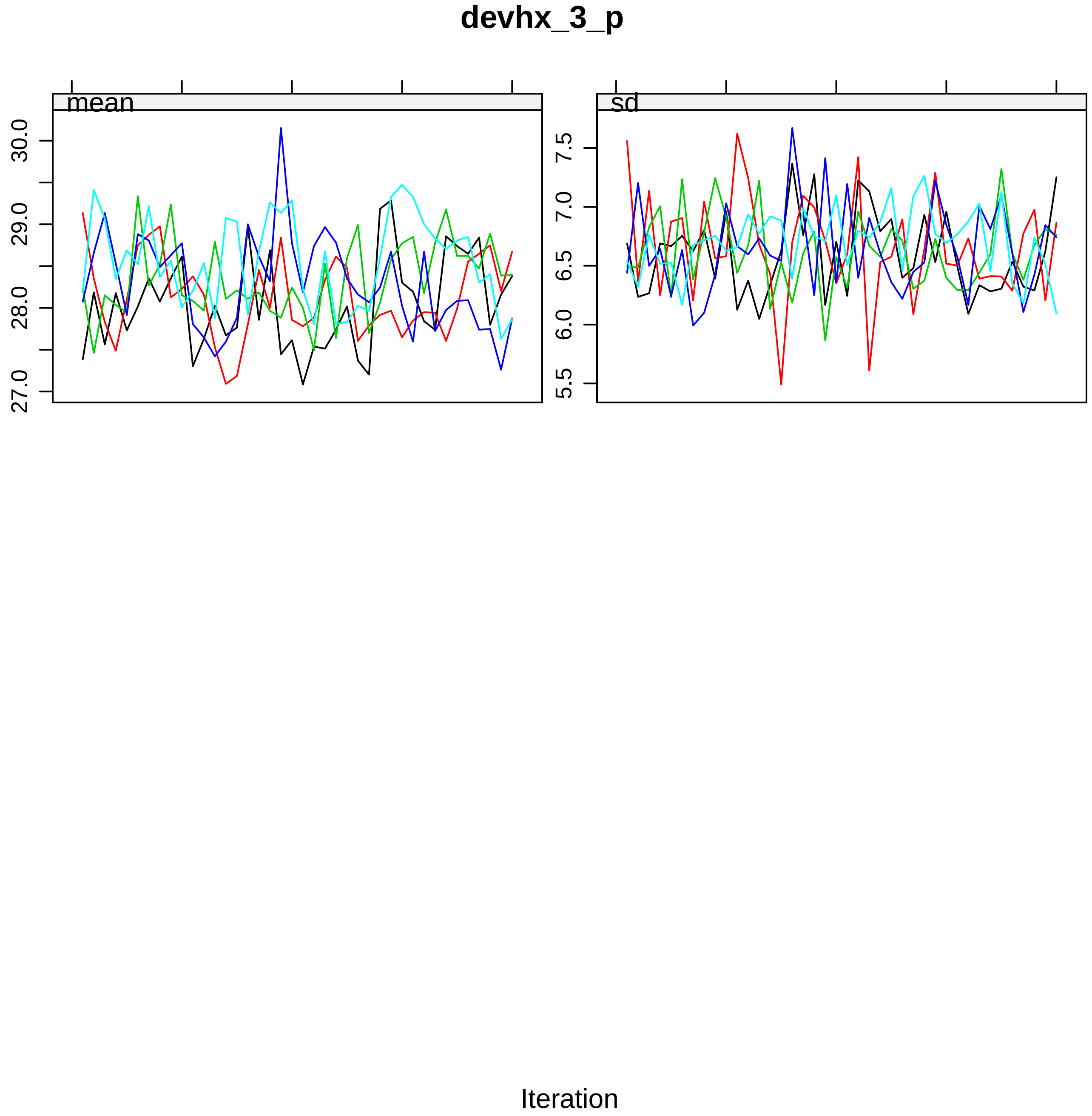

devhx_3_p
mean
sd
30.0
29.0
28.0
27.0
7.5
7.0
6.5
6.0
5.5
Iteration

devhx_4_p

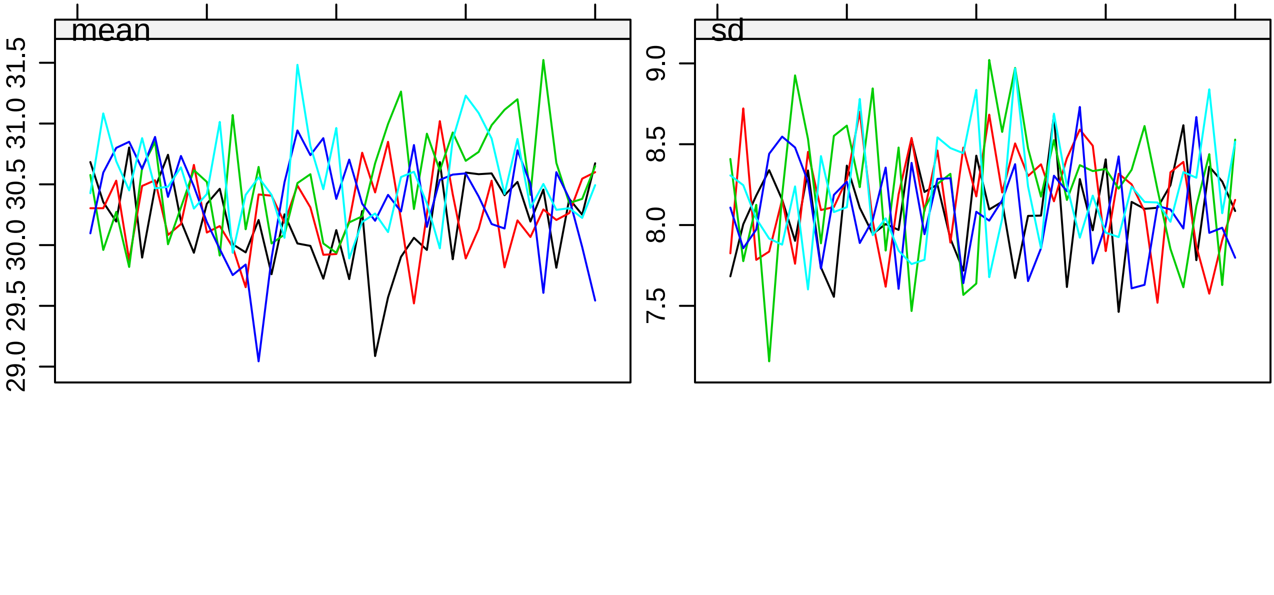

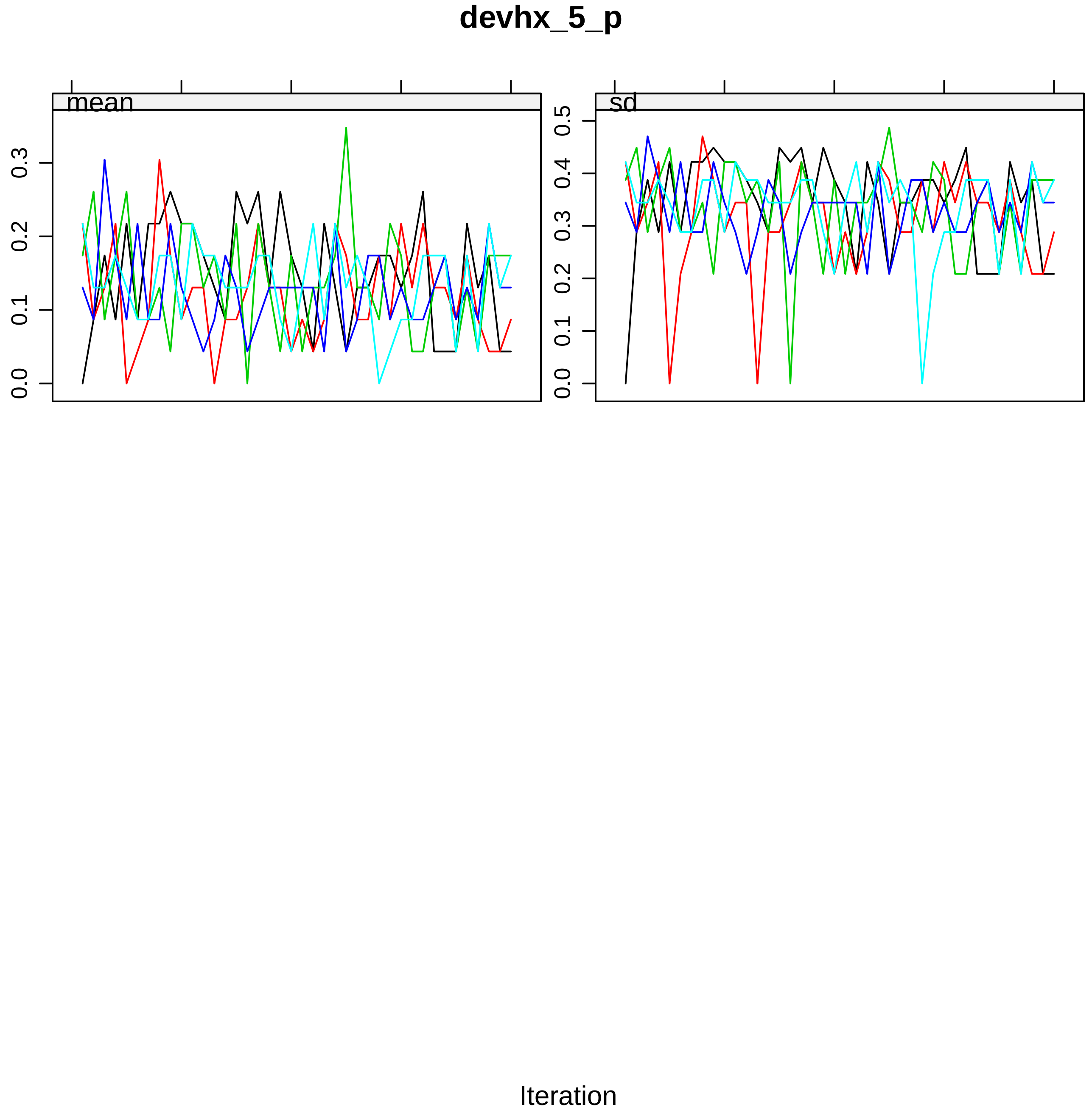
devhx_5_p
mean
sd
0.0
0.1
0.2
0.3
0.4
0.5
Iteration

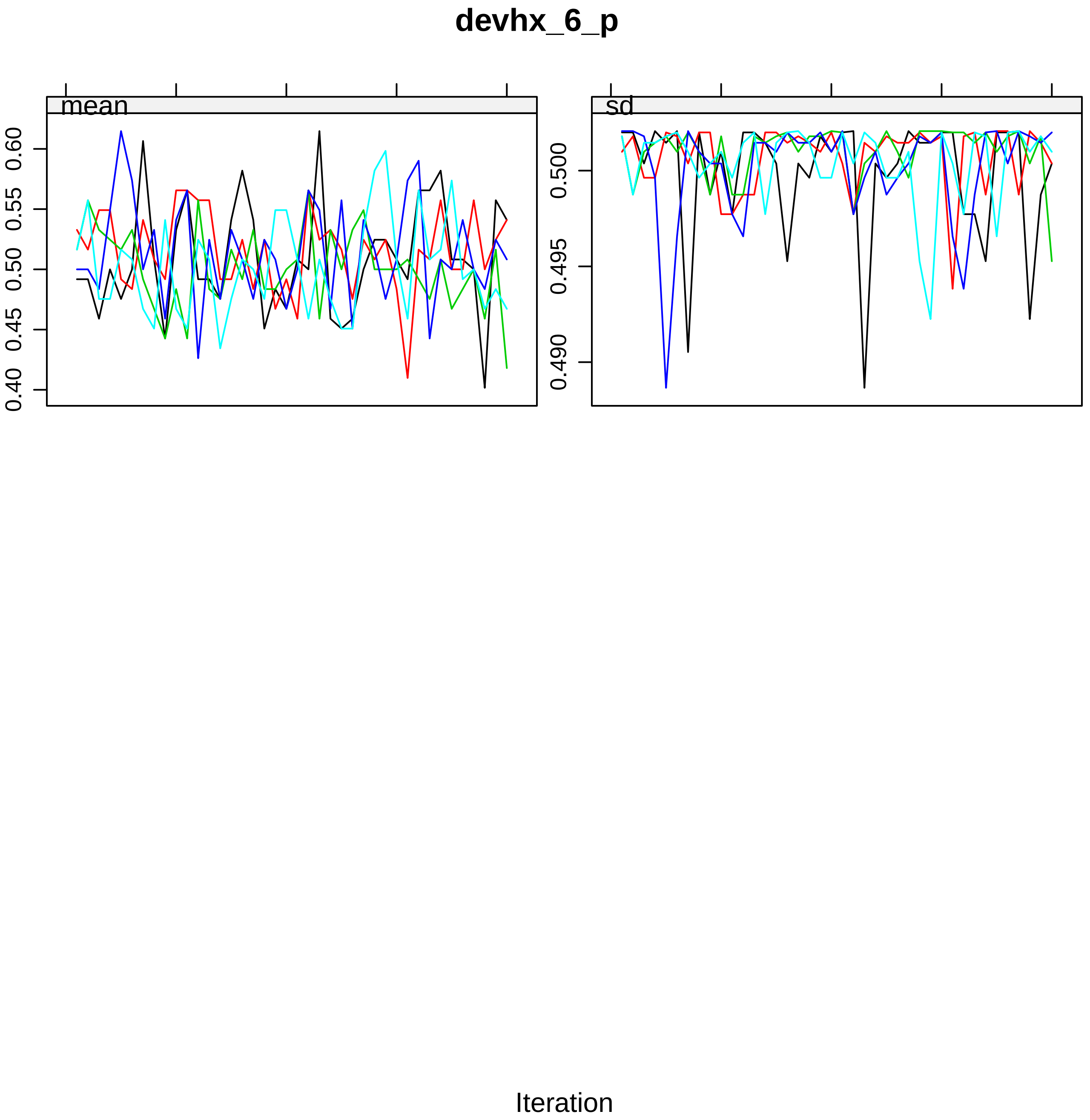
devhx_6_p
mean
sd
0.40
0.45
0.50
0.55
0.60
0.490
0.495
0.500
Iteration

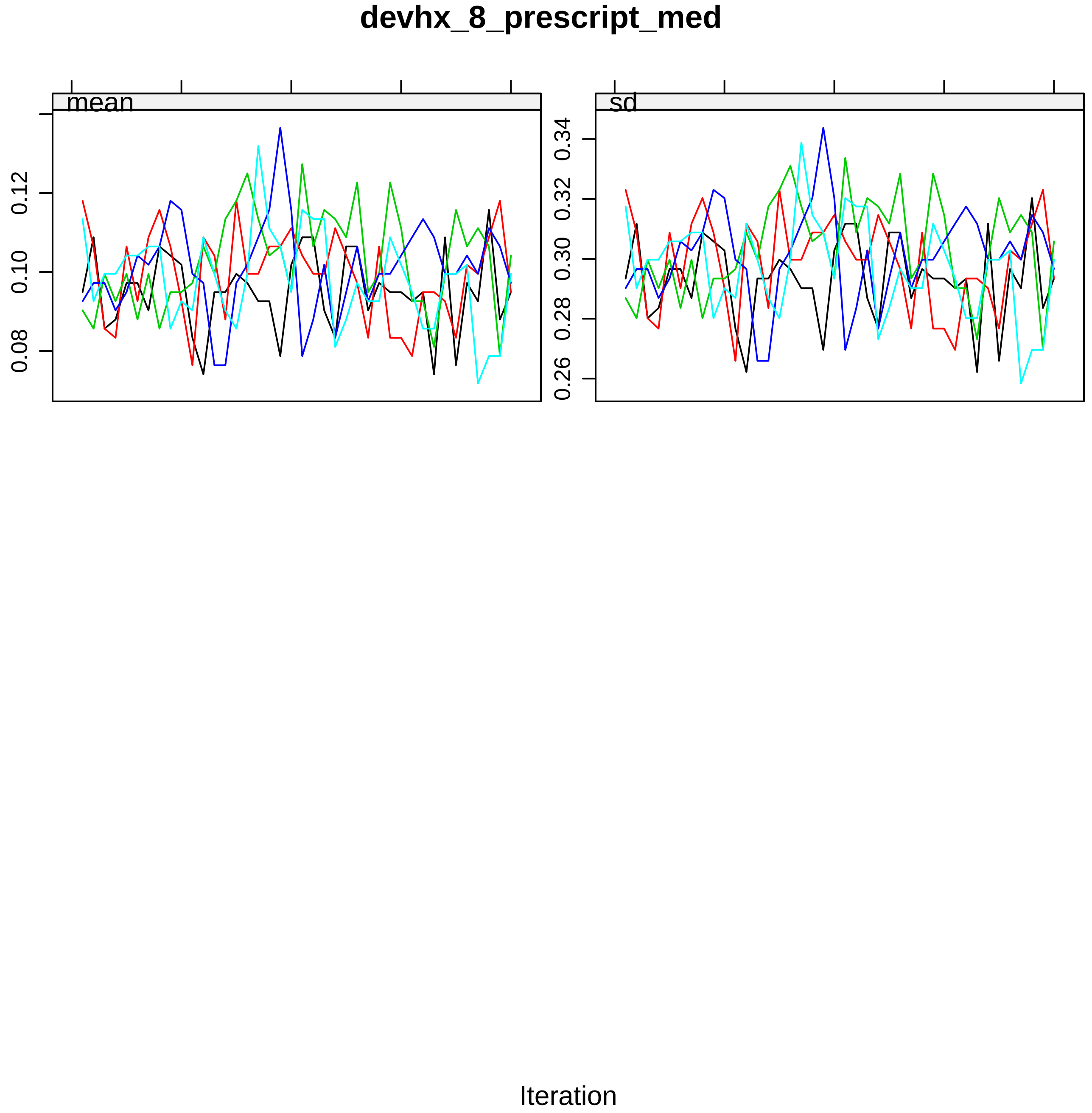
devhx_8_prescript_med
mean
sd
0.08
0.10
0.12
0.26
0.28
0.30
0.32
0.34
Iteration

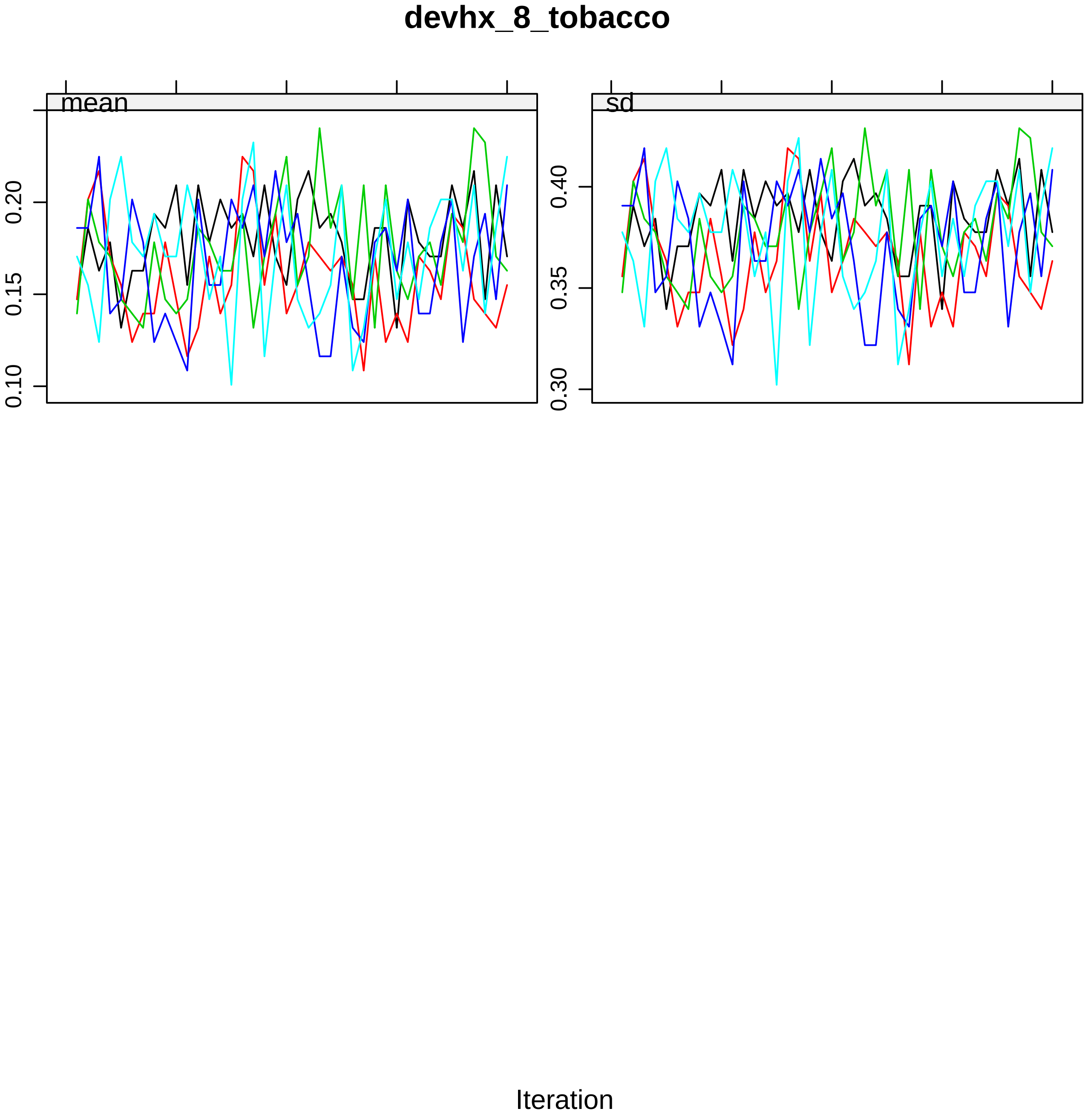
devhx_8_tobacco
mean
sd
0.10
0.15
0.20
0.30
0.35
0.40
Iteration

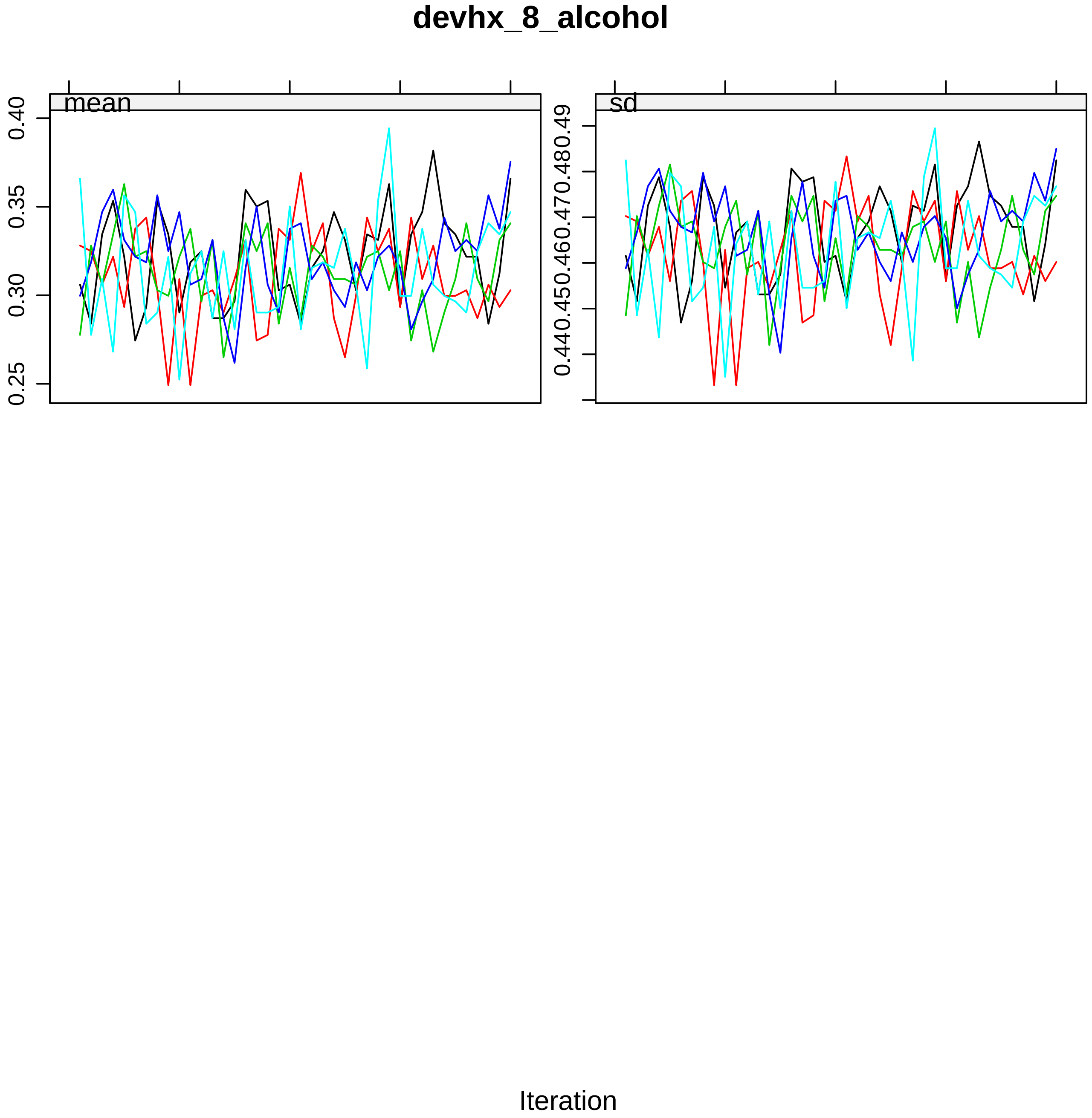
devhx_8_alcohol
mean
sd
0.25
0.30
0.35
0.40
0.44
0.45
0.46
0.47
0.48
0.49
Iteration

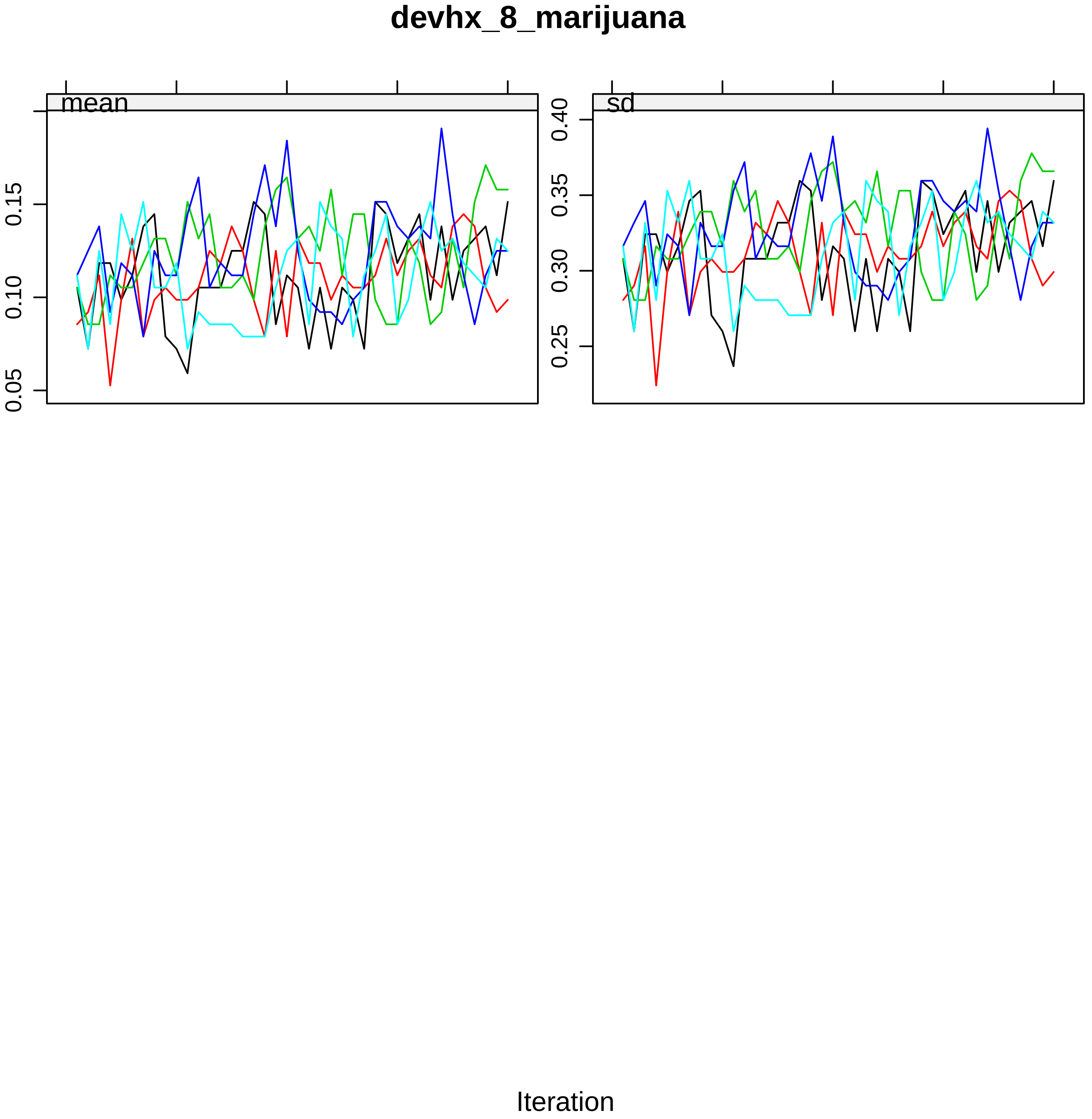
devhx_8_marijuana
mean
sd
0.05
0.10
0.15
0.25
0.30
0.35
0.40
Iteration

devhx_9_prescript_med

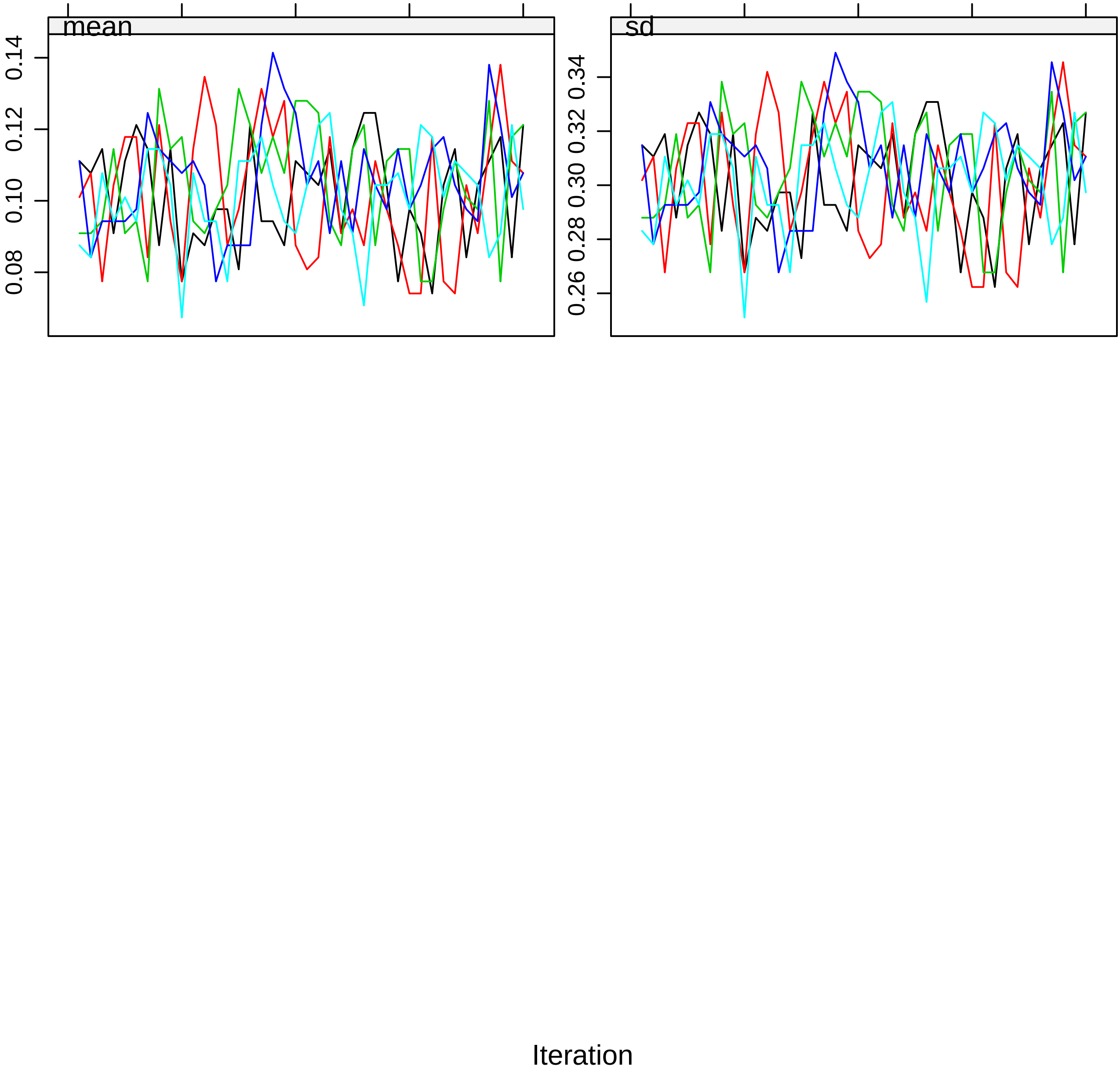

devhx_9_tobacco

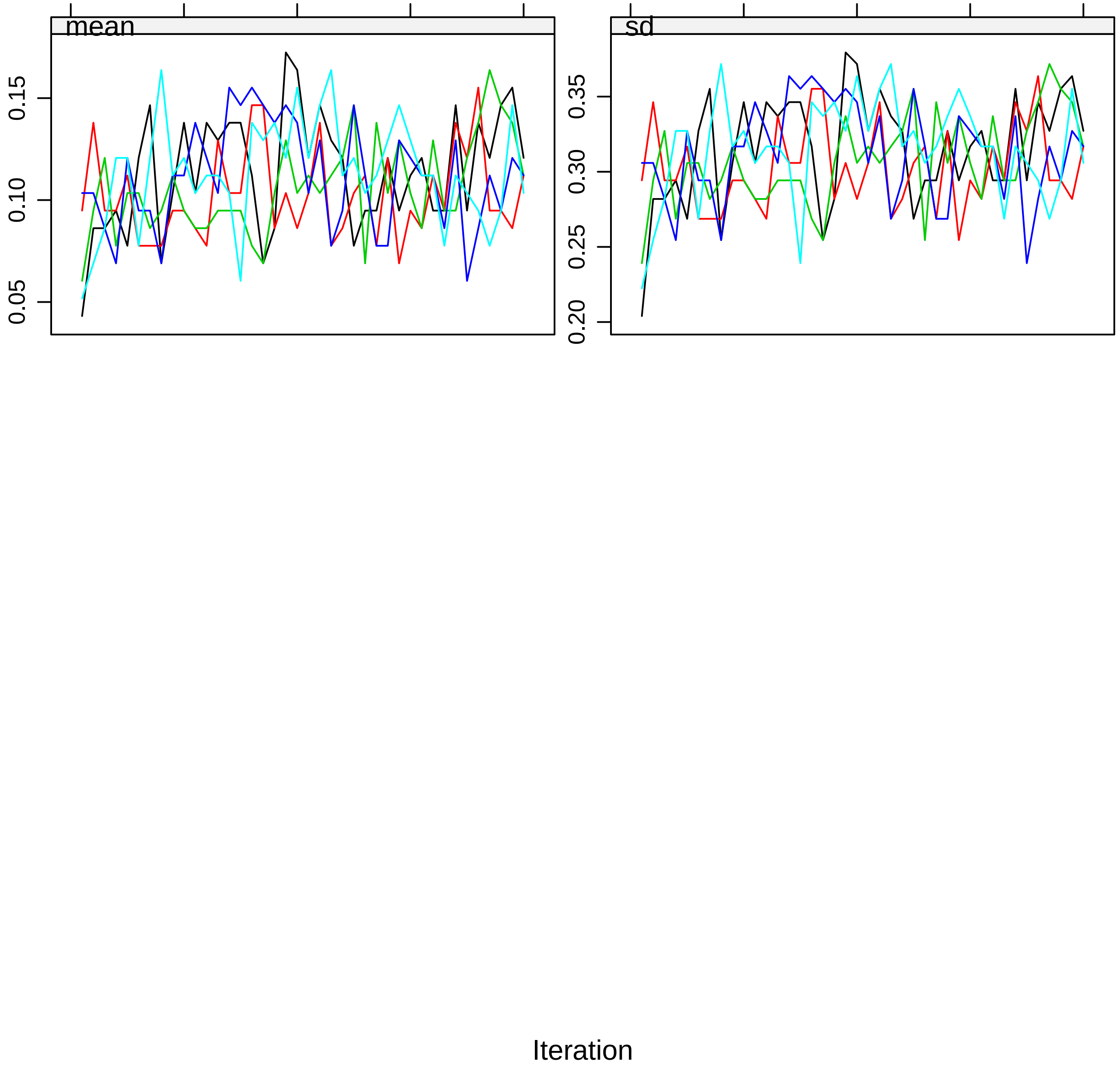

devhx_9_alcohol

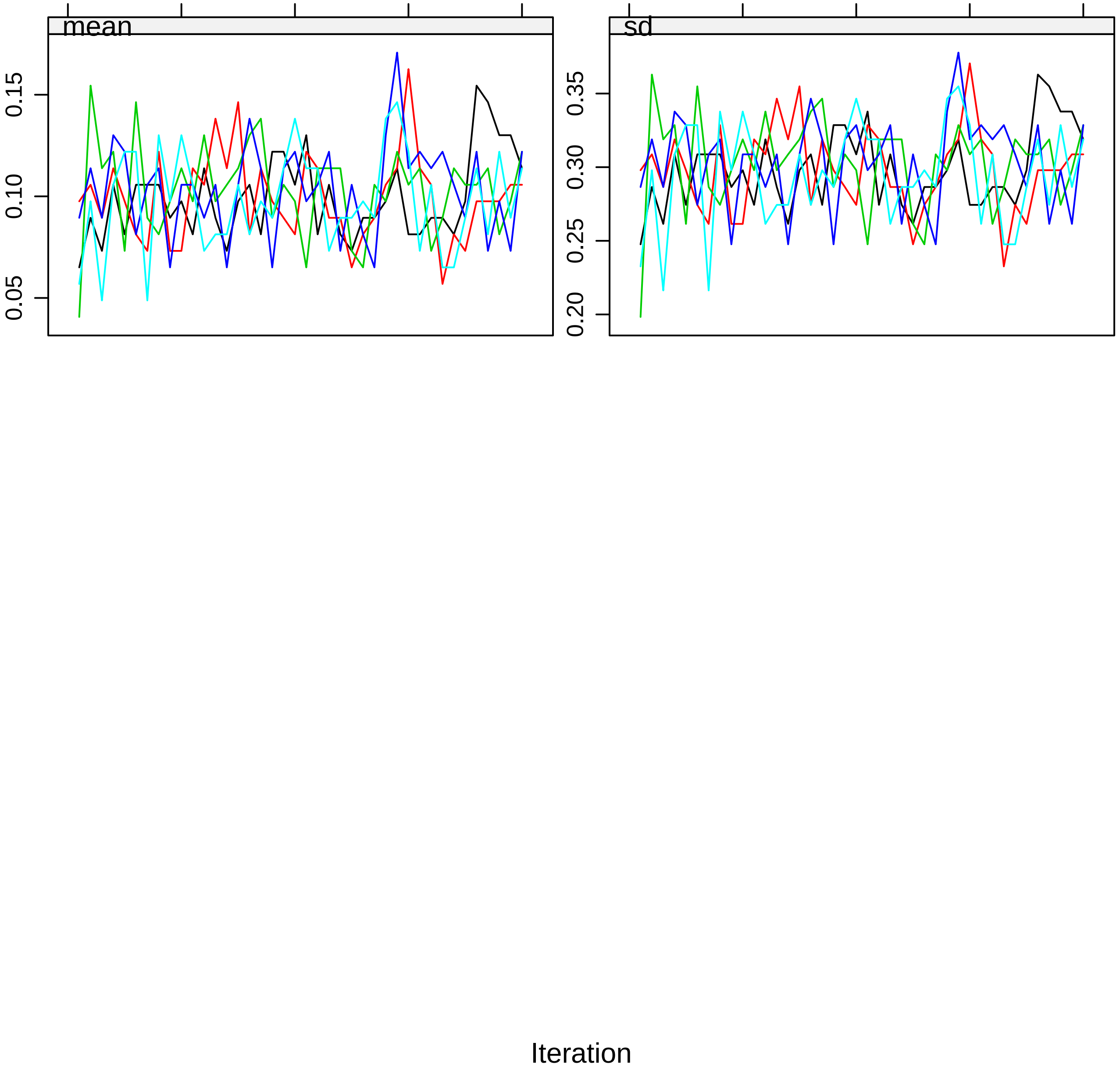

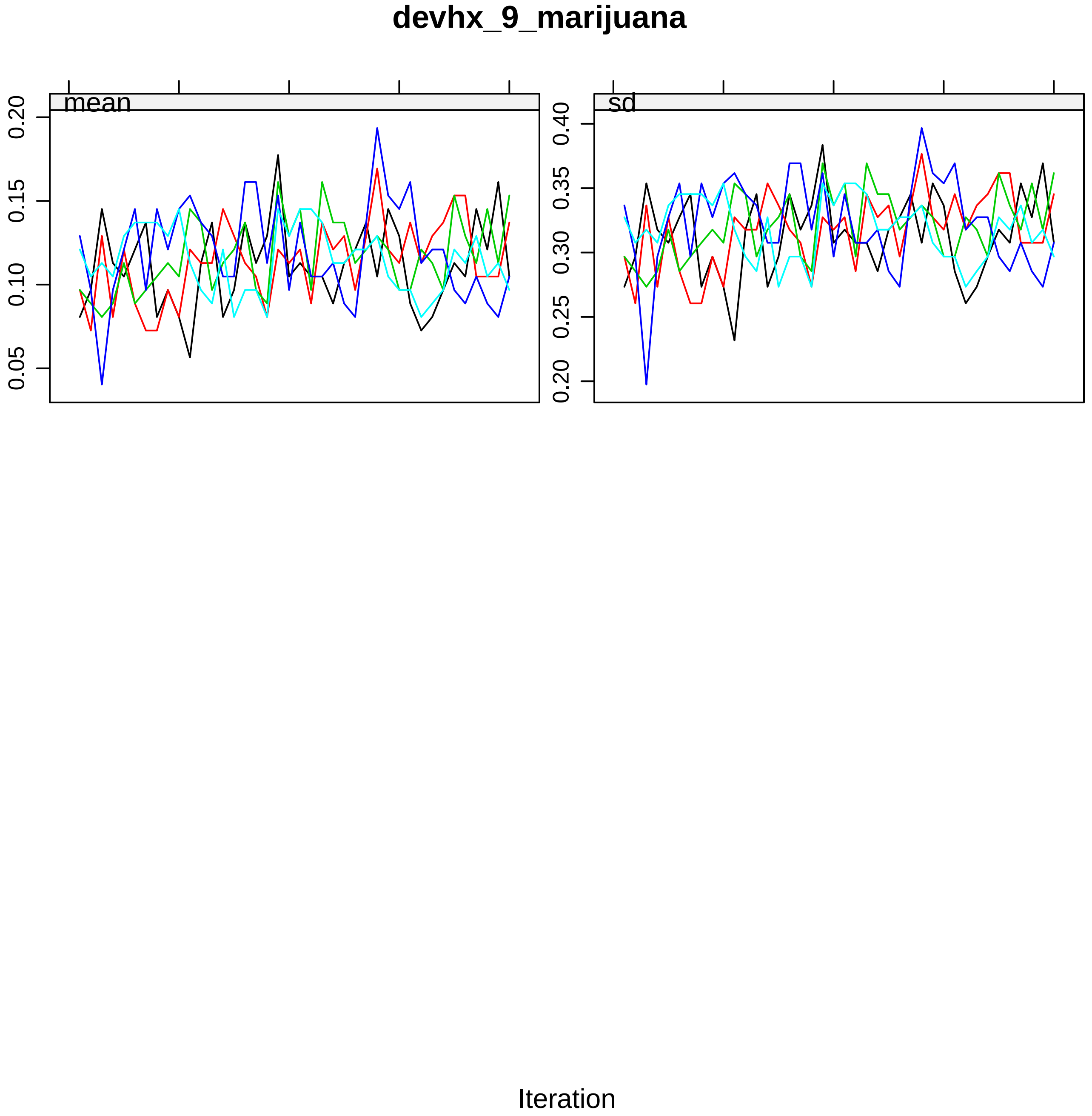
devhx_9_marijuana
mean
sd
0.05
0.10
0.15
0.20
0.20
0.25
0.30
0.35
0.40
Iteration

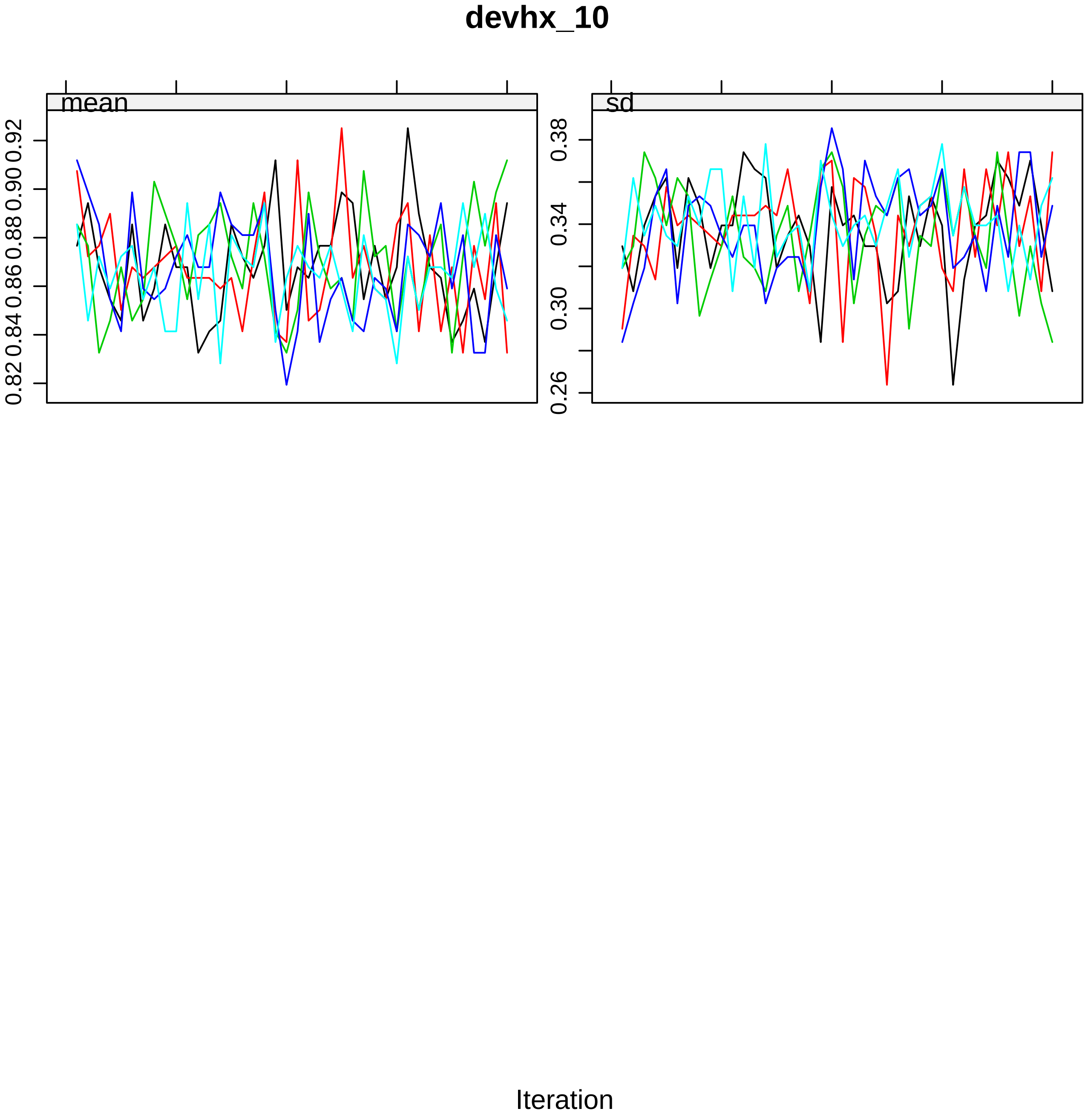
devhx_10
mean
sd
0.82
0.84
0.86
0.88
0.90
0.92
0.26
0.30
0.34
0.38
Iteration

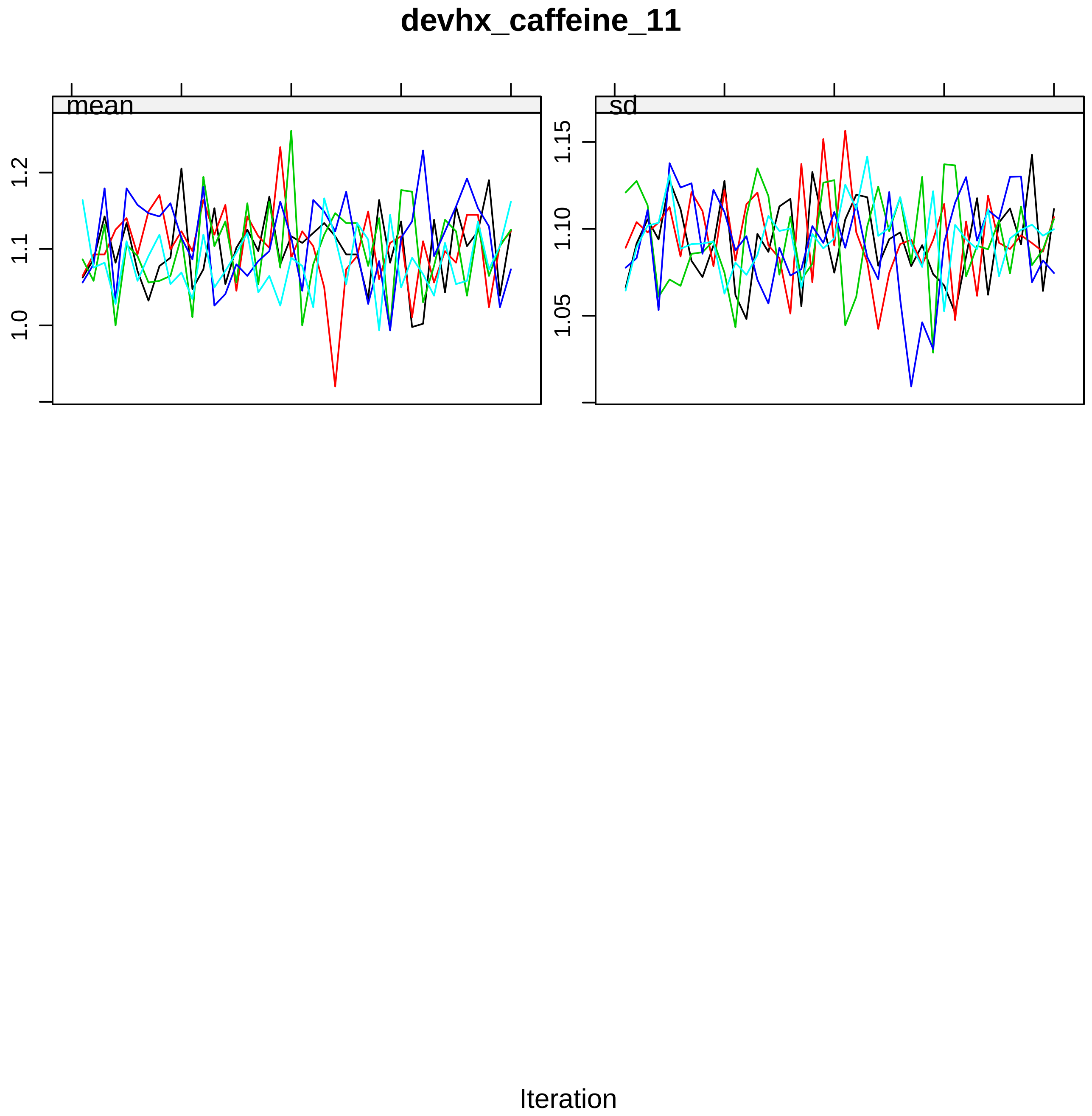
devhx_caffeine_11
mean
sd
1.0
1.1
1.2
1.05
1.10
1.15
Iteration

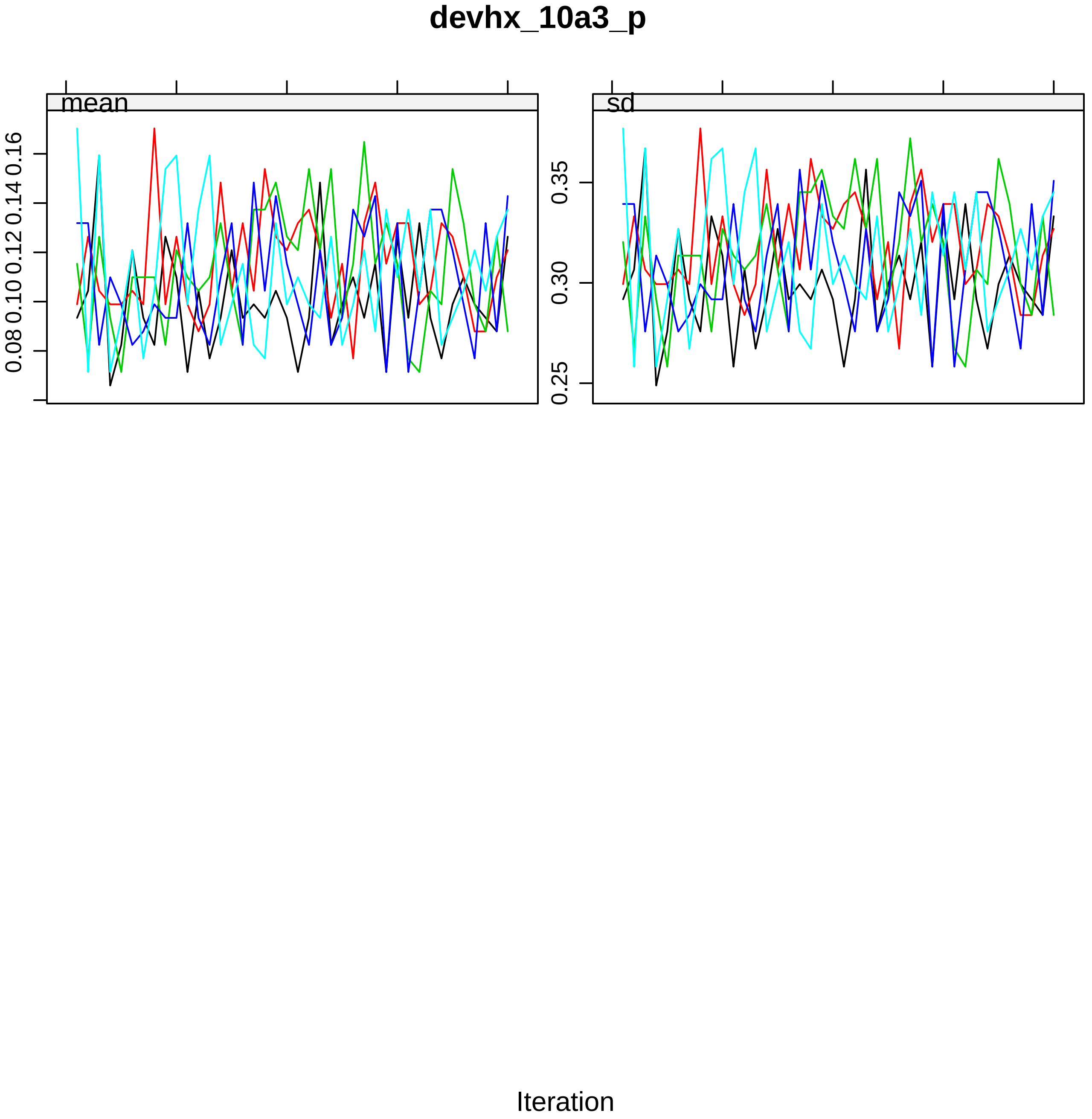
devhx_10a3_p
mean
sd
0.08 0.10 0.12 0.14 0.16
0.25
0.30
0.35
Iteration

devhx_10b3_p

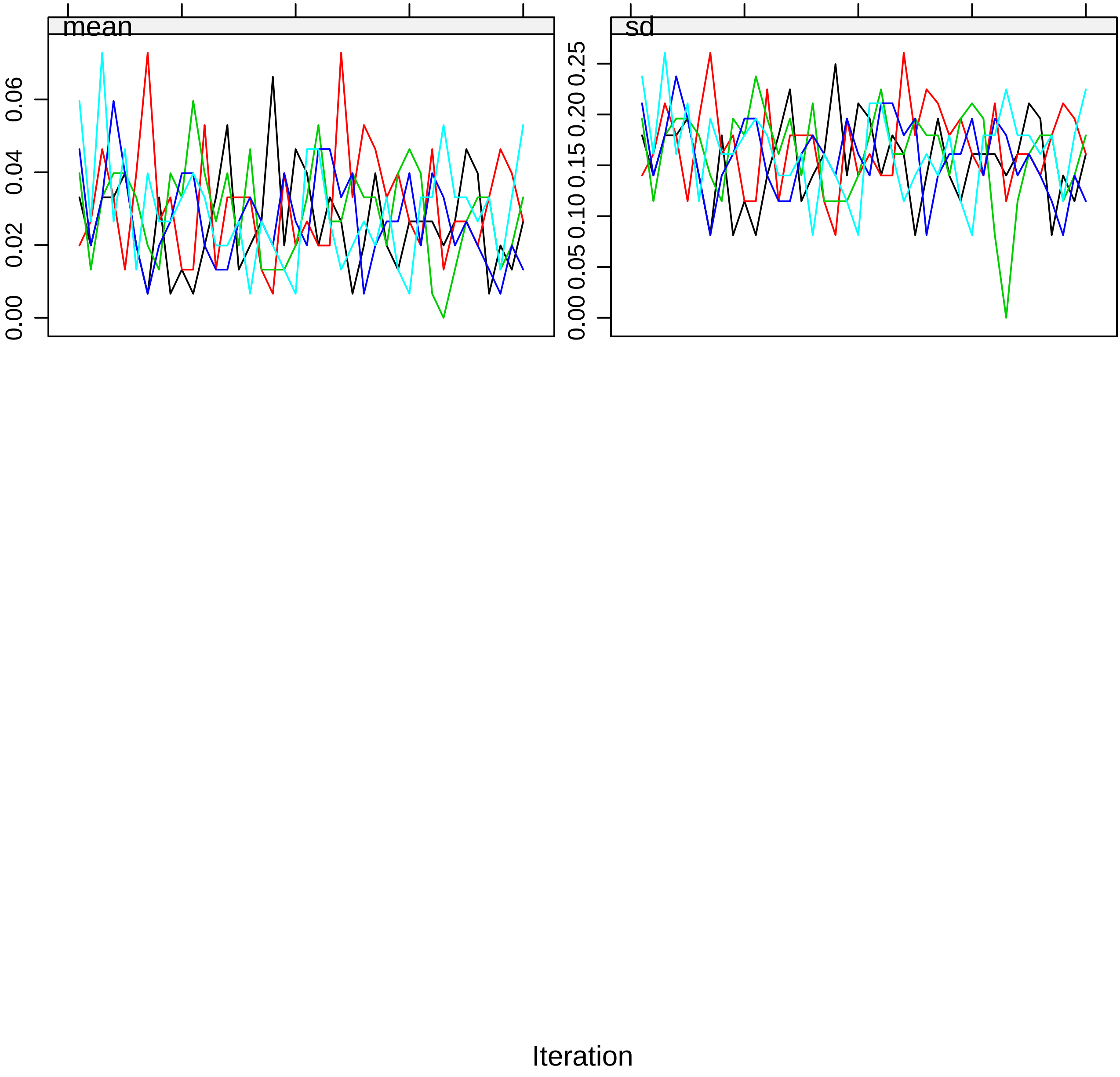

devhx_10c3_p

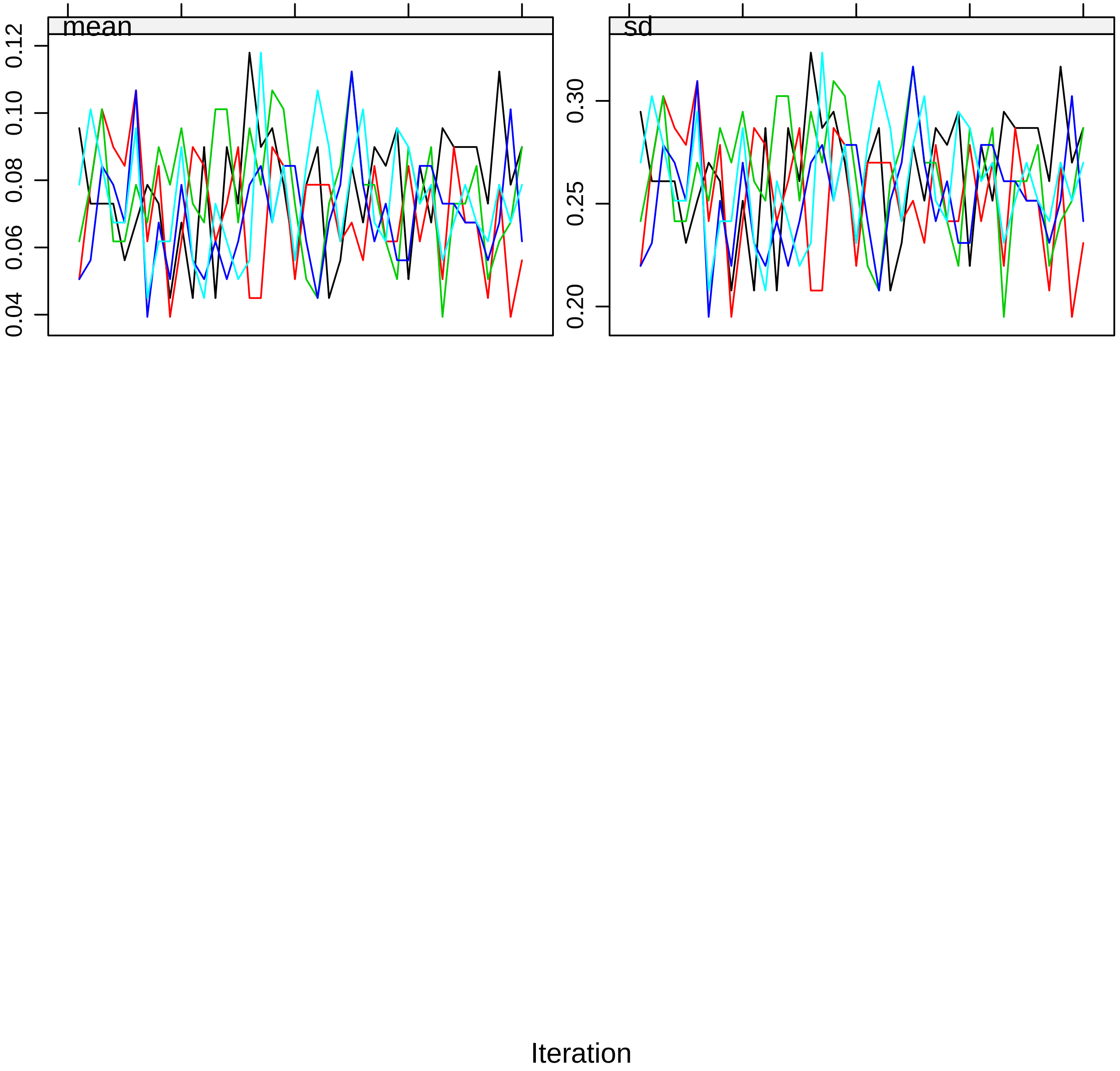

devhx_10d3_p

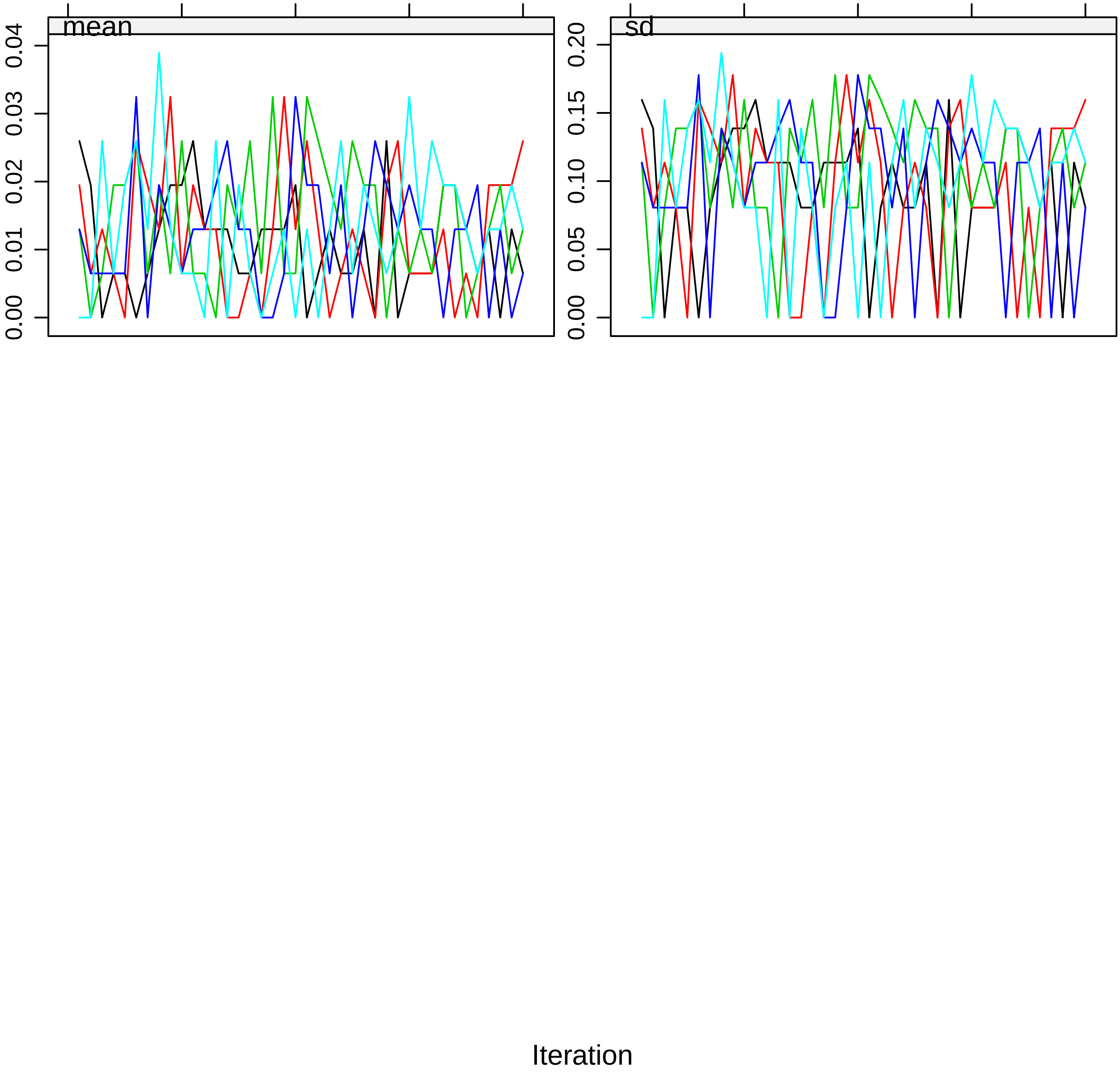

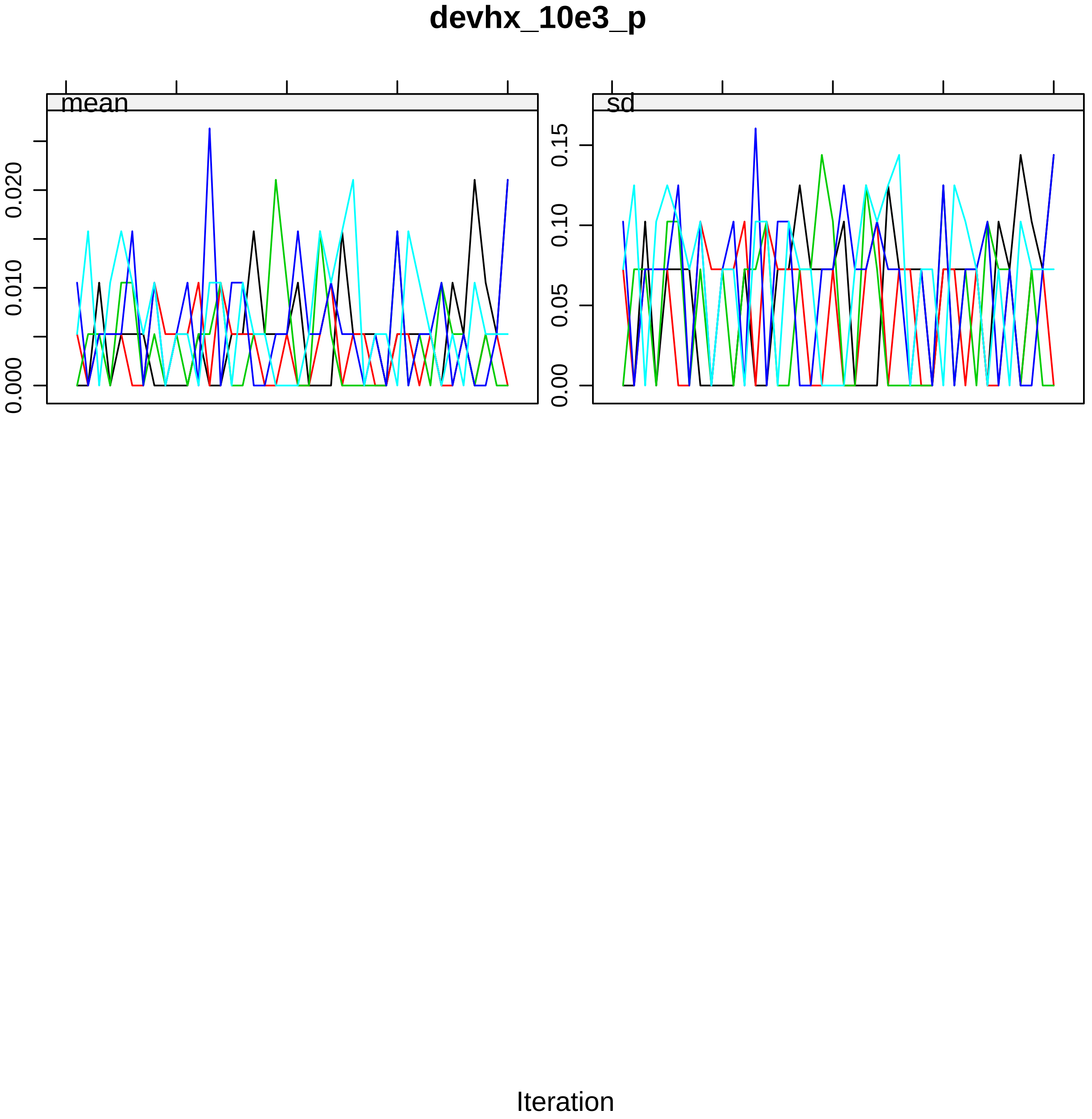
devhx_10e3_p
mean
sd
0.000
0.010
0.020
0.00
0.05
0.10
0.15
Iteration

devhx_10f3_p

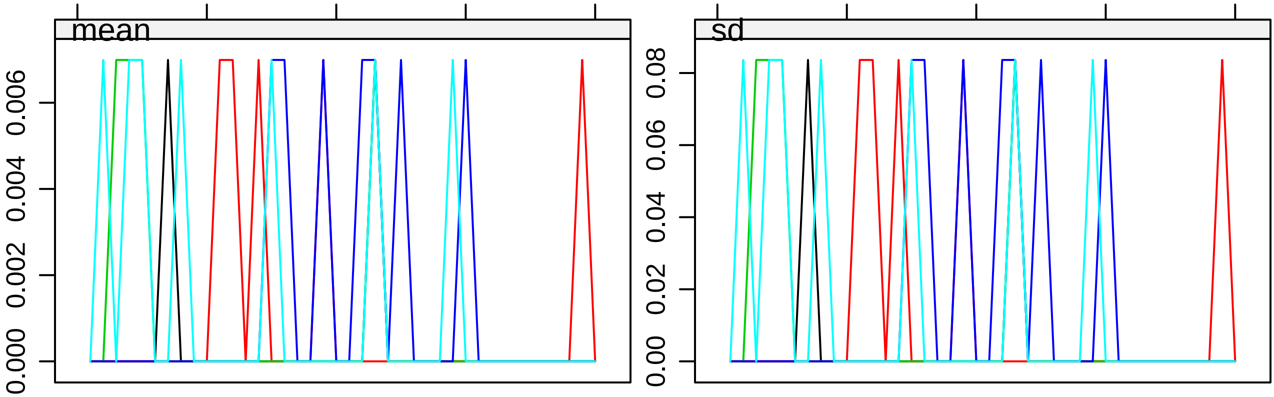

Iteration

devhx_10g3_p

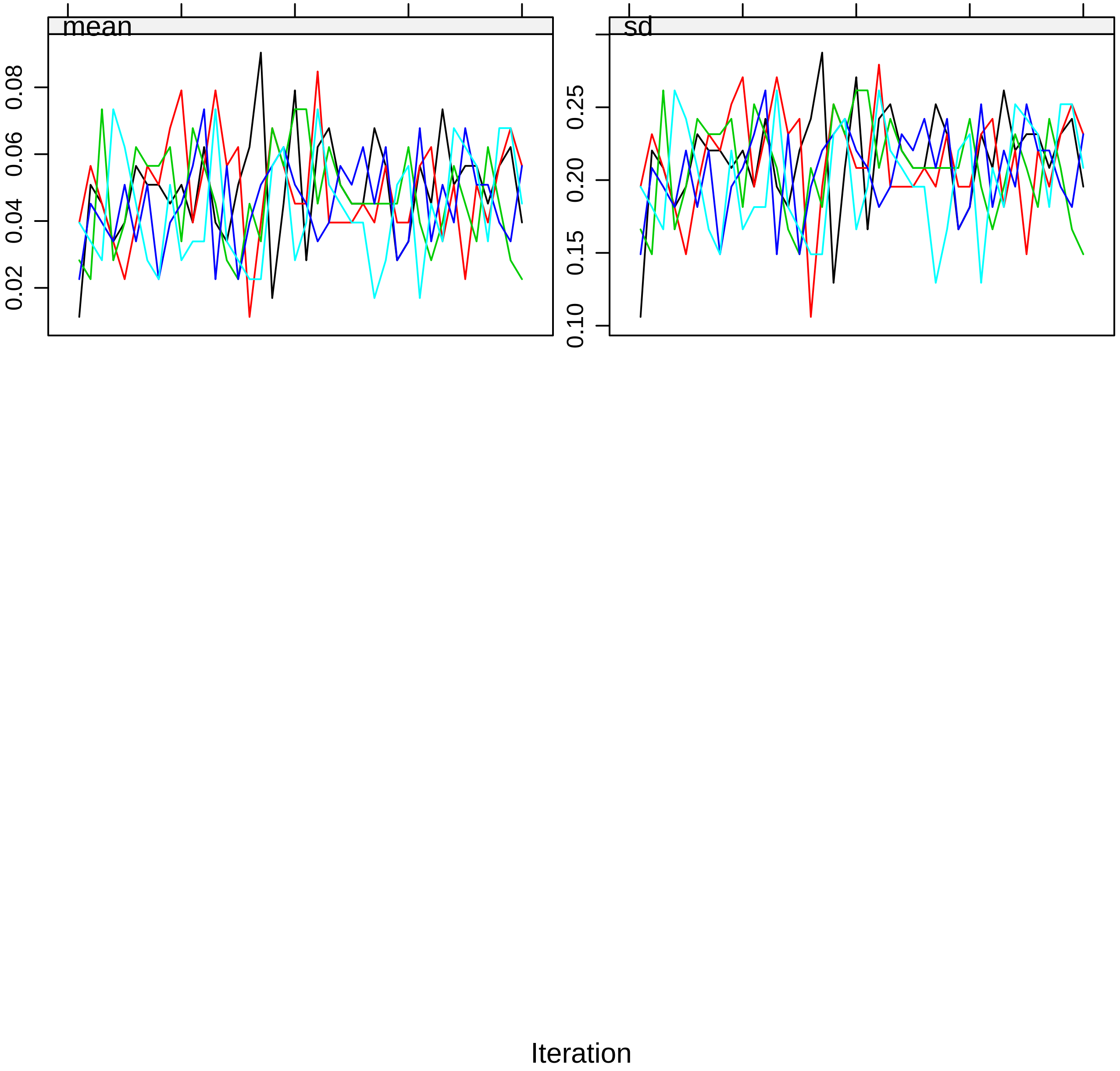

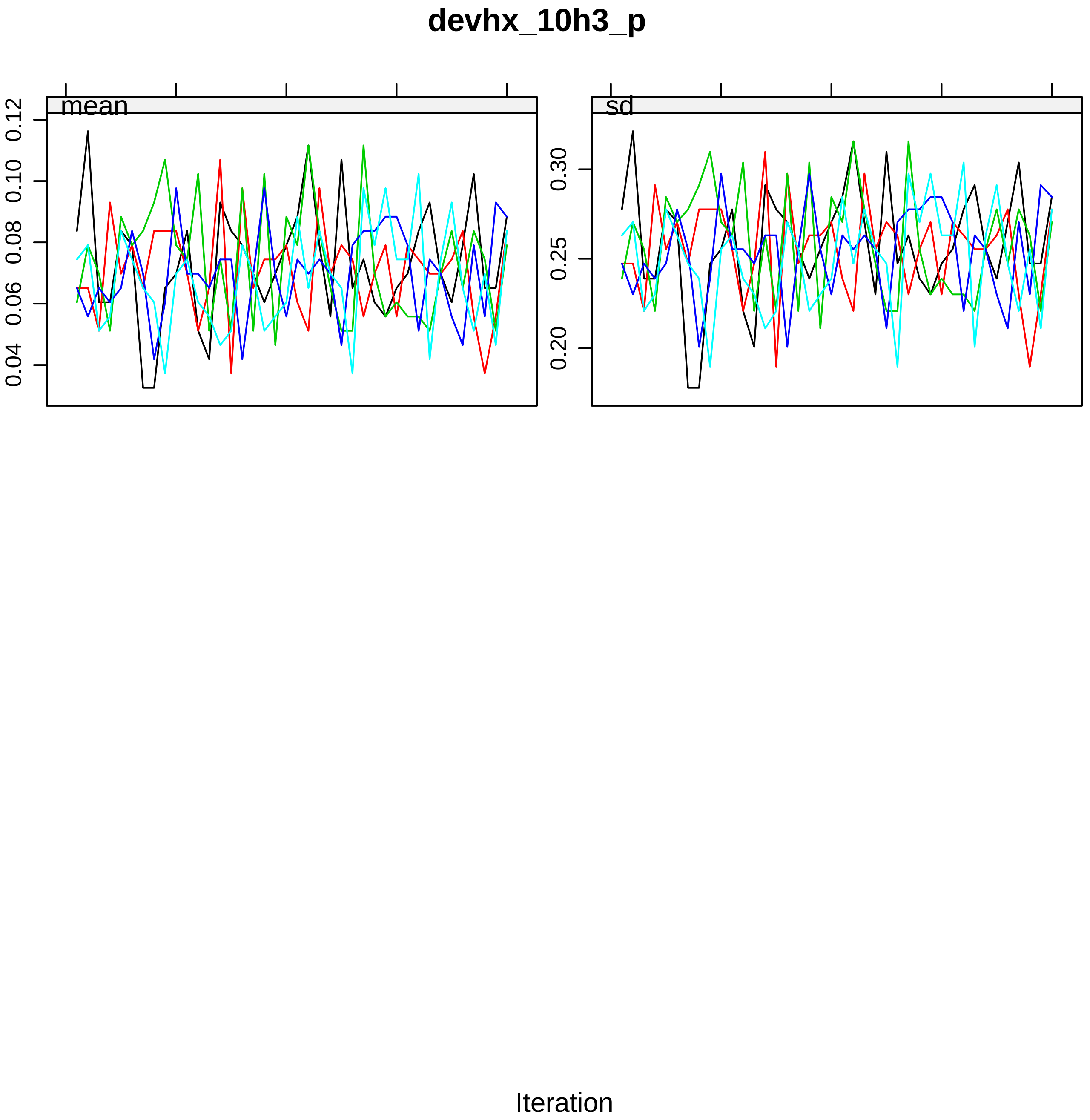
devhx_10h3_p
mean
sd
0.04
0.06
0.08
0.10
0.12
0.20
0.25
0.30
Iteration

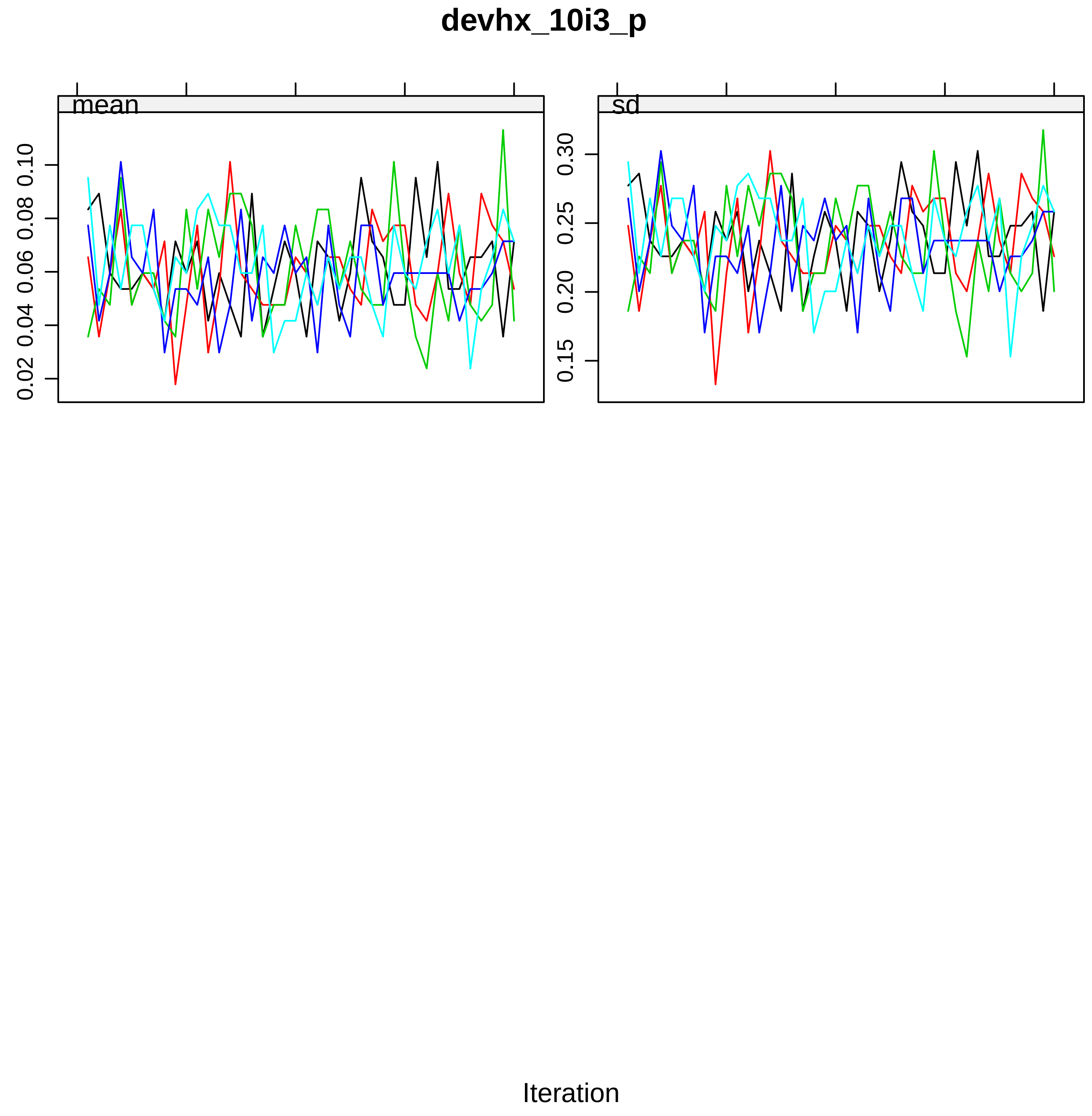

devhx_10i3_p
mean
sd
0.02
0.04
0.06
0.08
0.10
0.15
0.20
0.25
0.30
Iteration

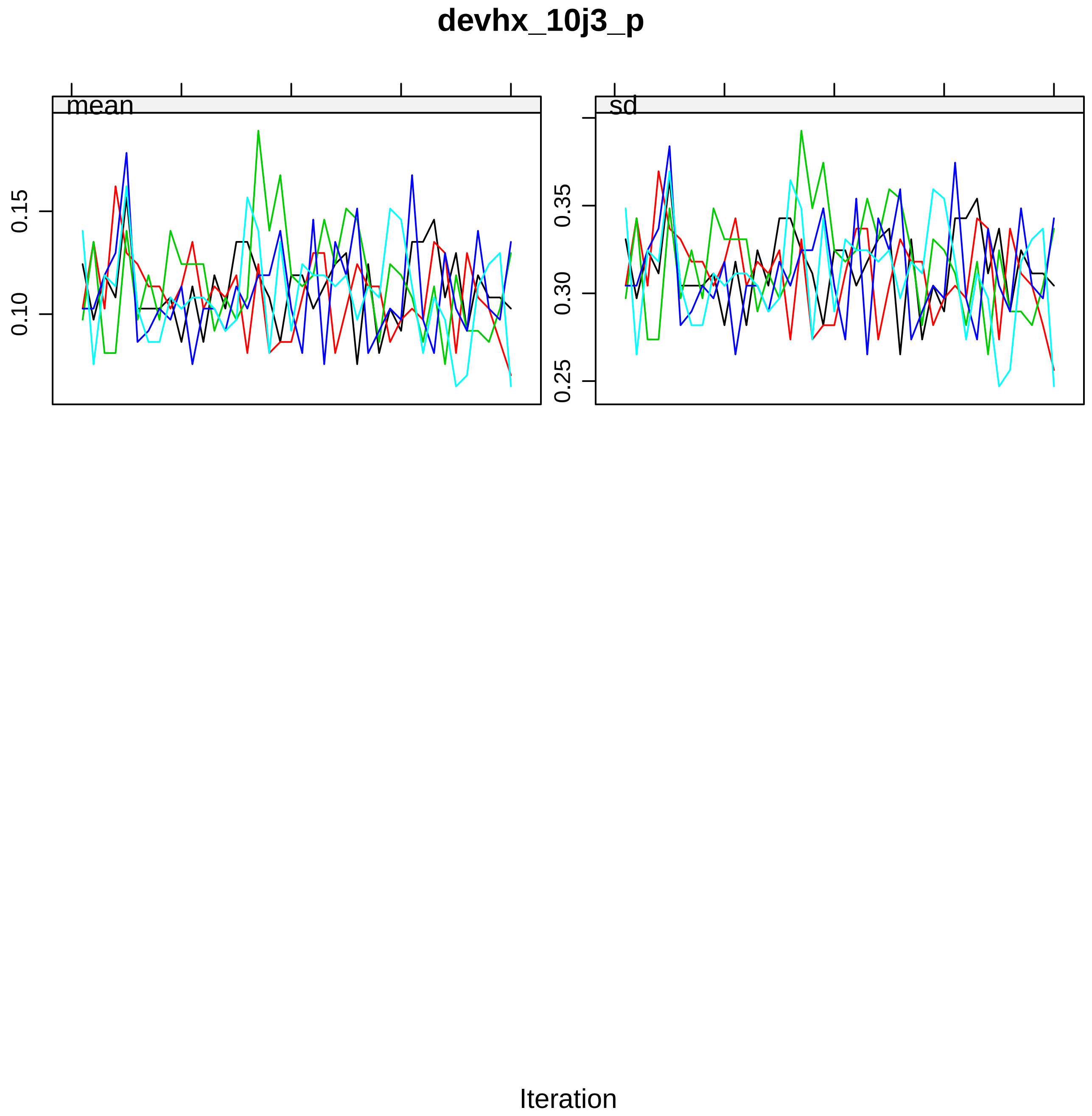
devhx_10j3_p
mean
sd
0.10
0.15
0.25
0.30
0.35
Iteration

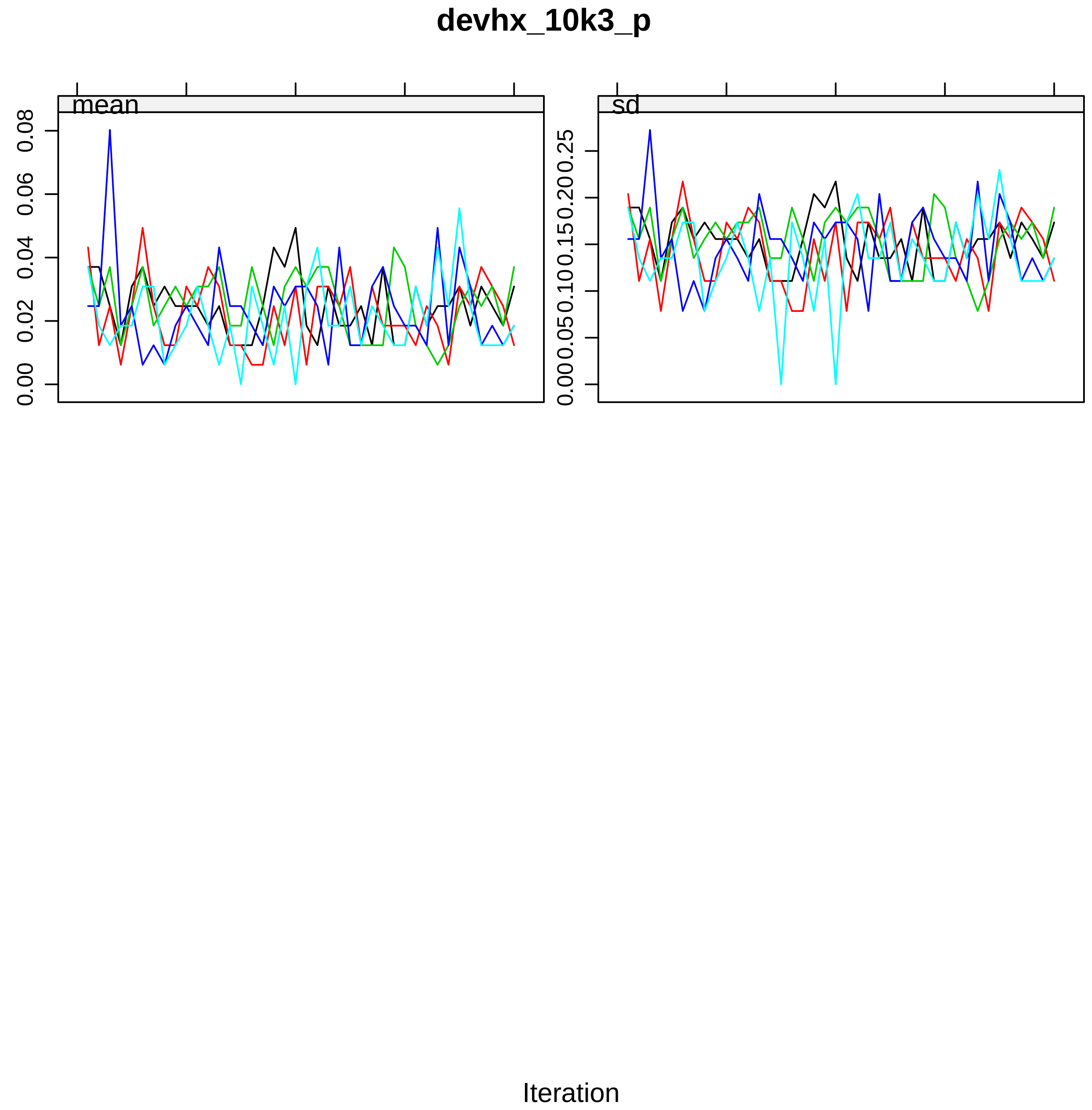
devhx_10k3_p
mean
sd
0.00
0.02
0.04
0.06
0.08
0.00
0.05
0.10
0.15
0.20
0.25
Iteration

devhx_10l3_p

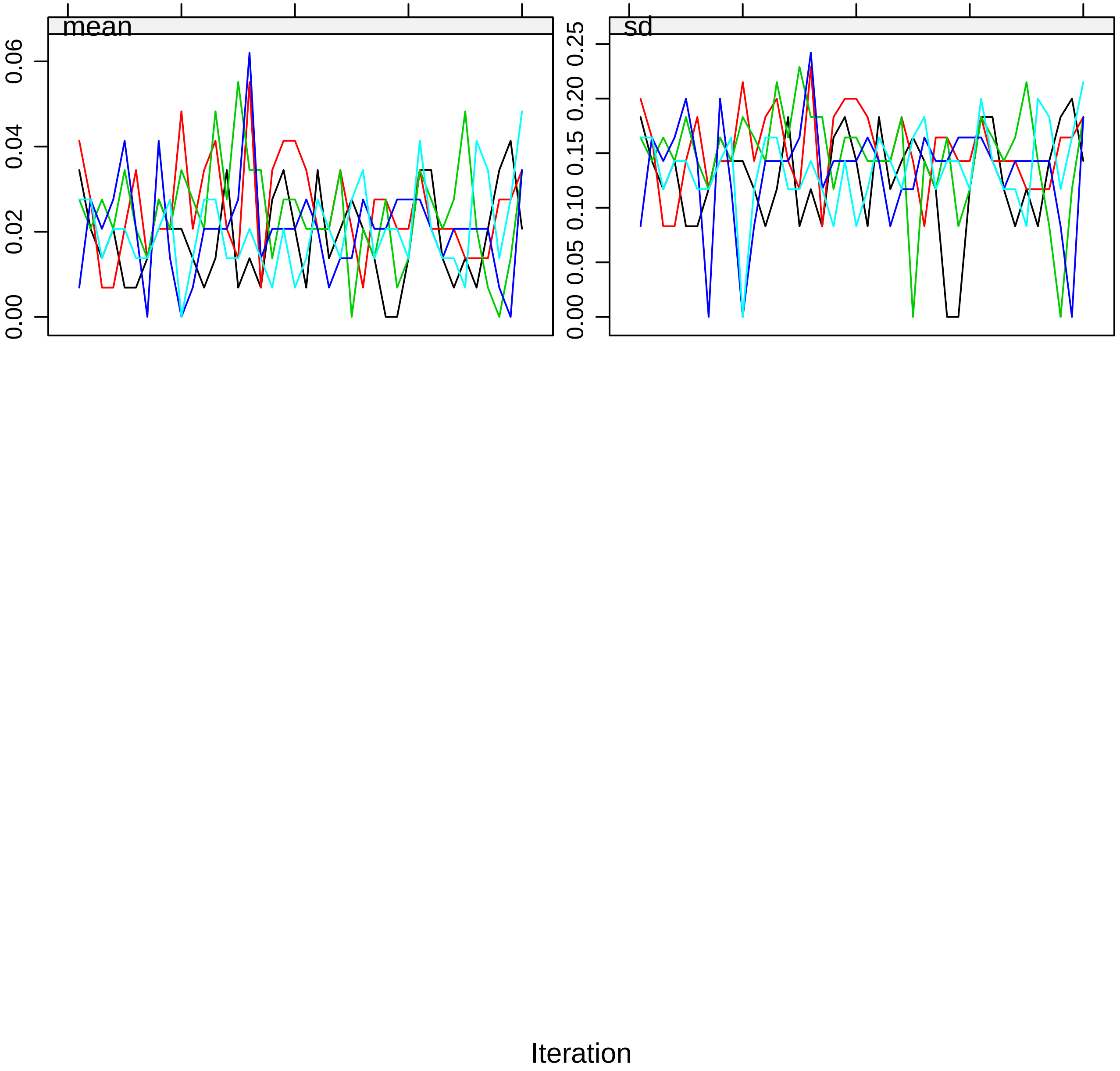

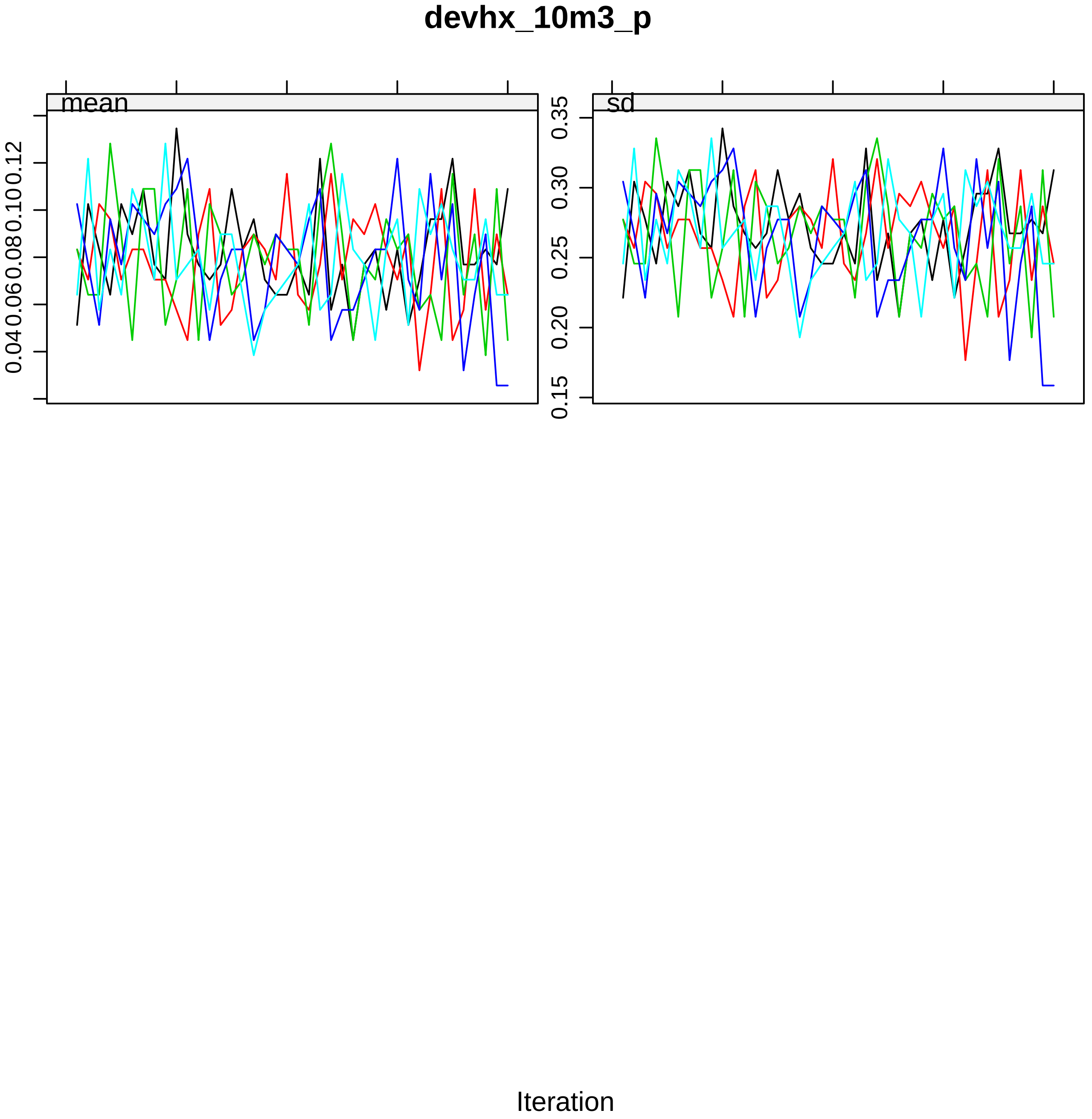
devhx_10m3_p
mean
sd
0.04
0.06
0.08
0.10
0.12
0.15
0.20
0.25
0.30
0.35
Iteration

devhx_12a_p

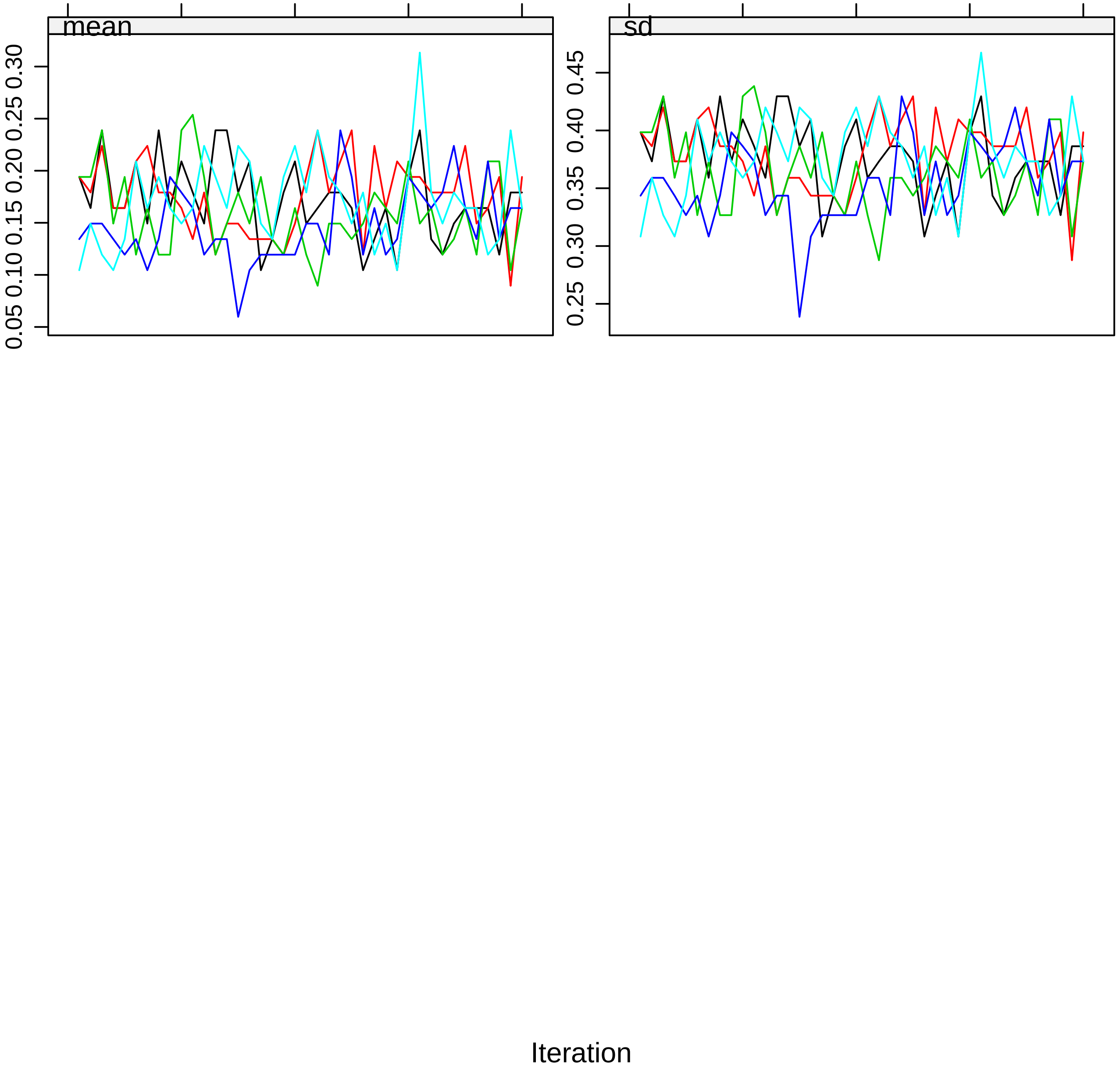

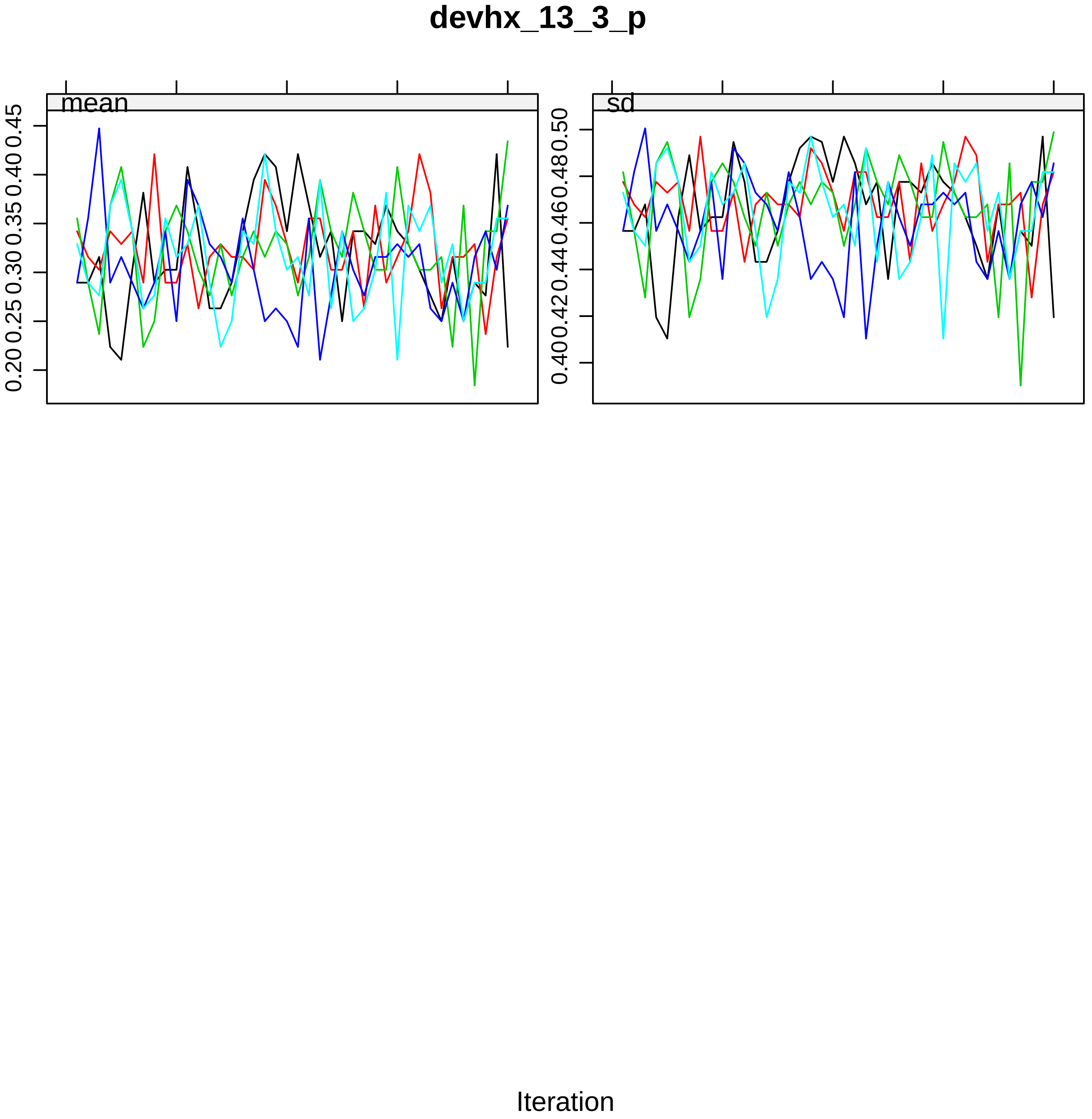
devhx_13_3_p
mean
sd
0.20 0.25 0.30 0.35 0.40 0.45
0.400.420.440.460.480.50
Iteration

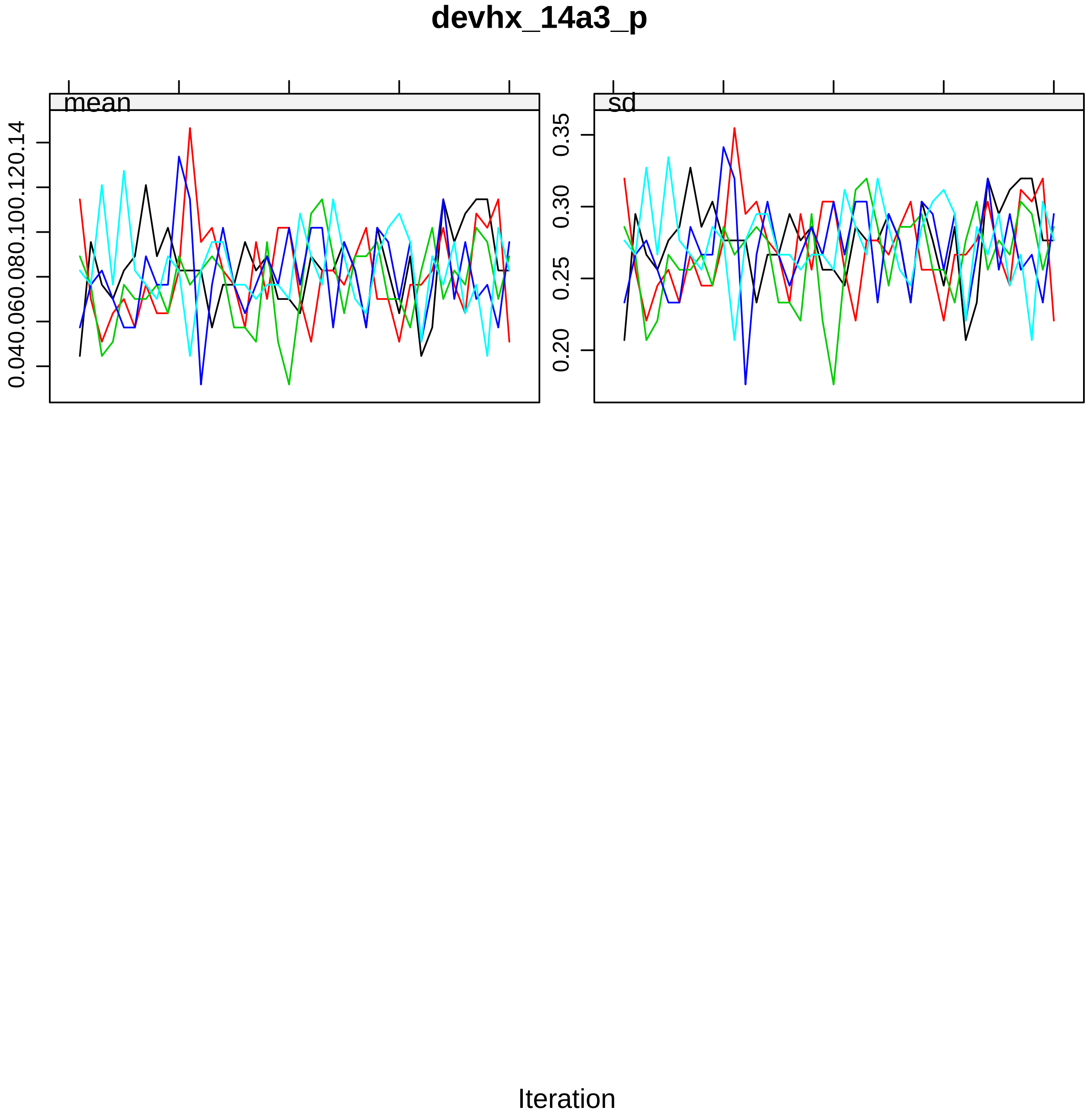
devhx_14a3_p
mean
sd
0.04
0.06
0.08
0.10
0.12
0.14
0.20
0.25
0.30
0.35
Iteration

devhx_14b3_p

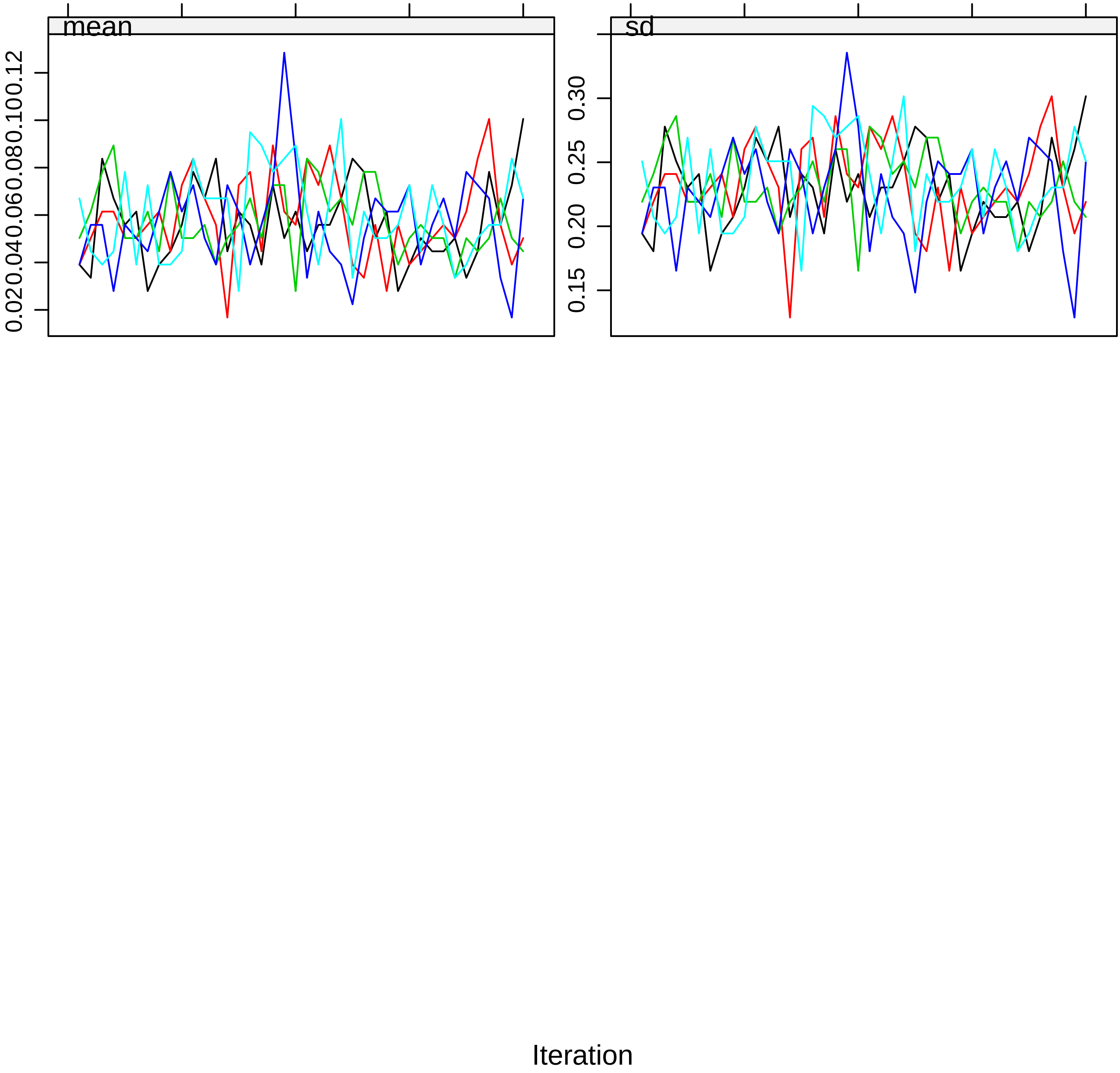

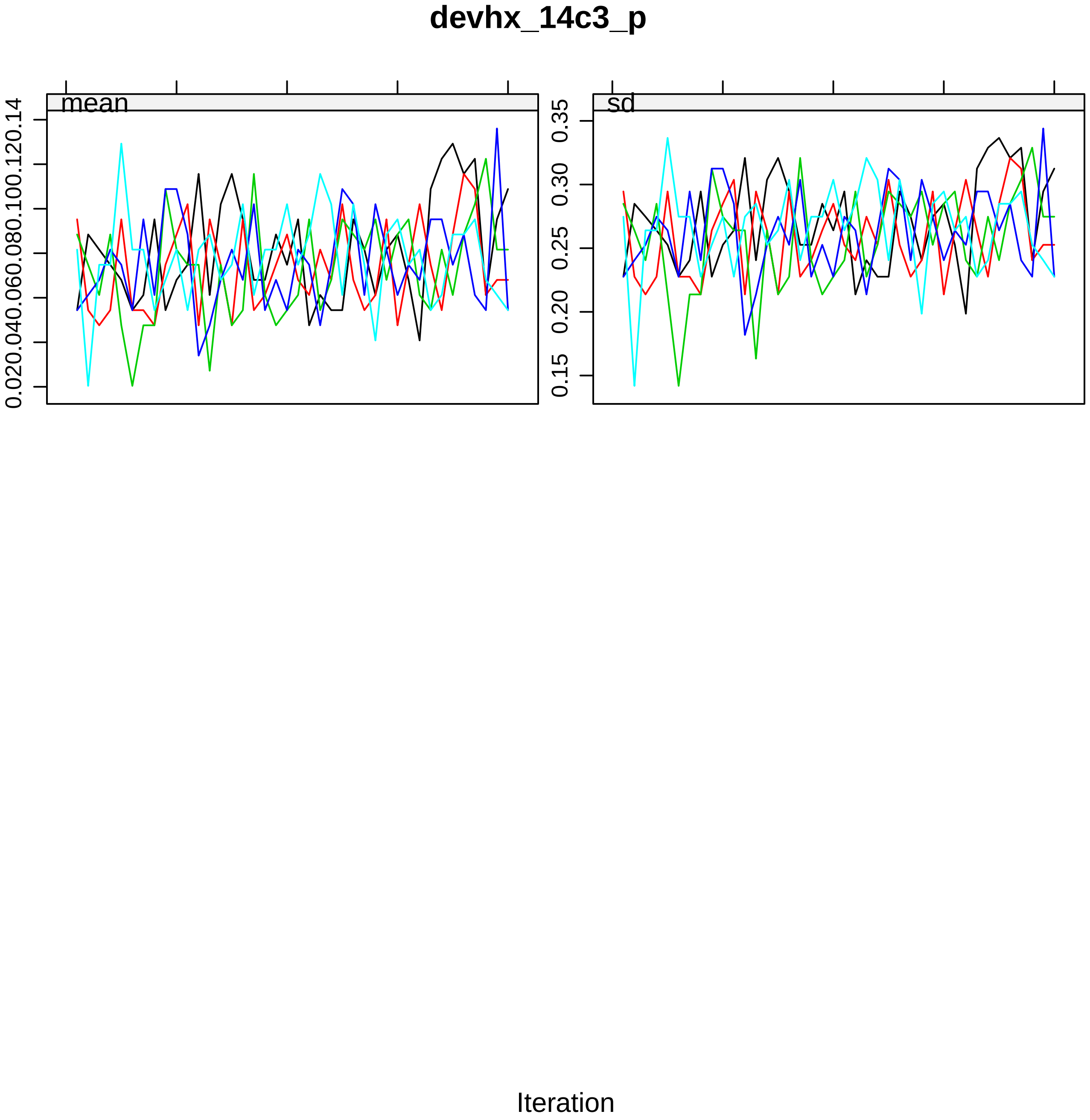
devhx_14c3_p
mean
sd
0.02
0.04
0.06
0.08
0.10
0.12
0.14
0.15
0.20
0.25
0.30
0.35
Iteration

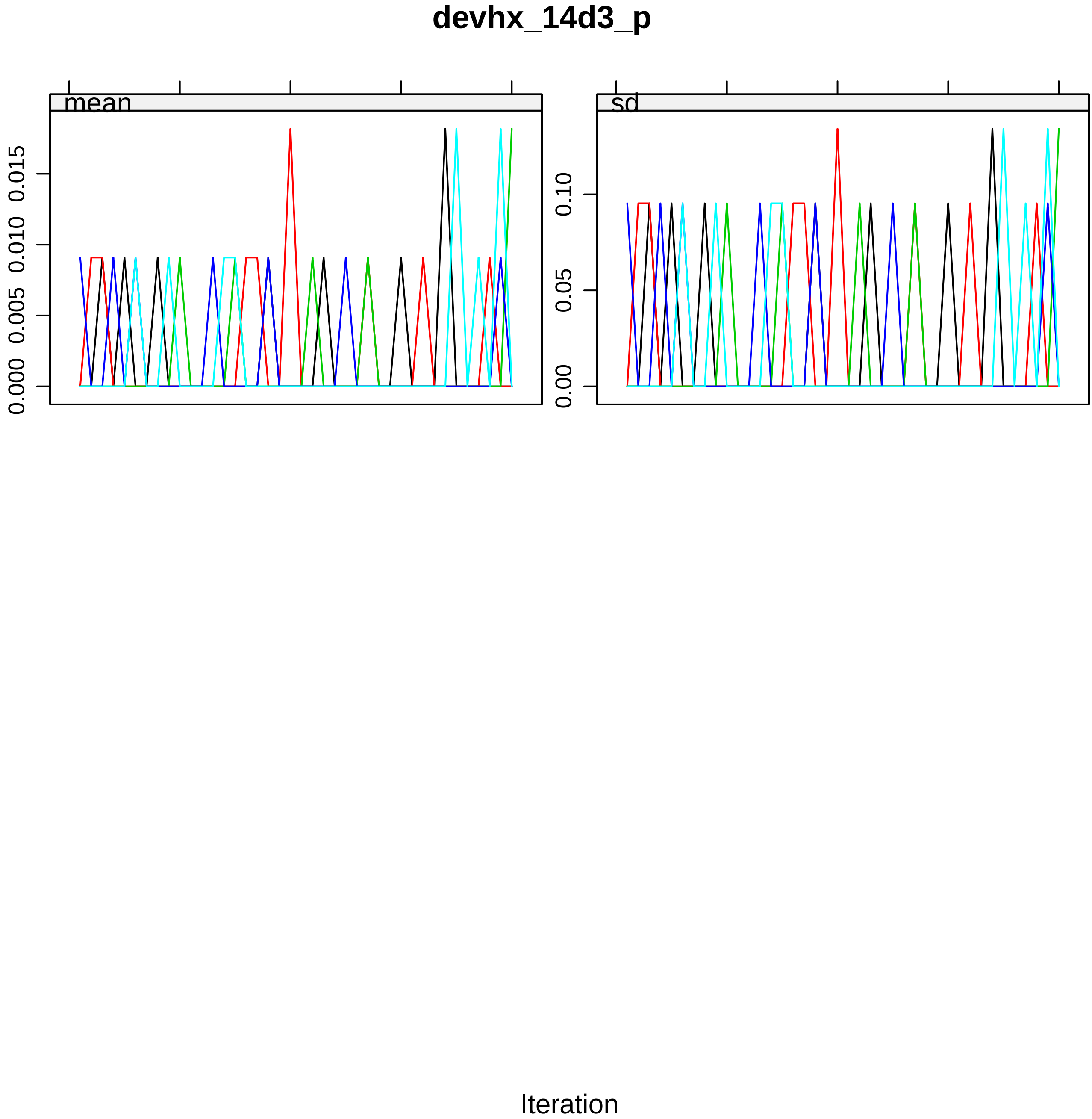
devhx_14d3_p
mean
sd
0.000
0.005
0.010
0.015
0.00
0.05
0.10
Iteration

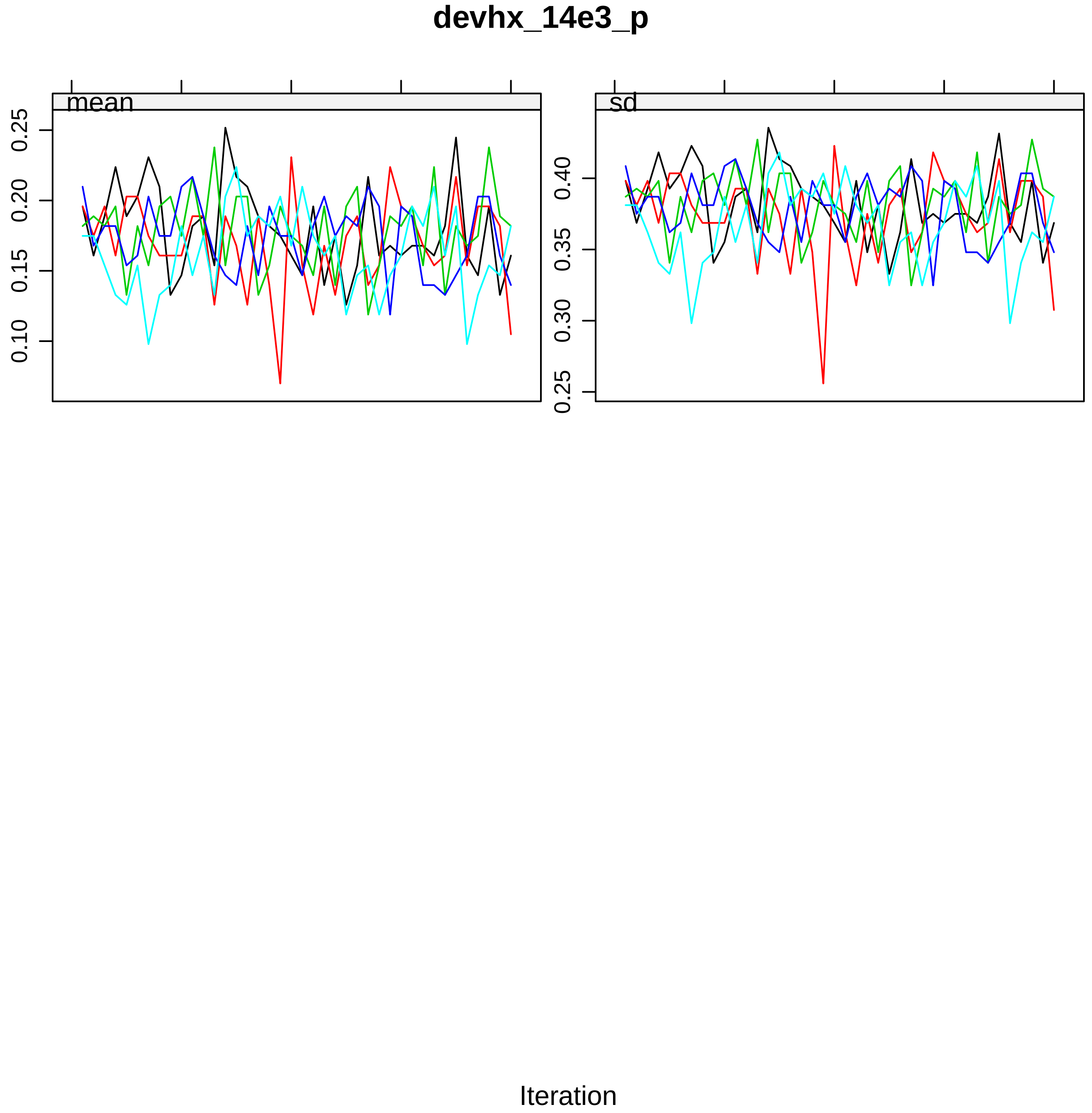
devhx_14e3_p
mean
sd
0.10
0.15
0.20
0.25
0.25
0.30
0.35
0.40
Iteration

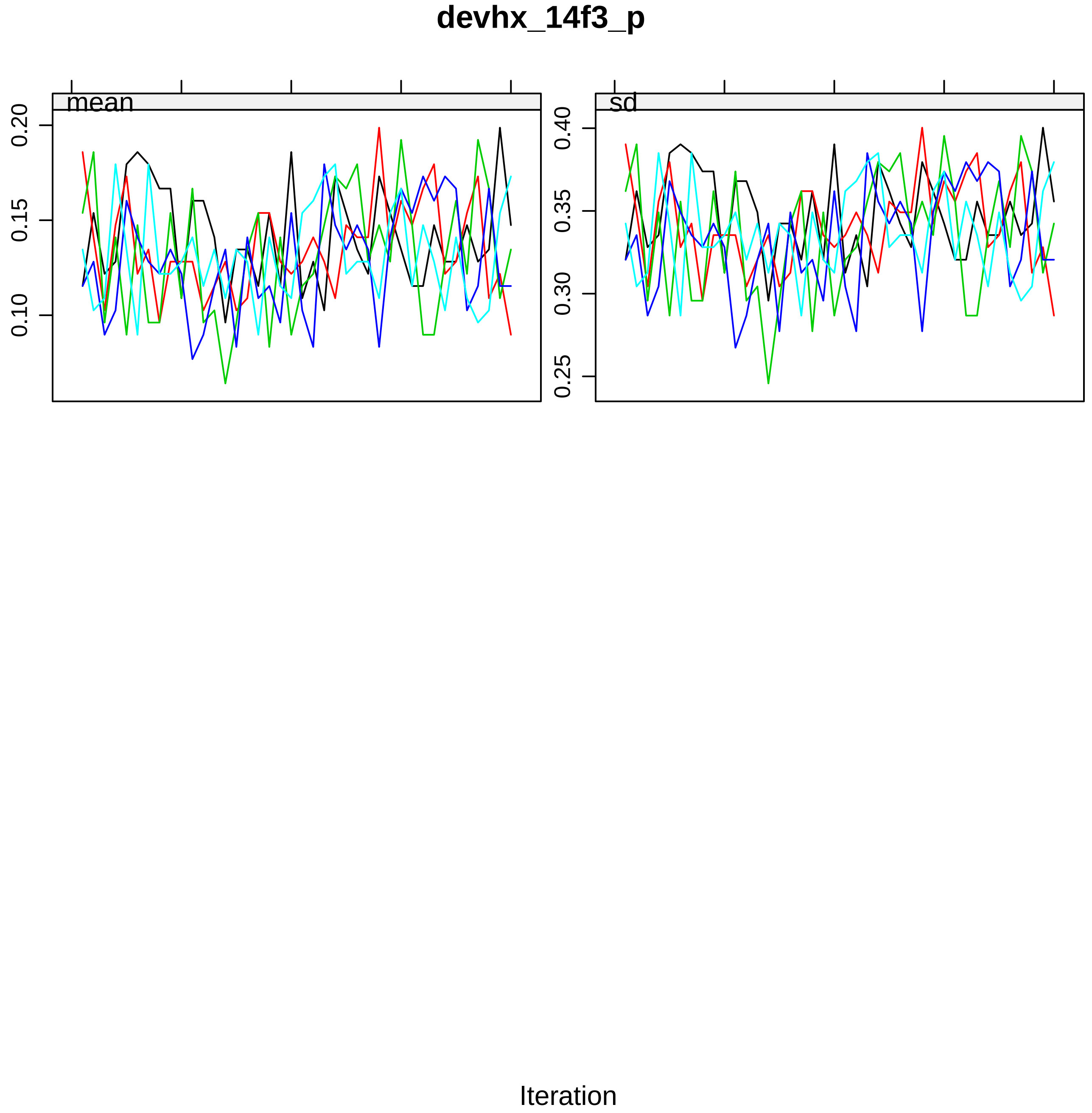
devhx_14f3_p
mean
sd
0.20
0.15
0.10
0.40
0.35
0.30
0.25
Iteration

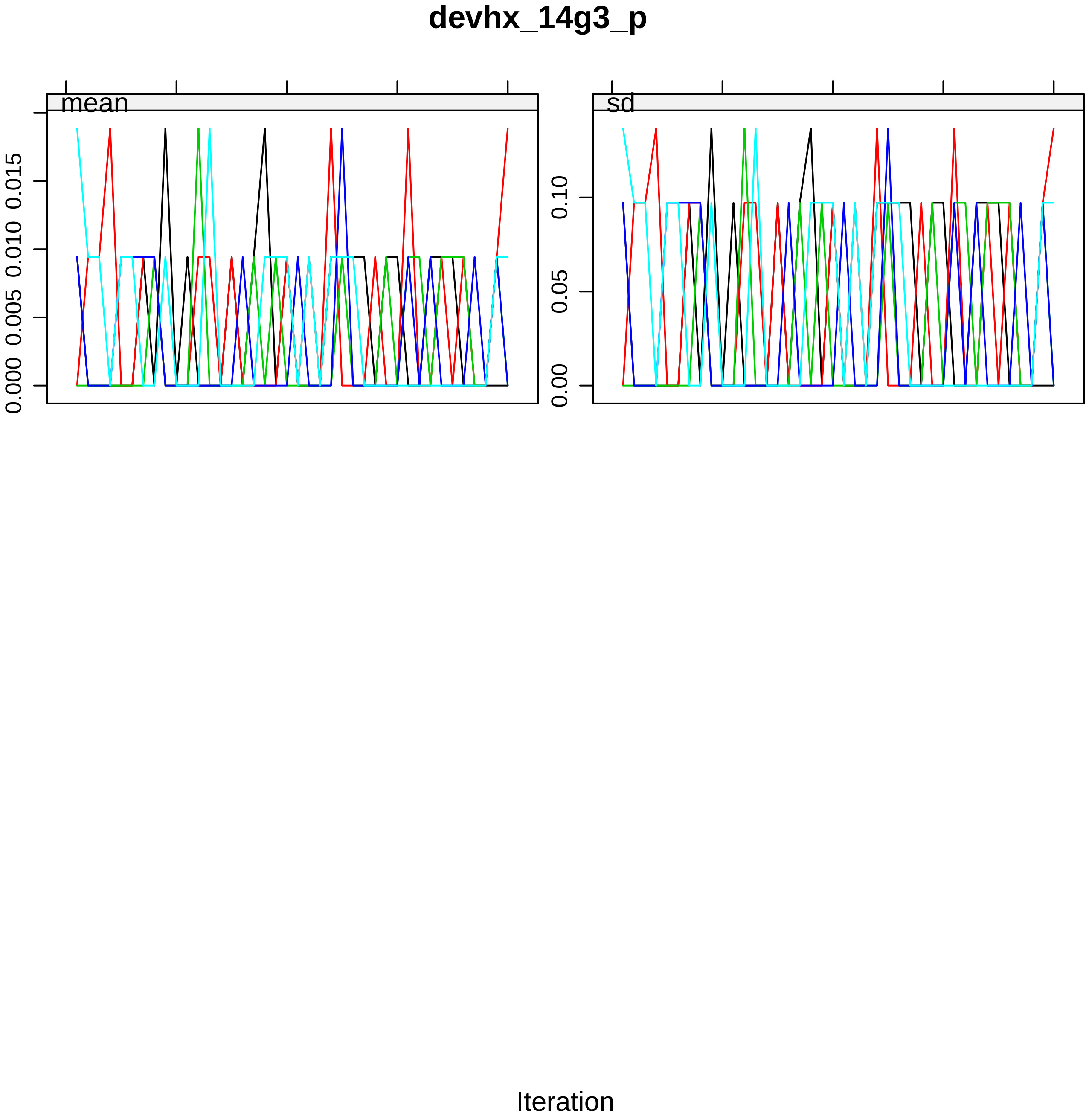
devhx_14g3_p
mean
sd
0.000
0.005
0.010
0.015
0.00
0.05
0.10
Iteration

devhx_14h3_p

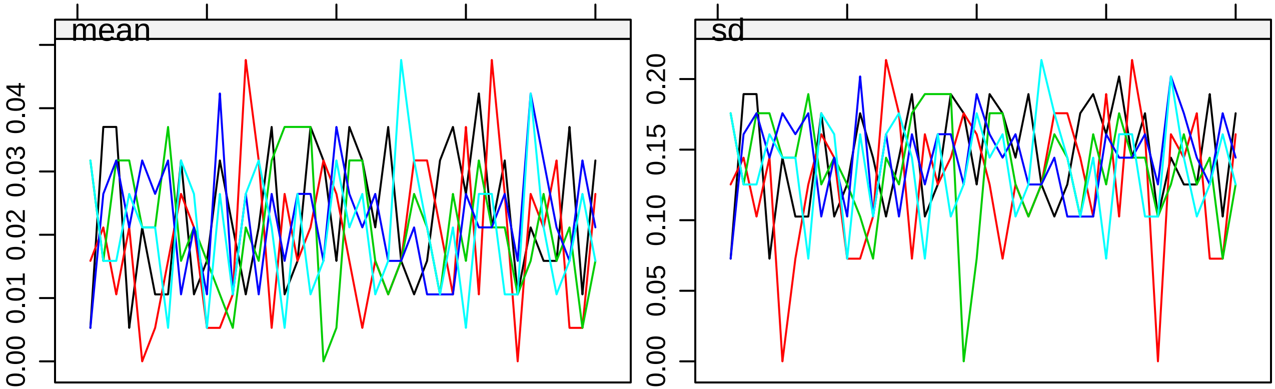

Iteration

devhx_15

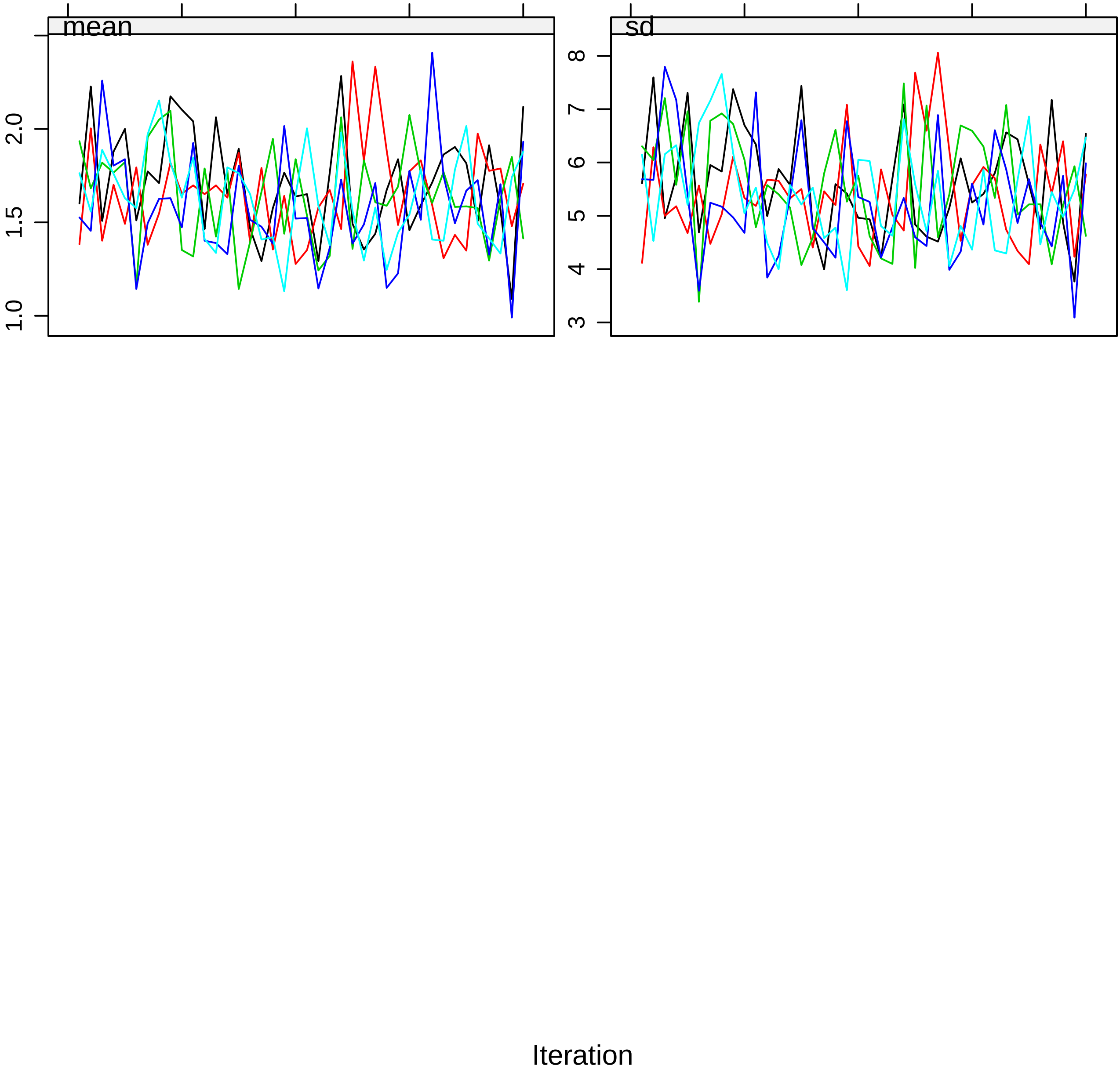

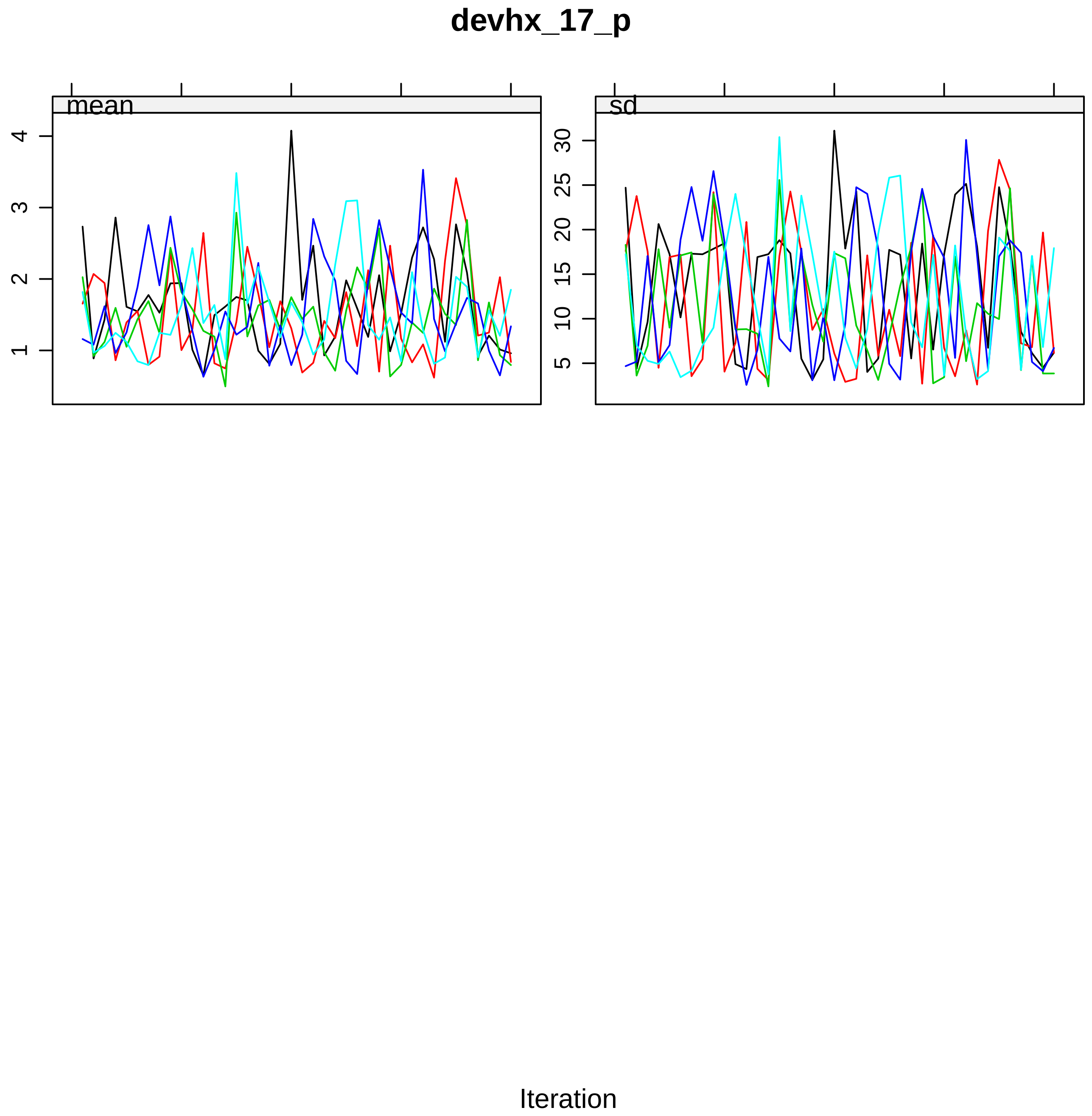
devhx_17_p
mean
sd
1
2
3
4
5
10
15
20
25
30
Iteration

devhx_18_p

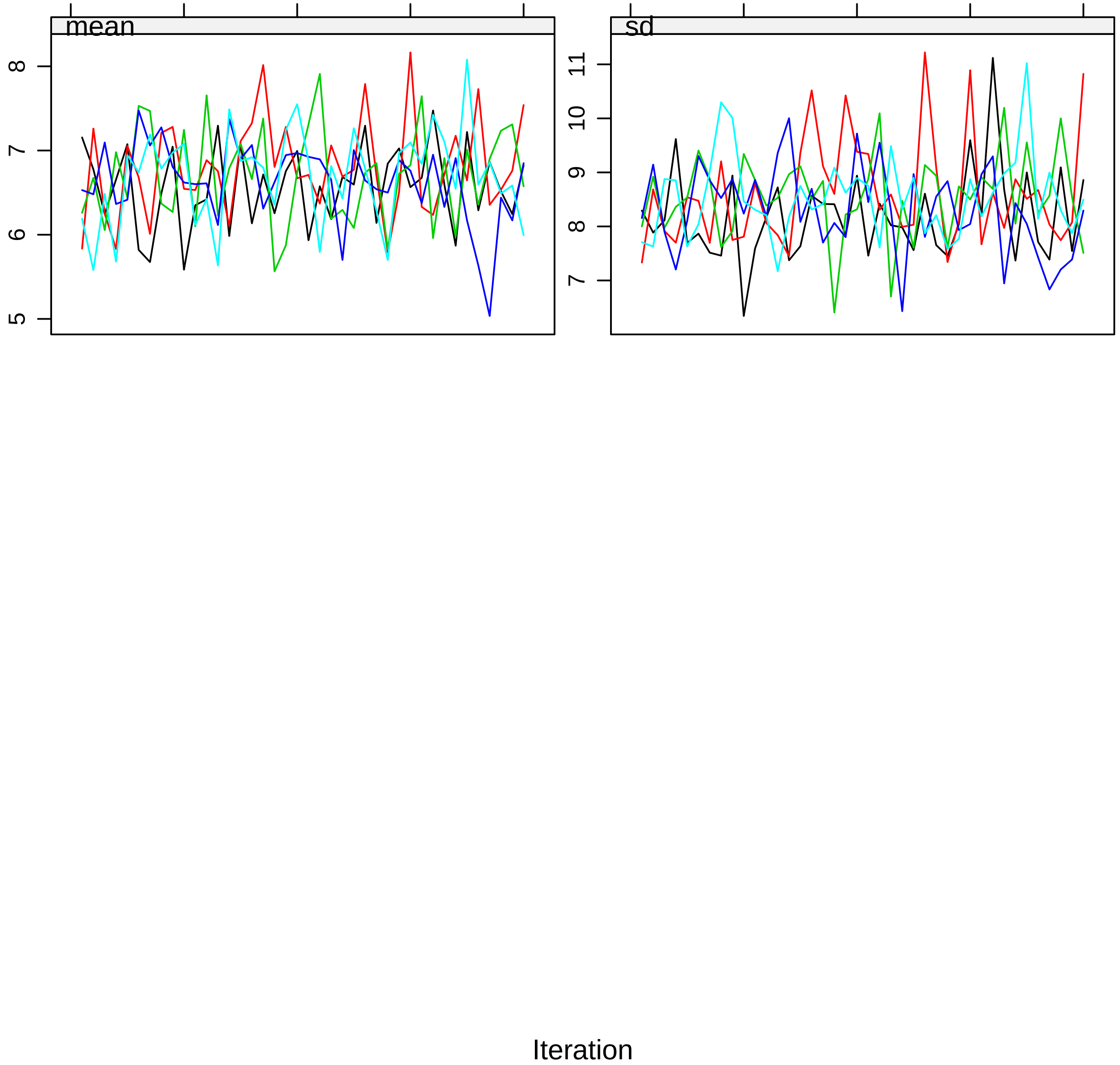

devhx_19c_p

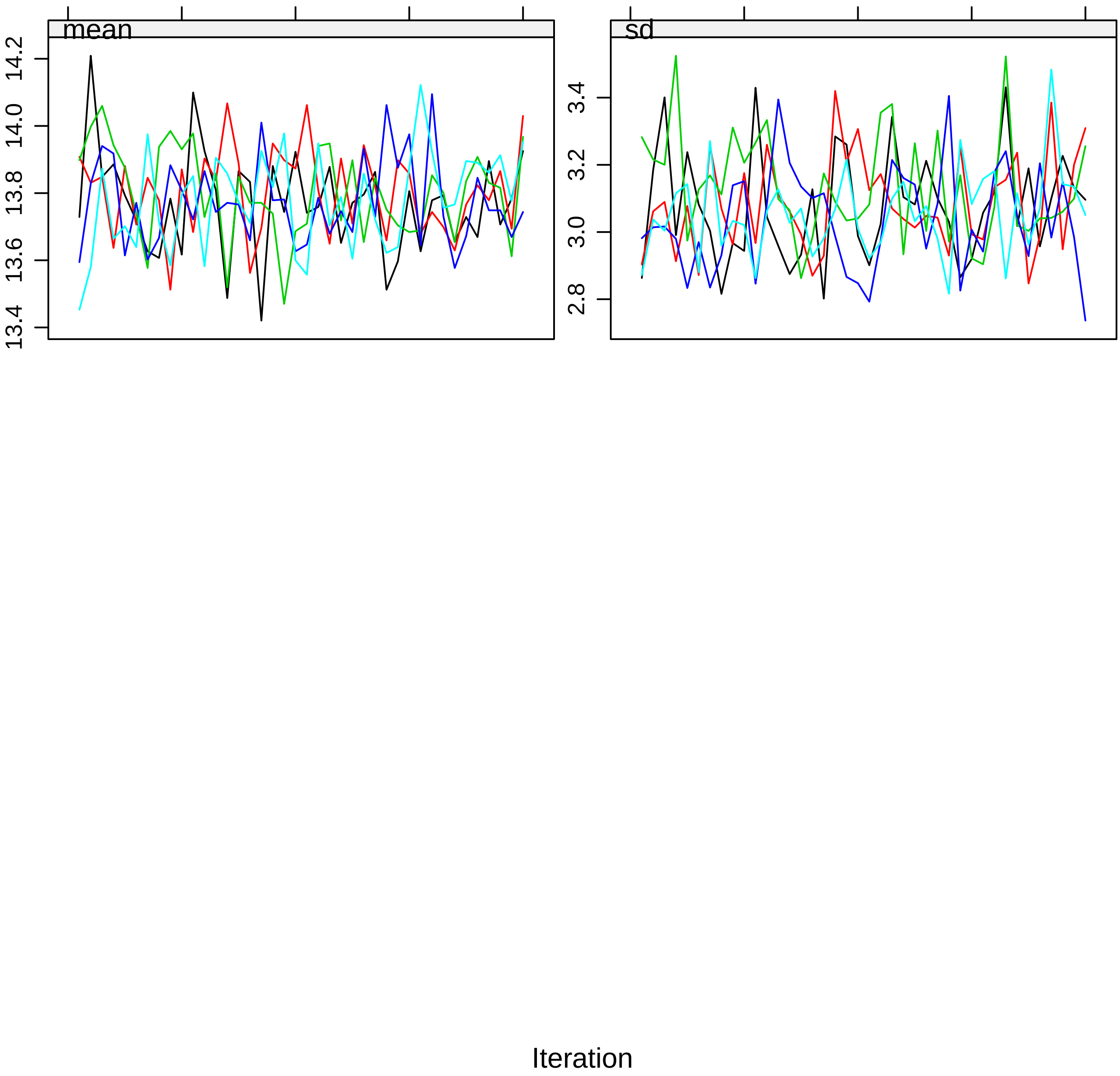

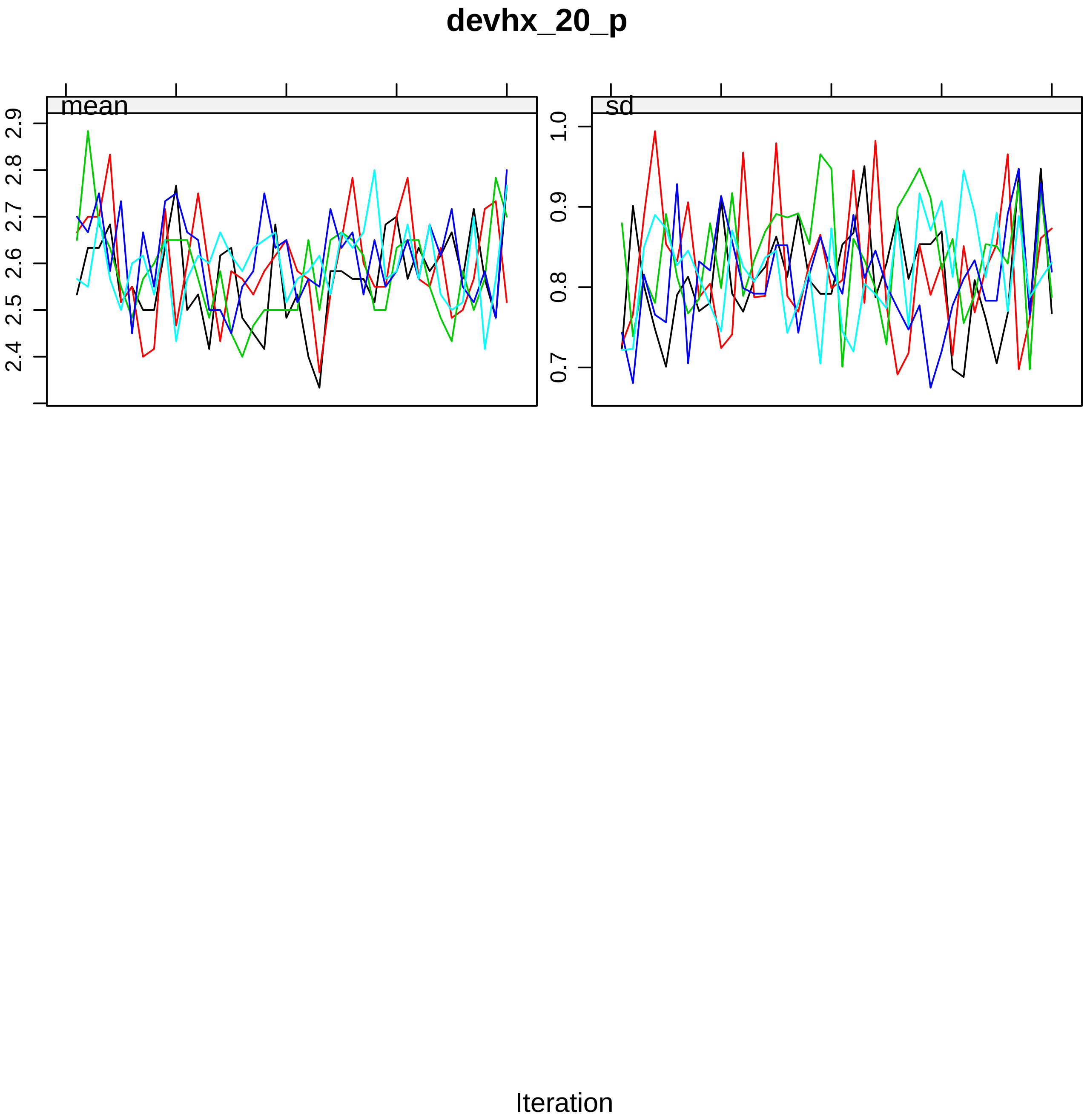

devhx_20_p
mean
sd
2.4
2.5
2.6
2.7
2.8
2.9
0.7
0.8
0.9
1.0
Iteration

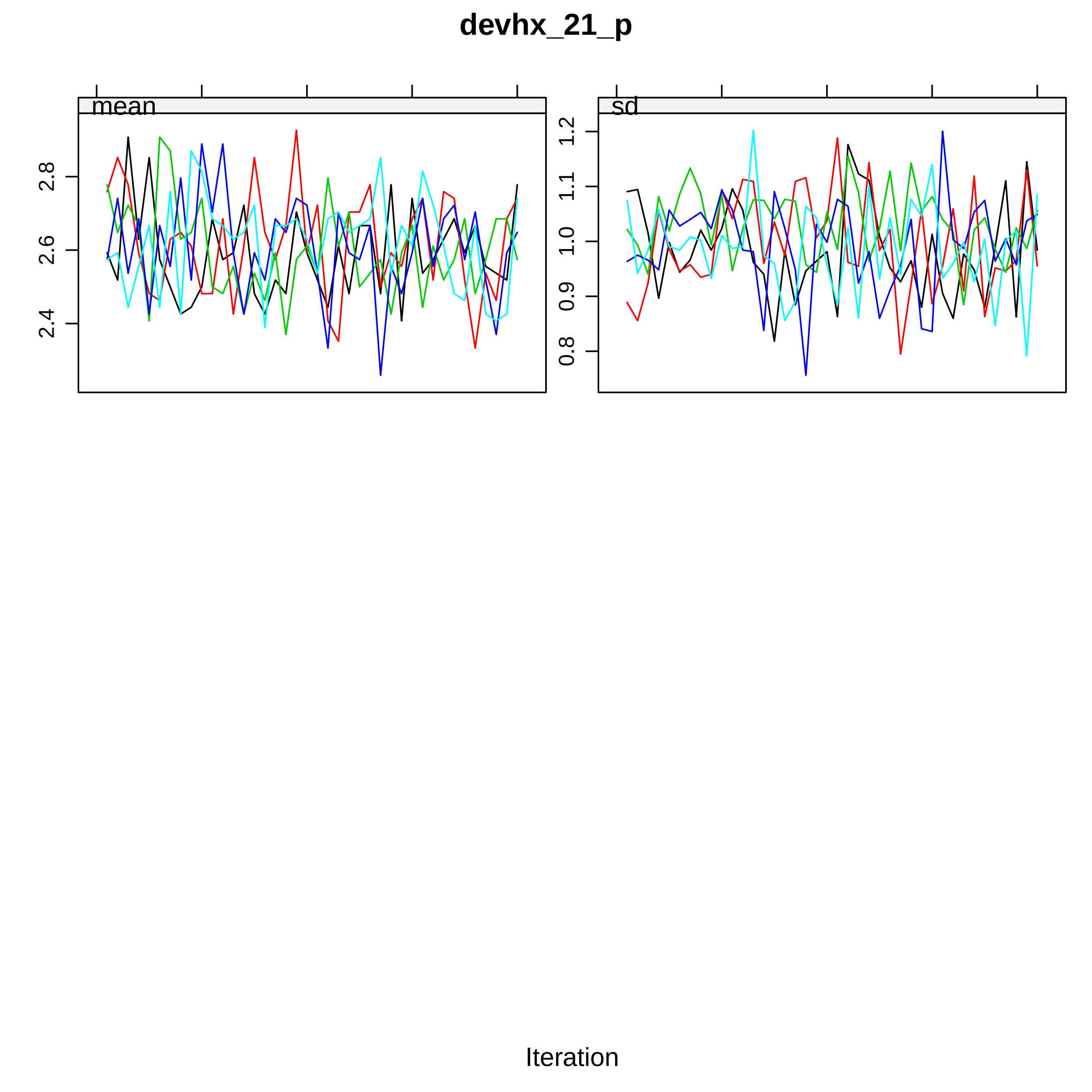
devhx_21_p
mean
sd
2.4
2.6
2.8
0.8
0.9
1.0
1.1
1.2
Iteration

devhx_22_3_p

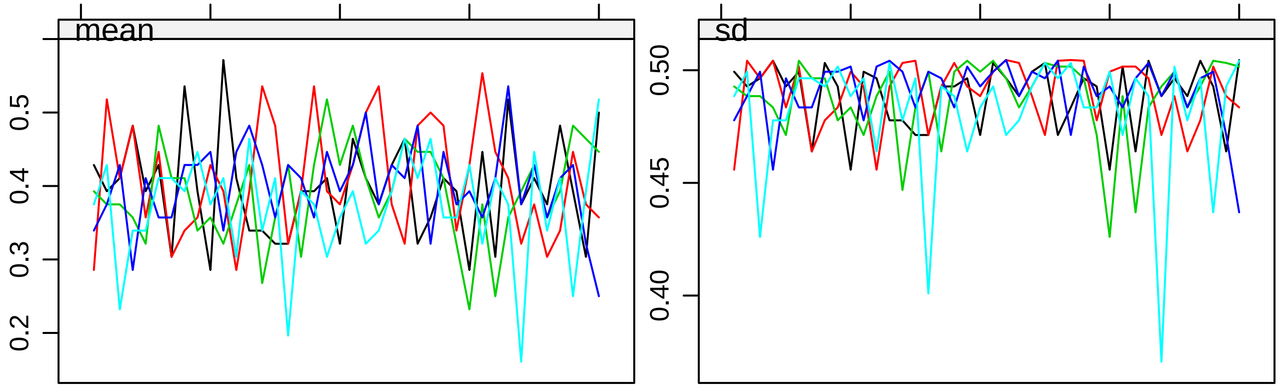

Iteration
